# Comprehensive analysis of the prognostic implications and functional exploration of PAK gene family in human cancer

**DOI:** 10.1186/s12935-022-02689-6

**Published:** 2022-09-05

**Authors:** Kunjian Lei, Min Luo, Zewei Tu, Shigang Lv, Junzhe Liu, Chuandong Gong, Minhua Ye, Miaojing Wu, Yilei Sheng, Xiaoyan Long, Jingying Li, Xingen Zhu, Kai Huang

**Affiliations:** 1 Department of Neurosurgery, The Second Affiliated Hospital of Nanchang University, Nanchang, 330006 Jiangxi People’s Republic of China; 2 Institute of Neuroscience, Nanchang University, Nanchang, 330006 Jiangxi People’s Republic of China; 3 East China Institute of Digital Medical Engineering, Shangrao, 334000 Jiangxi People’s Republic of China; 4 Nanchang University, Nanchang, 330006 Jiangxi People’s Republic of China; 5 Department of Comprehensive Intensive Care Unit, The Second Affiliated Hospital of Nanchang University, Nanchang, 330006 Jiangxi People’s Republic of China

**Keywords:** p21-activated kinase (PAK), Pan-cancer, Biomarker, Tumor immunity, Therapeutic targets

## Abstract

**Background:**

The p21-activated kinase (PAK) family (PAKs) plays a key role in the formation and development of human tumors. However, a systematic analysis of PAKs in human cancers is lacking and the potential role of PAKs in cancer immunity has not been explored.

**Methods:**

We used datasets from in The Cancer Genome Atlas (TCGA) database and Genotype-Tissue Expression database (GTEx).

**Results:**

Based on TCGA datasets most PAKs show noteworthy differences in expression between tumors and corresponding normal tissues or across different tumor tissues. Patients with high expression of PAKs often show a worse prognosis. However, copy number variation, mutation, and DNA methylation of PAKs have limited impact on tumor development. Further analysis showed that the impact of PAKs on immunity varies with the type of tumor and the respective tumor microenvironment. PAK1 and PAK4 may be stronger predictors of immune characteristics, and are more suitable as drugs and molecular therapeutic targets. Furthermore, Cox regression analysis revealed that a PAK gene signature could be used as an independent prognostic factor for lower grade glioma (LGG) and glioblastoma (GBM). Gene set enrichment analysis (GSEA) analysis indicated that PAK genes may affect the occurrence and development of GBM through the PI3K signaling pathway. Further experiments verified that PAK1 and AKT1 have a significant interaction in GBM cells, and inhibiting the overactivation of PAK1 can significantly inhibit the proliferation of GBM cells.

**Conclusions:**

Our study provides a rationale for further research on the prognostic and therapeutic potential of PAKs in human tumors.

**Supplementary Information:**

The online version contains supplementary material available at 10.1186/s12935-022-02689-6.

## Background

The remarkable characteristics of cancer cells include disorder of cell homeostasis, uncontrollable cell proliferation, escape from apoptotic signals, dysregulated gene products, and dynamic changes in the cytoskeleton [1]. Several protein kinases have been identified as driving factors of human carcinogenesis and development, and several oncogenic kinases have been successfully targeted by drugs, but more therapeutic targets and drugs still need to be explored because of universal and established drug resistant mechanisms [2]. Growing evidence has shown that serine/threonine kinases of the p21 activated kinase (PAK) family play a significant role in above processes [3, 4]. PAK gene family members (PAKs) are upregulated or overactivated in various human cancers, including pancreatic, breast, colorectal, ovary, and thyroid cancer [5–10]. Therefore, a comprehensive analysis of the expression and variation of PAKs expression in tumor tissues may allow their adoption as diagnostic biomarkers and therapeutic targets in the future.

The six known mammalian PAKs can be divided into two groups according to their structure and sequence: Group I comprises PAK1, PAK2, and PAK3; Group II includes PAK4, PAK7 (also known as PAK5), and PAK6 [11–13]. The members of the PAK family each have a unique expression pattern. High-level expression of PAK1 is found in in embryogenesis and some adult tissues, including the brain, muscle, and spleen [14, 15]. PAK4 is expressed at a high level during embryonic development [16]. Other members of the PAK family are also highly expressed in the nervous system [15]. In human tumors, the overexpression and overactivation of PAKs kinases, especially PAK1 and PAK4, are usually associated with cancer, because both kinases are found to be elevated at DNA, RNA, or protein levels in many types of cancer [2]. In addition, other members of the PAK family are also associated with some cancers [2]. Interestingly, members of the PAKs family are less subject to mutation but are mainly linked to cancer through overexpression and overactivation. Therefore, detecting the expression level of PAKs in tumor tissues may be more suitable as a diagnostic biomarker for cancer. In pan-cancer, PAKs are believed to be associated with abnormal cell proliferation, cell signal changes, enhanced migration ability, drug resistance, and immune system regulation [2, 15, 17]. Numerous studies have demonstrated that PAKs mediate a series of cancer-related signaling pathways, including cell migration, the PAK-PI3K/AKT, and PAK-Wnt/β-catenin, kinase-independent signaling pathways, in addition to Cdc42 independent PAK functions [18–23]. However, there have been no relevant studies analyzing the influence of amplification, mutation, and methylation of PAK genes on carcinogenesis and tumor progression, immunity, and drug resistance. A comprehensive analysis of the prognostic and functional implications of the PAK gene family may provide valuable information to further expand the application of therapy targeting PAKs in a wider range of cancers.

In this study, we extracted data from TCGA and GTEx databases to conduct a comprehensive analysis of tumor and normal samples of 28 cancers we: (1) investigated the expression comparison of PAKs in tumor tissues and normal tissues; (2) detected and compared PAKs copy number variation (CNV), methylation, mutation, and deletion in 28 tumors in the TCGA database, and their impact on the prognosis of these patients; (3) performed a comprehensive prognosis analysis through combining the survival data and expression data of each tumor patient in The Cancer Genome Atlas (TCGA) database; (4) performed a comprehensive analysis of the correlation of PAK family with immune cells and immune genes in human cancers; (5) performed modeling and detailed prognostic analysis for low-grader glioma (LGG) and glioblastoma (GBM) patients, and GSEA analysis for PAK1 in GBM patients; and (6) using GBM cell lines we identified the mechanisms involving PAK1 regulation of cell development through PI3K signaling pathway. Taken together, these data cast important new insights into the nature of PAK1 dysregulation in cancer, including its possible causes and potential consequences. By revealing susceptible cancer types and identifying potential biomarkers for therapeutic research, the proposed research results provide a rationale for targeting of PAKs in cancer.

## Methods:

### Data source and processing

The gene expression data of PAKs in 28 human tumors and corresponding normal samples (Table 1) were downloaded from TGGA portal (http://cancergenome.nih.gov/) and the Genotype-Tissue Expression database (GTEx, https://gtexportal.org/home/datasets). Clinical details and data relative to DNA methylation, mutation status, and CNV of tumor patients were obtained from the UCSC Xena website (https://xenabrowser.net/datapages/). For data acquired from TCGA database, we converted fragments per kilobase million (FPKM) values into transcripts per million (TPM) values, and displayed PAKs expression data in the form of log2 (TPM + 1). The PAKs mRNA expression levels and z-scores of cell sensitivity data in 59 cell lines were extracted from the NCI-60 database through the CellMiner interface (https://discover.nci.nih.gov/cellminer/), and Pearson’s correlation was performed to investigate the relationship between gene expression and drug sensitivity of 262 drugs that are on trial or approved by the US FDA. Four LGG cohorts, including two Chinese Glioma Genome Atlas (CGGA) cohorts (CGGA1 and CGGA2), the GSE16011 cohort, and the Rembrandt cohort, were selected as the combined validation set for the PAKs prognostic model. From the CGGA website (http://www.cgga.org.cn/), mRNA expression data and clinical information relative to the CGGA1 and CGGA2 cohorts were downloaded and we obtained the microarray data and relevant clinical information of GSE16011 and Rembrandt datasets from the Gene Expression Omnibus (GEO) repository (https://www.ncbi.nlm.nih.gov/geo/). We downloaded the mRNA expression data of all tumor cell lines PAK1 and AKT1 from the CCLE database (https://portals.broadinstitute.org/ccle/data), and also obtained the protein expression matrix of AKT, AKt pS473 and Akt pT308.

### Gene set cancer analysis and GEPIA2 databases

The Gene Set Cancer Analysis (GSCA**,** http://bioinfo.life.hust.edu.cn/GSCA/) database tool can be utilized for genome and immune genome cancer analysis. GEPIA2 (http://gepia2.cancer-pku.cn/#index) is a powerful database that provides functional notation, tumor/normal differential expression analysis, patient survival analysis, and correlation analysis. The GSCA database was used for a variety of analyses, including survival difference between CNV groups, survival differences between high and low methylation status in different cancers, correlation analysis between methylation and mRNA expression, correlation between Genomics of Drug Sensitivity in Cancer (GDSC) drug sensitivity and mRNA expression, and correlation between Cancer Therapeutics Response Portal (CTRP) drug sensitivity and mRNA expression[24, 25]. According to the survival map of GEPIA2, 28 human tumors were divided into high and low expression groups in an attempt to analyze survival and disease-free survival (DFS) analysis, based on the median value of PAKs gene expression, which were displayed in the form of log2 (TPM + 1) (Additional file 1).

### NCI-60 analysis

Through the CellMiner interface (https://discover.nci.nih.gov/cellminer/), data from 60 different cancer cell lines and data from the nine different types of tumors in the NCI-60 database were extracted. We retrieved the PAKs mRNA expression level and cell sensitivity data z score (GI50) of 59 cell lines. Appling Pearson’s correlation analysis to the drug responses of 262 United States Food and Drug Administration (FDA) approved or clinical trial drugs to explore the correlation between PAKs expression and drug sensitivity.

### The human protein atlas

Images screened from The Human Protein Atlas (HPA, www.proteinatlas.org) were used to verify the PAK protein expression levels[26]. Two cancers with significant differences in the expression of PAK were selected from the database. The obtained images included PAK1 from glioma samples and PAK2 from prostate cancer samples (Additional file 2).

### Construction and verification of the PAKs signature

Least absolute shrinkage and selection operator (LASSO) regression analysis was performed to establish a prognostic signature based on the gene expression of PAKs of LGG patients in TCGA database, and to obtain an equation for calculating the risk score of LGG patients:

$$risk score={\sum }_{i=1}^{n}{Coef}_{i}*{x}_{i}$$

where, ${Coef}_{i}$ and ${x}_{i}$ are the coefficient and mRNA expression of each PAK family member, respectively (Additional file 25: Table S1).

Four verification datasets, CGGA1, CGGA2, GSE16011, and Rembrandt, were used to verify the prognostic ability of the PAKs signature in LGG patients, and the relevant patient information for these datasets is shown in Additional file 26: Table S2. For these four datasets, the risk score of each LGG patients was calculated based on the risk score formula in the TCGA dataset, and patients of each dataset were divided into the high- and low-risk subgroups based on the median value of risk score. We constructed Kaplan–Meier (K–M) survival curves to detect differences between high- and low-risk subgroups of the five cohorts.

In the training and verification datasets, the predictive ability of the risk score for overall survival (OS) were estimated using time-dependent receiver operating characteristic (ROC) curves. Univariate and multivariate Cox regression were used to identify the independent prognostic role of the risk score in each dataset. The “rsm” package was utilized to construct and validate the nomogram model. The patient’s age, WHO grade, and risk score were treated as continuous variables in our nomogram model. using the “calibrate” function of the “rms” package to carry out calibration plot.

### Single-sample gene-set enrichment analysis

using a single-sample gene-set enrichment analysis (ssGSEA) algorithm to quantify the enrichment level of 29 immune related gene sets in each LGG sample [27, 28]. The types, functions, and pathways of immune cells were included in these gene sets. The values obtained by the ssGSEA indicated the absolute enrichment degree of the gene set in each LGG sample.

### Cell cultures

Normal human astrocytes (NHA), U87-MG human GBM cell was kindly provided by Cell Bank/Stem Cell Bank, Chinese Academy of Sciences. and LN229 human GBM cell was obtained from iCell (Shanghai, China). NHA, U87-MG and LN229 were cultured in MEM and DMEM, respectively. For all cell lines, medium was supplement with 10% fetal bovine serum (FBS; Gibco). All cell lines were maintained in a constant temperature incubator of 37 °C in a 5% CO_2_ environment.

### Western blotting, co-immunoprecipitation, and quantitative real-time PCR analysis

GBM cells were harvested and lysed in RIPA buffer, containing phosphatase and protease inhibitors. Protein lysates (25 µg) were loaded and separated on 6–12% SDS-PAGE gels for western botting (WB) and transferred to polyvinylidene difluoride (PVDF) membranes. The membranes were incubated with primary antibodies, including GAPDH (1:2000, Cell signaling Technology, CST), AKT1 (1:1000, Proteintech), p-AKT(S473) (1:5000, Proteintech), PAK1 (1:1000, abcam), p-PAK1(T212) (1:1000, Abclonal). Then these membranes were incubated with HRP-conjugated secondary antibody. The ECL Plus western blot detection reagent (Us Everbright, UE) was used to visualize blots using an imaging system (5200 Multi chemiluminescent, Tanon) and the intensity of the protein bands was quantified by ImageJ software (1.53a, National institutes of Health, USA).

For co-immunoprecipitation (IP) assays, GBM cells lysates were collected and immunoprecipitated with protein A/G beads and the corresponding antibodies at 4℃ overnight. Precooled IP lysis buffer was used to wash the beads five times and the treated beads were boiled in loading buffer and then lysates were used for western blot analysis.

Total RNA was extracted from GBM cells using Simply P Total RNA Extraction Kit (Bioer Technology, China), and then RNA was reverse transcribed to cDNA using High-Capacity cDNA Reverse Transcription Kits (Bio-Rad). Quantitative real-time PCR (qRT-PCR) was conducted using cDNA specific primers and SYBR Green qPCR Mix following the manufacturer’s introductions. Primer sequences were provided by PrimerBank (https://pga.mgh.harvard.edu/primerbank/index.html) (Additional file 27: Table S3). The 2^−ΔΔCt^ method was performed to calculate the relative mRNA expression of PAK1 and AKT1 [29]. PAK1 mRNA expression was analyzed using GraphPad Prism software (version 8.0.1, USA) after normalizing to GAPDH.

### CCK8 assay

CCK-8 Kit (Beyotime, China) was used to measure the proliferation of U87 and LN229 GBM cells. We added 100 µL medium containing 2000 cells to each well of a 96-well plate with three repeated wells in each group. Then, following the treatment period, 10 μL CCK-8 reagent was added to 90 µL cell culture medium per well and 100 μL detection solution was added before incubating for 1.5 h. The detection was performed at the 0, 24, 48, 72, 96, and 120 h time points.

### Cell imaging

The cells were washed three times with PBS and fixed with 4% paraformaldehyde for 30 min. After and additional three washes with PBS, the cells were incubated in 0.2% Triton-X-100 for 10 min. 5% goat serum used for blocked for 1 h, which was followed by three washes with PBS. Next, the cells were treated with anti-PAK1 (Abcam, 1:100) and anti-AKT1 (Proteintech, 1:50) for 4 °C and incubated overnight. Then the cells were incubated with a fluorescein binding secondary antibody (CST, 1:200) at room temperature for 1 h. The cell nucleus was stained for 30 s with 4,6-diamino-2-phenylindole (DAPI, Solarbio). A confocal fluorescence microscope (60 ×) was used to acquire images.

### Statistical analyses

Box plots were applied to reveal the differential gene expression of PAKs between 28 tumors and the corresponding normal samples. Univariate or multivariate Cox proportional hazard regression analysis were performed to investigate the correlation between PAKs gene expression and survival data, including OS, DFS, progression-free interval (PFI), and disease-specific survival (DSS) of tumor patients. In addition, these were also used to determine the independent prognostic value of various clinical characteristics of LGG or GBM patients and for the construction of the risk score based on PAKs expression. To explore the relationship between PAK expression and immunity in human tumors, we comprehensively analyzed the correlation and significance between PAKs and immune-related genes and immune cells in pan-cancer. In addition, the ESTIMATE score, defined as the estimation of stromal and immune cells in malignant tumor tissues using expression data, the immune score, stromal score, and tumor purity were used to analyze the infiltration level of immune cells and stromal cells in different tumors [30]. The analysis was based on gene expression profiles retrieved from TCGA expression data. Spearman’s correlation was used to test the association between PAK gene expression and these scores. In GBM patients, two subgroups were divided by the median expression of PAK1 in TCGA database. Next, we investigated the differences in biological processes between the two subgroups using GSEA analysis, and detected the significantly enriched pathways and tumor hallmarks. ns: no significance; *: p < 0.05; **: p < 0.01; ***:p < 0.001; ****:p < 0.0001.

## Results

### Landscape of PAKs expression across human cancers

**Fig. 1:**
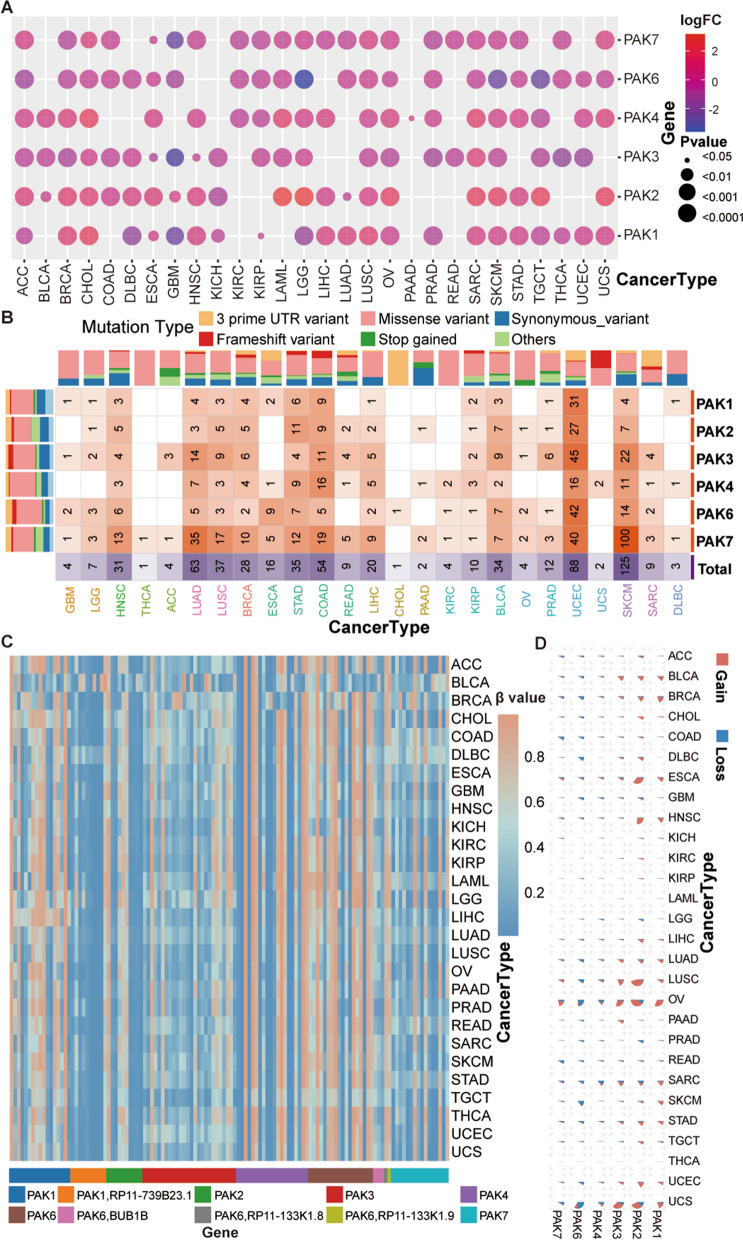
Comprehensive analysis of PAKs in human tumors and corresponding normal samples. **A** Differences in PAKs gene expression between 28 tumors and corresponding normal tissues, solid circles indicate p value < 0.05, no circle indicates p value > 0.05. **B** The mutation frequency and types of PAKs gene in pan-cancer, the numbers inside each cell indicate the total number of various mutation types. **C** The heatmap shows the degree of promoter methylation of PAKs gene in different tumors. **D** Gain and deletion of PAKs gene in pan-cancer

To explore the pattern of dysregulation of PAK expression in 28 human tumors, we extracted PAK gene expression data of 28 human tumors and corresponding normal tissues from TCGA and GTEx databases, and then analyzed the landscape of PAKs through mRNA expression, gene mutation, CNV, and DNA methylation data in pan-cancers. To detect expression level and differences in PAK expression in 28 human tumors and corresponding normal tissues, we analyzed the extracted mRNA expression data of PAKs using the FPKM format. The results are shown in Additional file 3: Figure S1, and Fig. 1A shows the logFC (Fold Change) values and significant differences for each tumor and the corresponding normal tissues.

For all members of the PAK gene family, significant expression differences were detected between the same tumor and normal tissue, while for any member of the PAK gene family, significant heterogeneity was found across different tumors and normal tissues. First, we found PAK1, PAK2, and PAK4 were highly expressed in all tumors. In addition, we found that the expression of PAKs in most tumors was distinctly higher than that in the corresponding normal tissues, while PAK1, PAK2 and PAK4 showed the most significant differences but only in Cholangiocarcinoma (CHOL) and Acute Myeloid Leukemia (LAML). Interestingly, in Adrenocortical carcinoma (ACC), Breast invasive carcinoma (BRCA), Lymphoid neoplasm diffuse large B-cell lymphoma (DLBC), Kidney chromophobe (KICH), GBM, LGG, Prostate adenocarcinoma (PRAD), Skin cutaneous melanoma (SKCM), Testicular germ cell tumors (TGCT), Thyroid carcinoma (THCA) and Uterine corpus endometrial carcinoma (UCEC), the expression of some PAK family members was significantly lower in tumor tissues (logFC < 1 and P < 0.05), a difference which was more significant in GBM and LGG. In general, significant differences in the expression of PAKs could be detected in most tumors. Consistent with many existing studies, PAK1 and PAK4 may be considered oncogenes in many tumors, and the expression level is higher than that in normal tissues.

To comprehensively analyze the landscape of PAKs in 28 tumor types, we further studied the genetic variation of PAKs (Fig. 1B–D). We found that the missense variant was the most common mutation in most tumors and for all PAKs, while no mutations were detected in KICH, LAML, and TGCT. PAK7 appeared to mutate most frequently while PAK4 showed the least variation (Additional file 28: Table S4). Overall, the mutation frequency of PAKs in tumors was relatively low, which was consistent with previous reports. We analyzed the methylation sites of six PAK family genes, and found that there were relatively stable methylation sites in all tumors. In addition, multiple methylation sites of PAK2, PAK3, PAK4, and PAK7 promoters were found to be fully methylated in various tumors. To study the relationship between the methylation level of PAKs and their mRNA expression in more depth, we obtained the results from the GSCA, which showed that the expression of PAKs was negatively correlated with the methylation level in most tumors (Additional file 4: Figure S2A).

In the evaluation of the CNV of PAKs in human tumors, we found that the frequency of CNV in most tumors is very low, and only a few tumors have higher frequency of CNV. To analyze whether the high frequency of CNV in PAKs family gene was related to the CNV of PAKs, we obtained compared relationship between CNV data from GSCA, and found that the occurrence of CNV only had a significant impact on the prognosis of patients in a small number of tumors such as UCEC, Kidney renal papillary cell carcinoma (KIRP), LGG, and Kidney renal clear cell carcinoma (KIRC), while for most tumors, the impact of CNV on patients was limited (Additional file 4: Figure S2B-E). Therefore, the PAKs detected in different cancers showed different regulatory patterns, which indicated that the regulation the expression patterns of PAK gene family members expression patterns are tumor specific.

### Association of patient survival with PAK gene expression

**Fig. 2:**
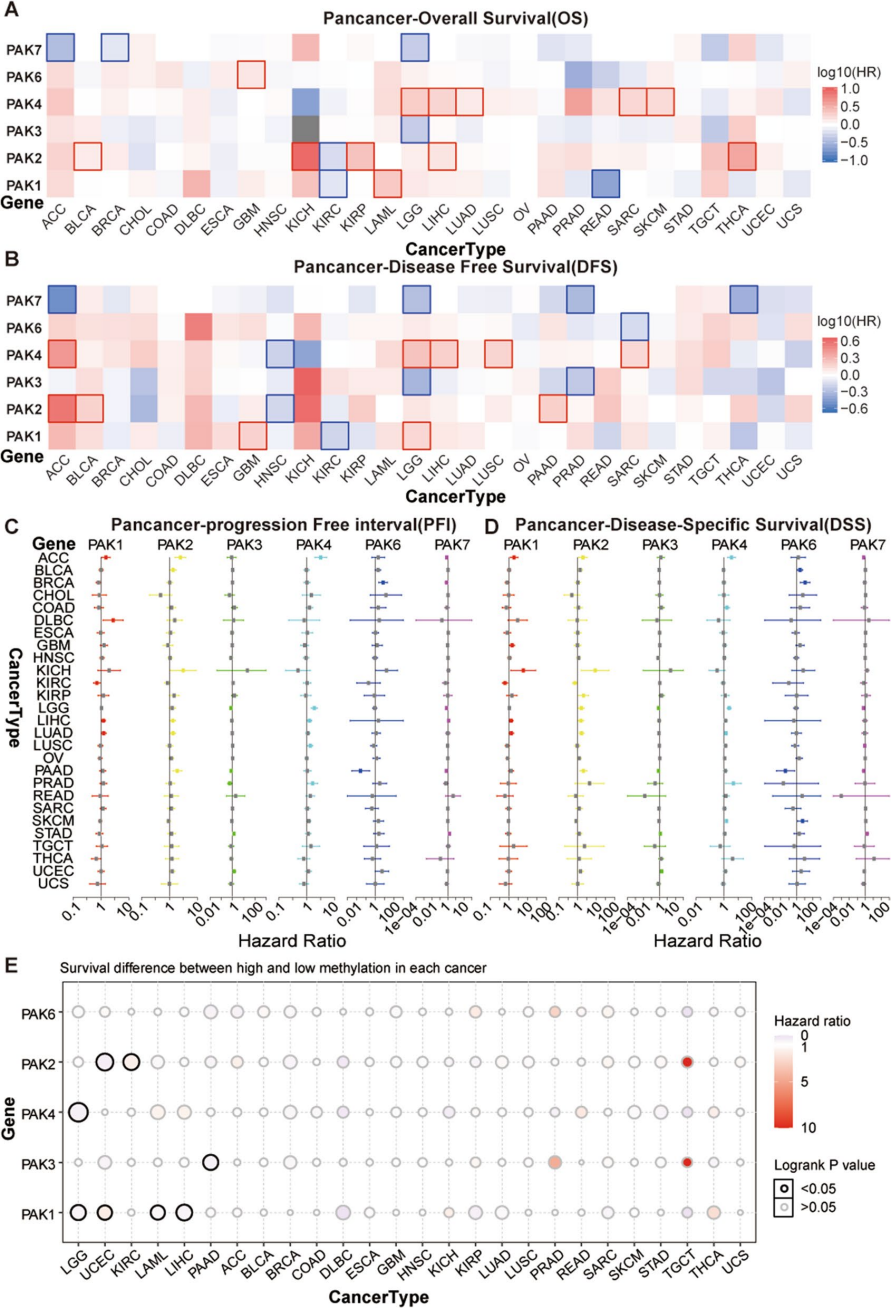
The heatmap shows the association of PAKs expression with the Overall Survival (**A**) and Disease-Free Survival (**B**) of patients with different cancer types. Cells with borders represent p < 0.05. **C**, **D** The forest plot of Progression Free Interval and Disease-Specific Survival in pan-cancer patients shows the survival advantages and disadvantages of increased PAK gene expression. Univariate Cox proportional hazards regression model for association test. **E** Survival differences between high and low methylation of different tumor types. The size of the point represents the p value

**Fig. 3:**
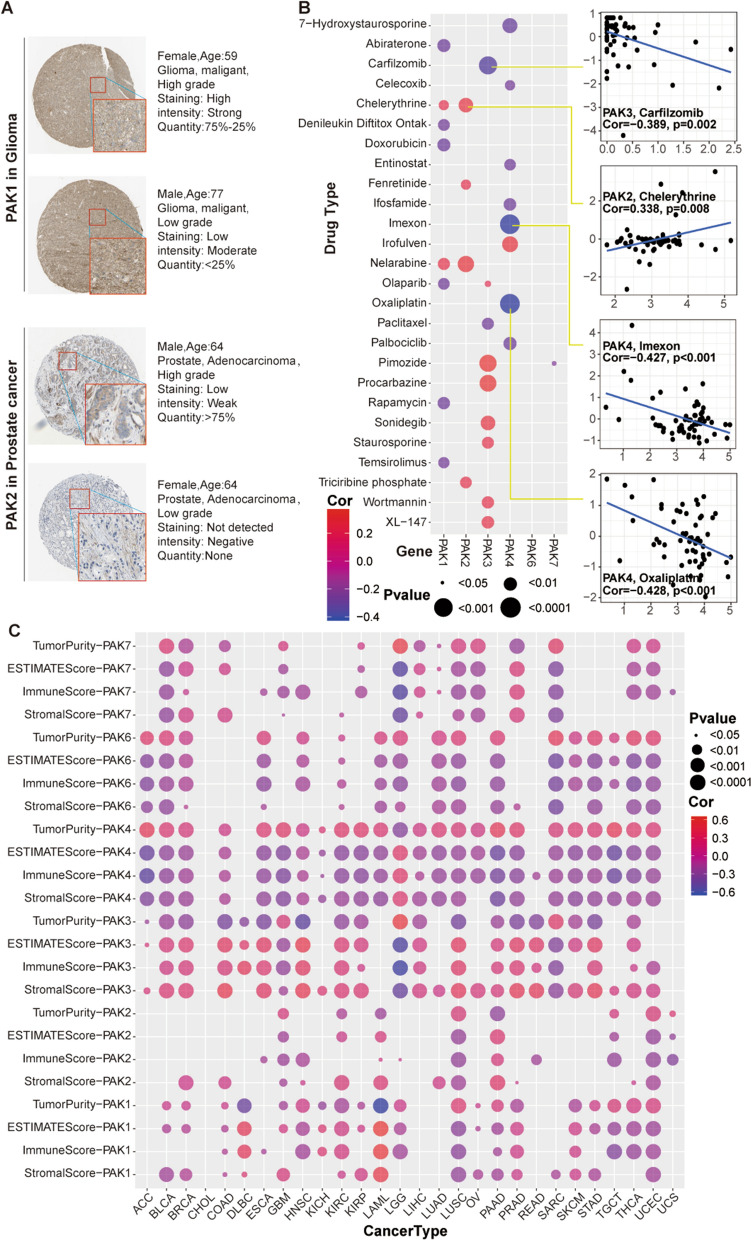
**A** Immunohistochemical staining of PAK1 and PAK2 in Glioma and prostate cancer tissue samples from The Human Protein Atlas. **B** Heatmap showing the association between PAKs gene expression and drug sensitivity (Z-score from the CellMiner interface) using NCI-60 cell line data. The horizontal axis represents the expression of PAKs, and the vertical axis represents the drug sensitivity. **C** Correlation between PAKs mRNA expression and Tumor Purity, ESTIMATE Score, Immune Score, and Stormal Score, respectively

To determine the effects of PAKs on the prognosis of 28 kinds of human tumor patients, we combined the mRNA expression data of PAKs and survival data, including OS, DFS, PFS, and DSS of the patients, and then divided them into high- and low- expression groups according to the median expression of 6 PAK gene family members, respectively. We used univariate Cox proportional hazard regression model for analysis, with P < 0.05 as the significance cutoff value (Fig. 2A–D, Additional file 5: Figure S3, Additional file 6: Figure S4, Additional file 7: Figure S5, Additional file 29: Table S5). We found that PAK7 was mainly associated with a survival advantage, while PAK1, PAK2, PAK4, and PAK6 were mainly associated with survival disadvantages. Nevertheless, the specific survival advantages and disadvantages of PAKs depended on the cancer type. PAK7 showed a survival advantage in survival data of ACC and LGG patients, indicating that patients with high expression of PAK7 in ACC and LGG had a better prognosis. PAK3 also showed a stable survival advantage in LGG, which implied that LGG patients with high expression of PAK3 exhibited better survival. Although PAKs were mainly associated with survival disadvantages in most cancer patients, the survival analysis results of some PAK gene family members combined with multiple survival data in many cancer patients were not conclusive. For example, the association between OS and PAK4 gene expression of ACC patients was not significantly correlated, while for DFS, PFI, and DSS, ACC patients with high expression of PAK4 might achieve a better prognosis, which suggests that the effects of radiotherapy and chemotherapy in some tumors may partly depend on the expression fluctuations of PAKs family members. In addition, due to the lack of PAK7 data in the GSVA database, we grouped 28 tumor patients according to the methylation status of PAK1, PAK2, PAK3, PAK4, and PAK6, respectively, and then analyzed the survival difference of tumor patients (Fig. 2E, Additional file 31: Table S7). The results showed that the methylation of PAK1 had a critical impact on the prognosis of LGG, UCEC, LAML, and LIHC patients, and in LGG, LAML and LIHC, the methylation of PAK1 was associated with a survival advantage, suggesting that patients with high PAK1 methylation may have better prognosis. However, in general, the methylation of PAKs had no significant impact on the prognosis of most human tumor patients. Nonetheless, the role of methylation of individual PAK gene family members in some tumors warrants further study. Based on HPA, we compared the results our RNA level analysis above regarding PAKs in pan-cancer. Surprisingly, we found that high-grade gliomas contained higher levels of PAK1 protein, and samples with lower PAK1 levels were from low-grade gliomas. The difference of PAK2 protein in glioma samples is similar to that of PAK1. (Fig. 3A).

### Cancer cell sensitivity to chemotherapy and PAKs associated with tumor immunization

PAK gene family members may play a key role in tumor resistance. To ascertain whether the expression of PAKs in NCI-60 cell lines was associated with the occurrence of drug resistance, we systematically tested the correlation between the expression of PAKs in 60 human cancer cell lines (NCI-60) and the sensitivity towards more than 200 chemotherapeutic drugs (Fig. 3B, Additional file 8: Figure S6 and Additional file 9: Figure S7). Drug sensitivity was measured using the Z-score. The higher the score, the more sensitive the tumor cells were predicted to be to drug therapy. In general, the increased expression of PAK1 and PAK4 was mainly associated with the decreased drug sensitivity of different cell lines, while the increased expression of PAK2 and PAK3 was mainly related to the increased drug sensitivity of different cell lines. Interestingly, the correlation between the higher expression of PAK6 and PAK7 and the sensitivity of different cell lines to multiple drugs was very limited, which indicated that there was no significant correlation between expression of PAK6 and PAK7 and treatment with chemotherapeutic drugs. In addition, we obtained GDSC and CTRP drug sensitivity data and their correlation with PAKs expression from the GSCA (Additional file 10: Figure S8). The results showed that PAK1 expression was generally negatively correlated with drug sensitivity, while PAK4 expression was mostly positively correlated with drug sensitivity.

We divided patients of each tumor into high- and low-PAKs expression groups based on the median expression of each PAK gene and compared the above four indicators across expression groups to explore whether differences in expression of PAKs were associated with the matrix score, immune score, evaluation score, or tumor purity. As shown in Fig. 3C, most human tumors, except LGG, had significantly higher tumor purity in the PAK4 high expression group than in the PAK4 low expression group (p < 0.001), while the stromal, immune, and ESTIMATE scores show the opposite trend. These results indicated that in most tumors, high expression of PAK4 was associated with a lower number of immune cells and stromal cells, but the number of tumor cells was higher than present in low-PAK4 expressing samples, which was particularly relevant for LGG samples. With regarded to PAK3, in three types of tumors, including LGG, GBM, and SARC, samples, the number of infiltrating tumor cells was higher than that of low expression samples, which was contrary to most other tumors. For the other PAKs, the strong tumor specificity was reflected by the stromal, immune, and ESTIMATE scores and by tumor purity of tumor samples, and did not show a constant trend or significant difference in pan-cancer.

### Comprehensive analysis of immune correlation of PAKs

**Fig. 4:**
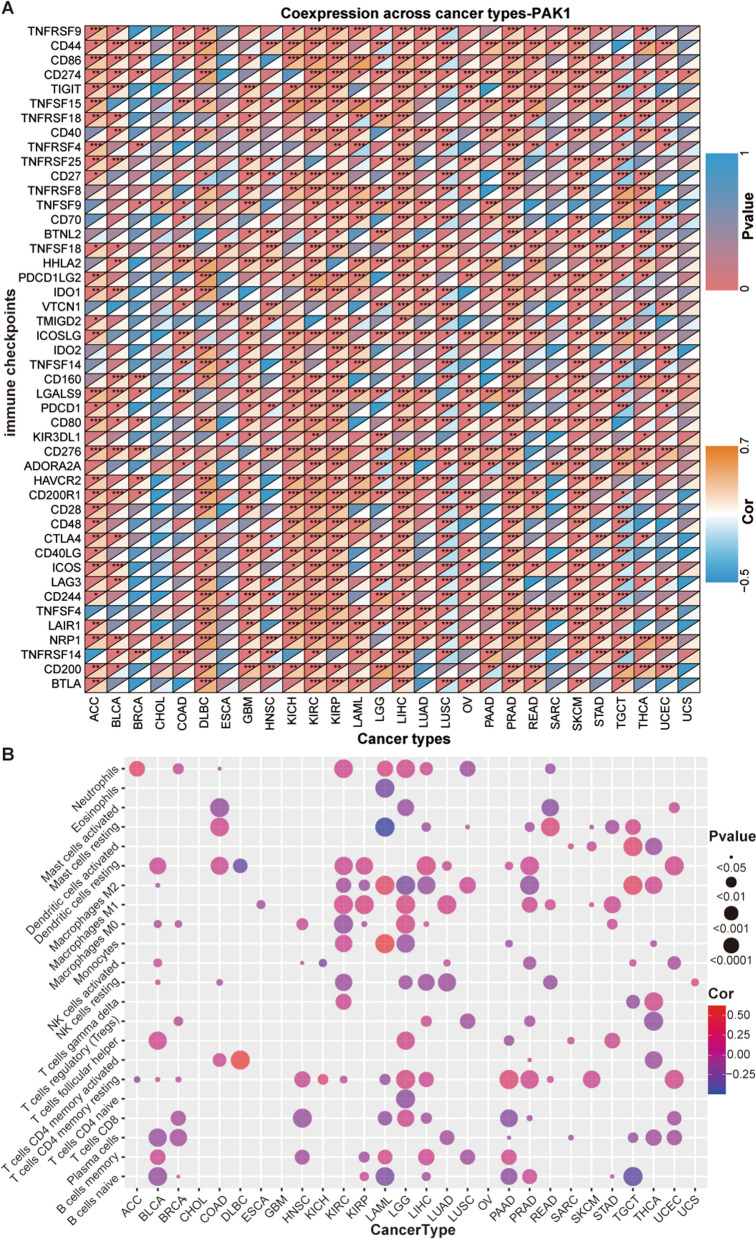
**A** Correlation between PAK1 gene expression level and 46 immune checkpoints in pan-cancers. **B** Correlation between PAK1 gene expression level and different immune-related cell

Studies have shown that the immune checkpoint (ICP) genes have an important effect on immune cell infiltration and immunotherapy [31, 32]. We tested the correlation between PAKs expression and ICP genes in human cancers to study the potential role of PAKs in immunotherapy. Among the 46 ICP genes studied, the expression of PAKs, especially PAK1, was surprisingly significantly correlated, and most were positively correlated, with ICP genes in numerous cancers (Fig. 4A, Additional file 11: Figure S9 and Additional file 12: Figure S10). Herein, we focused our analysis on the relationship between PAK1 expression and ICP genes in pan-cancer. As shown in Fig. 4A, the high expression of PAK1 may be associated with the poor therapeutic effect of immunotherapy targeting ICPs. In GBM, LUSC, TGCT, BRCA, COAD, and THCA, PAK1 was positively correlated with the ICP gene expression, which suggested that PAK1 may regulated tumor immune response by immune checkpoint regulation. Studies have shown that tumor mutational burden (TMB) and microsatellite instability (MSI) in the tumor microenvironment (TME) are related to anti-tumor immunity and can be used to predict the efficacy of tumor immunotherapy [33–35].

To explore the role of PAKs in immune mechanisms and the immune response of the TME, we analyzed the correlation between the expression of PAK gene family members and TMB and MSI (Additional file 13: Figure S11, Additional file 14: Figure S12). We determined that the correlation between PAKs and TMB and MSI showed strong tumor specificity, and the PAK gene may exert different effects on the TMB and MSI of different tumors. Accordingly, the expression of PAK1, PAK3, PAK4, and PAK5 was significantly positively correlated with TMB in THYM, while the expression of PAK2 was negatively correlated with the TMB. The expression of PAK1, PAK2, PAK3, PAK4, and PAK6 was significantly negatively correlated with the MSI of DLBC, while the expression of PAK7 was positively correlated with MSI. Next, we evaluated the relationship between PAKs expression and several common immune cells and genetic markers of infiltrating immune cells (Fig. 4B, Additional file 15: Figure S13, Additional file 16: Figure S14, Additional file 17: Figure S15, Additional file 18: Figure S16, Additional file 19: Figure S17). Through our analysis, we determined that the correlation between PAK expression and immune cells infiltration showed significant tumor specificity, and different PAK genes in the same tumor may show completely different expression patterns. In addition, in our analysis of the correlation between the expression of genetic markers of infiltrating immune cells and PAKs, we found that the expression of PAKs in LGG patients was significantly correlated with most of the 46 ICP genes, although some were positively and others were negatively correlated.

Similar to the many studies described above, the correlation between PAKs expression and genetic markers of infiltrating immune cells is also tumor-specific, but their correlation in CHOL and UCS was relatively poor with no significant difference. Thus, the correlation between immune cells and PAKs expression was determined to be very similar and indicates that the relationship between PAK expression and the immune response in these two tumors is weak. Altogether, our comprehensive assessment of the above findings allows to speculate that PAKs may affect anti-tumor immunity in some cancers by regulating the composition and immune mechanism of the TME.

### Construction and validation of prognostic models based on PAKs in LGG and GBM

**Fig. 5:**
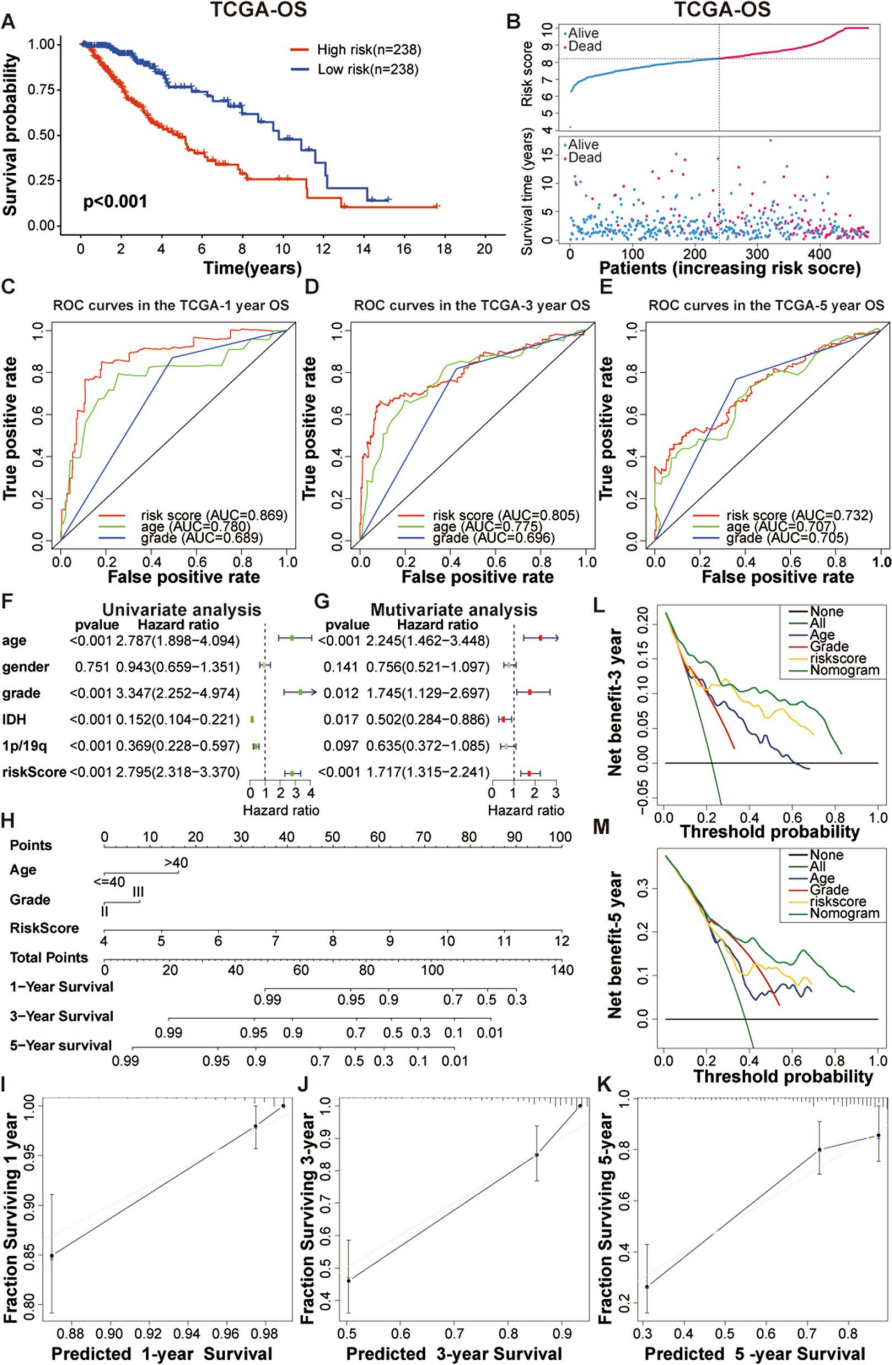
Construction and verification of the prognostic model of PAKs in low-grader glioma (LGG). **A** Kaplan–Meier (K–M) curves of OS of high- and low- subgroups divided by the risk score of LGG patients in the TCGA dataset. **B** The distribution of LGG patients’ survival status, risk scores in the TCGA dataset. **C**–**E** Receiver operating characteristic (ROC) curves for the PAKs signature for the prediction of 1/3/5-year survival of LGG patients in the TGGA dataset. **F**, **G** Univariate and multivariate Cox analyses of different variables, including WHO grade, age, gender, 1p/19q co-deletion status, IDH mutational status, and risk scores. **H** A nomogram was constructed by combining age, WHO grade and risk score to predict the overall survival (OS) of low-grader glioma (LGG). The total score projected on the bottom scale implies the probability of 1-, 3-, and 5-years overall survival. **I**–**K** Nomogram calibration curves were used to predict 1-, 3-, and 5-years survival rates. **L** Decision curve analysis (DCA) curve analysis shows that nomogram has the highest Net Benefit

Our previous studies revealed that the multi-faceted analysis of LGG and GBM showed that these patients exhibit unique performances, and the current prognostic predictive measures of PAKs in LGG and GBM need to be supplemented. Therefore, to analyze the impact of PAKs expression on the survival and prognosis of LGG and GBM patients, we constructed and verified prognostic models of LGG and GBM based on the expression of PAKs. First, using TCGA datasets, the LASSO regression model was used to identify PAKs with stable characteristics to predict OS in LGG patients. The coefficients obtained by regression analysis are shown in Additional file 25: Table S1. Based on this model, we determined the risk scores for all LGG patients in TCGA dataset, and stratified patients into high- and low-risk subgroups based on the median risk values. Survival analysis was used to detect differences in survival between high- and low-risk groups of LGG patients. The results showed that high-risk patients often exhibited a poor prognosis (Fig. 5A) and the patient's survival and risk scores are shown in Fig. 5B.

To determine the predictive power of PAKs signature, we used the risk scores of LGG patients in TCGA dataset to construct a time-dependent ROC curve (Fig. 5C–E). The PAKs signature showed an extremely reliable ability to predict OS in LGG patients. To further determine whether the risk score can be used as a stable and reliable independent prognostic factor for LGG patients, we performed univariate and multivariate regression analysis on age, grade, sex, 1p/19q co-deletion status, IDH mutation status, and risk score to identify hazard ratios (HR), 95% confidence intervals (Cis), and P values (Fig. 5F, 5G), the results showed that patient’s age, risk score, and tumor grade were all positively correlated with the overall risk score, which means these three properties could be used as independent prognostic factors for LGG patients, and can be considered risk factors.

A nomogram was constructed to evaluate the predictive ability of the PAKs signature (Fig. 5H). In TCGA dataset, patient age, tumor grade, and risk score were chosen as independent prognostic factors to construct the nomogram model. The risk score revealed to be the most effective parameter for survival prediction compared with the other two clinical characteristics. In addition, the calibration curves generated for this nomogram to predict the 1-/3-/5-year survival rates also showed remarkable predictive accuracy (Fig. 5I–K). The above analysis was validated in the CGGA seq1, CGGA seq2, GSE16011, and Rembrandt datasets (Additional file 20: Figure S18). In addition, to evaluate the clinical gains of the different strategies, we conducted a decision curve analysis (DCA) (Fig. 5L, 5M). The results showed that the nomogram model was a stronger contributor than other independent predictors. For GBM, due to the extremely high degree of malignancy and poor prognosis, we performed modeling analysis using only the TCGA dataset. Following LASSO regression analysis, only PAK1 was shown to be an adequate stable prognostic factor. A similar analysis of the impact of PAKs signature on the prognosis of LGG patients was performed using PAK1 gene expression in the risk score for GBM patients in TCGA dataset (Additional file 21: Figure S19). PAK1 expression can also be used as a stable independent prognostic factor in GBM. Interestingly, there were no significant differences in the methylation pattern of PAKs between LGG patients in the high- and low-risk groups, and the same trend was shown for GBM patients (Additional file 22: Figure S20).

### Single-sample and gene set enrichment analysis in gliomas

**Fig. 6:**
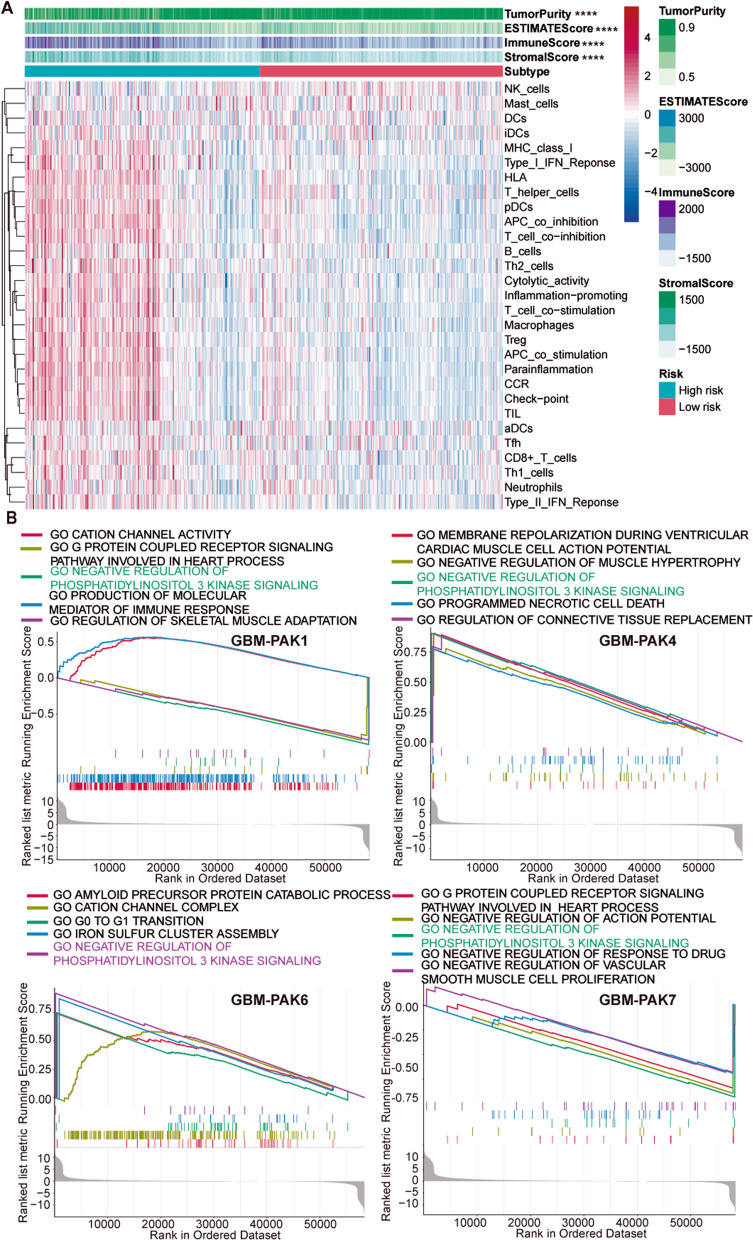
**A** Patients with low-grader glioma (LGG) were divided into high- and low-risk groups based on PAKs signature. Tumor purity, estimated scores, immune scores, and stromal scores were assessed by ESTIMATE based on 29 immune related gene sets. **B** Gene set enrichment analysis (GSEA) shows that significant difference in the enrichment of PI3K signaling pathway between the PAKs high- and low- expression groups in Glioblastoma patients

To evaluate whether there is a significant difference in tumor purity between the high- and low-risk groups of LGG patients based on the PAKs signature, we performed ssGSEA analysis. As shown in Fig. 6A, the high-risk group presented lower tumor purity than the low-risk group with higher immune, ESTIMATE, and stromal scores (P < 0.0001). Furthermore, there were significant differences in most immune-related gene sets between the high- and low-risk groups (P < 0.001). Because the prognosis of LGG patients tends to be superior to than that of GBM patients, and LGG may progress to GBM [36], and we studied the unique prognostic ability of PAK1 in GBM, we then stratified GBM patients according to the levels of PAKs expression. GSEA analysis was performed to detect which pathways presented gene enrichment among patients with high- or low-expression of PAKs (Fig. 6B). We found that in the patient groups with differences in PAK1, PAK4, PAK6, and PAK7 expression, the negative regulatory PI3K pathway was significantly enriched (P < 0.05), which indicated that the evaluated PAKs and the PI3K pathway may jointly participate in the regulation of the occurrence and development of GBM (Additional file 31: Table S7).

### Function role of PAK1 in GBM

**Fig. 7:**
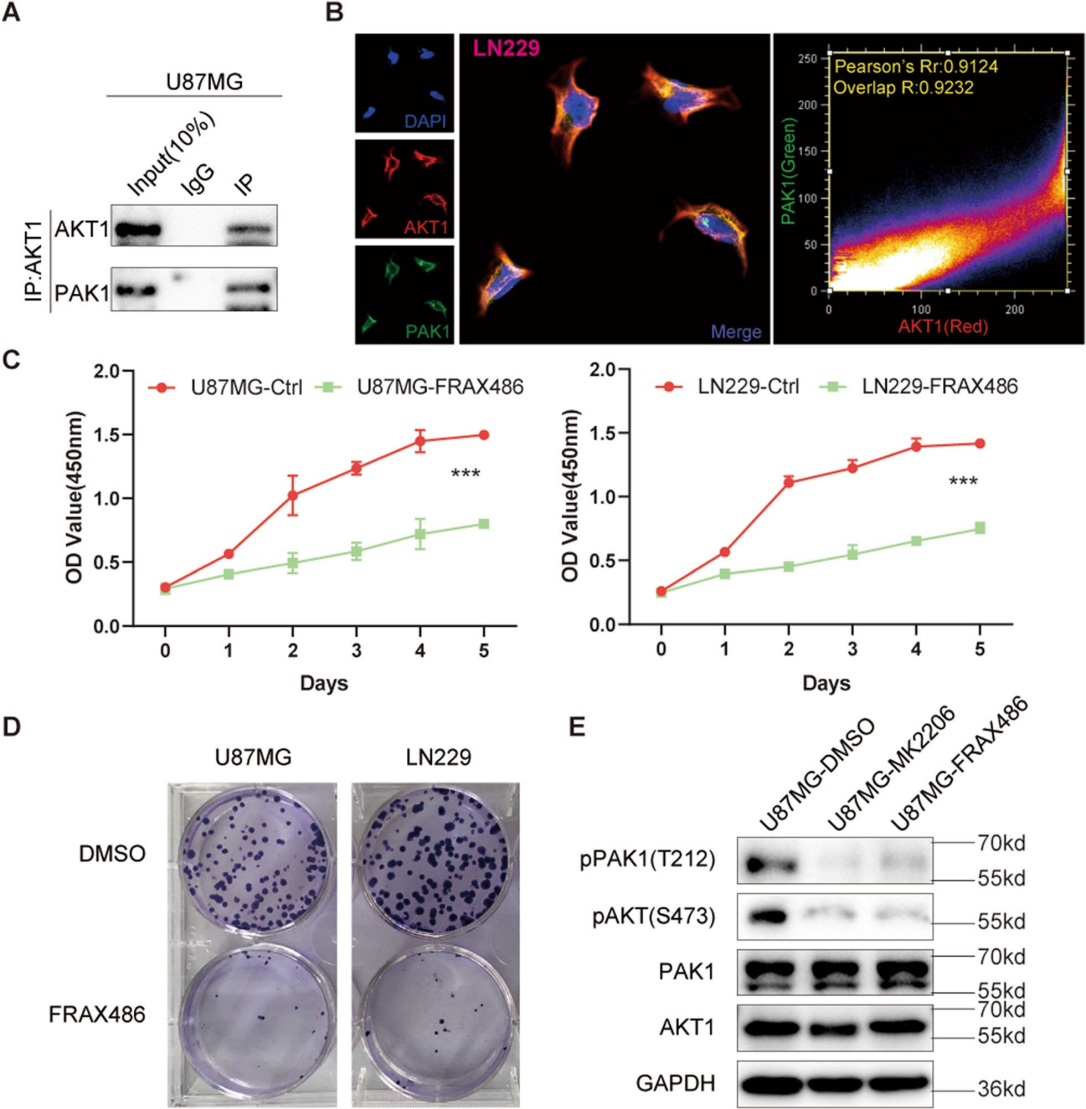
Experiments to verify the effect of PAK1 on the occurrence and development of GBM cells. **A** Immunoprecipitation analysis was performed to detect the mutual binding between AKT1 and PAK1 in U87 cells. **B** Cellular immunofluorescence shows the co-localization of AKT1 and PAK1 in LN229 cells. CCK8 assay (**C**) and Clonogenic assay (**D**) showed that the PAK1 inhibitor FRAX486 has a significant inhibitory effect on the proliferation of glioma cells. **E** Western blot analysis was performed for detecting the protein lysates of PAK1, p-PAK1(T212), AKT1, p-AKT(S473) and GAPDH of U87 cells after being treated with 12 μM MK2206 and 1.2 μM FRAX486 for 24 h

There are many associations between PAK kinase and the PI3K signaling pathway in different tumors [37, 38]. PAK is clearly identified as the effector of Rac and Cdc42, and PAK1 is also believed to play a part in the activation of AKT by Rac [21, 39, 40]. As a key member of the PI3K pathway and a core functional protein, AKT1 plays an important role in the occurrence and development of a variety of tumors [41, 42]. To explore whether there is an interaction between PAK1 and AKT1 in GBM cells, we performed immunoprecipitation analysis on U87MG and LN229 GBM cell lines. The results showed that AKT1 and PAK1 exhibit significant mutual binding in GBM cells (Fig. 7A, Additional file 23: Figure S21A), and cellular immunofluorescence assays also showed the evidence of cellular co-localization in GBM cells (Fig. 7B, Additional file 23: Figure S21B). After treatment with FRAX486, a PAK1 inhibitor, cell proliferation was significantly inhibited (Fig. 7C, D). To further explore the effects of abnormal PAK1 activation on GBM cells further, we treated the GBM cell lines with FRAX486, MK2206 (AKT1 inhibitor), and DMSO as the control condition. Through WB experiments, we found that PAK1 and AKT1 inhibitors both significantly reduced phosphorylated (p)-PAK1 and p-AKT1 levels in GBM cells (Fig. 7E, Additional file 23: Figure S21E), while the protein and mRNA levels of PAK1 and AKT1 were not significantly altered (Fig. 7E, Additional file 23: Figure S21C–E). We also examined the mRNA levels of AKT1 and PAK1 in NHA, U87MG and LN229 cells, AKT1 showed an upward trend, while PAK1 showed a significant decrease (Additional file 24: Figure S22A). In order to further examine the relationship between PAK1 and AKT and pAKT, based on the CCLE database, we obtained the mRNA expression of PAK1 and AKT1 in tumor cell lines, the protein levels of AKT and pAKT in tumor cell lines, and analyzed the PAKs mRNAs and their correlations. The results showed that there was no significant correlation between PAK1 mRNA level and AKT and pAKT protein levels (Additional file 24: Figure S22B, C; Additional file 32: Table S8, Additional file 33: Table S9, Additional file 34: Table S10). Accordingly, PAK1 may promote the occurrence and development of GBM via the abnormal activation of PAK1, which suggests that the development of specific PAK1 inhibitors to reduce p-PAK1 for the treatment of some cancers may be very promising.

## Discussion

PAK is a serine/threonine-specific intracellular protein kinase, located at the intersection of multiple important signaling pathways required for tumorigenesis and development [43]. PAKs have been shown to play an important role in the growth, survival, and progression of human cancer [44]. In the context of the proliferative signal transduction of many tissues, PAK activity can often regulate the effective activation of ERK, AKT, and β-catenin pathways [45, 46]. Due to these effects, cells may be particularly sensitive to targeting by specific PAK small molecule inhibitors. Although drug-targeted therapy of oncogenic kinases is a promising approach, and many oncogenic kinases have successfully been developed as drug-targeted therapies, drug resistance is a very common and foreseeable problem; thus, additional therapeutic targets and targeted drugs need to be explored. When PAK subtypes are overexpressed, they are mutated or abnormally activated by upstream elements including small GTPases Rac or the cell division control protein 42 (Cdc42). After being activated, most PAK subtypes exert carcinogenic effects on cells, including the promotion of growth signal autonomy, invasion, and metastasis, and avoidance of apoptosis [44]. Thus, this study focused on the comprehensive analysis of PAK in pan-cancer, exploring specific PAK family members that can potentially serve as a key carcinogenic signal in the occurrence and development of human tumors, and further, influence the prognosis of patients as well as the occurrence and development of tumors. Importantly, we focused on the activation mechanism of these PAK genes in specific cancers, the key substrates that mediate the development and carcinogenesis of these PAK kinases, and the potential value of PAK as a drug target for the treatment of cancer.

We analyzed the landscape of PAK gene involvement in pan-cancer based on their mRNA, CNV, mutation, and methylation status. Studies have shown that PAKs have significantly different mRNA expression levels in tumors, and there are also significant differences in expression levels between many tumor tissues and normal tissues. These differences may reflect as up- or down-regulated expression of PAK genes. Among the different tumors, GBM showed very different PAK expression levels. For instance, PAK1, PAK3, and PAK7 were significantly down-regulated in GBM compared to the corresponding normal tissues, while PAK2 and PAK6 were significantly up-regulated. Studies on PAKs in gliomas showed that PAK1 and PAK4 play an important role in the occurrence and development of GBM, and the abnormal activation of PAK1 is even more prominent [1, 43, 47]. In addition, PAKs show generally low methylation, CNVs, and mutations. In only a few tumor patients can prognosis or tumor development may be related to these changes. Although there are few studies on the methylation and mutation status of PAKs, it is still unclear whether these changes affect their kinase activity or the occurrence and progression of tumors [5]. Nevertheless, some studies have shown that the changes exhibited by some PAKs may play an important role in tumorigenesis and progression. [5, 48–50].

In the survival analysis of pan-cancer patients, high expression of PAK1, PAK2, PAK4, and PAK6 as well as the low expression of PAK7 was mainly associated with poor prognosis of tumor patients. Specifically, the role of PAKs in specific pan-cancers was significantly tumor-specific, which implied that the role of PAKs-specific targeted drugs in tumors may also be different, indicating more research on PAKs is warranted.

Specific inhibitors or targeted drugs are used to treat various tumors. Interestingly, the HPA database shows that patients with high expression of PAK1 have a worse prognosis, which is opposite to the trend in differential mRNA expression of PAK1 between LGG, GBM and the corresponding normal samples. Thus, experimental validation of the role and mechanisms involving PAK1 in promoting occurrence and development of GBM is needed. Drug sensitivity studies support the view that drugs targeting PAK1, PAK2, PAK3 and PAK4 can be used as therapeutic agents [3, 43, 51]. Our comprehensive immune analysis found that except for PAK1, PAK3, PAK4, PAK6, and PAK7, the immune purity and ESTIMATE scores of many tumors can be significantly distinguished by stratifying patients into high- and low-expression groups and this stratification reveals the differential expression of immune cells, immune genes, and immune infiltration. This provides us with a rationale for combining PAKs with immunotherapy and the selection of immune targets. Different levels of PAK gene expression can significantly distinguish immune cells and genes of different tumors, showing strong tumor specificity. The underlying mechanisms for these results have yet to be studied. Further research is also required to identify additional therapeutic targets and potential small molecule inhibitors.

In view of the specific association between PAKs, especially PAK1, in GBM and LGG, we constructed a stable prognostic model based on the expression of PAKs in LGG and GBM patients using TCGA dataset and verified its validity. Our findings indicated that PAKs could be used as a prognostic factor in LGG and GBM patients. The nomogram model was also shown to be a stable and reliable prognostic factor, and further confirmed the predictive stability of PAKs.

Many studies have shown that PAKs can influence the occurrence and development of tumors through multiple signaling pathways [3]. Through GSEA analysis, we have identified differences in the enrichment degree of PI3K pathway genes among GBM patients with high- and low-expression of PAK1, PAK4, PAK6, and PAK7. AKT is a key molecule of the PI3K pathway, and thus we focused on the impact of the interaction between PAK1 and AKT1 on GBM cells, and the effect of PAK1 inhibitors on the occurrence and development of GBM [52, 53]. The results show that the increase of p-PAK1 levels was extremely important for the maintenance of the malignant behavior of GBM cells, which also explained why the expression of PAK1 in glioma and paracancerous tissues showed differentially higher expression of PAK1 in glial cells. At the same time, we also found that there was no significant correlation between the expression of PAK family and the level of pAKT, and our study showed that there was a significant relationship between the level of pPAK1 and the content of pAKT, which may suggest that PAK1 regulates AKT1 mainly through pPAK interacts with AKT, but pAKT will not be induced to degrade after activation or inhibition, which also indicates that PAK mainly acts as a kinase to phosphorylate AKT protein. In addition, the mRNA expression of PAK family genes may not be linearly correlated with AKT expression, indicating that they have no significant regulatory relationship at the transcriptional level.. Because the abnormal activation of PAK1 in tumor tissues may promote tumorigenesis and development, this provides a direction for the development of PAK inhibitors. Similarly, the development of specific PAKs inhibitors may also be beneficial for targeting other tumors.

Although this study conducted a comprehensive analysis of PAKs expression in pan-cancer from multiple perspectives, some obvious limitations still exist. First, the study was mainly based on data analysis and the discussion stemmed mainly from bioinformatics analysis, coupled with insufficiently extensive data sources, which may intrinsically result in further limitations. In addition, despite using the many samples included in TCGA datasets for analysis, our findings lack sufficient data validation and verification in tumor cell lines from other large datasets. Furthermore, most of the data used in the study were collected retrospectively, which does not allow absolute and definitive conclusions. In addition, the data collected by different data sources may derive from different processing methods without taking into consideration tumor heterogeneity, which may reduce the reliability of our findings. Nonetheless, the conclusions drawn from this comprehensive analysis are more comprehensive and reliable, and provide a reliable basis for future experimental verification.

## Conclusions

Although the current study has produced meaningful discoveries, many conclusions and phenomena need to be verified in more complete in vivo and in vitro models. Further, we propose the active development and testing of new specific anti-tumor drugs targeting PAKs.

**Table 1:** The abbreviations, number and sourse of samples for the 28 tumor typesin our study

Cancer type	Abbreviation	Number of samples	Type	Source of samples
Adrenocortical carcinoma	ACC	127	Tumor	The Cancer Genome Atlas
		79	normal	The Genotype-Tissue Expression
Bladder urothelial carcinoma	BLCA	19	Tumor	The Cancer Genome Atlas
		411	normal	The Cancer Genome Atlas
Breast invasive carcinoma	BRCA	292	Tumor	The Cancer Genome Atlas
		1104	normal	The Genotype-Tissue Expression
Cholangiocarcinoma	CHOL	9	Tumor	The Cancer Genome Atlas
		36	normal	The Cancer Genome Atlas
Colon adenocarcinoma	COAD	41	Tumor	The Cancer Genome Atlas
		471	normal	The Cancer Genome Atlas
Lymphoid neoplasm diffuse large B-cell lymphoma	DLBC	107	Tumor	The Cancer Genome Atlas
		48	normal	The Genotype-Tissue Expression
Esophageal carcinoma	ESCA	11	Tumor	The Cancer Genome Atlas
		162	normal	The Cancer Genome Atlas
Glioblastoma multiforme	GBM	5	Tumor	The Cancer Genome Atlas
		168	normal	The Cancer Genome Atlas
Head and Neck squamous cell carcinoma	HNSC	44	Tumor	The Cancer Genome Atlas
		502	normal	The Cancer Genome Atlas
Kidney chromophobe	KICH	24	Tumor	The Cancer Genome Atlas
		65	normal	The Cancer Genome Atlas
Kidney renal clear cell carcinoma	KIRC	72	Tumor	The Cancer Genome Atlas
		535	normal	The Cancer Genome Atlas
Kidney renal papillary cell carcinoma	KIRP	32	Tumor	The Cancer Genome Atlas
		289	normal	The Cancer Genome Atlas
Acute Myeloid Leukemia	LAML	337	Tumor	The Cancer Genome Atlas
		151	normal	The Genotype-Tissue Expression
Brain lower grade glioma	LGG	206	Tumor	The Cancer Genome Atlas
		529	normal	The Genotype-Tissue Expression
Liver hepatocellular carcinoma	LIHC	50	Tumor	The Cancer Genome Atlas
		374	normal	The Cancer Genome Atlas
Lung adenocarcinoma	LUAD	59	Tumor	The Cancer Genome Atlas
		526	normal	The Cancer Genome Atlas
Lung squamous cell carcinoma	LUSC	49	Tumor	The Cancer Genome Atlas
		501	normal	The Cancer Genome Atlas
Ovarian serous cystadenocarcinoma	OV	88	Tumor	The Cancer Genome Atlas
		379	normal	The Genotype-Tissue Expression
Pancreatic adenocarcinoma	PAAD	4	Tumor	The Cancer Genome Atlas
		178	normal	The Cancer Genome Atlas
Prostate adenocarcinoma	PRAD	52	Tumor	The Cancer Genome Atlas
		499	normal	The Cancer Genome Atlas
Rectum adenocarcinoma	READ	10	Tumor	The Cancer Genome Atlas
		167	normal	The Cancer Genome Atlas
Sarcoma	SARC	396	Tumor	The Cancer Genome Atlas
		263	normal	The Genotype-Tissue Expression
Skin cutaneous melanoma	SKCM	812	Tumor	The Cancer Genome Atlas
		471	normal	The Genotype-Tissue Expression
Stomach adenocarcinoma	STAD	32	Tumor	The Cancer Genome Atlas
		375	normal	The Cancer Genome Atlas
Testicular germ cell tumors	TGCT	165	Tumor	The Cancer Genome Atlas
		156	normal	The Genotype-Tissue Expression
Thyroid carcinoma	THCA	58	Tumor	The Cancer Genome Atlas
		510	normal	The Cancer Genome Atlas
Uterine corpus endometrial carcinoma	UCEC	35	Tumor	The Cancer Genome Atlas
		548	normal	The Cancer Genome Atlas
Uterine carcinosarcoma	UCS	78	Tumor	The Cancer Genome Atlas
		56	normal	The Genotype-Tissue Expression

## Supplementary Information

**Additional file 1**: PAK1, AKT1 protein levels in NHA and GBM cells.

**Additional file 2**: The raw experimental data related to this study.

**Additional file 3: Figure S1**. Differences in gene expression of PAKs in 28 tumor tissues and corresponding tissues.

**Additional file 4: Figure S2**. (A) Correlation between mRNA expression and methylation of PAKs. Survival difference between different CNV groups (B), survival analysis showed that there was no significant difference(P<0.05) in survival between ESCA and OV patients with higher CNV levels of some PAKs. The size of the point represents the absolute value of the correlation.

**Additional file 5: Figure S3**. The forest plot of Progression Free Interval and Disease-Specific Survival in pan-cancer patients shows the survival advantages and disadvantages of increased PAK gene expression.

**Additional file 6: Figure S4**. Kaplan–Meier (KM) survival curves revealed that the high- and low-expression group of PAKs had a significant difference in the overall survival and progression free interval.

**Additional file 7: Figure S5**. Kaplan–Meier (KM) survival curves revealed that the high- and low-expression group of PAKs had a significant difference in the overall survival and progression free interval.

**Additional file 8: Figure S6**. Association between PAKs gene expression and drug sensitivity (Z-score from the CellMiner interface) using NCI-60 cell line data.

**Additional file 9: Figure S7**. Association between PAKs gene expression and drug sensitivity (Z-score from the CellMiner interface) using NCI-60 cell line data.

**Additional file 10: Figure S8**. Correlation between CTRP and GDSC drug sensitivity and mRNA expression of PAKs.

**Additional file 11: Figure S9**. Correlation between PAK2, PAK3, PAK4, PAK6, and PAK7 gene expression level and 46 immune checkpoints in pan-cancers.

**Additional file 12: Figure S10**. Correlation between PAK2, PAK3, PAK4, PAK6, and PAK7 gene expression level and 46 immune checkpoints in pan-cancers.

**Additional file 13: Figure S11**. Correlation of PAKs gene expression with tumor mutation burden (TMB) in different tumor types.

**Additional file 14: Figure S12**. Correlation of PAKs gene expression with microsatellite instability (MSI) in different tumor types.

**Additional file15: Figure S13**. Correlation between PAKs expression and several common immune cells and genetic markers of infiltrating immune cells.

**Additional file 16: Figure S14**. Correlation between PAKs expression and several common immune cells and genetic markers of infiltrating immune cells.

**Additional file 17: Figure S15**. Correlation between PAKs expression and several common immune cells and genetic markers of infiltrating immune cells.

**Additional file 18: Figure S16**. Correlation between PAKs gene expression level and different immune-related cell.

**Additional file 19: Figure S17**. Correlation between PAKs gene expression level and different immune-related cell.

**Additional file 20: Figure S18**. Verifying the prognostic stability of PAKs signature in CGGA seq1, CGGA seq2, GSE16011 and Rembrandt datasets.

**Additional file 21: Figure S19**. Nomogram calibration curves were used to predict 1-/3-/5-years survival rates in CGGA seq1, CGGA seq2, GSE16011 datasets.

**Additional file 22: Figure S20**. (A) Kaplan–Meier(K-M) curves of OS of high- and low- subgroups divided by the risk score of GBM patients in the TCGA dataset. (B) The distribution of GBM patients’ survival status, risk scores in the TCGA dataset. (C-D) Univariate and multivariate Cox analyses of different variables, including age, gender, IDH mutational status, and risk scores, in TCGA dataset. (E-G) Receiver operating characteristic (ROC) curves for the PAK1 expression for the prediction of 1/3/5-year survival of GBM patients in the TGGA dataset.

**Additional file 23: Figure S21**. (A) Immunoprecipitation analysis was performed to detect the mutual binding between AKT1 and PAK1 in LN229 cells. (B) Cellular immunofluorescence shows the co-localization of AKT1 and PAK1 in U87 cells. (C-D) Application of qPCR in U87 and LN229 cells to detect the levels of PAK1 and AKT1 after FRAX486 treatment, standardized by GAPDH mRNA expression. (E) Western blot analysis was performed for detecting the protein lysates of PAK1, p-PAK1(T212), AKT1, p-AKT(S473) and GAPDH of LN229 cells after being treated with 10μM MK2206 and 1μM FRAX486 for 24h.

**Additional file 24: Figure S22**. (A) PAK1 and AKT1 mRNA, protein levels of AKT, AKT pS473, AKT pT308 in all tumor cell lines. (B) Western blot analysis was used to detect PAK1 andPAKs in Pan-cancerpAKT levels in NHA, U87MG, and LN229 GBM cells. (C) PCR in NHA, U87MG, and LN229 for detect AKT1 and PAK1 mRNA levels.

**Additional file 25: Table S1**. The coefficient of each PAK gene family member.

**Additional file 26: Table S2**. LGG patient-related information in the four validation datasets.

**Additional file 27: Table S3**. Primer information of PAK1, GAPDH and AKT1.

**Additional file 28: Table S4**. Mutation frequency of PAKs in pan-cancers.

**Additional file 28: Table S5**. The HR and P value between PAKs gene expression with the OS, DFS, PFI, and DSS of patients with different cancer types.

**Additional file 29: Table S6**. Analyzed the survival difference of tumor patients based on methylation degree of PAK1, PAK2, PAK3, PAK4 and PAK6.

**Additional file 30: Table S7**. Divided the expression of PAK1, PAK4, PAK6, and PAK7 gene into high- and low- gene expression groups for GSEA analysis in GBM.

**Additional file 32: Table S8**. Correlation between PAK mRNAs and protein levels of Akt, Akt pS473, and Akt pT308 in all tumor cell lines based on CCLE database.

**Additional file 33: Table S9**. PCR in NHA, U87MG, and LN229 for detect AKT1 and PAK1 mRNA levels.

**Additional file 34: Table S10**. Correlation between PAK mRNAs and protein levels of Akt, Akt pS473, and Akt pT308 in all GBM cell lines; Correlation between PAK1 and AKT1 mRNAs in all tumor cell lines and GBM cell lines.

## Data Availability

The datasets presented in this study can be found in online repositories. The names of the repository/repositories and accession number(s) can be found in the article/Supplementary Material.

## Abbreviations

PAKs: P21-activated kinase family
TCGA: The Cancer Genome Atlas
GSEA: Gene set enrichment analysis
GTEx: The Genotype-Tissue Expression
FPKM: Fragments per kilobase million
TPM: Transcripts per million
CGGA: Chinese Glioma Genome Atlas
GEO: Gene Expression Omnibus
GSCA: Gene Set Cancer Analysis
GDSC: Genomics of Drug Sensitivity in Cancer
CTRP: Cancer Therapeutics Response Portal
ROC: Receiver operating characteristic
ssGSEA: Single-sample gene-set enrichment analysis
ATCC: American Type Culture Collection
ESTIMATE: Estimation of stromal and immune cells in malignant tumor tissues using expression data
ICP: Immune checkpoint
TMB: Tumor mutational burden
MSI: Microsatellite instability
TME: Tumor microenvironment
OS: Overall survival
PFI: Progression-free interval
DFS: Disease-free survival
DSS: Disease-specific survival
ACC: Adrenocortical carcinoma
BLCA: Bladder urothelial carcinoma
BRCA: Breast invasive carcinoma
CHOL: Cholangiocarcinoma
COAD: Colon adenocarcinoma
DLBC: Lymphoid neoplasm diffuse large B-cell lymphoma
ESCA: Esophageal carcinoma
GBM: Glioblastoma multiforme
LASSO: Least absolute shrinkage and selection operator
HNSC: Head and Neck squamous cell carcinoma
KICH: Kidney chromophobe
KIRC: Kidney renal clear cell carcinoma
KIRP: Kidney renal papillary cell carcinoma
LAML: Acute Myeloid Leukemia
LGG: Brain lower grade glioma
LIHC: Liver hepatocellular carcinoma
LUAD: Lung adenocarcinoma
LUSC: Lung squamous cell carcinoma
OV: Ovarian serous cystadenocarcinoma
PAAD: Pancreatic adenocarcinoma
PRAD: Prostate adenocarcinoma
READ: Rectum adenocarcinoma
SARC: Sarcoma
SKCM: Skin cutaneous melanoma
STAD: Stomach adenocarcinoma
TGCT: Testicular germ cell tumors
THCA: Thyroid carcinoma
UCEC: Uterine corpus endometrial carcinoma
UCS: Uterine carcinosarcoma
