# Supplementary figures and images for: Comprehensive analysis of the prognostic implications and functional exploration of PAK gene family in human cancer (part 2 of 2)
Source: Cancer Cell Int. 2022 Sep 5;22:275. doi: 10.1186/s12935-022-02689-6 (PMC9442929; doi:10.1186/s12935-022-02689-6)

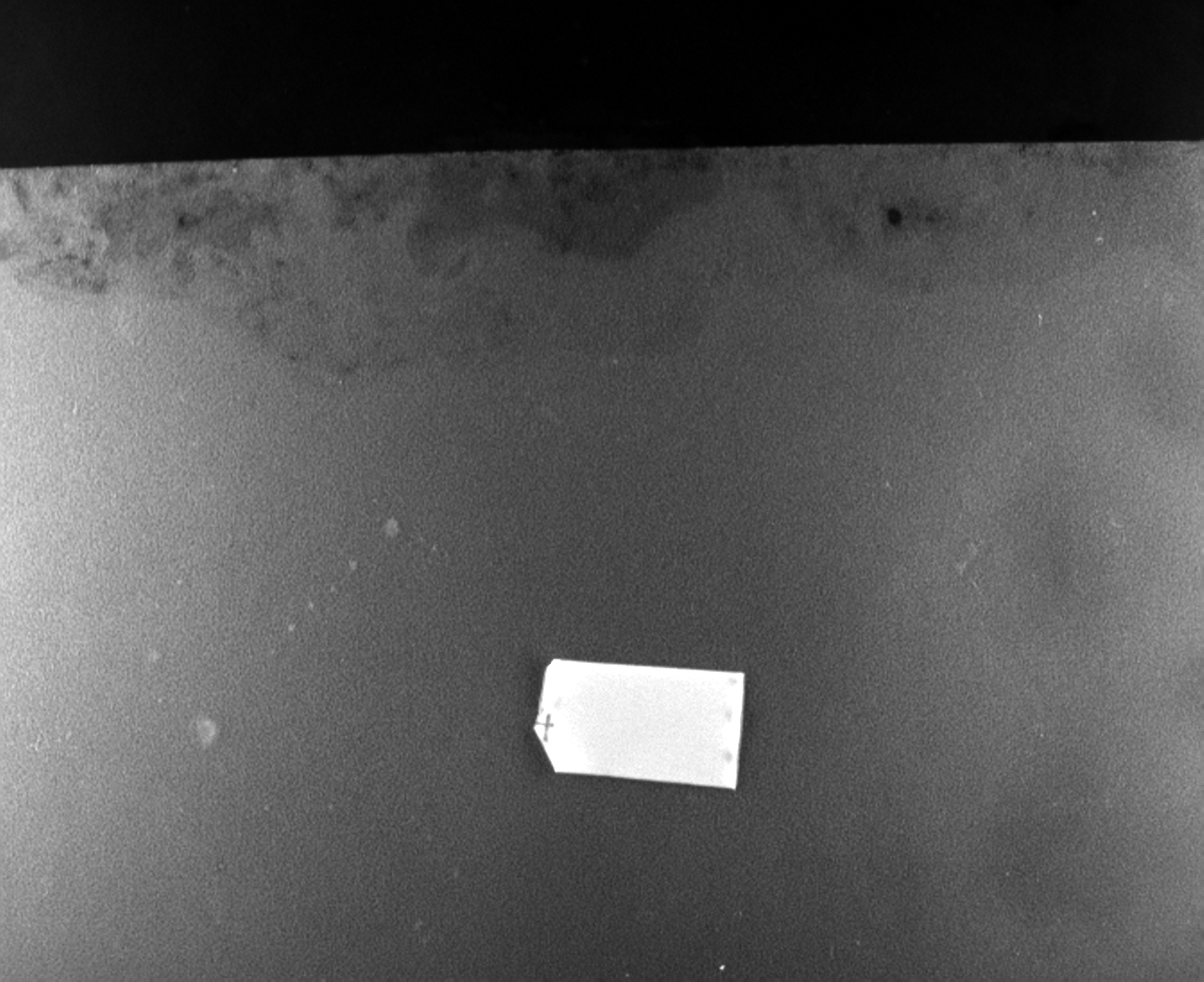

Supplement: Supplementary file 2 — Additional file 2: The raw experimental data related to this study. [file 12935_2022_2689_MOESM2_ESM.zip › WB/pPAK/87-2-2.Tif]

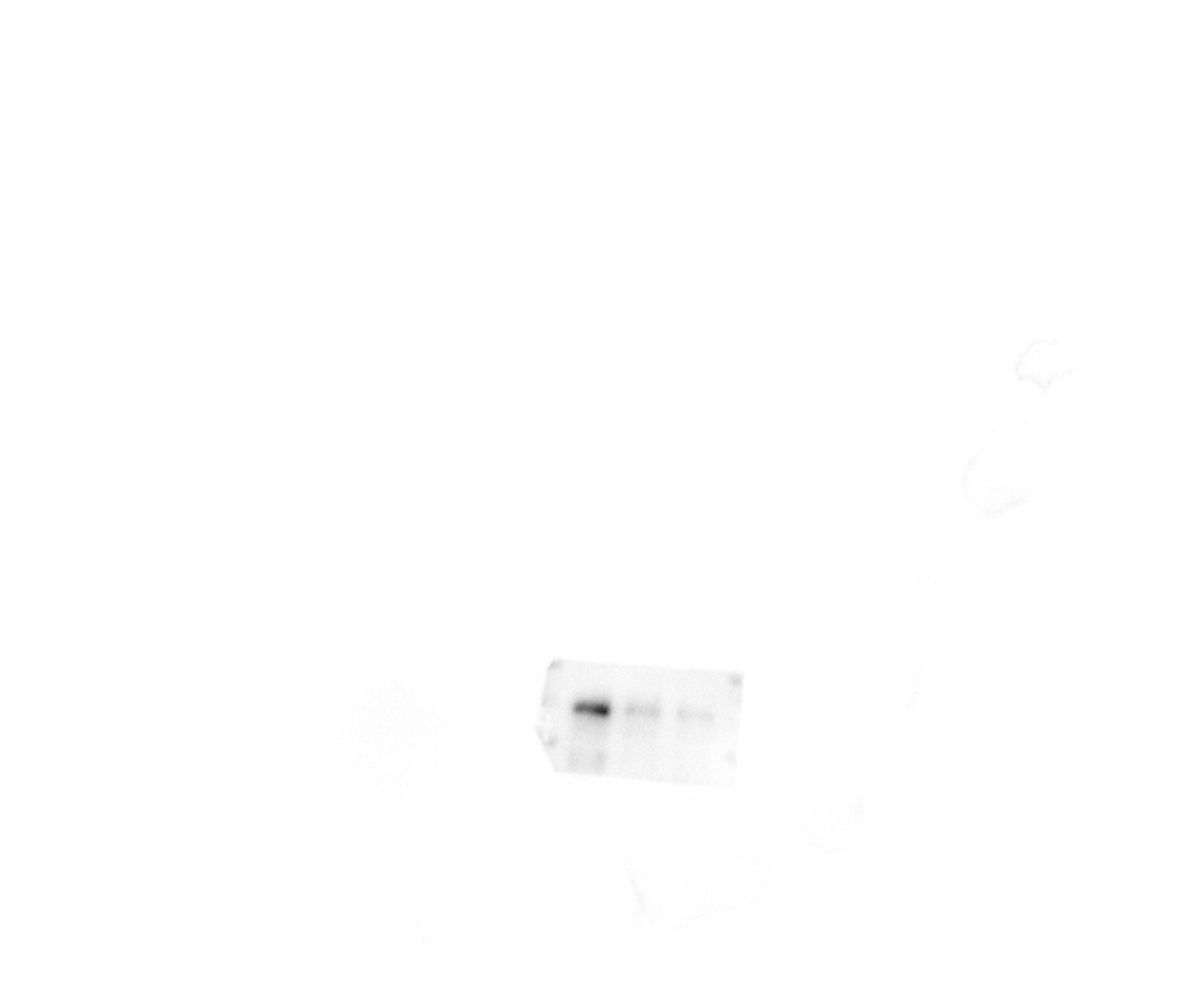

Supplement: Supplementary file 2 — Additional file 2: The raw experimental data related to this study. [file 12935_2022_2689_MOESM2_ESM.zip › WB/pPAK/87-2.Tif]

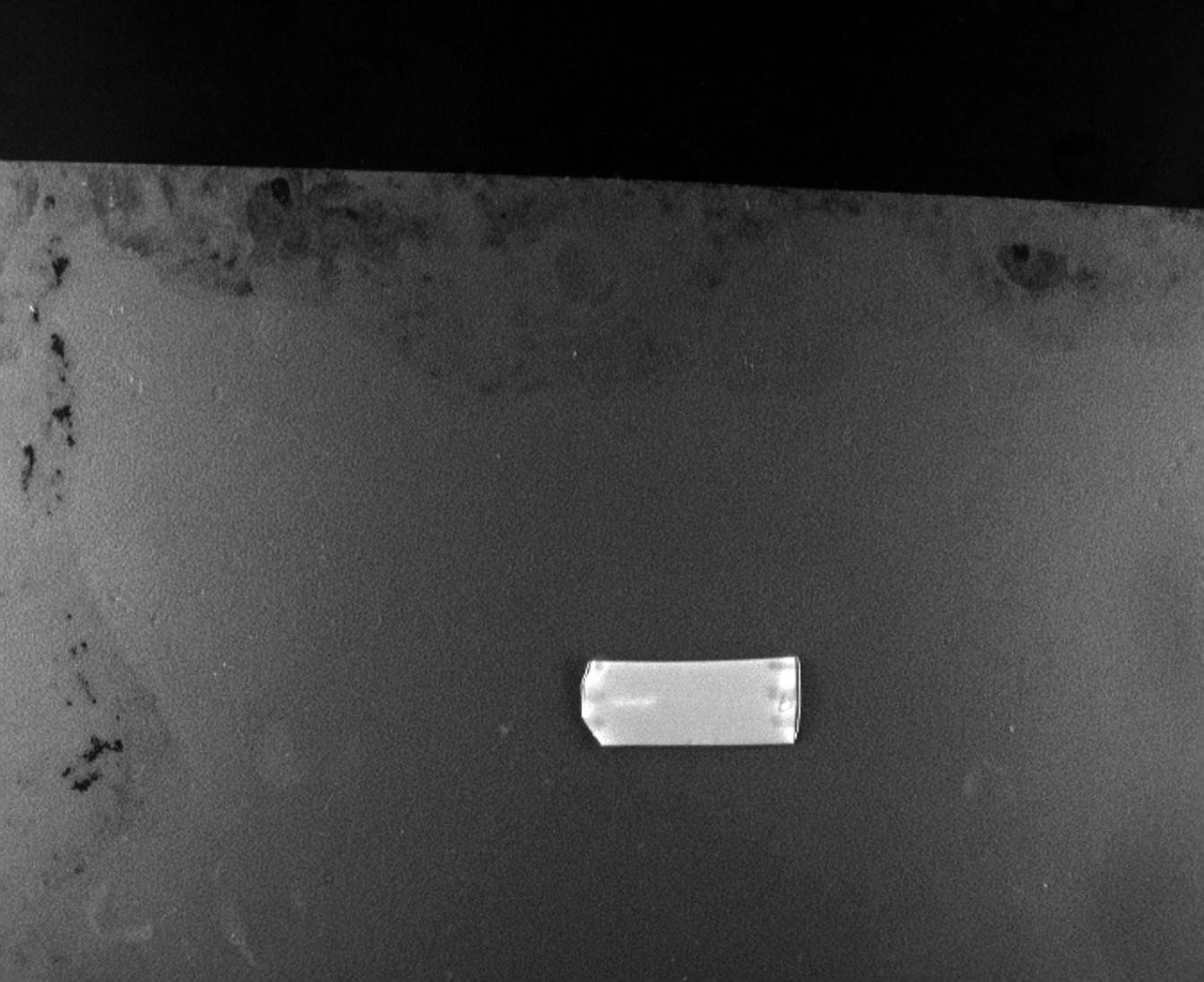

Supplement: Supplementary file 2 — Additional file 2: The raw experimental data related to this study. [file 12935_2022_2689_MOESM2_ESM.zip › WB/pPAK/87-3-2.Tif]

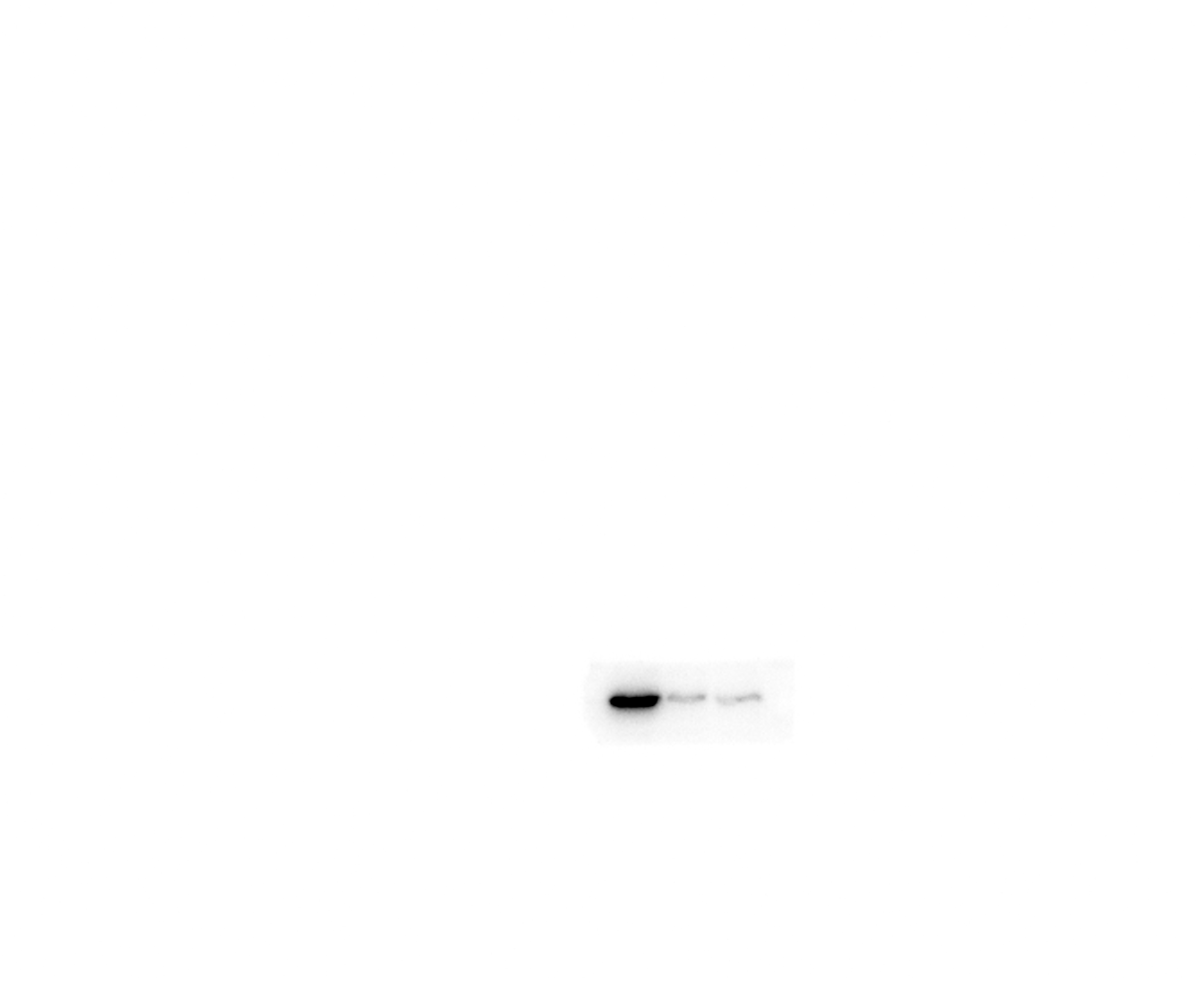

Supplement: Supplementary file 2 — Additional file 2: The raw experimental data related to this study. [file 12935_2022_2689_MOESM2_ESM.zip › WB/pPAK/87-3.Tif]

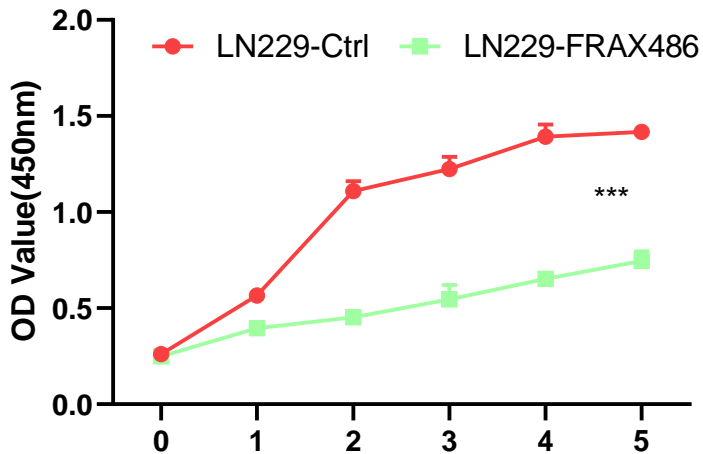

Supplement: Supplementary file 2 — Additional file 2: The raw experimental data related to this study. [file 12935_2022_2689_MOESM2_ESM.zip › CCK8/229.pdf]

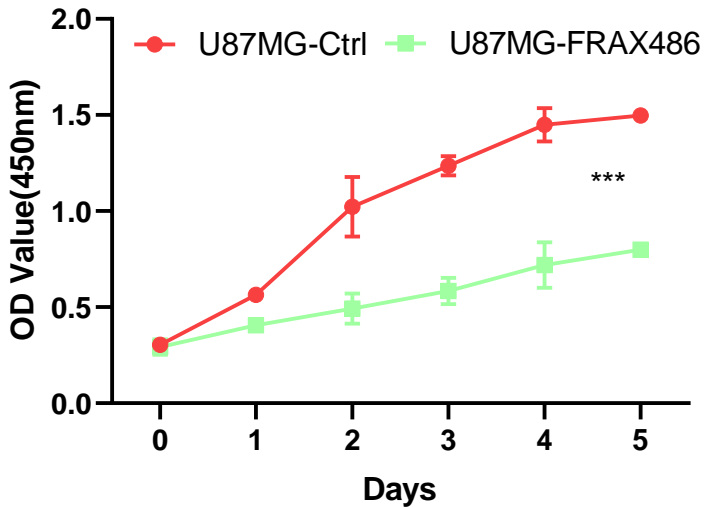

Supplement: Supplementary file 2 — Additional file 2: The raw experimental data related to this study. [file 12935_2022_2689_MOESM2_ESM.zip › CCK8/87.pdf]

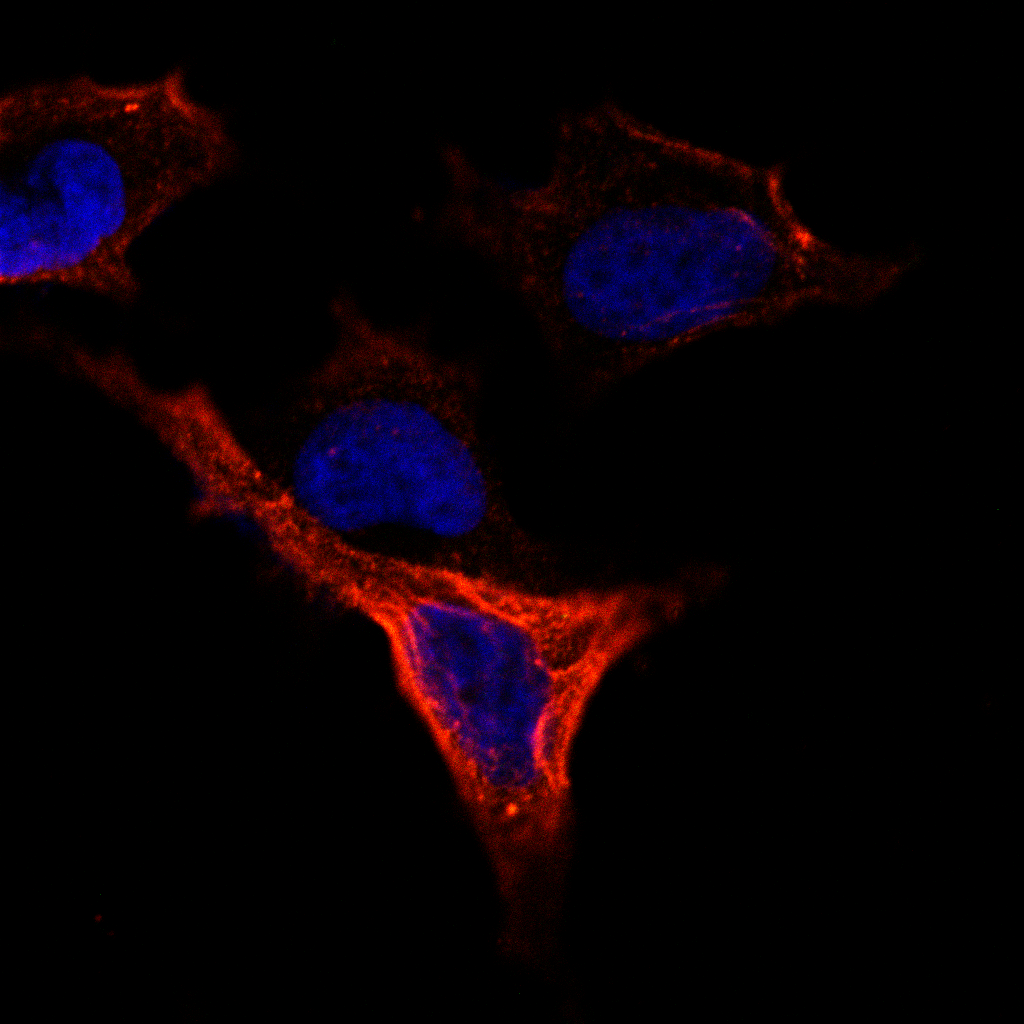

Supplement: Supplementary file 2 — Additional file 2: The raw experimental data related to this study. [file 12935_2022_2689_MOESM2_ESM.zip › Confocal/Export_229-AKT1+PAK1-1_RGB.tif]

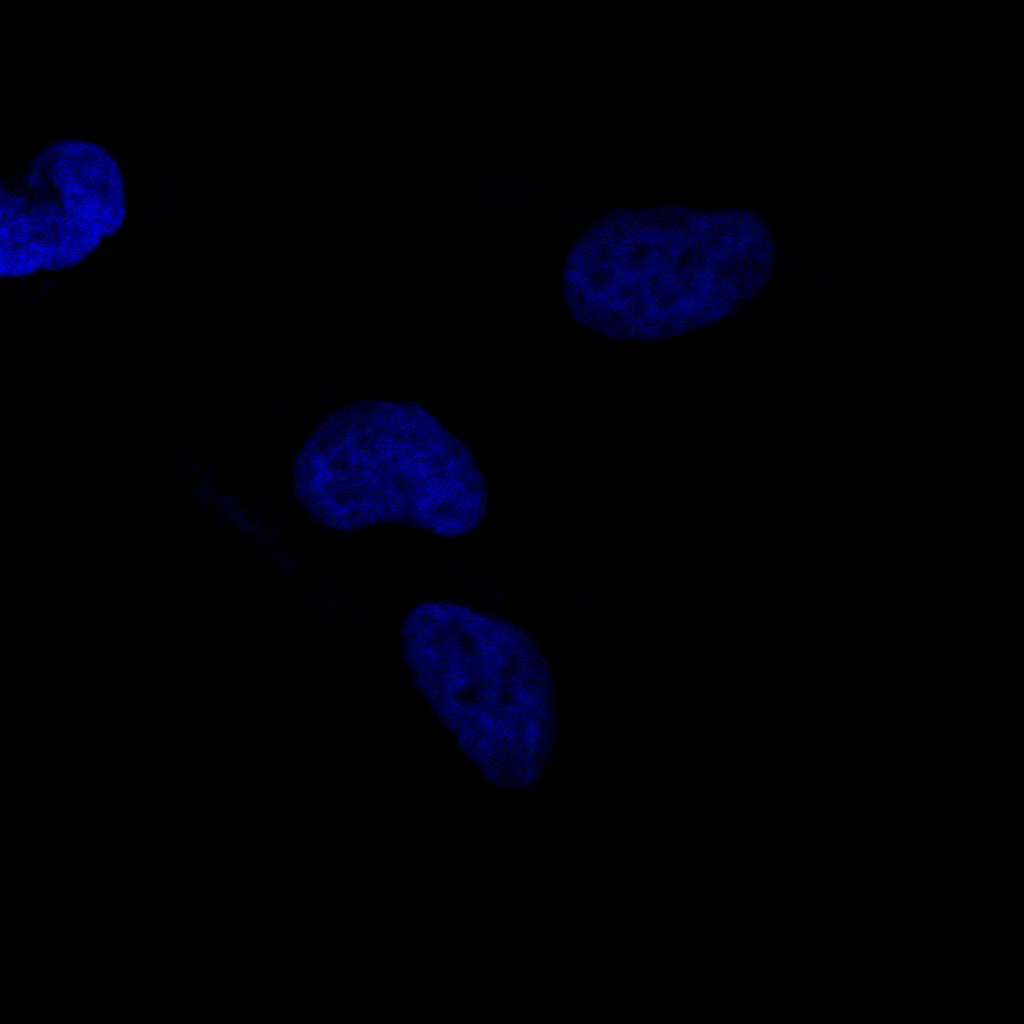

Supplement: Supplementary file 2 — Additional file 2: The raw experimental data related to this study. [file 12935_2022_2689_MOESM2_ESM.zip › Confocal/Export_229-AKT1+PAK1-1_RGB_DAPI.tif]

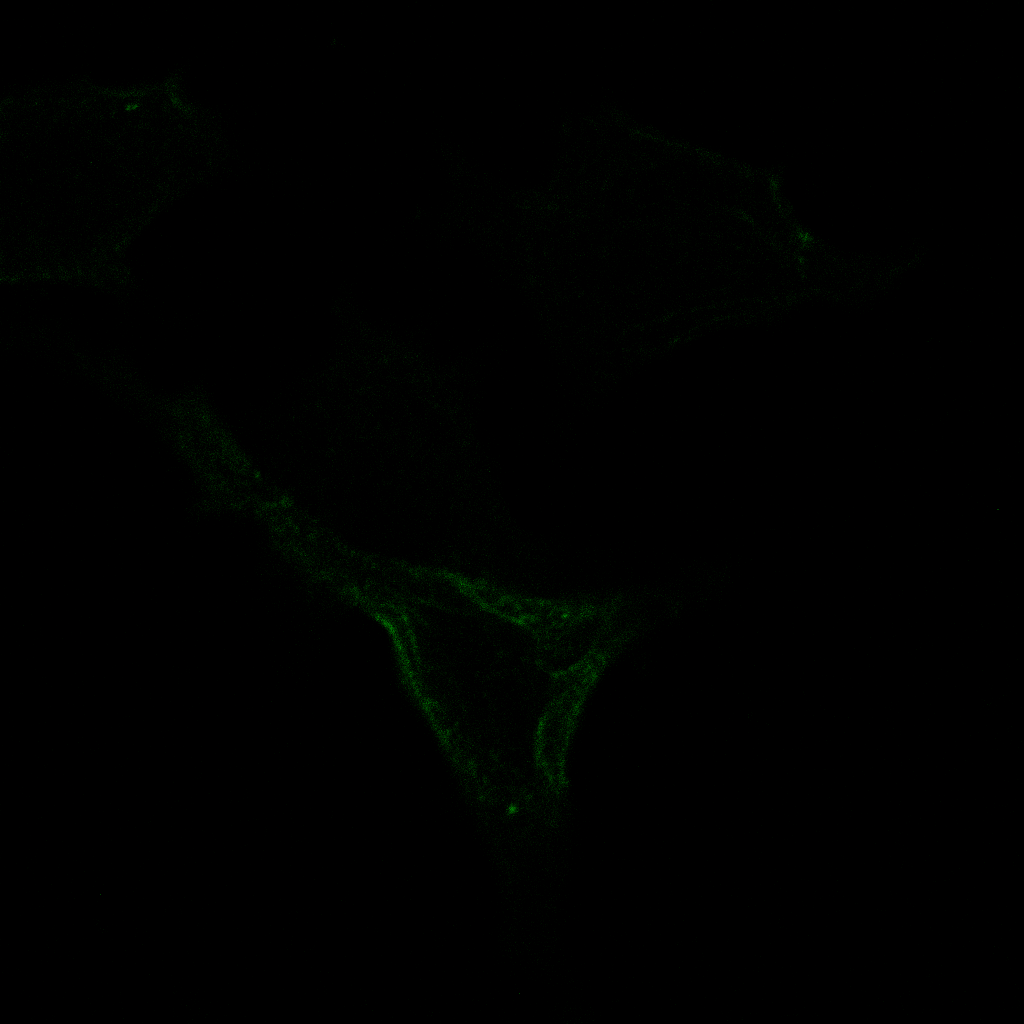

Supplement: Supplementary file 2 — Additional file 2: The raw experimental data related to this study. [file 12935_2022_2689_MOESM2_ESM.zip › Confocal/Export_229-AKT1+PAK1-1_RGB_FITC.tif]

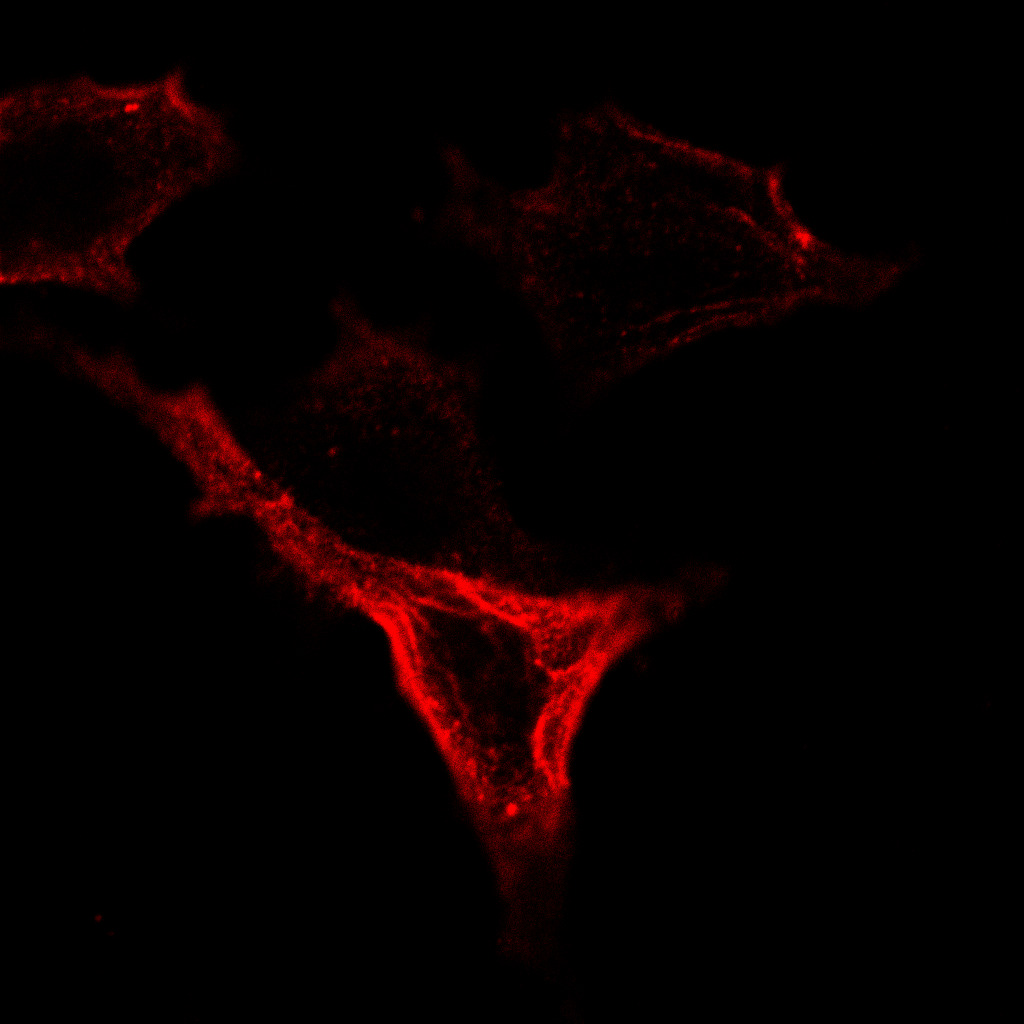

Supplement: Supplementary file 2 — Additional file 2: The raw experimental data related to this study. [file 12935_2022_2689_MOESM2_ESM.zip › Confocal/Export_229-AKT1+PAK1-1_RGB_TRITC.tif]

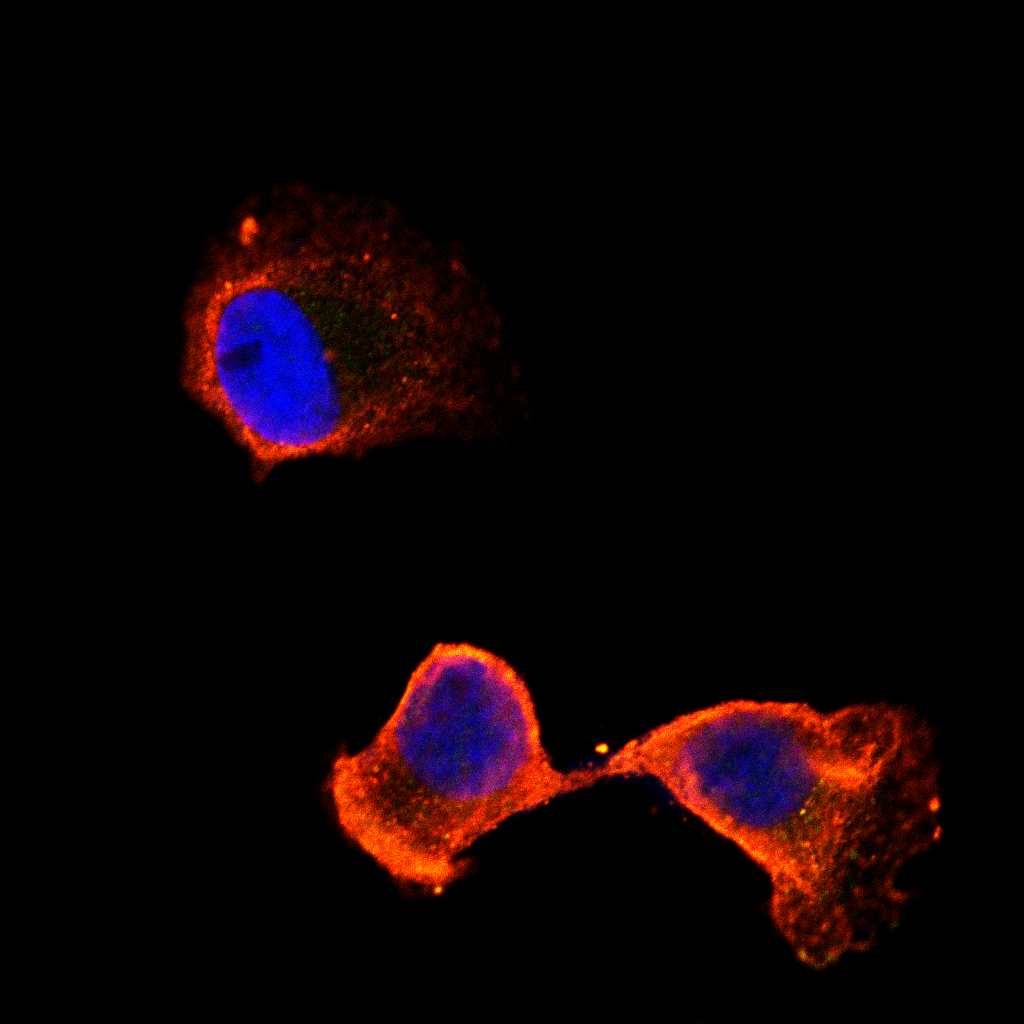

Supplement: Supplementary file 2 — Additional file 2: The raw experimental data related to this study. [file 12935_2022_2689_MOESM2_ESM.zip › Confocal/Export_229-AKT1+PAK1-2_RGB.tif]

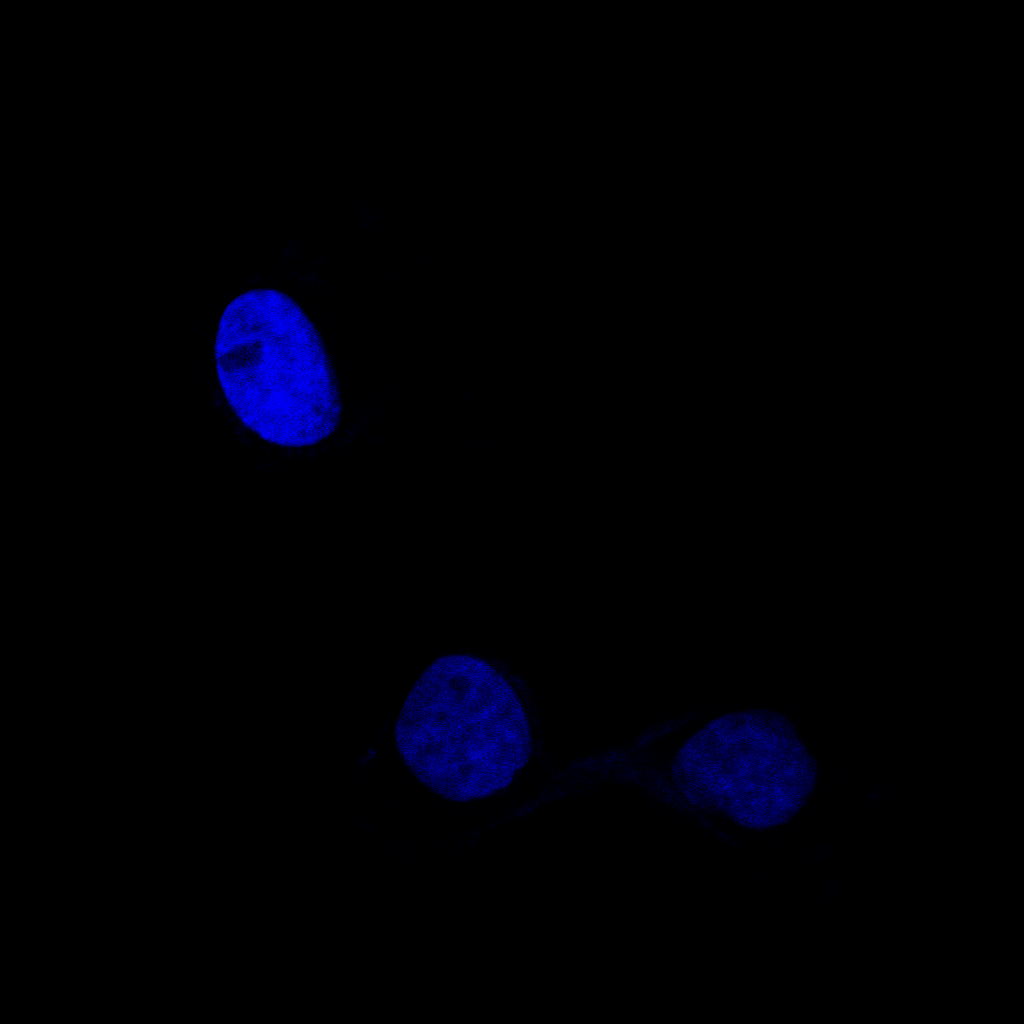

Supplement: Supplementary file 2 — Additional file 2: The raw experimental data related to this study. [file 12935_2022_2689_MOESM2_ESM.zip › Confocal/Export_229-AKT1+PAK1-2_RGB_DAPI.tif]

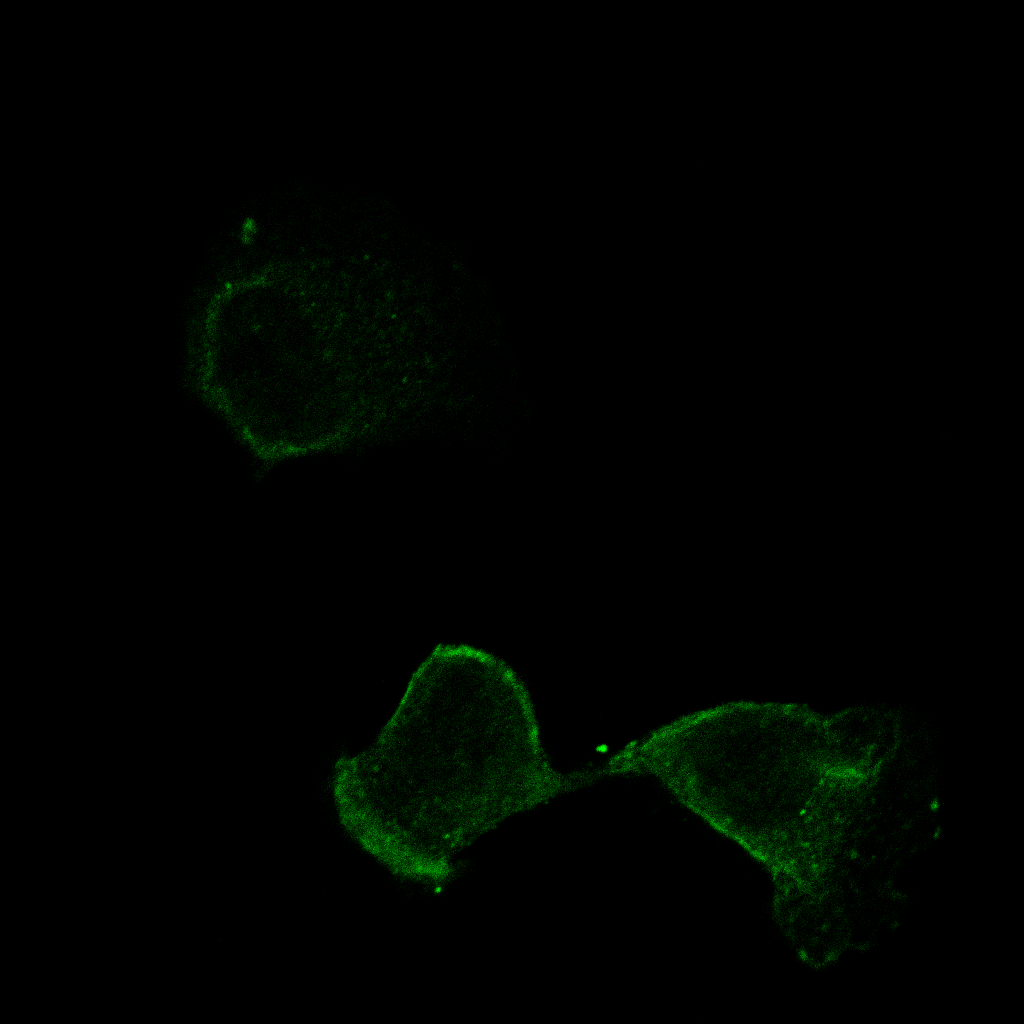

Supplement: Supplementary file 2 — Additional file 2: The raw experimental data related to this study. [file 12935_2022_2689_MOESM2_ESM.zip › Confocal/Export_229-AKT1+PAK1-2_RGB_FITC.tif]

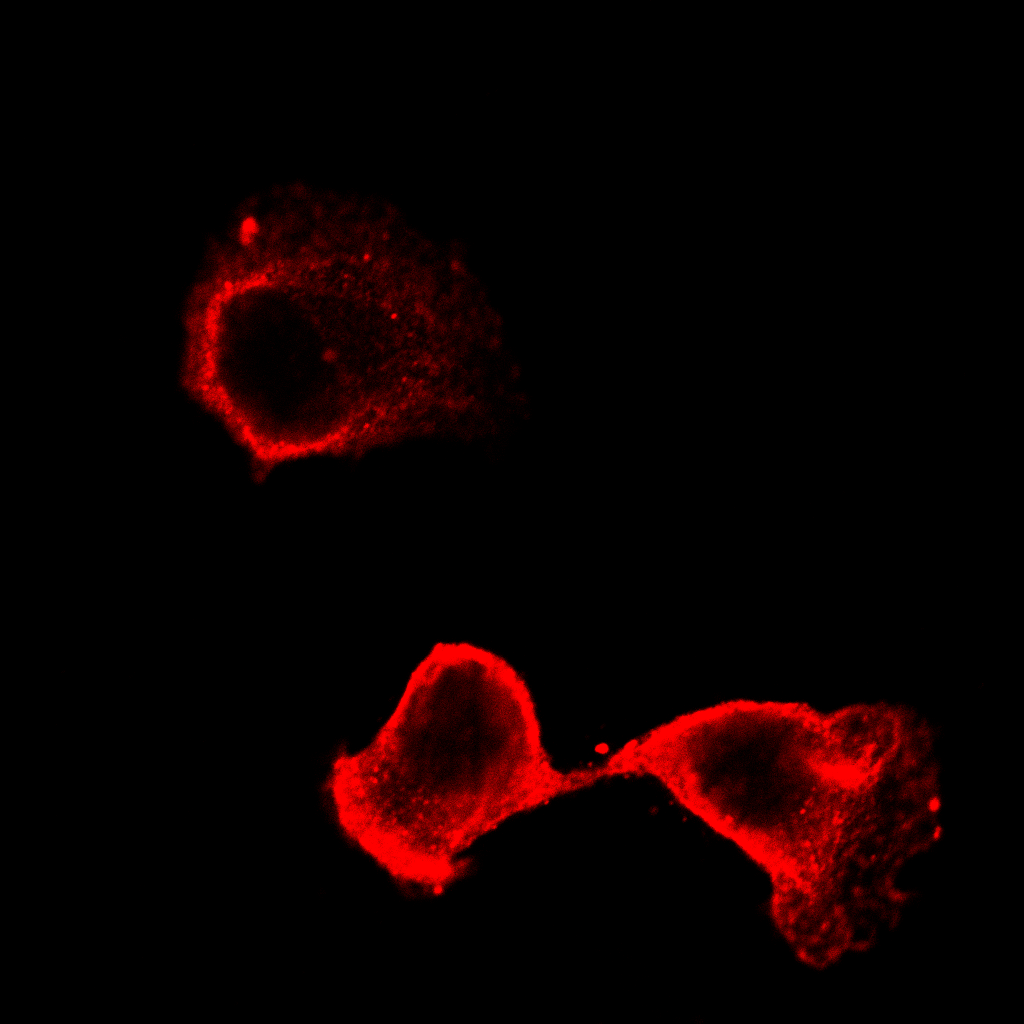

Supplement: Supplementary file 2 — Additional file 2: The raw experimental data related to this study. [file 12935_2022_2689_MOESM2_ESM.zip › Confocal/Export_229-AKT1+PAK1-2_RGB_TRITC.tif]

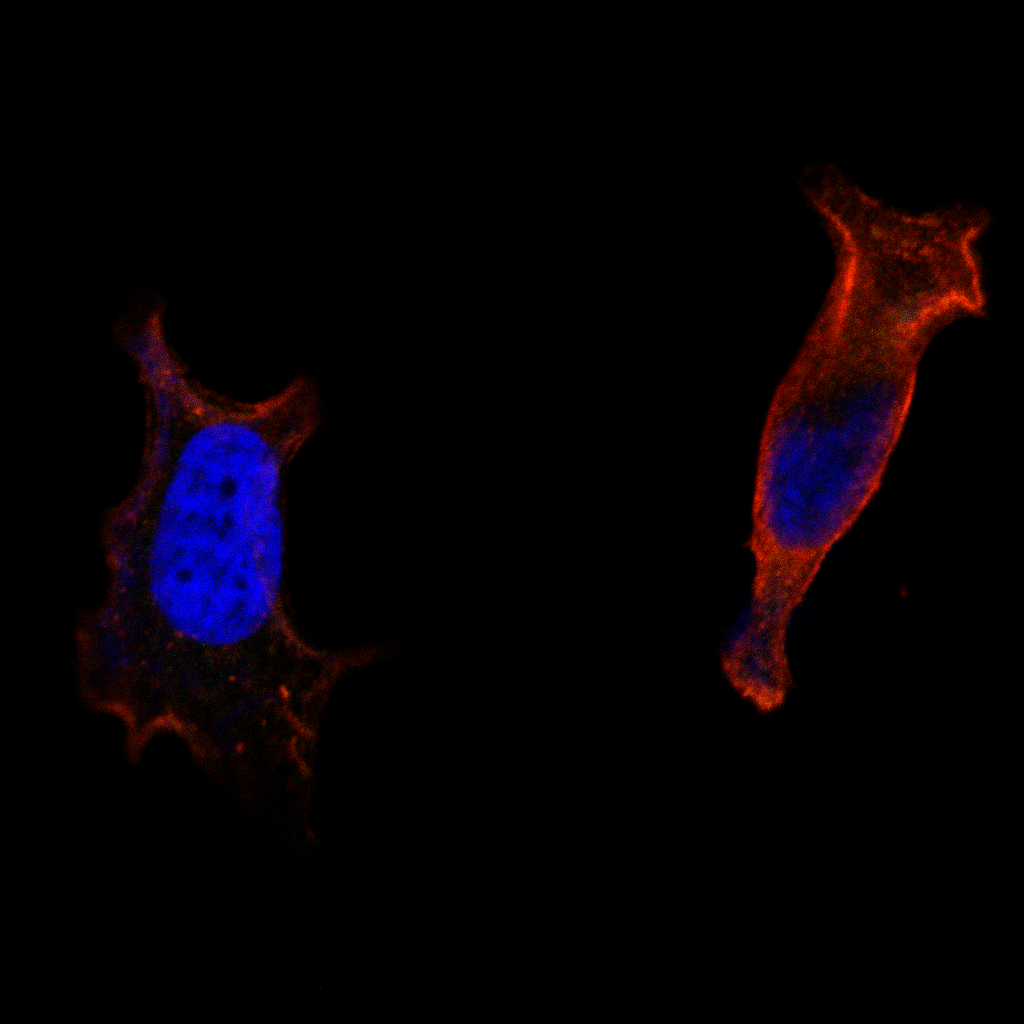

Supplement: Supplementary file 2 — Additional file 2: The raw experimental data related to this study. [file 12935_2022_2689_MOESM2_ESM.zip › Confocal/Export_229-AKT1+PAK1-3_RGB.tif]

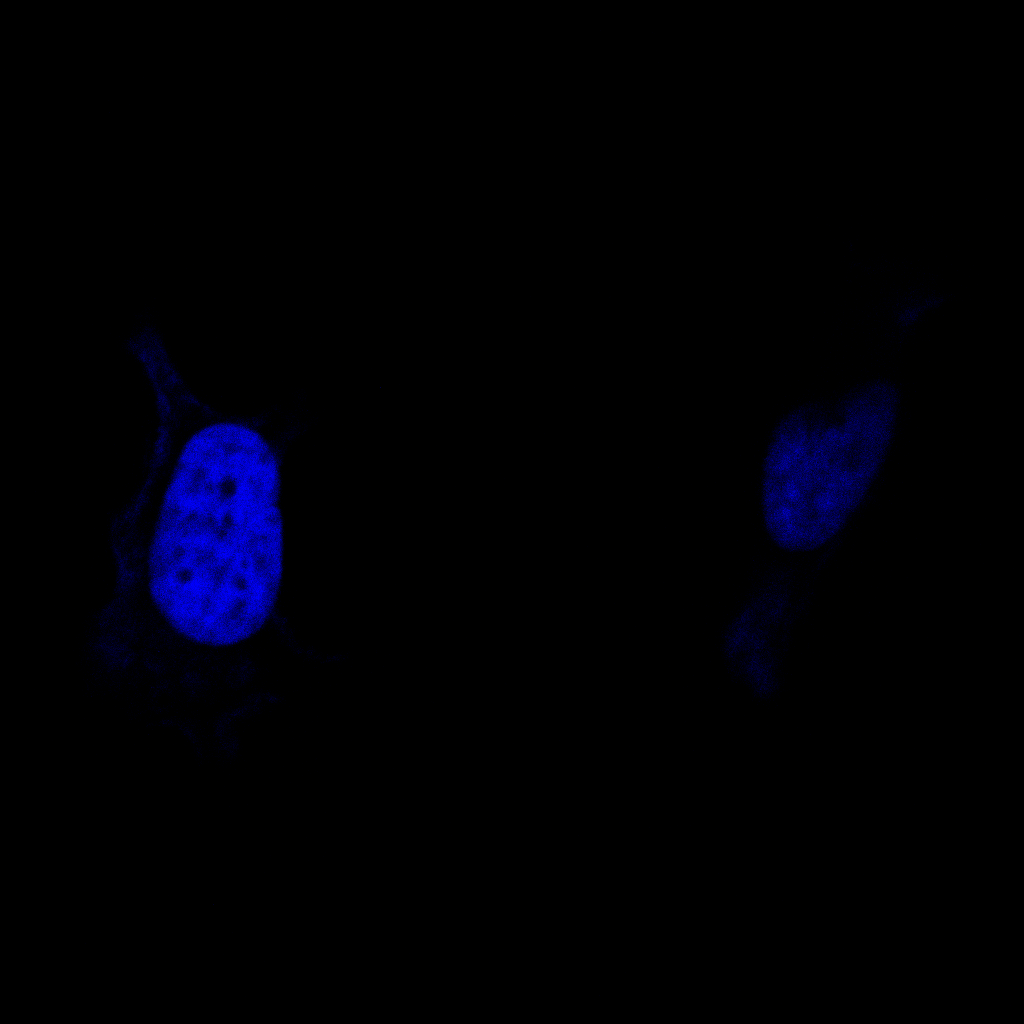

Supplement: Supplementary file 2 — Additional file 2: The raw experimental data related to this study. [file 12935_2022_2689_MOESM2_ESM.zip › Confocal/Export_229-AKT1+PAK1-3_RGB_DAPI.tif]

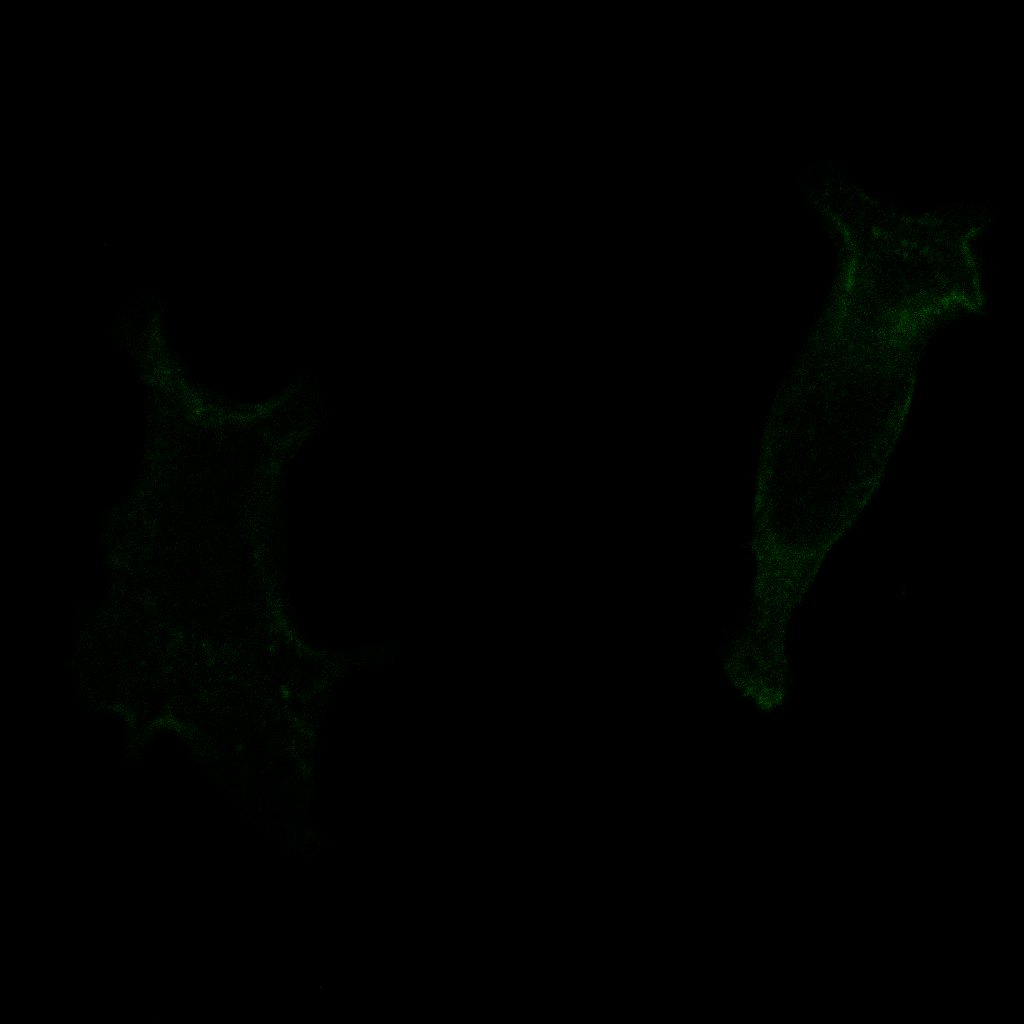

Supplement: Supplementary file 2 — Additional file 2: The raw experimental data related to this study. [file 12935_2022_2689_MOESM2_ESM.zip › Confocal/Export_229-AKT1+PAK1-3_RGB_FITC.tif]

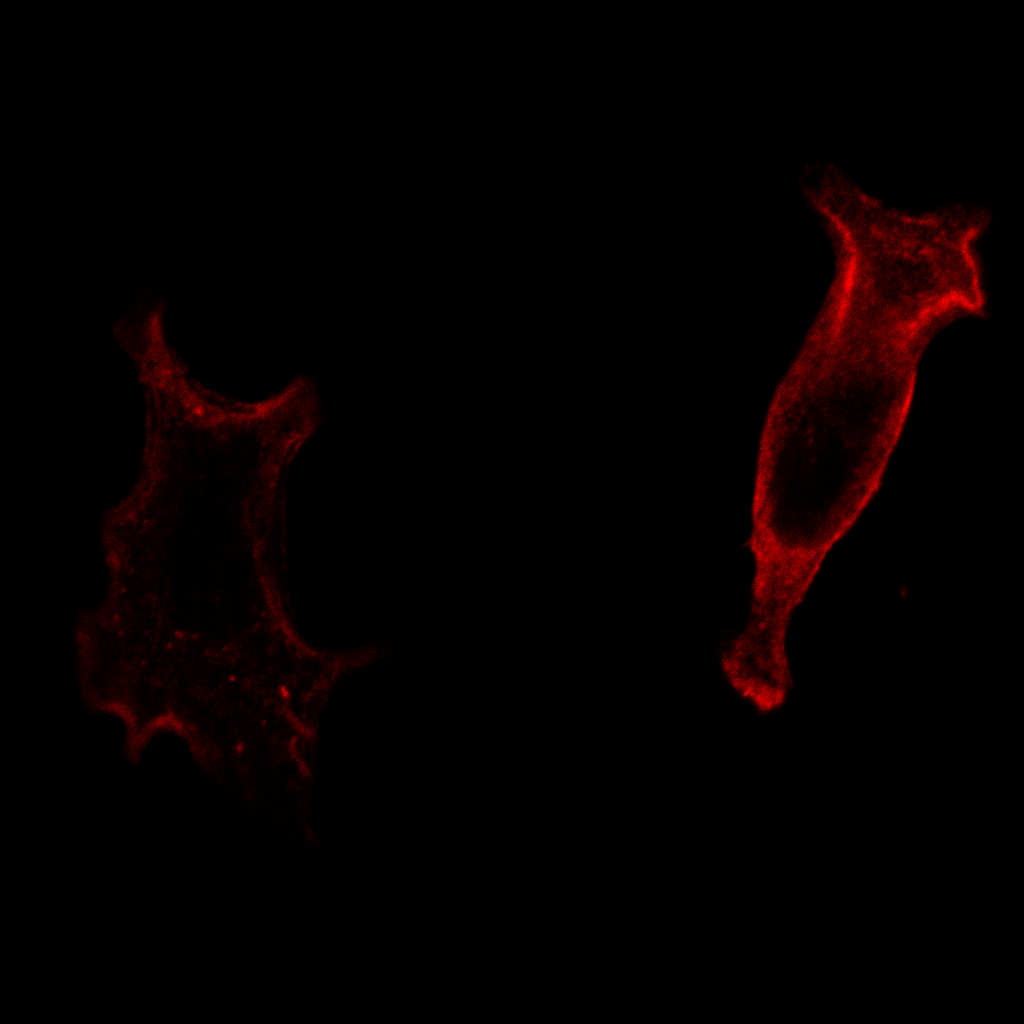

Supplement: Supplementary file 2 — Additional file 2: The raw experimental data related to this study. [file 12935_2022_2689_MOESM2_ESM.zip › Confocal/Export_229-AKT1+PAK1-3_RGB_TRITC.tif]

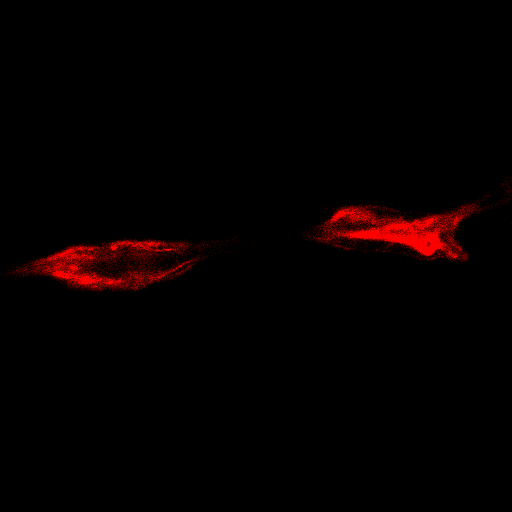

Supplement: Supplementary file 2 — Additional file 2: The raw experimental data related to this study. [file 12935_2022_2689_MOESM2_ESM.zip › Confocal/Export_229-AKT1+PAK1-4_RGB.tif]

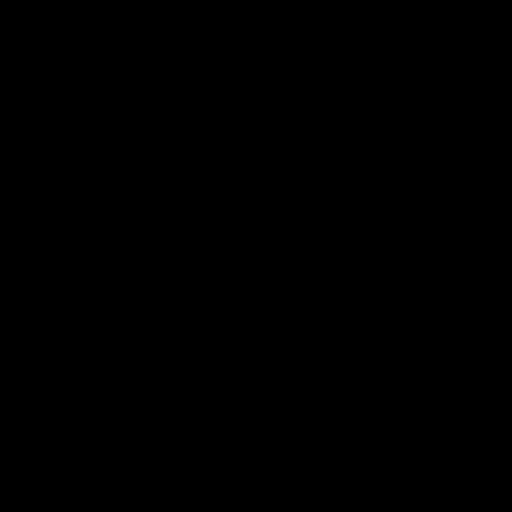

Supplement: Supplementary file 2 — Additional file 2: The raw experimental data related to this study. [file 12935_2022_2689_MOESM2_ESM.zip › Confocal/Export_229-AKT1+PAK1-4_RGB_DAPI.tif]

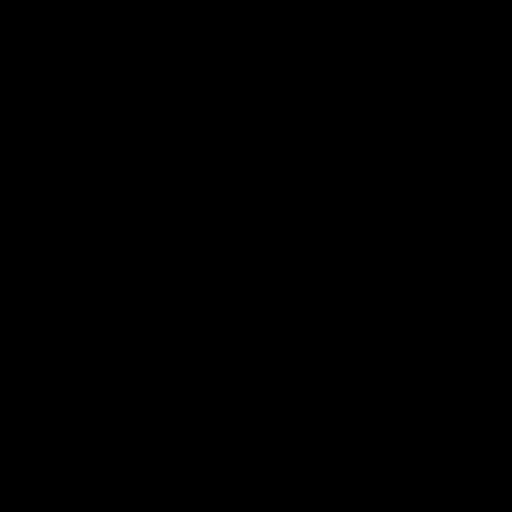

Supplement: Supplementary file 2 — Additional file 2: The raw experimental data related to this study. [file 12935_2022_2689_MOESM2_ESM.zip › Confocal/Export_229-AKT1+PAK1-4_RGB_FITC.tif]

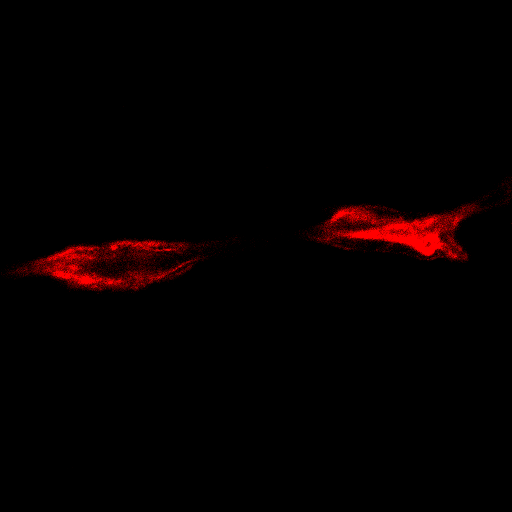

Supplement: Supplementary file 2 — Additional file 2: The raw experimental data related to this study. [file 12935_2022_2689_MOESM2_ESM.zip › Confocal/Export_229-AKT1+PAK1-4_RGB_TRITC.tif]

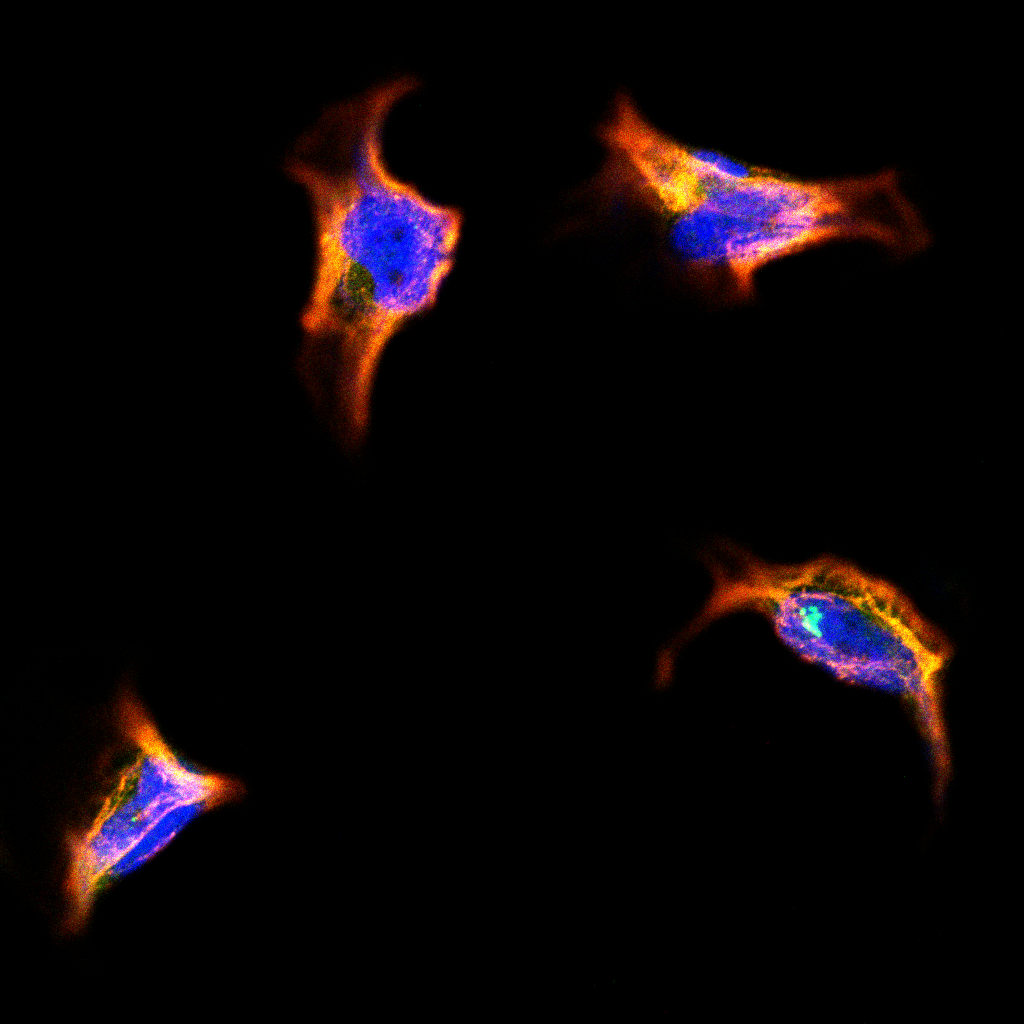

Supplement: Supplementary file 2 — Additional file 2: The raw experimental data related to this study. [file 12935_2022_2689_MOESM2_ESM.zip › Confocal/Export_87-AKT1+PAK1-1_RGB.tif]

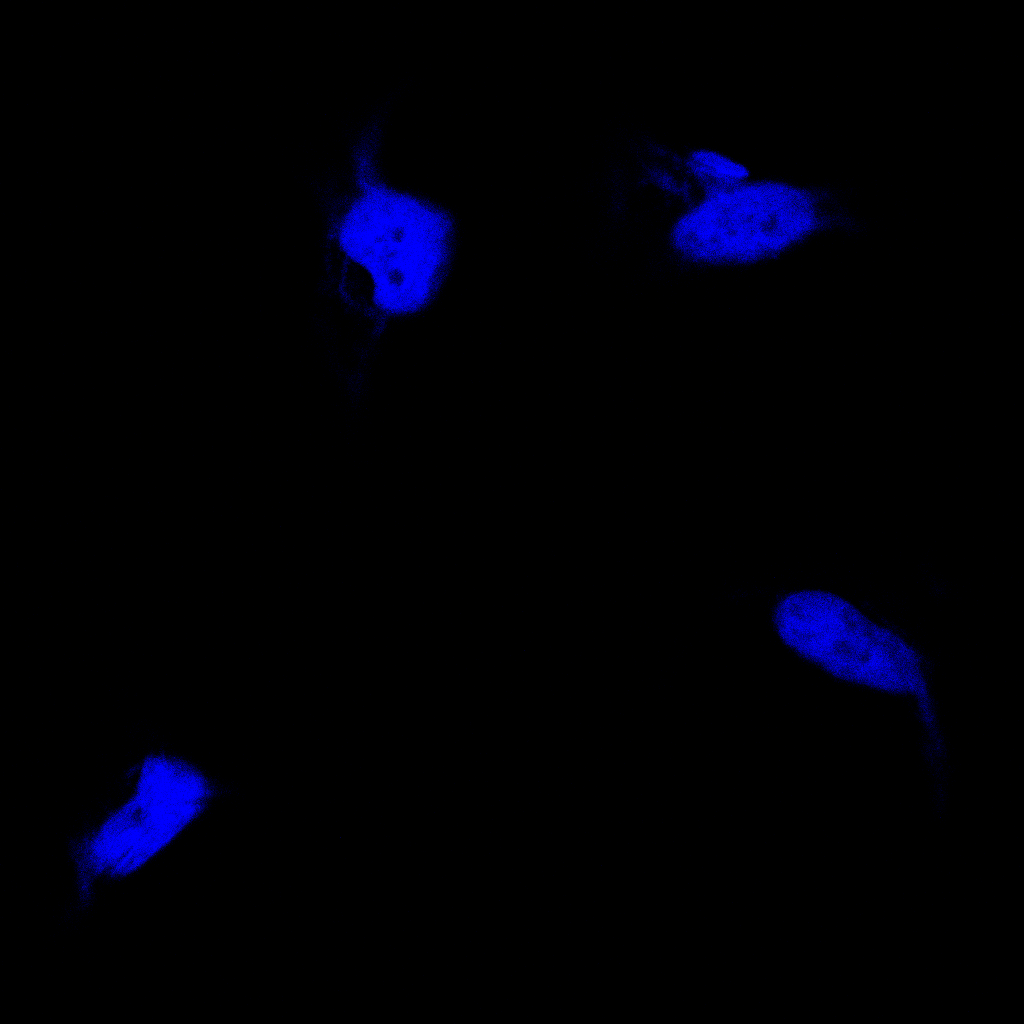

Supplement: Supplementary file 2 — Additional file 2: The raw experimental data related to this study. [file 12935_2022_2689_MOESM2_ESM.zip › Confocal/Export_87-AKT1+PAK1-1_RGB_DAPI.tif]

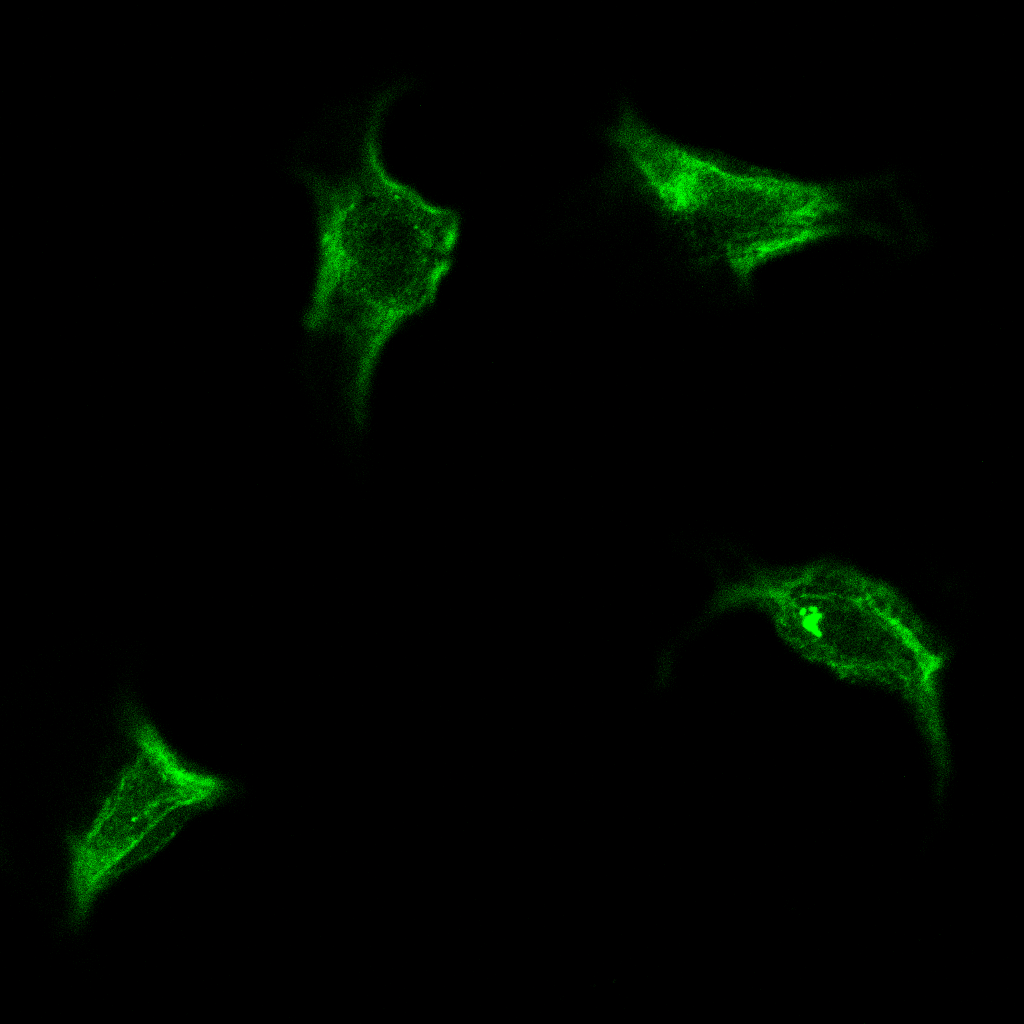

Supplement: Supplementary file 2 — Additional file 2: The raw experimental data related to this study. [file 12935_2022_2689_MOESM2_ESM.zip › Confocal/Export_87-AKT1+PAK1-1_RGB_FITC.tif]

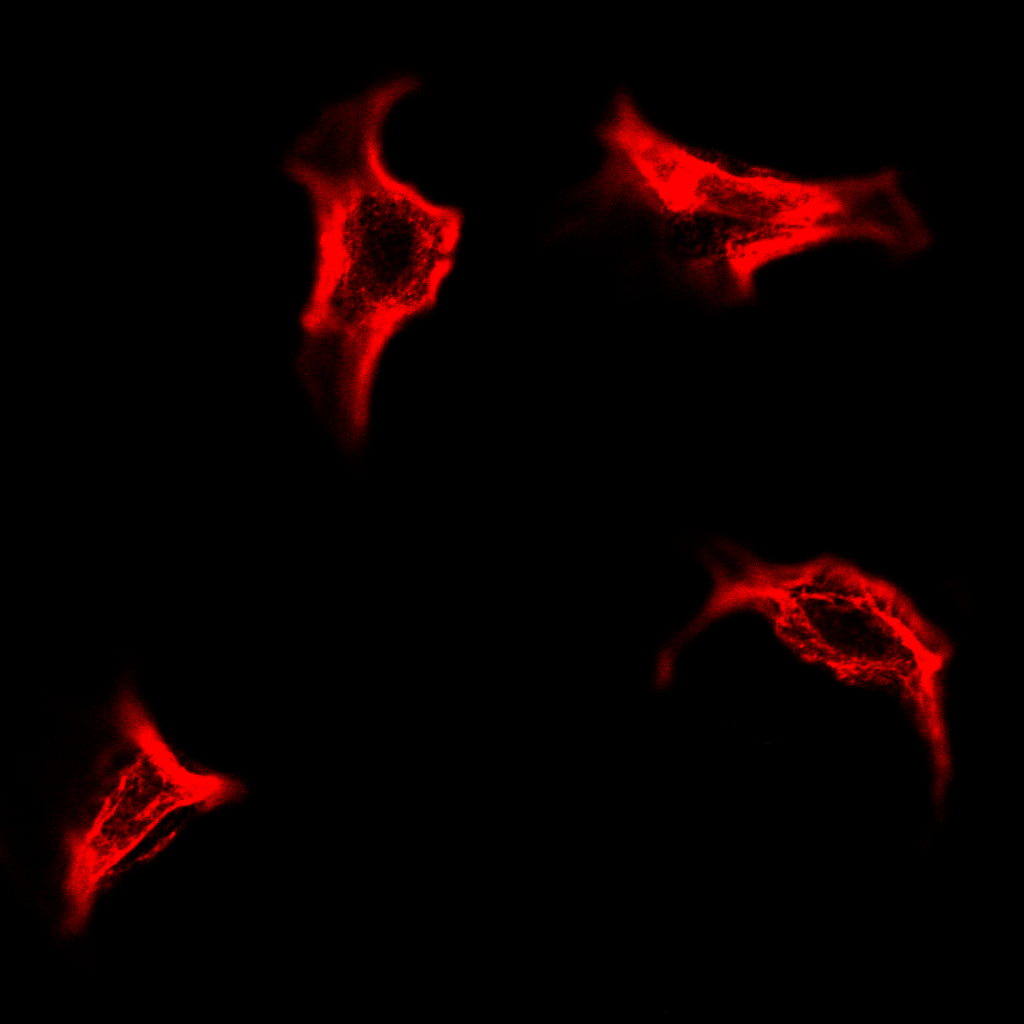

Supplement: Supplementary file 2 — Additional file 2: The raw experimental data related to this study. [file 12935_2022_2689_MOESM2_ESM.zip › Confocal/Export_87-AKT1+PAK1-1_RGB_TRITC.tif]

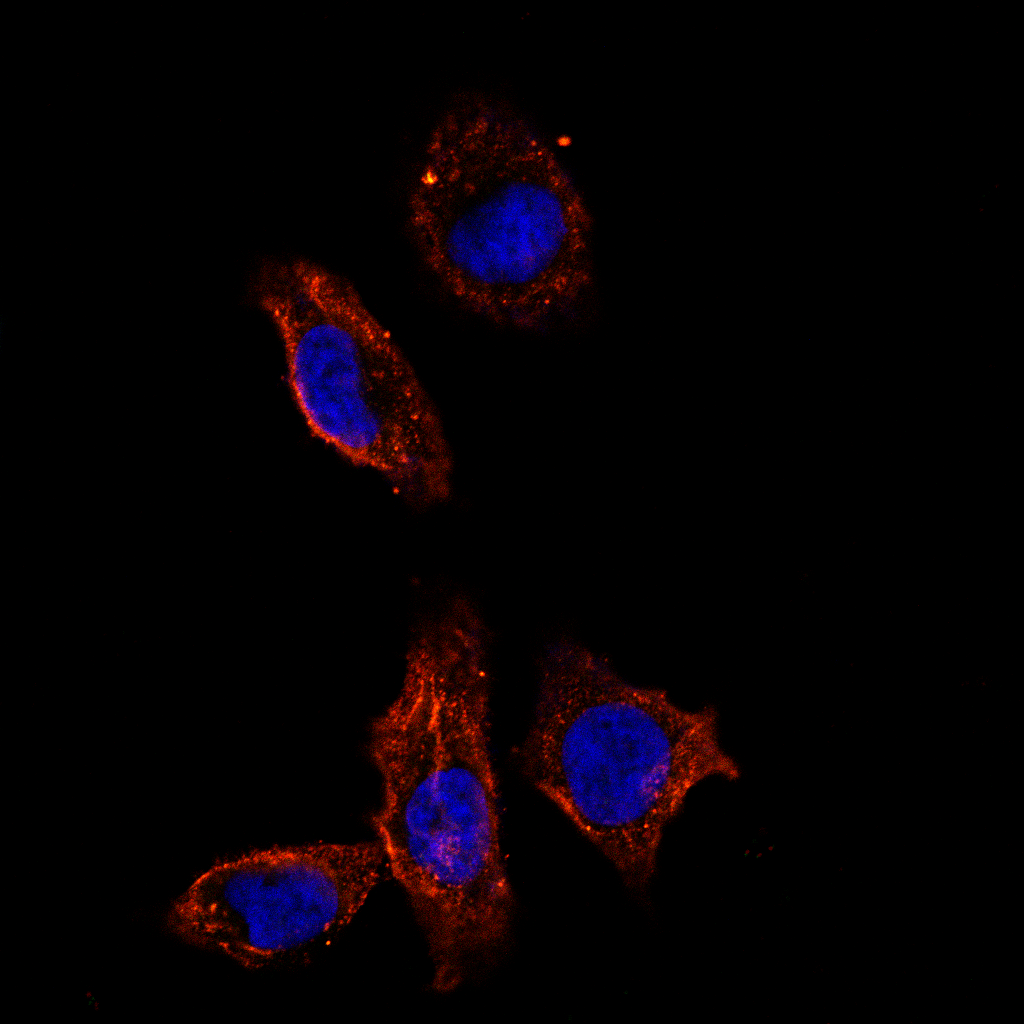

Supplement: Supplementary file 2 — Additional file 2: The raw experimental data related to this study. [file 12935_2022_2689_MOESM2_ESM.zip › Confocal/Export_87-AKT1+PAK1-2_RGB.tif]

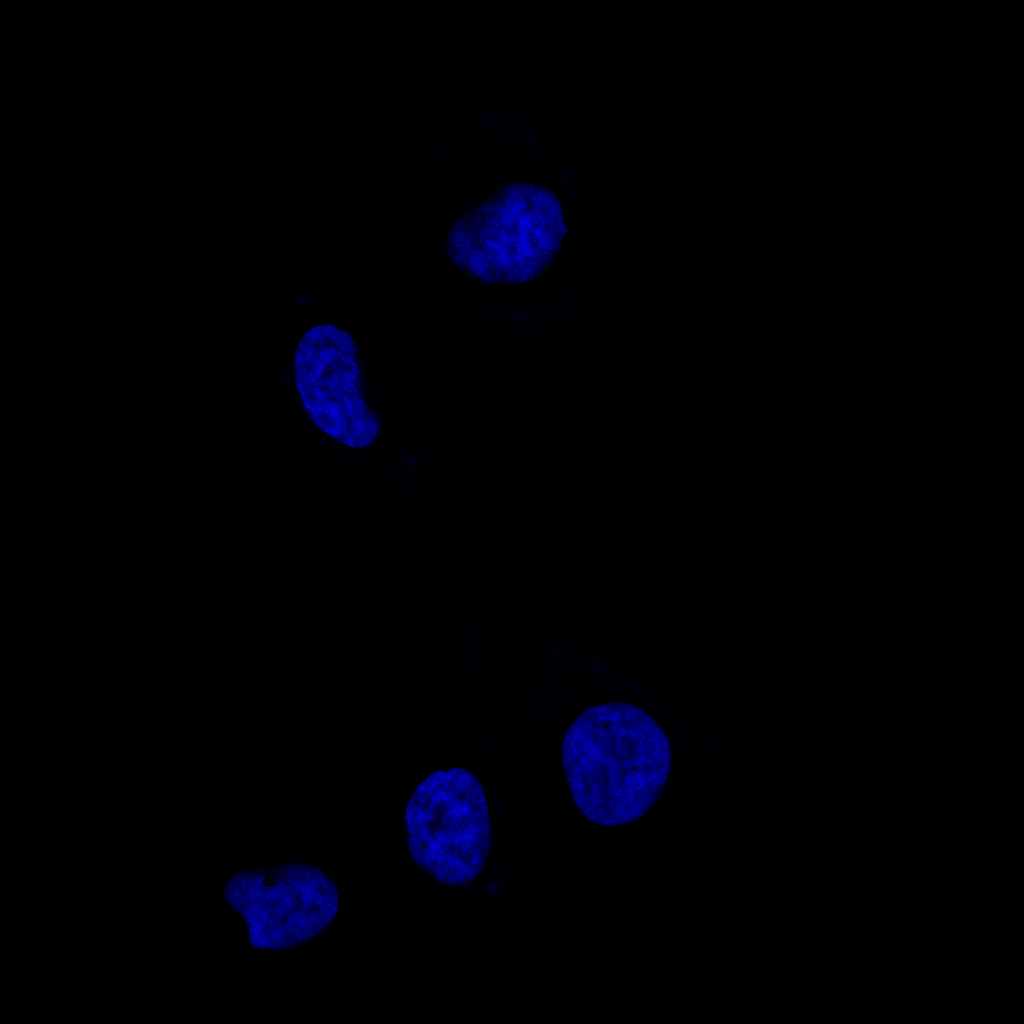

Supplement: Supplementary file 2 — Additional file 2: The raw experimental data related to this study. [file 12935_2022_2689_MOESM2_ESM.zip › Confocal/Export_87-AKT1+PAK1-2_RGB_DAPI.tif]

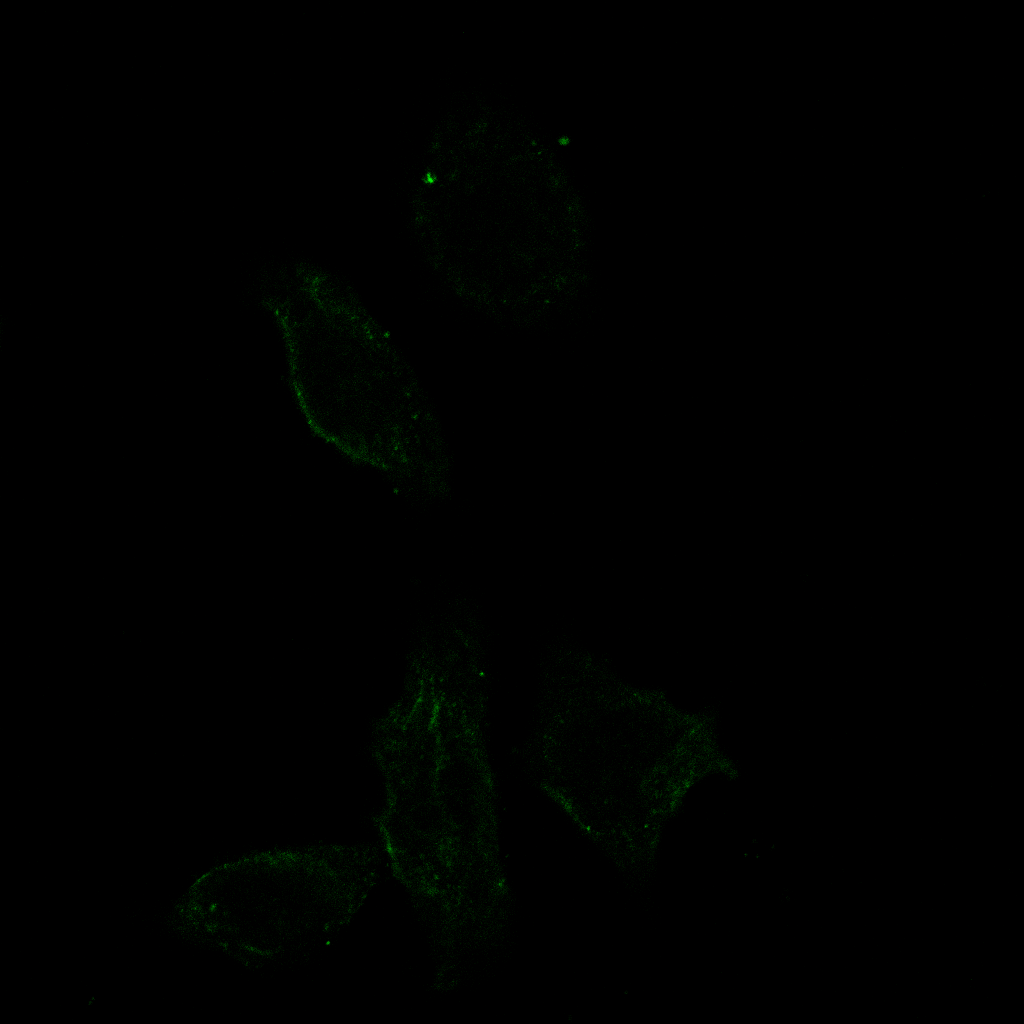

Supplement: Supplementary file 2 — Additional file 2: The raw experimental data related to this study. [file 12935_2022_2689_MOESM2_ESM.zip › Confocal/Export_87-AKT1+PAK1-2_RGB_FITC.tif]

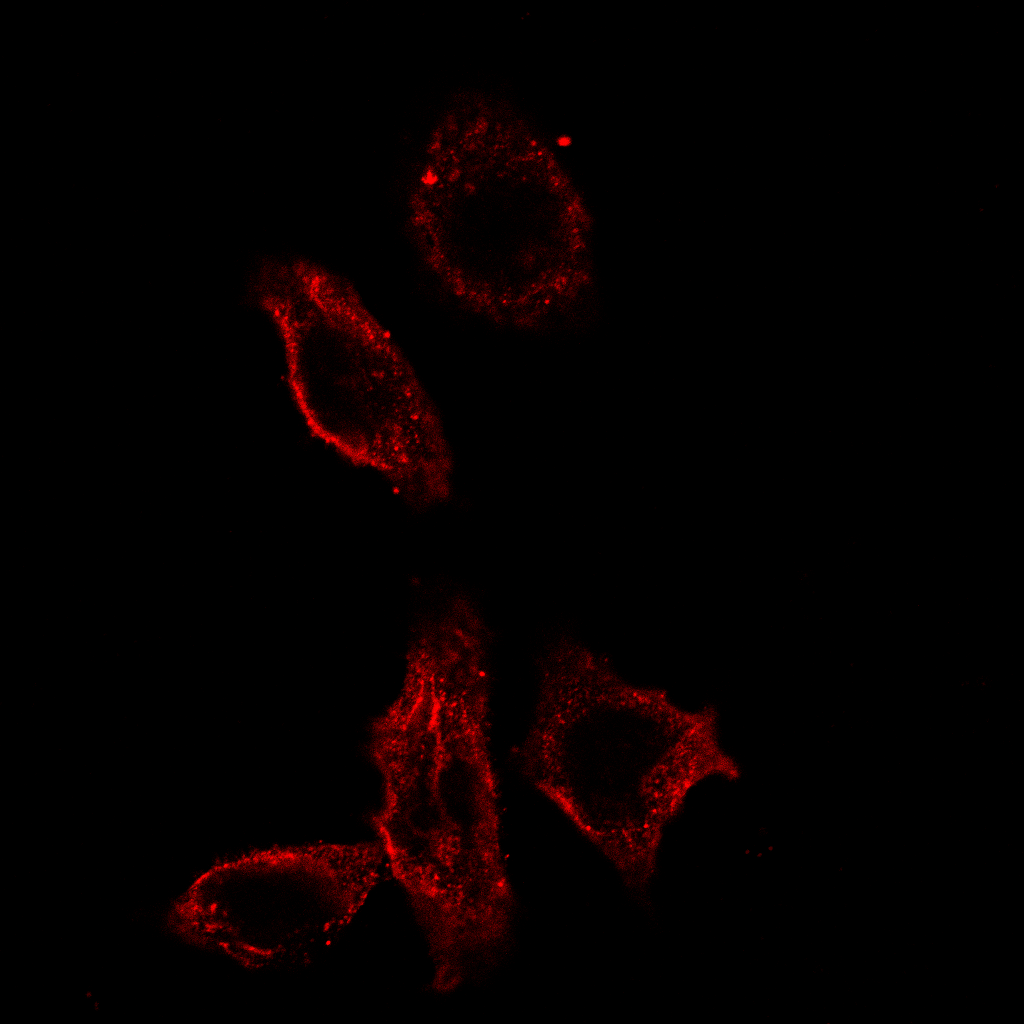

Supplement: Supplementary file 2 — Additional file 2: The raw experimental data related to this study. [file 12935_2022_2689_MOESM2_ESM.zip › Confocal/Export_87-AKT1+PAK1-2_RGB_TRITC.tif]

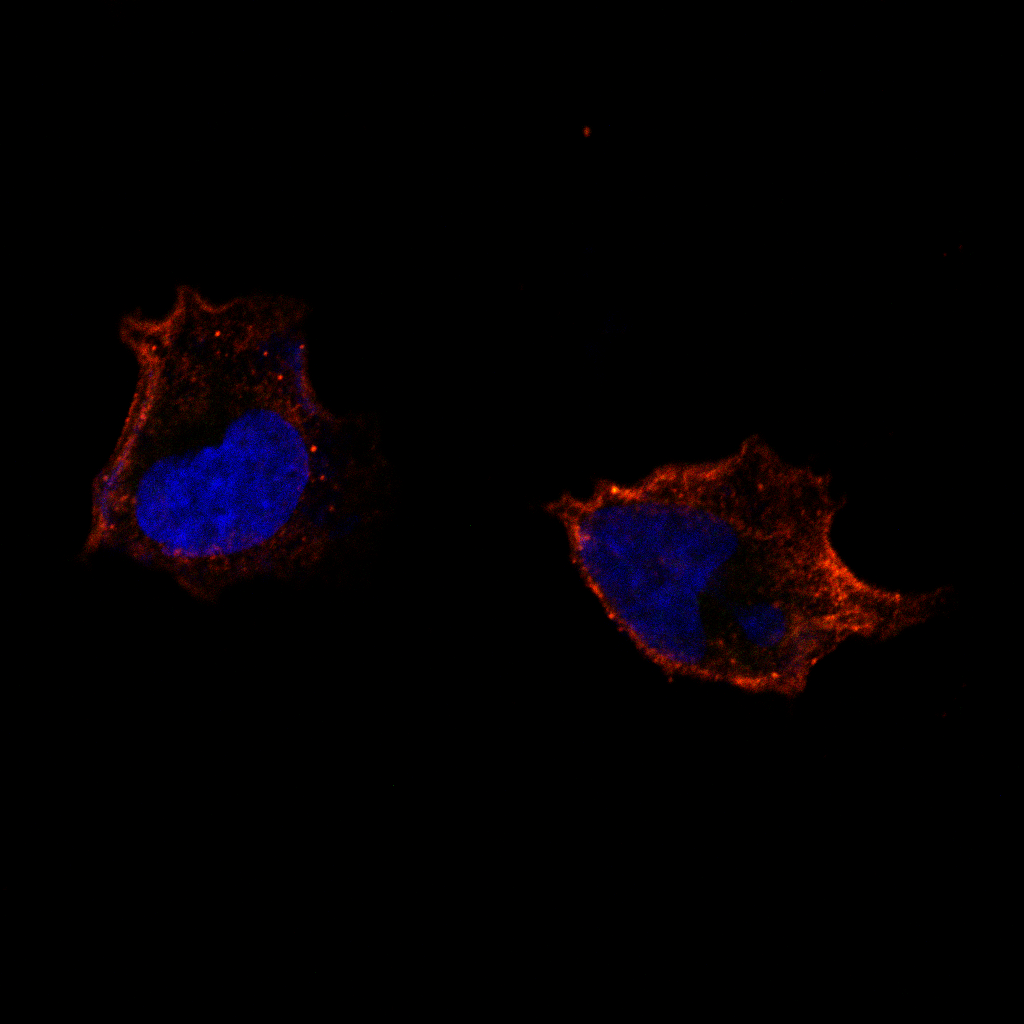

Supplement: Supplementary file 2 — Additional file 2: The raw experimental data related to this study. [file 12935_2022_2689_MOESM2_ESM.zip › Confocal/Export_87-AKT1+PAK1-3_RGB.tif]

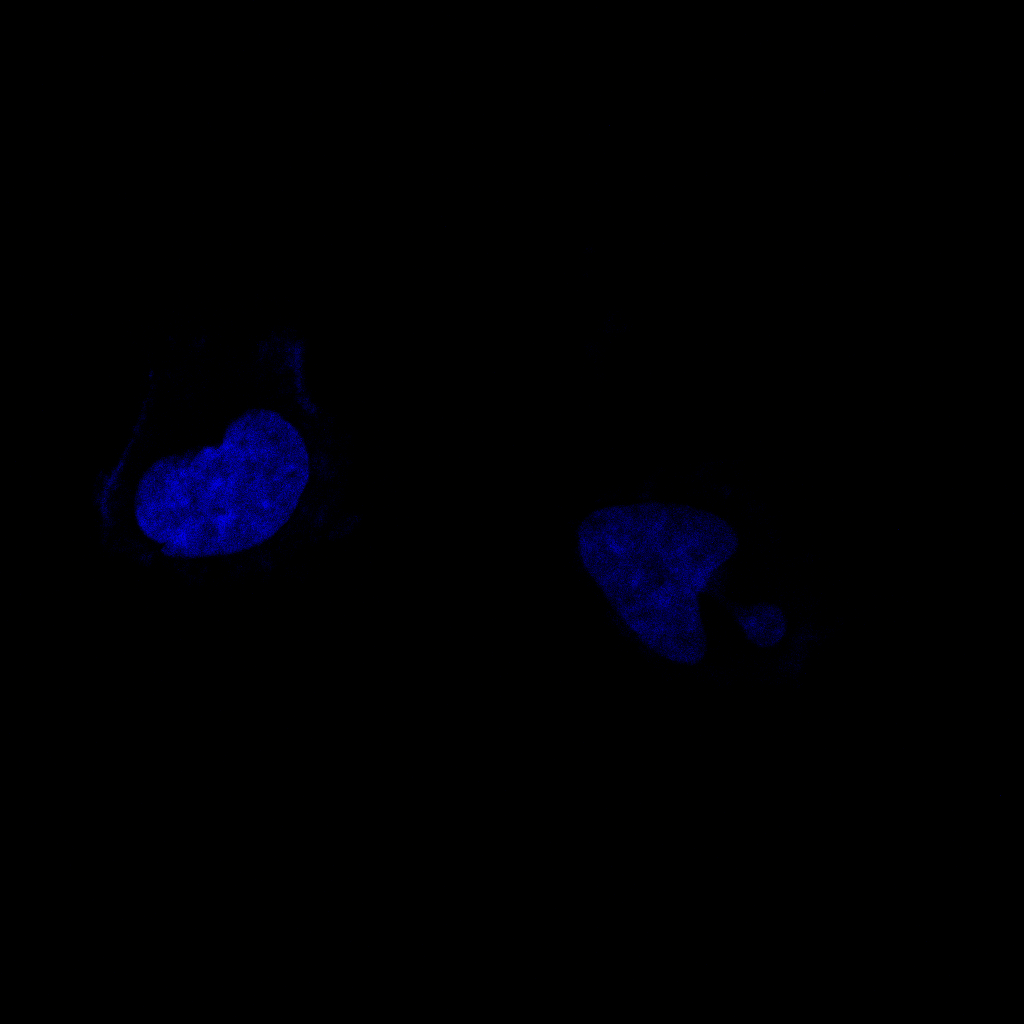

Supplement: Supplementary file 2 — Additional file 2: The raw experimental data related to this study. [file 12935_2022_2689_MOESM2_ESM.zip › Confocal/Export_87-AKT1+PAK1-3_RGB_DAPI.tif]

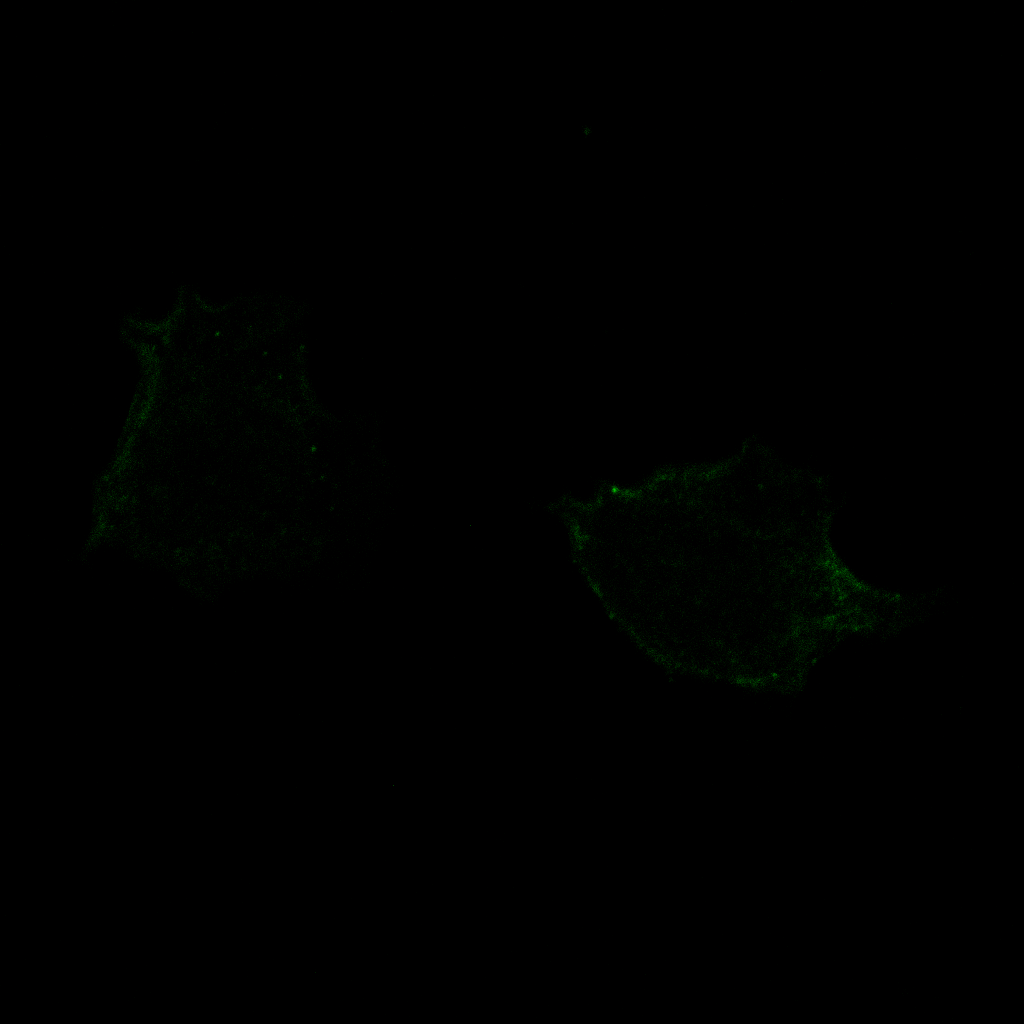

Supplement: Supplementary file 2 — Additional file 2: The raw experimental data related to this study. [file 12935_2022_2689_MOESM2_ESM.zip › Confocal/Export_87-AKT1+PAK1-3_RGB_FITC.tif]

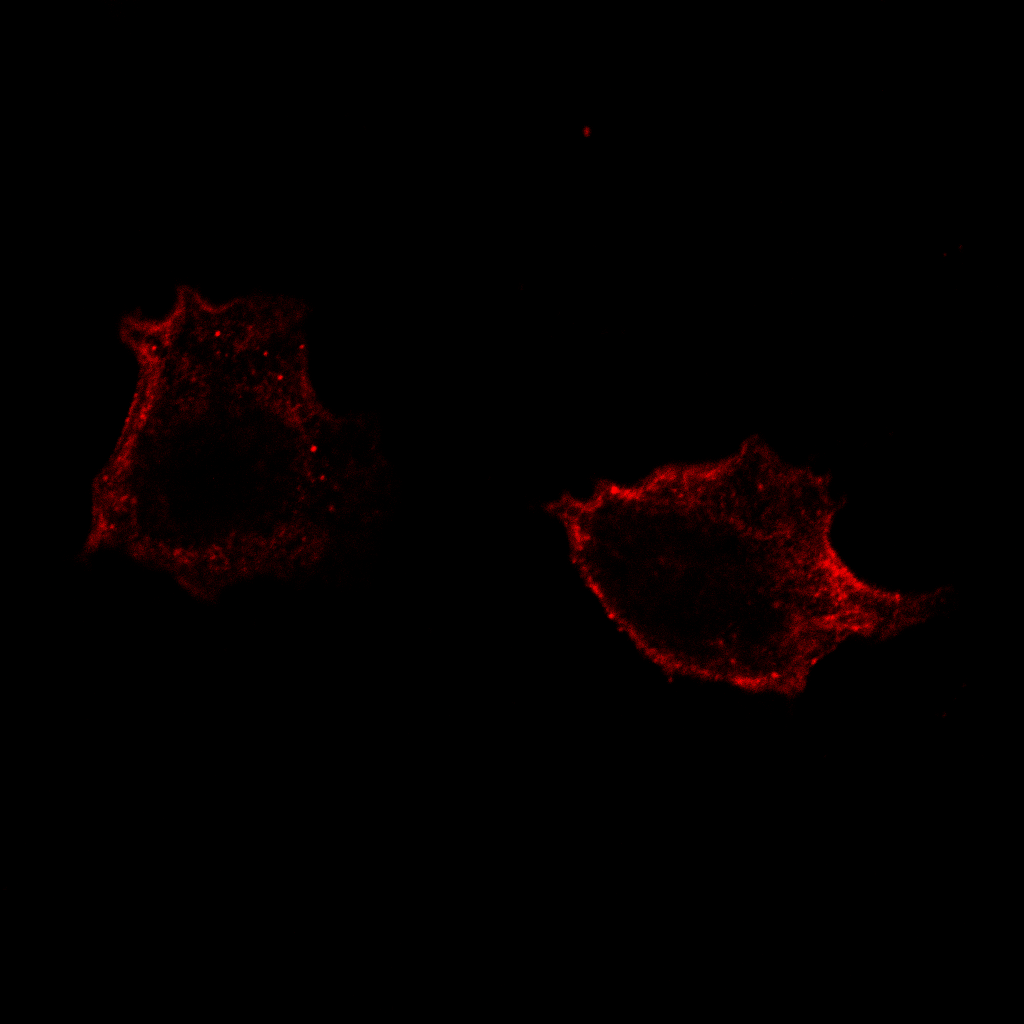

Supplement: Supplementary file 2 — Additional file 2: The raw experimental data related to this study. [file 12935_2022_2689_MOESM2_ESM.zip › Confocal/Export_87-AKT1+PAK1-3_RGB_TRITC.tif]

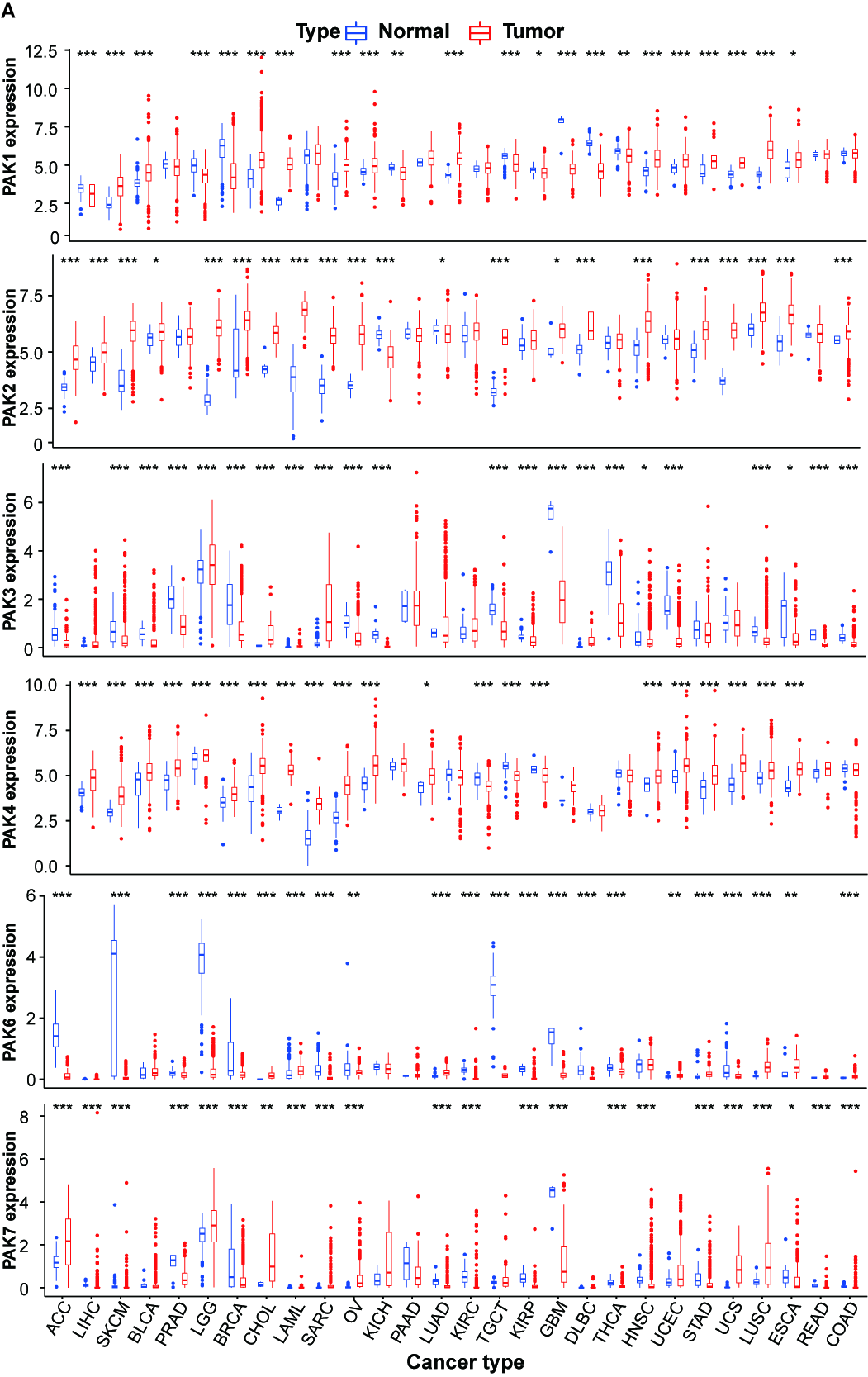

Supplement: Supplementary file 3 — Additional file 3: Figure S1. Differences in gene expression of PAKs in 28 tumor tissues and corresponding tissues. [file 12935_2022_2689_MOESM3_ESM.tif]

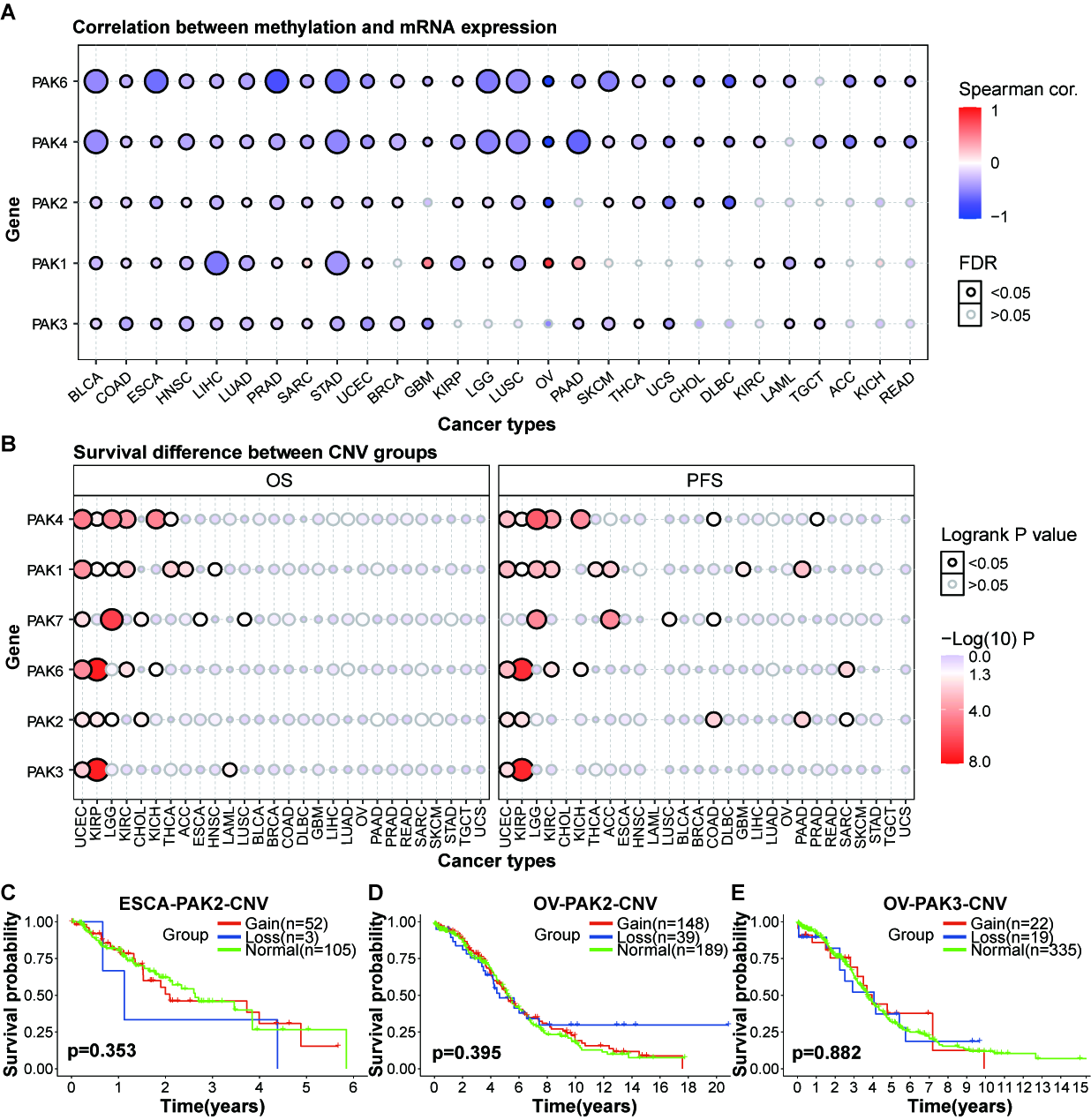

Supplement: Supplementary file 4 — Additional file 4: Figure S2. (A) Correlation between mRNA expression and methylation of PAKs. Survival difference between different CNV groups (B), survival analysis showed that there was no significant difference(P<0.05) in survival between ESCA and OV patients with higher CNV levels of some PAKs. The size of the point represents the absolute value of the correlation. [file 12935_2022_2689_MOESM4_ESM.tif]

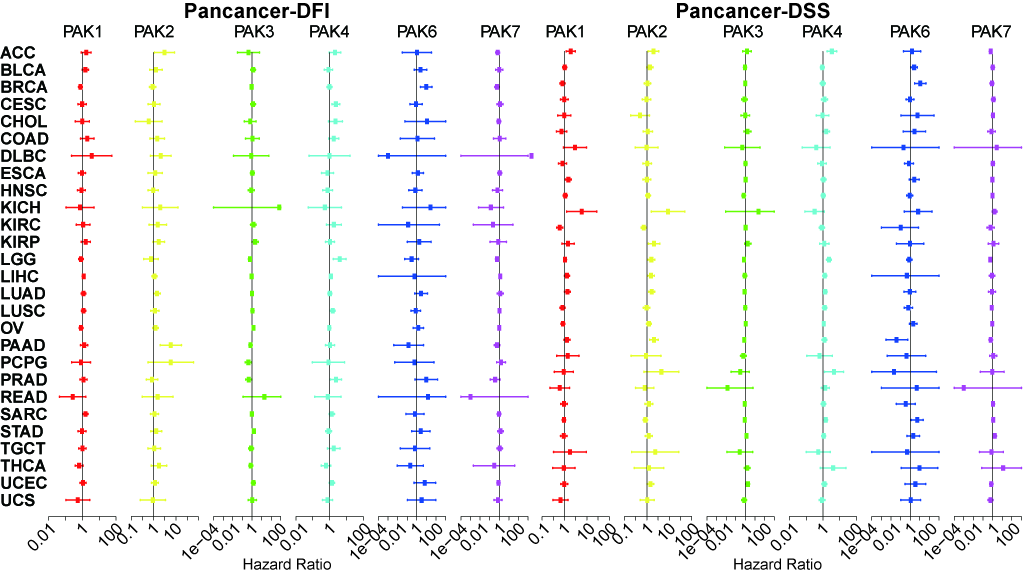

Supplement: Supplementary file 5 — Additional file 5: Figure S3. The forest plot of Progression Free Interval and Disease-Specific Survival in pan-cancer patients shows the survival advantages and disadvantages of increased PAK gene expression. [file 12935_2022_2689_MOESM5_ESM.tif]

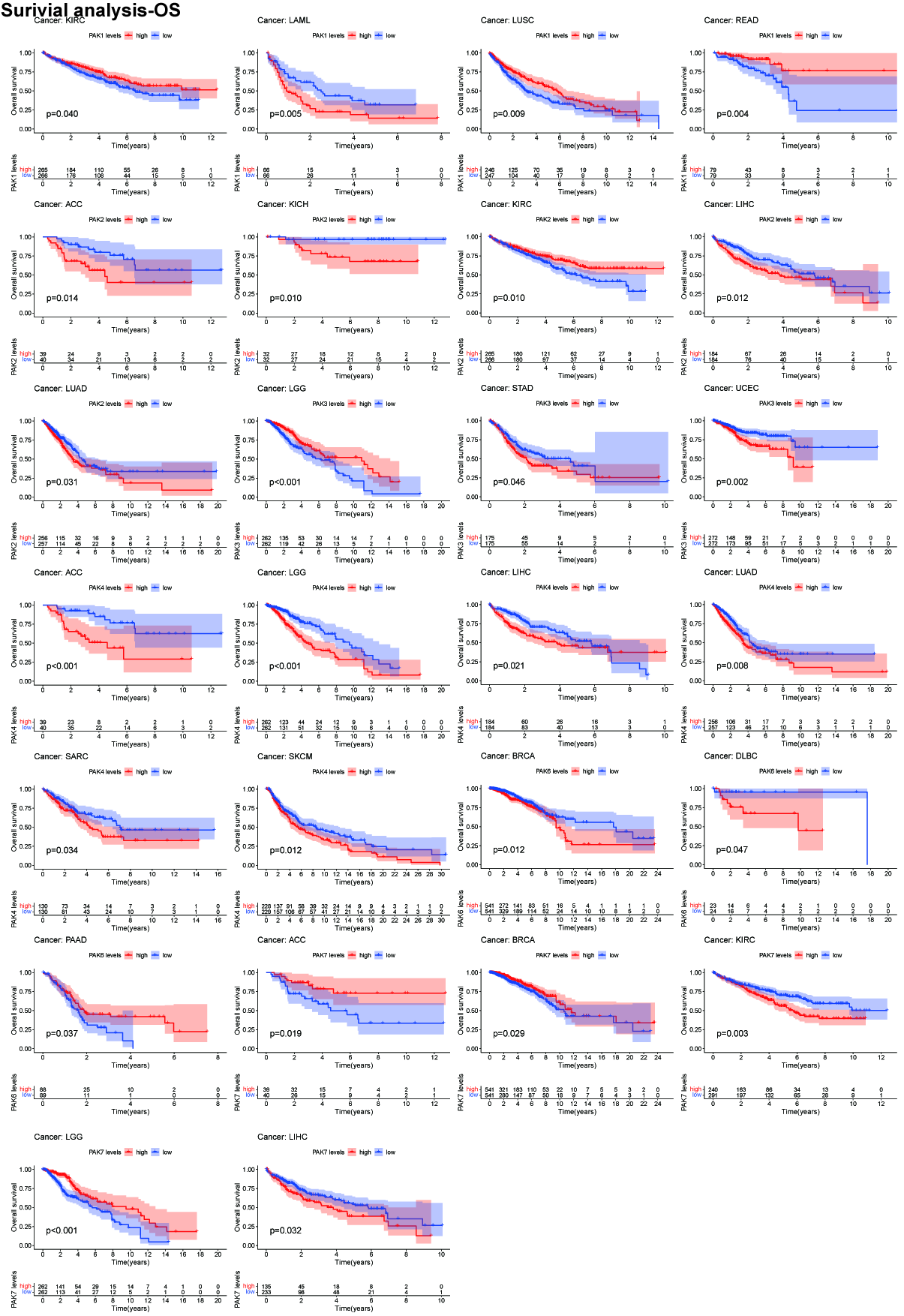

Supplement: Supplementary file 6 — Additional file 6: Figure S4. Kaplan–Meier (KM) survival curves revealed that the high- and low-expression group of PAKs had a significant difference in the overall survival and progression free interval. [file 12935_2022_2689_MOESM6_ESM.tif]

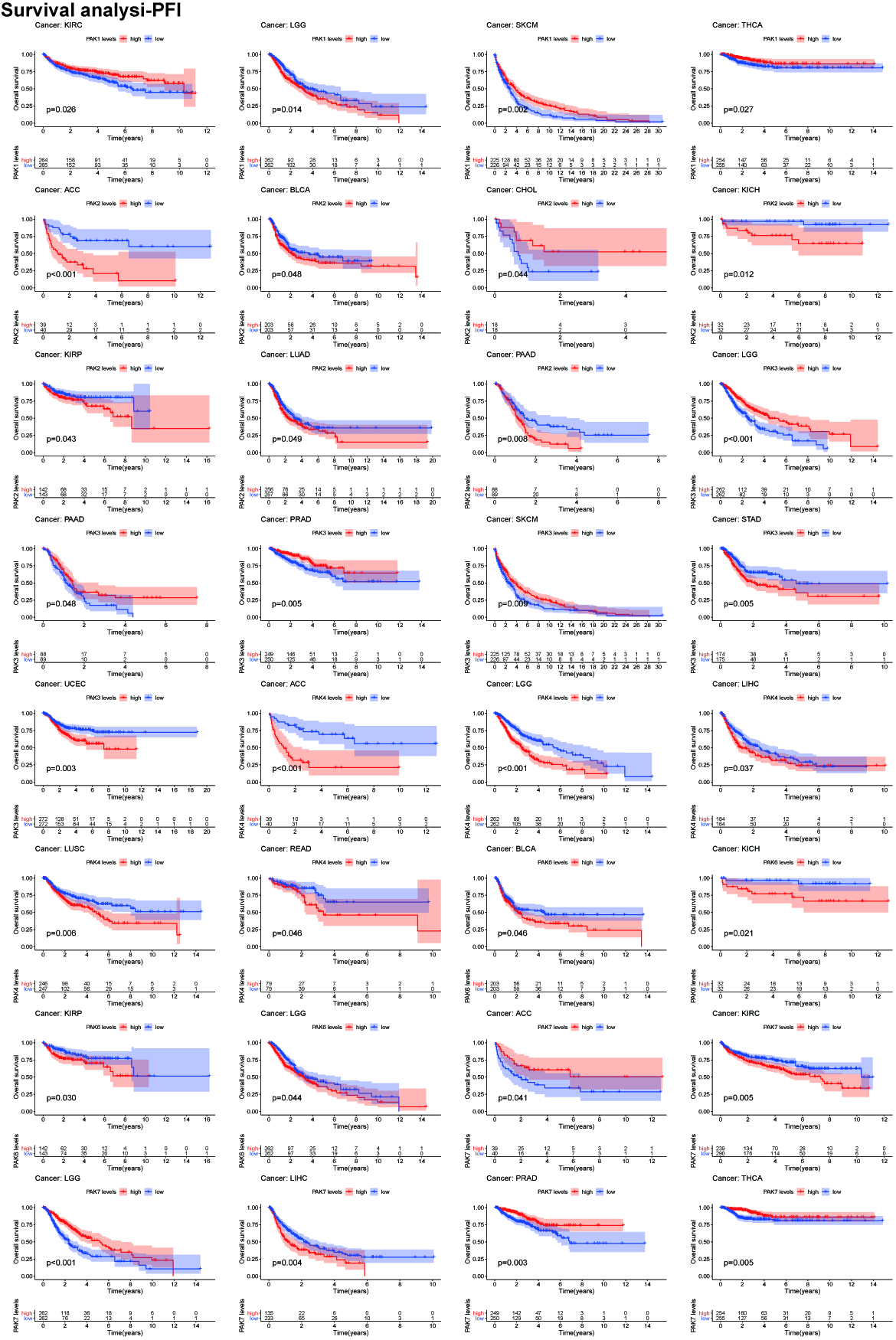

Supplement: Supplementary file 7 — Additional file 7: Figure S5. Kaplan–Meier (KM) survival curves revealed that the high- and low-expression group of PAKs had a significant difference in the overall survival and progression free interval. [file 12935_2022_2689_MOESM7_ESM.tif]

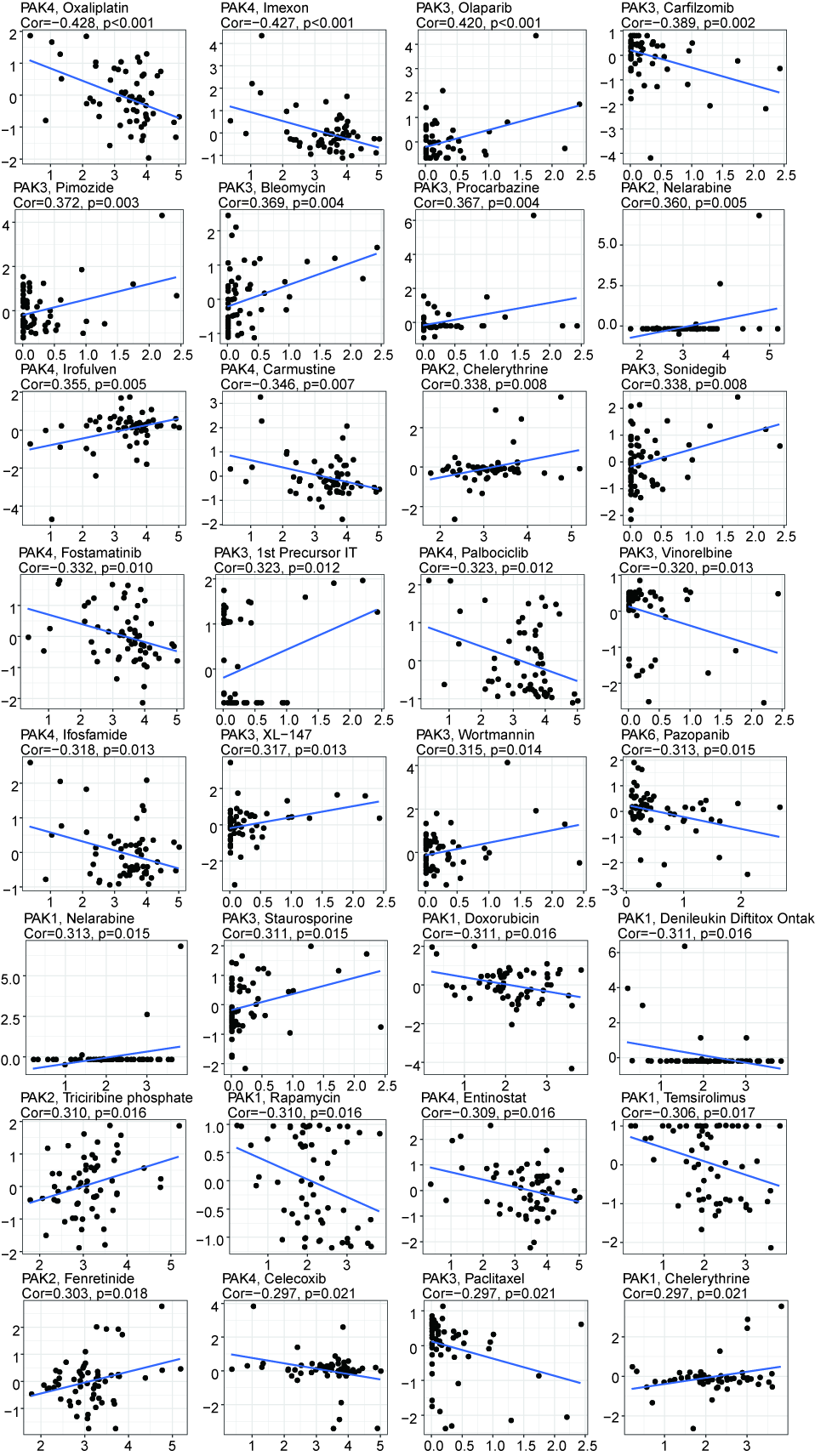

Supplement: Supplementary file 8 — Additional file 8: Figure S6. Association between PAKs gene expression and drug sensitivity (Z-score from the CellMiner interface) using NCI-60 cell line data. [file 12935_2022_2689_MOESM8_ESM.tif]

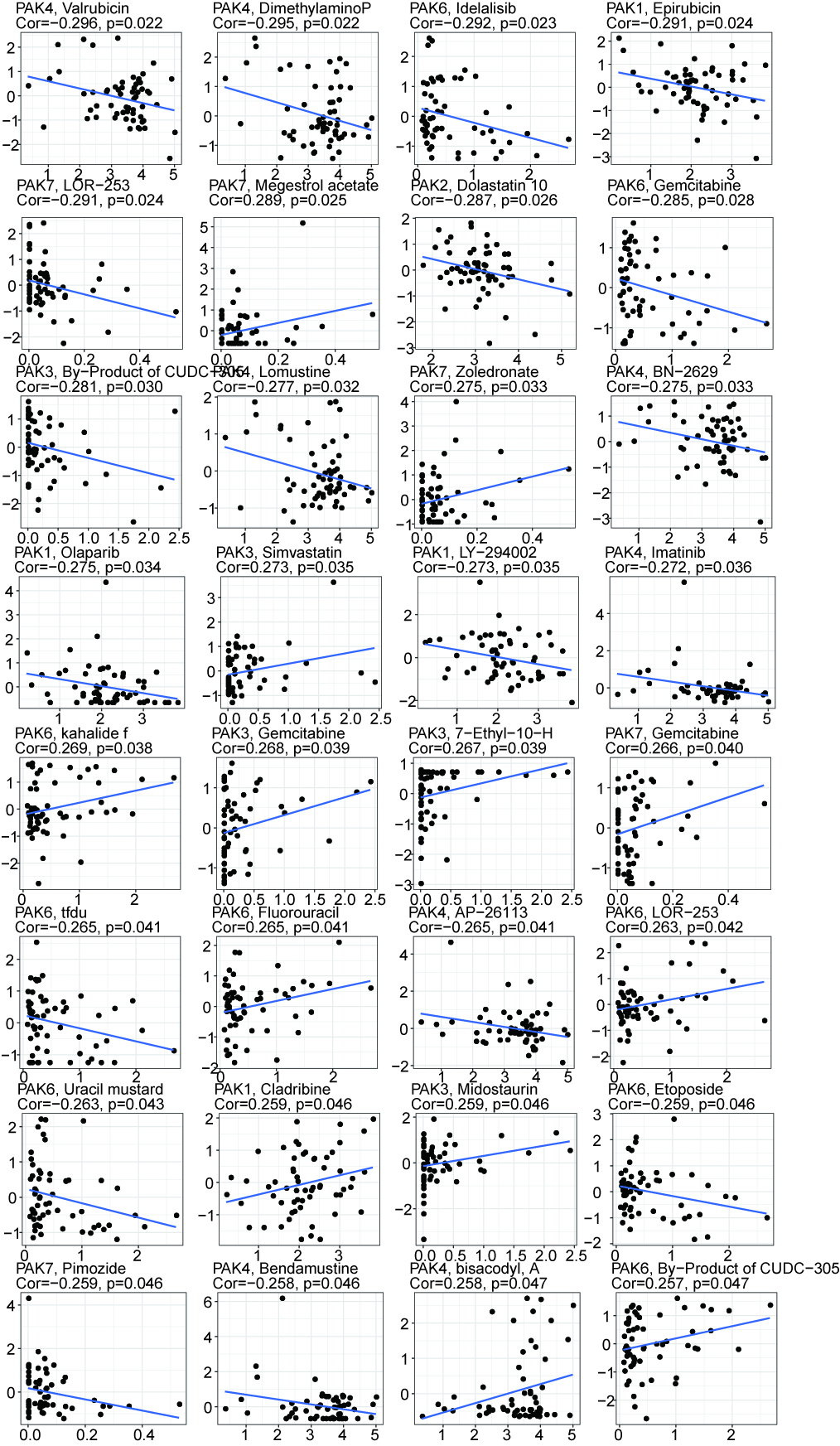

Supplement: Supplementary file 9 — Additional file 9: Figure S7. Association between PAKs gene expression and drug sensitivity (Z-score from the CellMiner interface) using NCI-60 cell line data. [file 12935_2022_2689_MOESM9_ESM.tif]

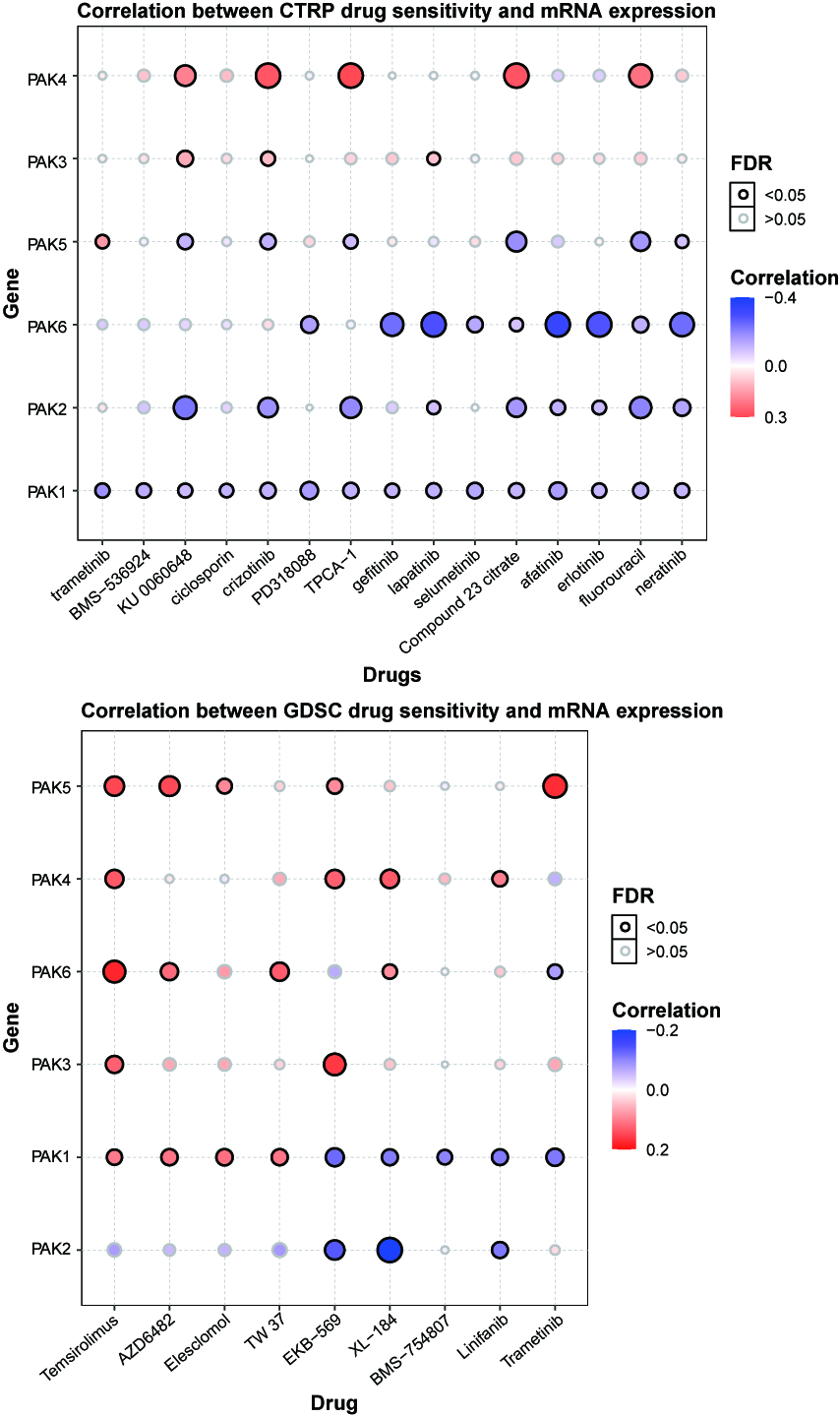

Supplement: Supplementary file 10 — Additional file 10: Figure S8. Correlation between CTRP and GDSC drug sensitivity and mRNA expression of PAKs. [file 12935_2022_2689_MOESM10_ESM.tif]

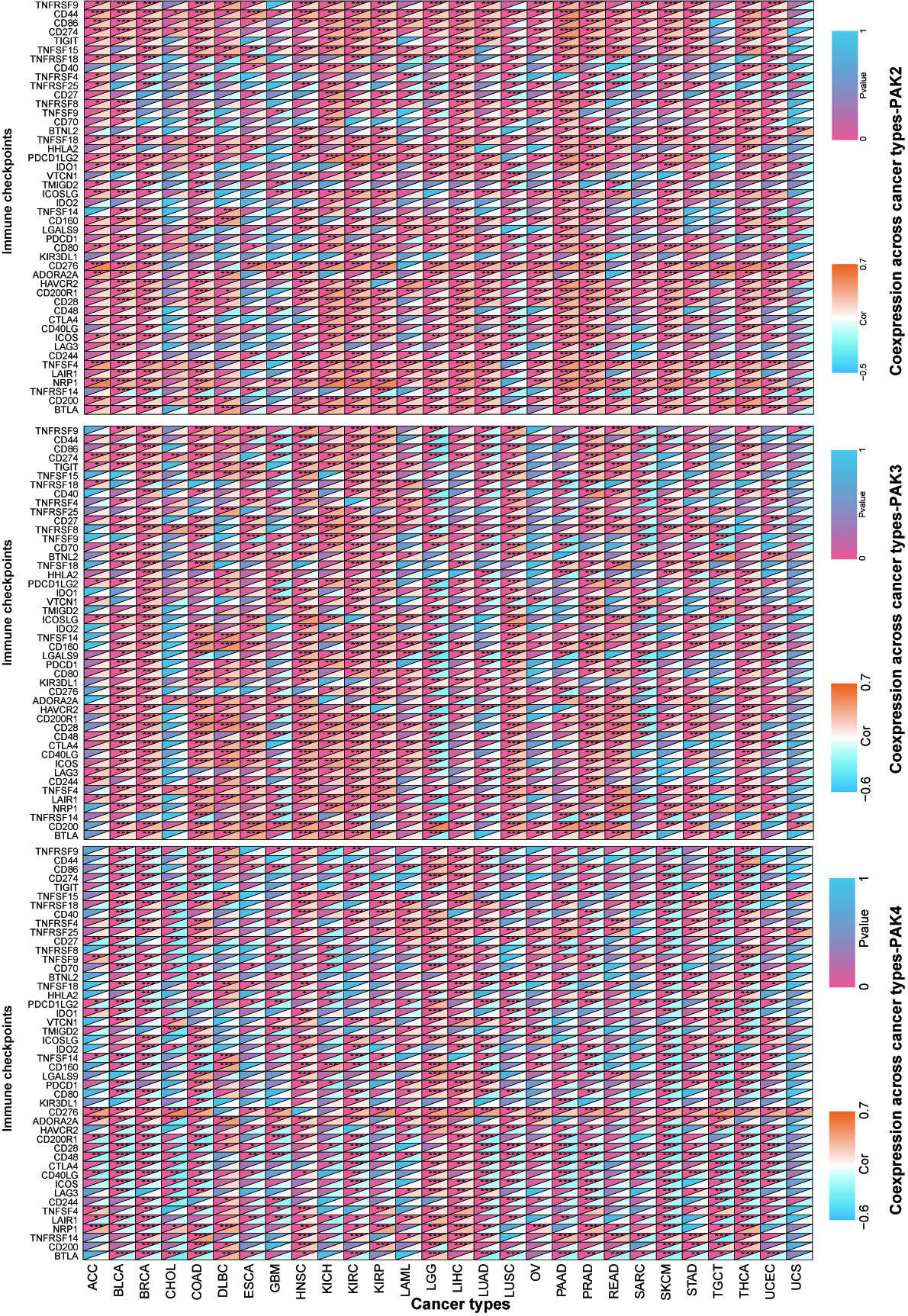

Supplement: Supplementary file 11 — Additional file 11: Figure S9. Correlation between PAK2, PAK3, PAK4, PAK6, and PAK7 gene expression level and 46 immune checkpoints in pan-cancers. [file 12935_2022_2689_MOESM11_ESM.tif]

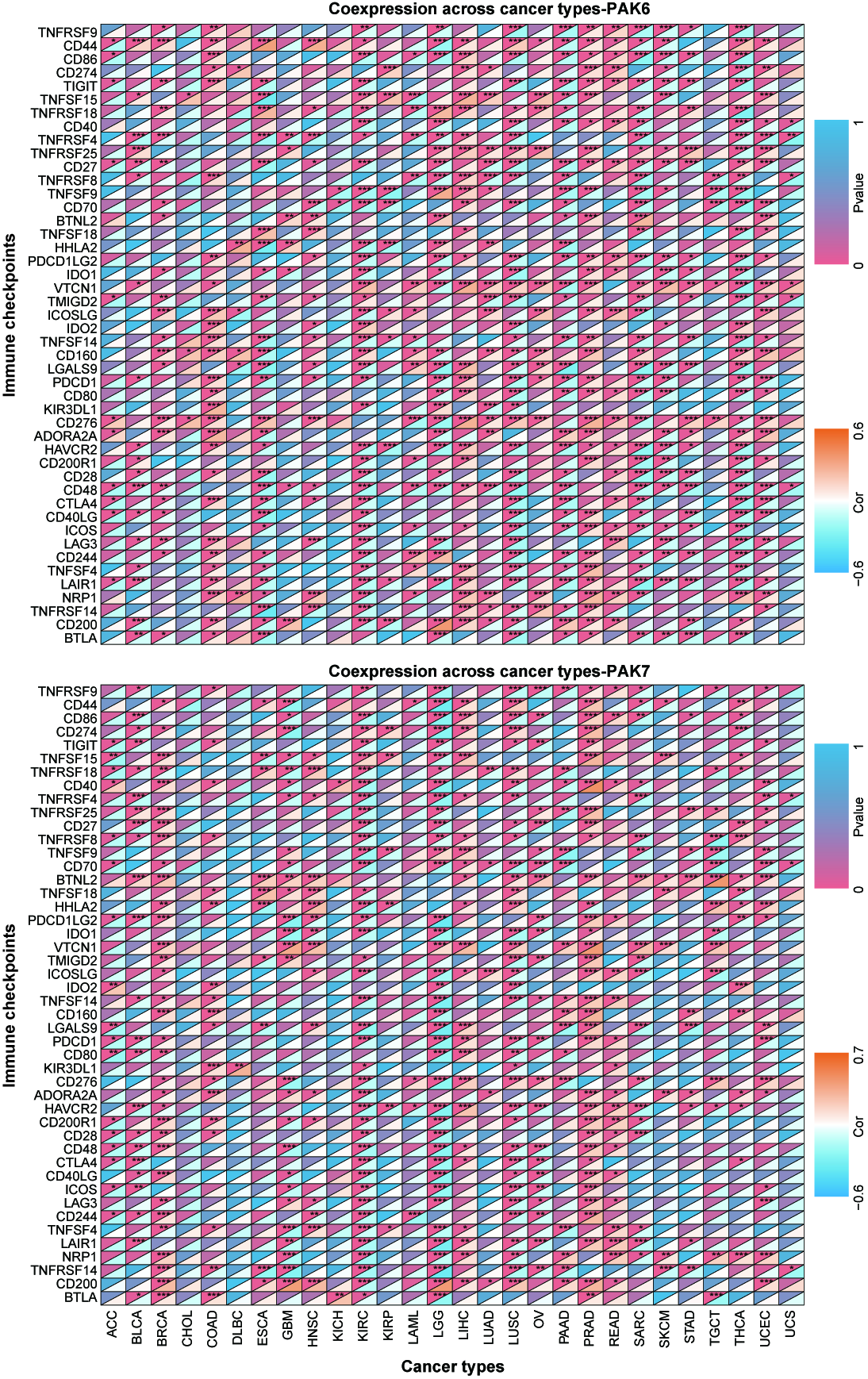

Supplement: Supplementary file 12 — Additional file 12: Figure S10. Correlation between PAK2, PAK3, PAK4, PAK6, and PAK7 gene expression level and 46 immune checkpoints in pan-cancers. [file 12935_2022_2689_MOESM12_ESM.tif]

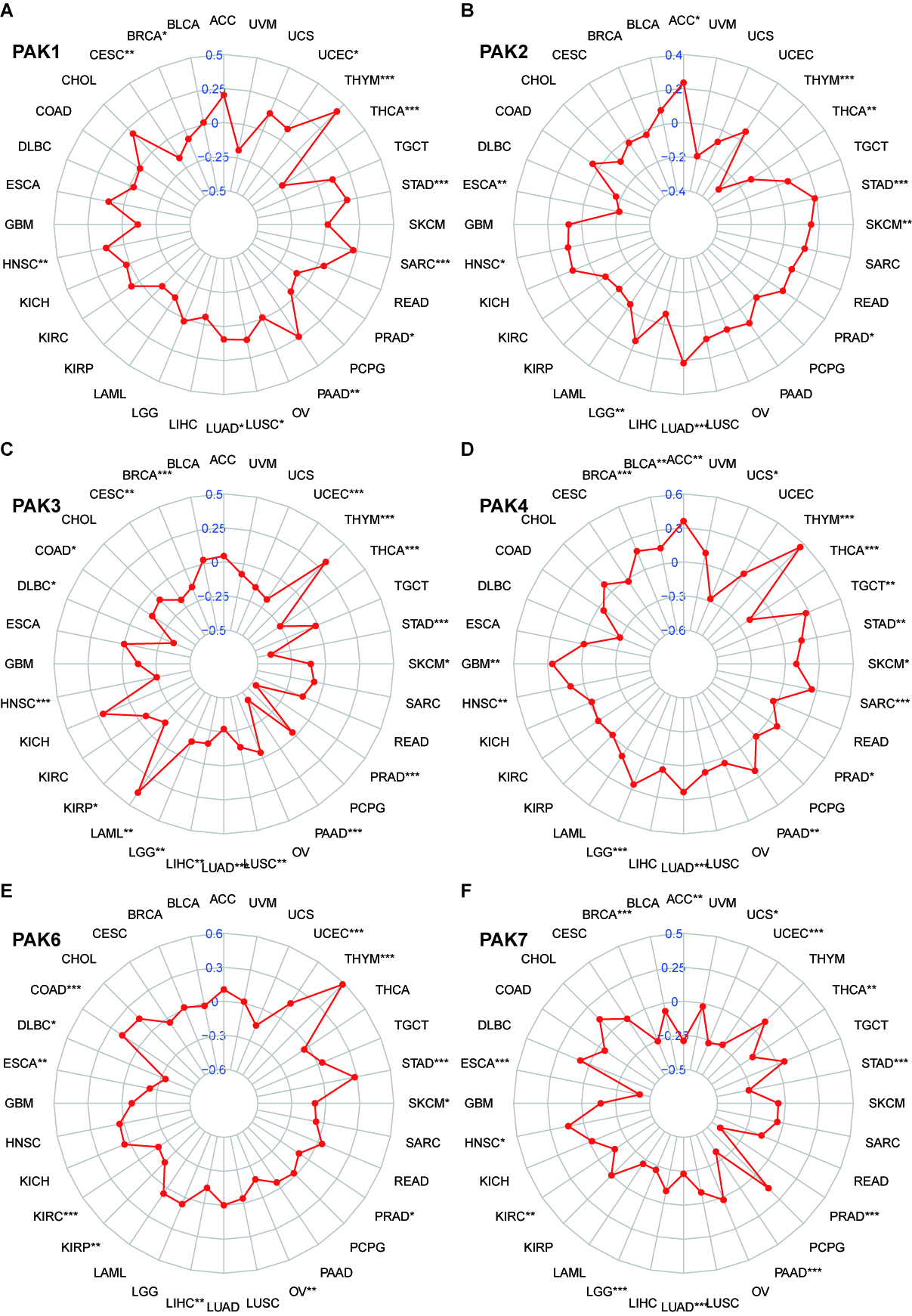

Supplement: Supplementary file 13 — Additional file 13: Figure S11. Correlation of PAKs gene expression with tumor mutation burden (TMB) in different tumor types. [file 12935_2022_2689_MOESM13_ESM.tif]

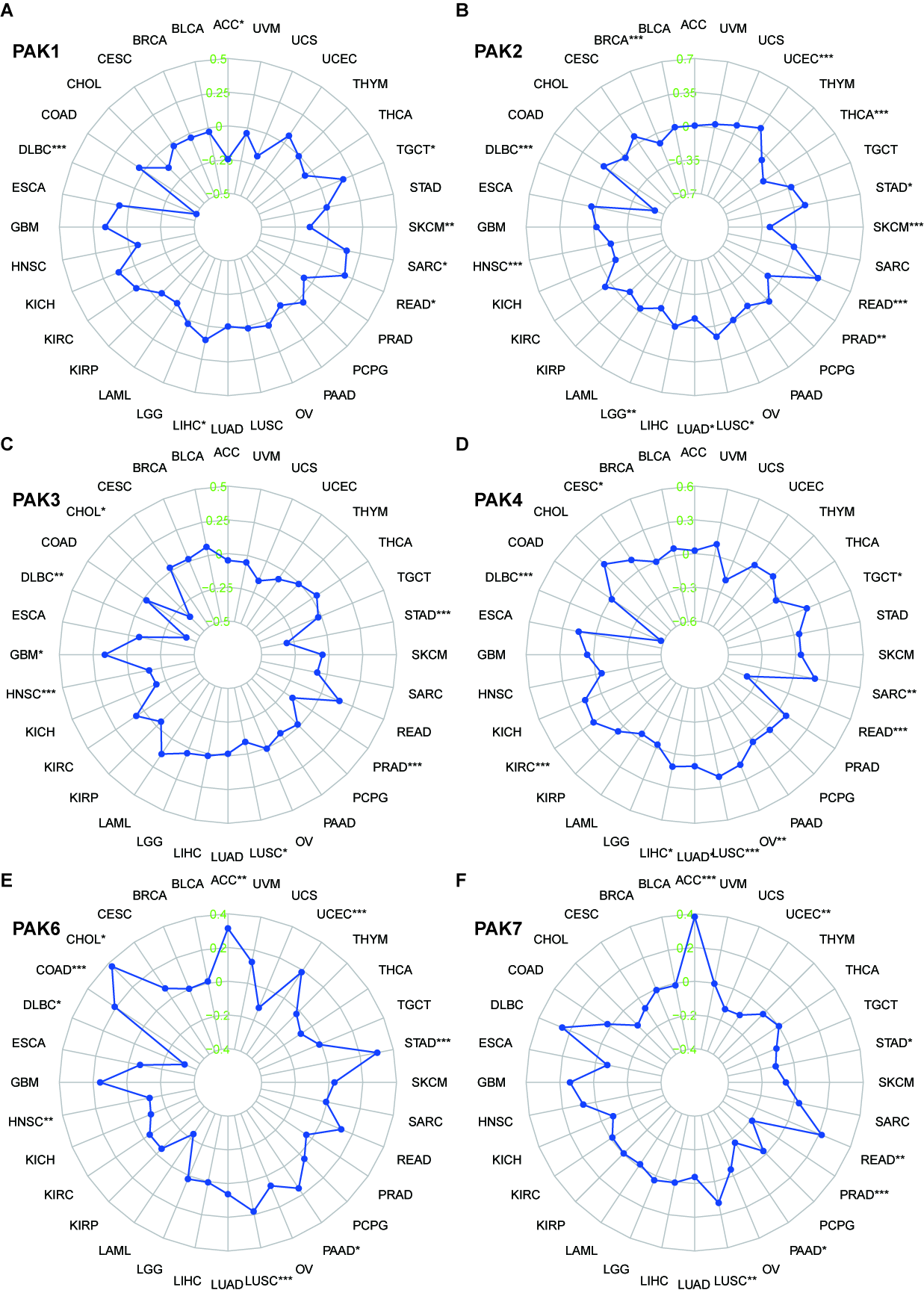

Supplement: Supplementary file 14 — Additional file 14: Figure S12. Correlation of PAKs gene expression with microsatellite instability (MSI) in different tumor types. [file 12935_2022_2689_MOESM14_ESM.tif]

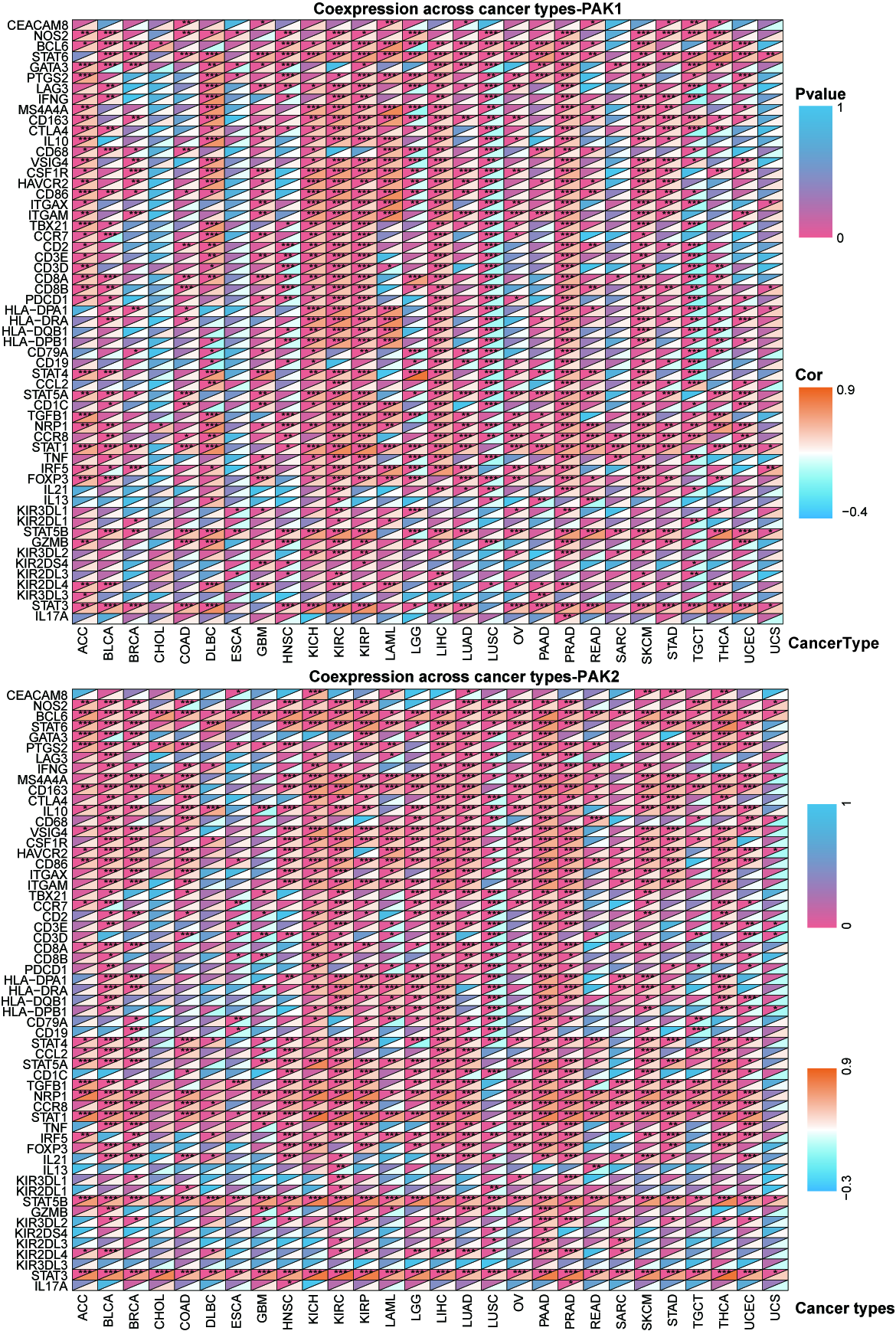

Supplement: Supplementary file 15 — Additional file15: Figure S13. Correlation between PAKs expression and several common immune cells and genetic markers of infiltrating immune cells. [file 12935_2022_2689_MOESM15_ESM.tif]

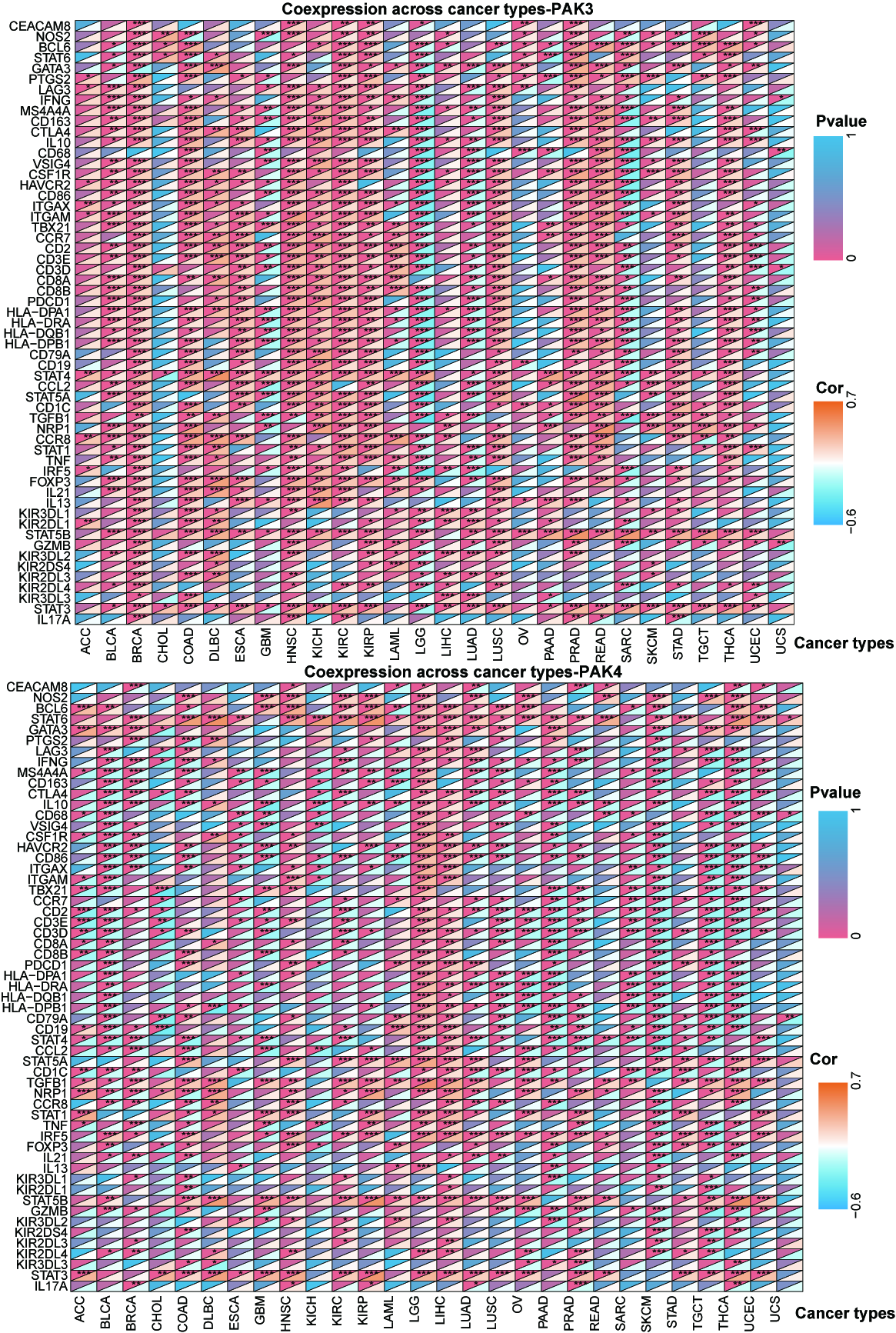

Supplement: Supplementary file 16 — Additional file 16: Figure S14. Correlation between PAKs expression and several common immune cells and genetic markers of infiltrating immune cells. [file 12935_2022_2689_MOESM16_ESM.tif]

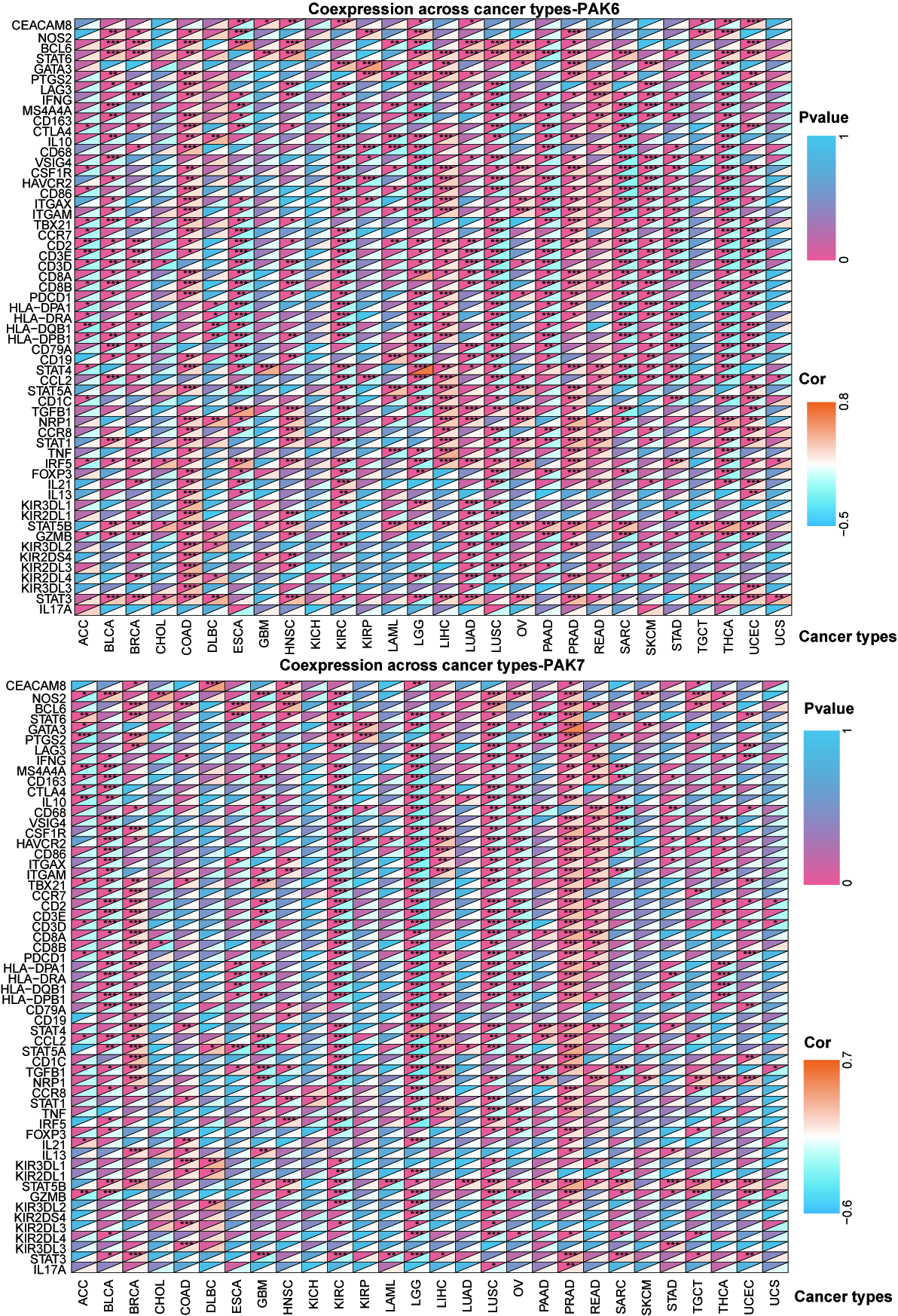

Supplement: Supplementary file 17 — Additional file 17: Figure S15. Correlation between PAKs expression and several common immune cells and genetic markers of infiltrating immune cells. [file 12935_2022_2689_MOESM17_ESM.tif]

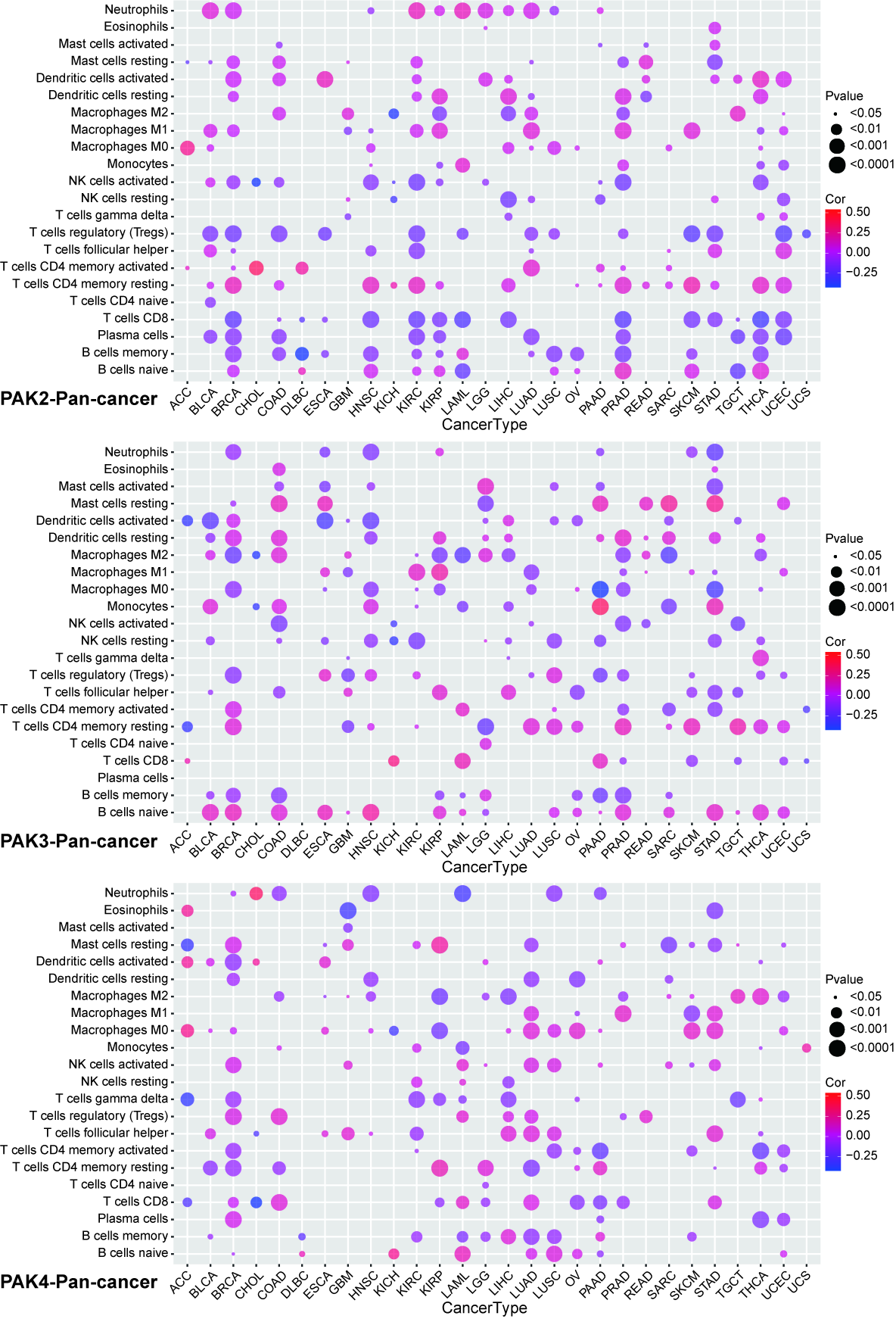

Supplement: Supplementary file 18 — Additional file 18: Figure S16. Correlation between PAKs gene expression level and different immune-related cell. [file 12935_2022_2689_MOESM18_ESM.tif]

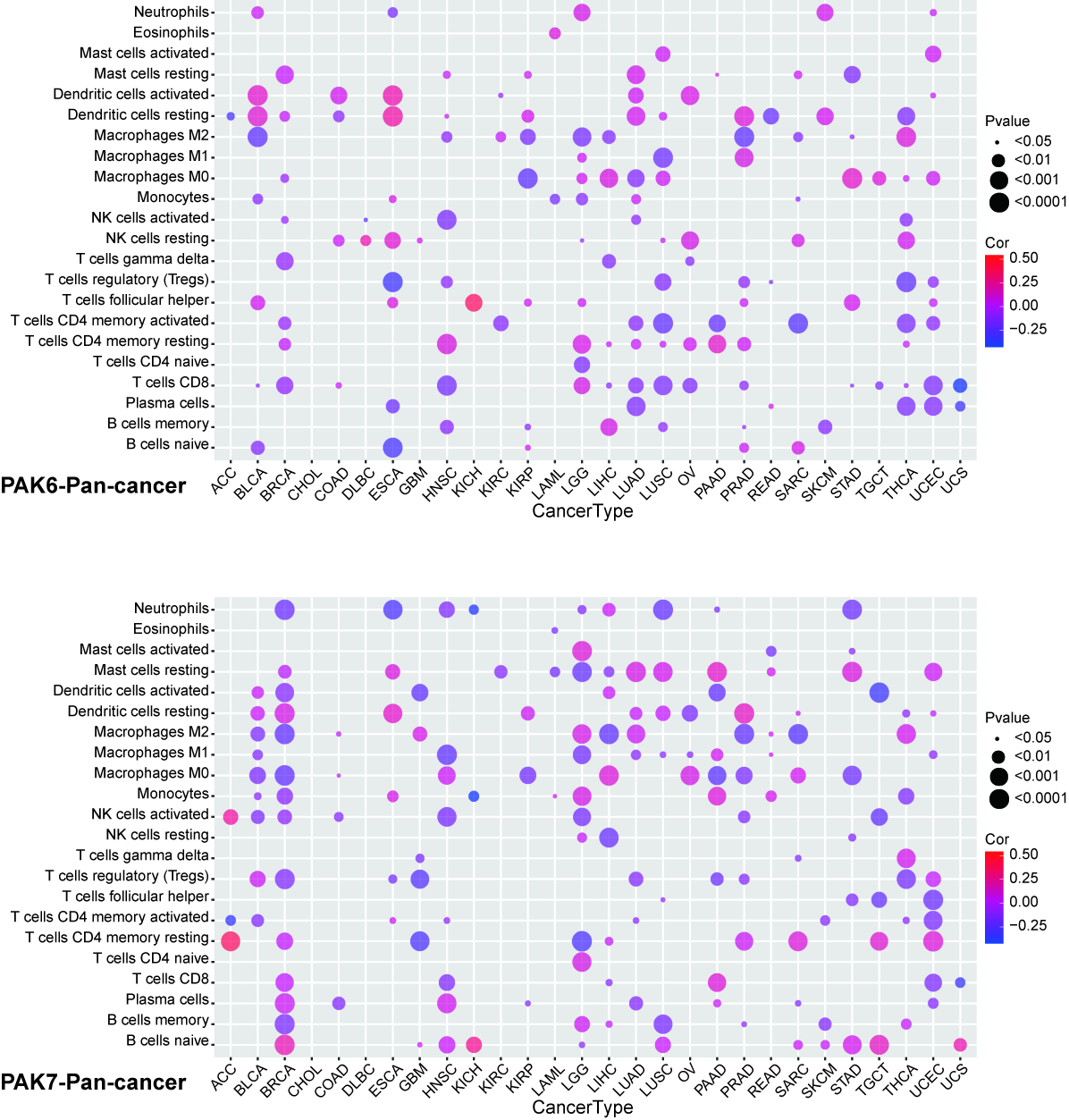

Supplement: Supplementary file 19 — Additional file 19: Figure S17. Correlation between PAKs gene expression level and different immune-related cell. [file 12935_2022_2689_MOESM19_ESM.tif]

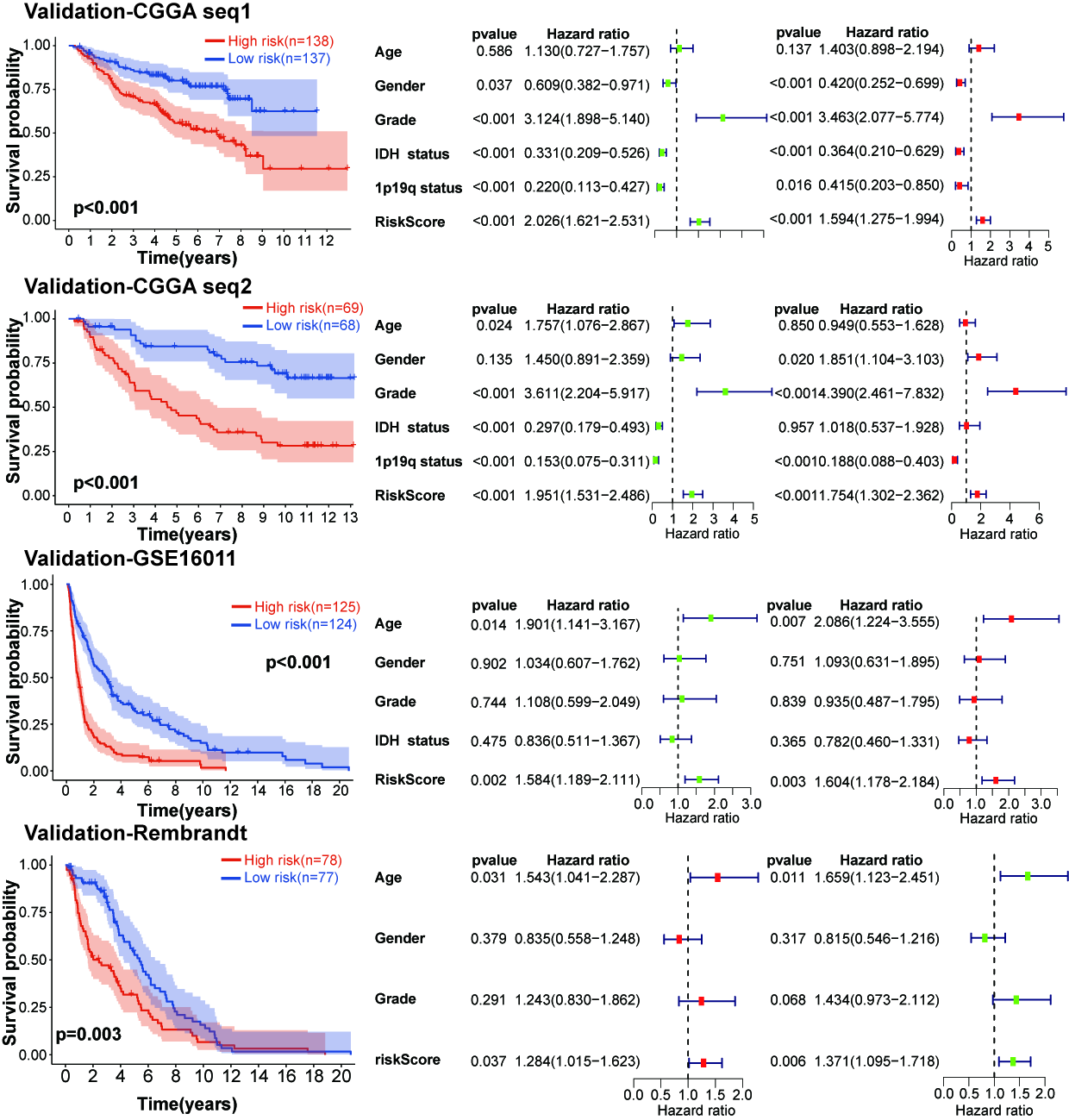

Supplement: Supplementary file 20 — Additional file 20: Figure S18. Verifying the prognostic stability of PAKs signature in CGGA seq1, CGGA seq2, GSE16011 and Rembrandt datasets. [file 12935_2022_2689_MOESM20_ESM.tif]

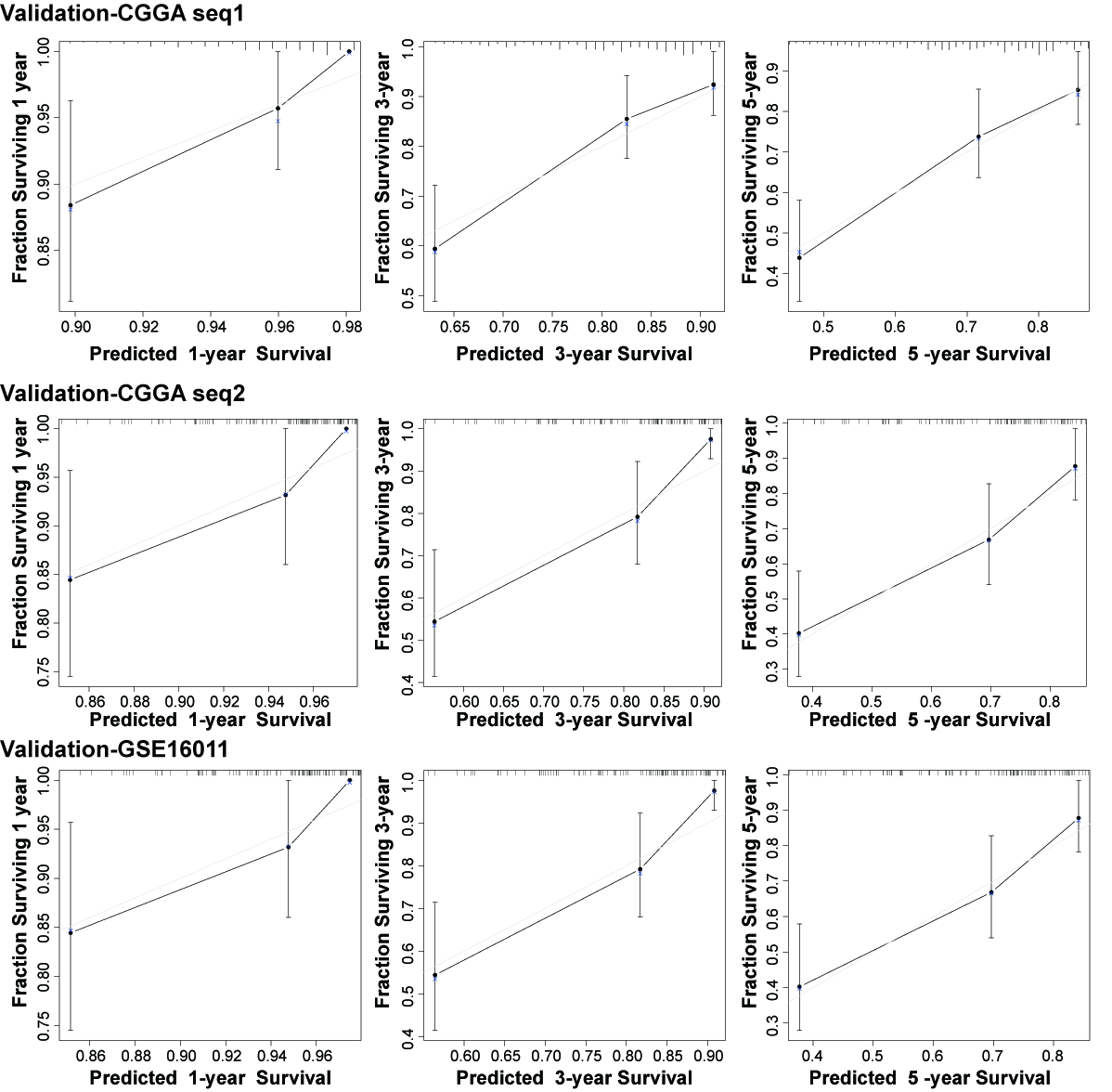

Supplement: Supplementary file 21 — Additional file 21: Figure S19. Nomogram calibration curves were used to predict 1-/3-/5-years survival rates in CGGA seq1, CGGA seq2, GSE16011 datasets. [file 12935_2022_2689_MOESM21_ESM.tif]

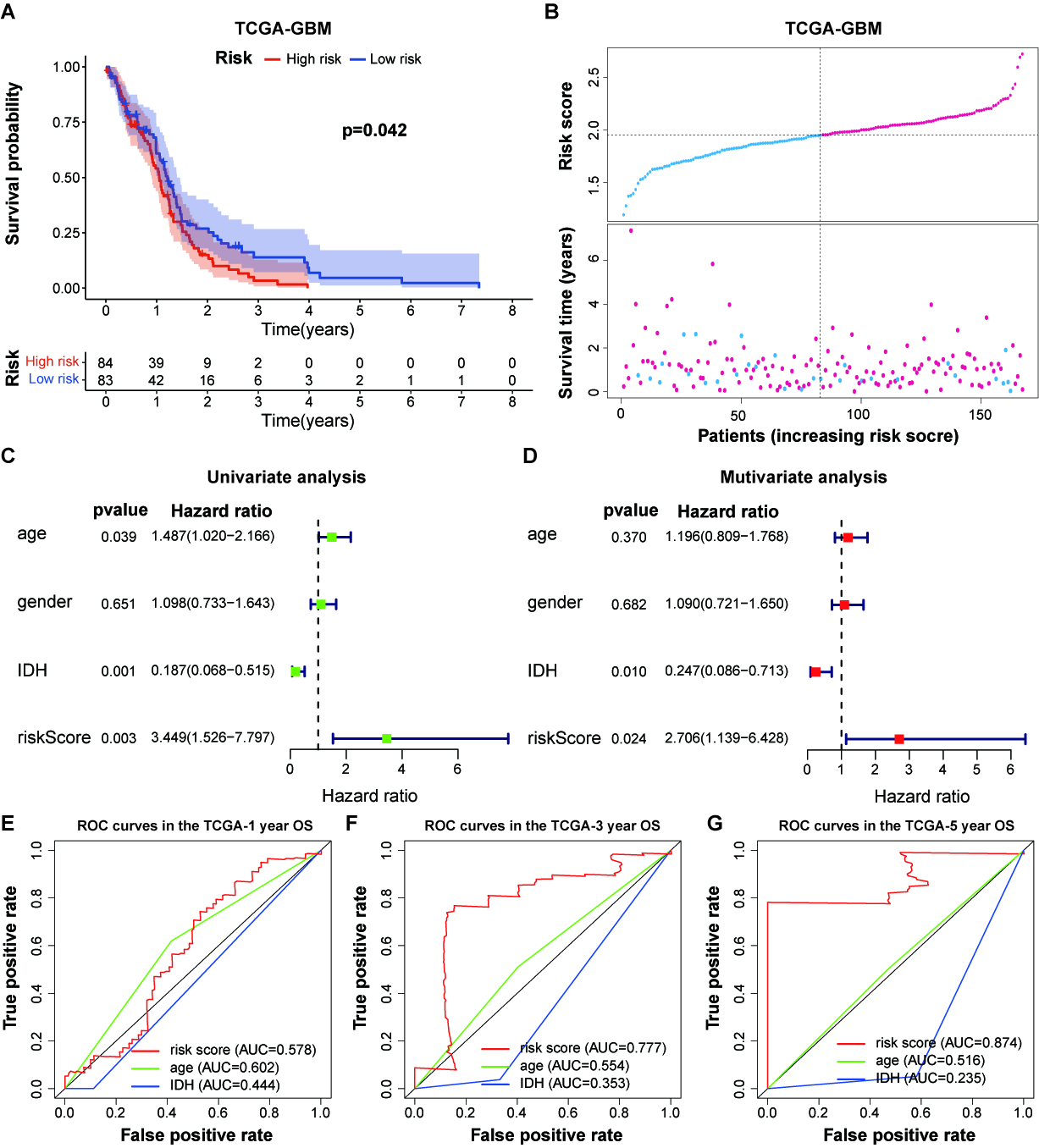

Supplement: Supplementary file 22 — Additional file 22: Figure S20. (A) Kaplan–Meier(K-M) curves of OS of high- and low- subgroups divided by the risk score of GBM patients in the TCGA dataset. (B) The distribution of GBM patients’ survival status, risk scores in the TCGA dataset. (C-D) Univariate and multivariate Cox analyses of different variables, including age, gender, IDH mutational status, and risk scores, in TCGA dataset. (E-G) Receiver operating characteristic (ROC) curves for the PAK1 expression for the prediction of 1/3/5-year survival of GBM patients in the TGGA dataset. [file 12935_2022_2689_MOESM22_ESM.tif]

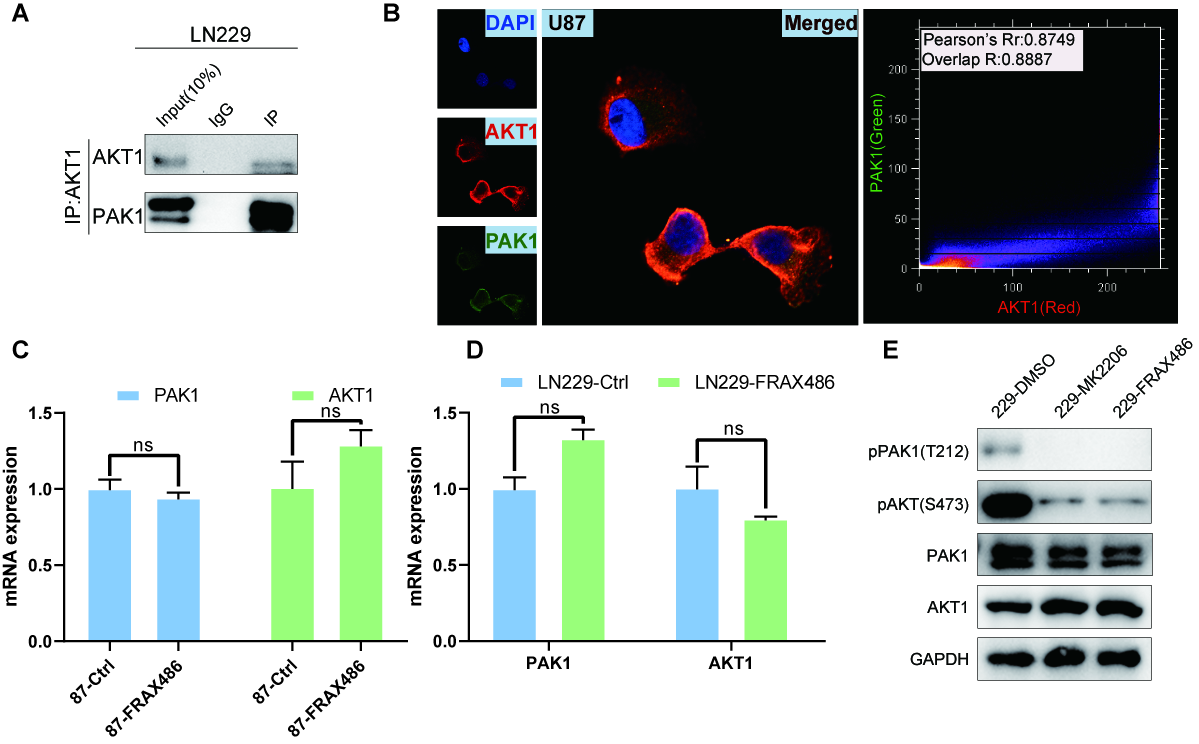

Supplement: Supplementary file 23 — Additional file 23: Figure S21. (A) Immunoprecipitation analysis was performed to detect the mutual binding between AKT1 and PAK1 in LN229 cells. (B) Cellular immunofluorescence shows the co-localization of AKT1 and PAK1 in U87 cells. (C-D) Application of qPCR in U87 and LN229 cells to detect the levels of PAK1 and AKT1 after FRAX486 treatment, standardized by GAPDH mRNA expression. (E) Western blot analysis was performed for detecting the protein lysates of PAK1, p-PAK1(T212), AKT1, p-AKT(S473) and GAPDH of LN229 cells after being treated with 10μM MK2206 and 1μM FRAX486 for 24h. [file 12935_2022_2689_MOESM23_ESM.tif]

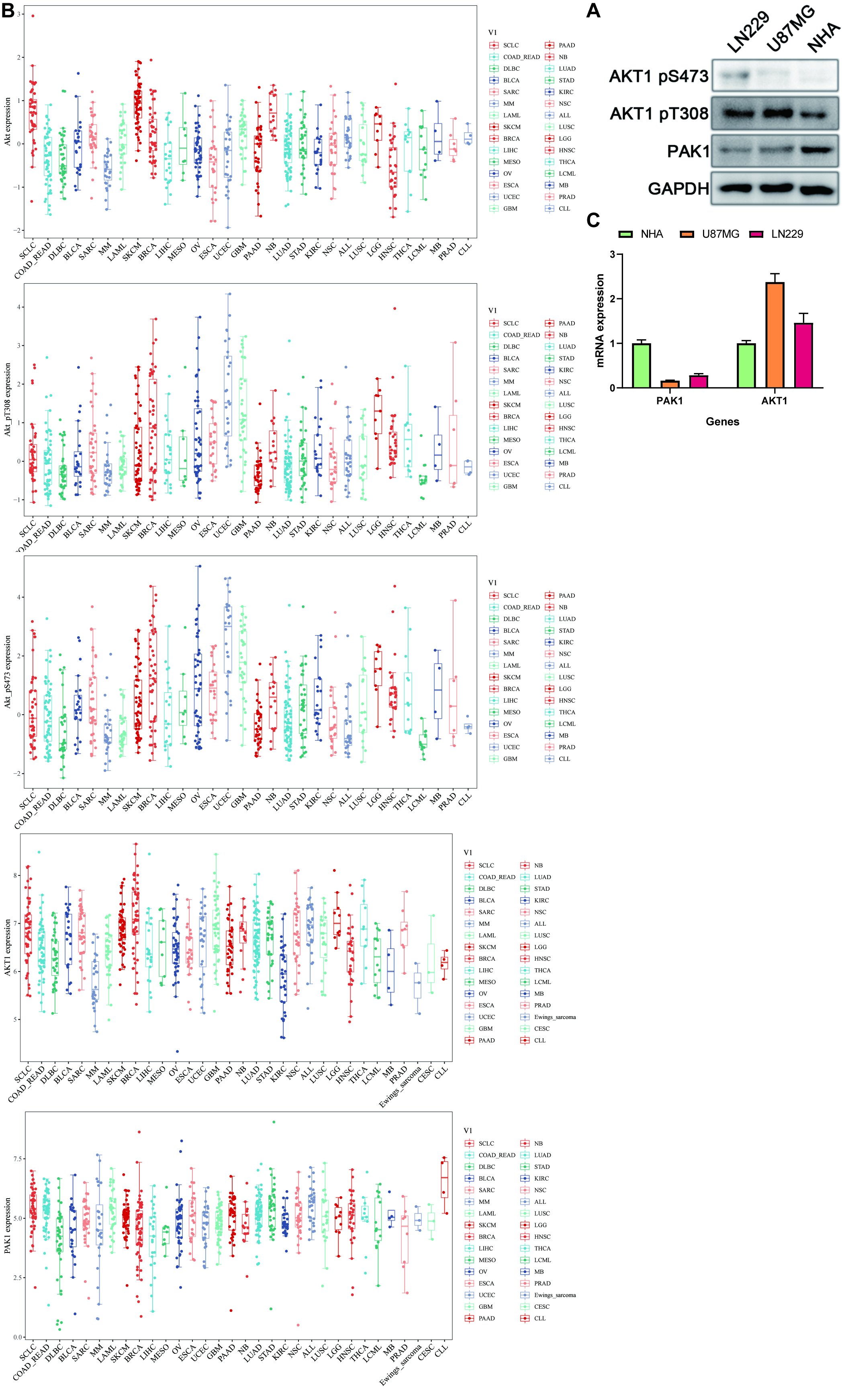

Supplement: Supplementary file 24 — Additional file 24: Figure S22. (A) PAK1 and AKT1 mRNA, protein levels of AKT, AKT pS473, AKT pT308 in all tumor cell lines. (B) Western blot analysis was used to detect PAK1 andPAKs in Pan-cancerpAKT levels in NHA, U87MG, and LN229 GBM cells. (C) PCR in NHA, U87MG, and LN229 for detect AKT1 and PAK1 mRNA levels. [file 12935_2022_2689_MOESM24_ESM.tif]
