# Supplementary figures and images for: Comprehensive analysis of the prognostic implications and functional exploration of PAK gene family in human cancer (part 1 of 2)
Source: Cancer Cell Int. 2022 Sep 5;22:275. doi: 10.1186/s12935-022-02689-6 (PMC9442929; doi:10.1186/s12935-022-02689-6)

LN229-Ctrl

LN229-FRAX486

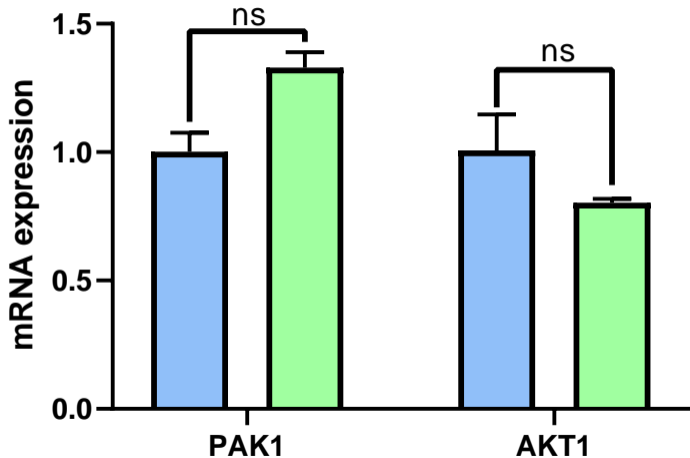

Supplement: Supplementary file 2 — Additional file 2: The raw experimental data related to this study. [file 12935_2022_2689_MOESM2_ESM.zip › PCR/LN229.pdf]

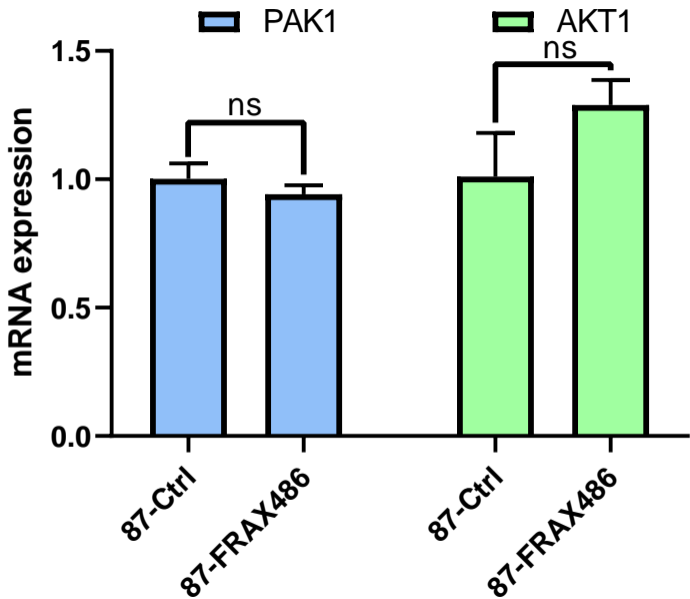

Supplement: Supplementary file 2 — Additional file 2: The raw experimental data related to this study. [file 12935_2022_2689_MOESM2_ESM.zip › PCR/U87MG.pdf]

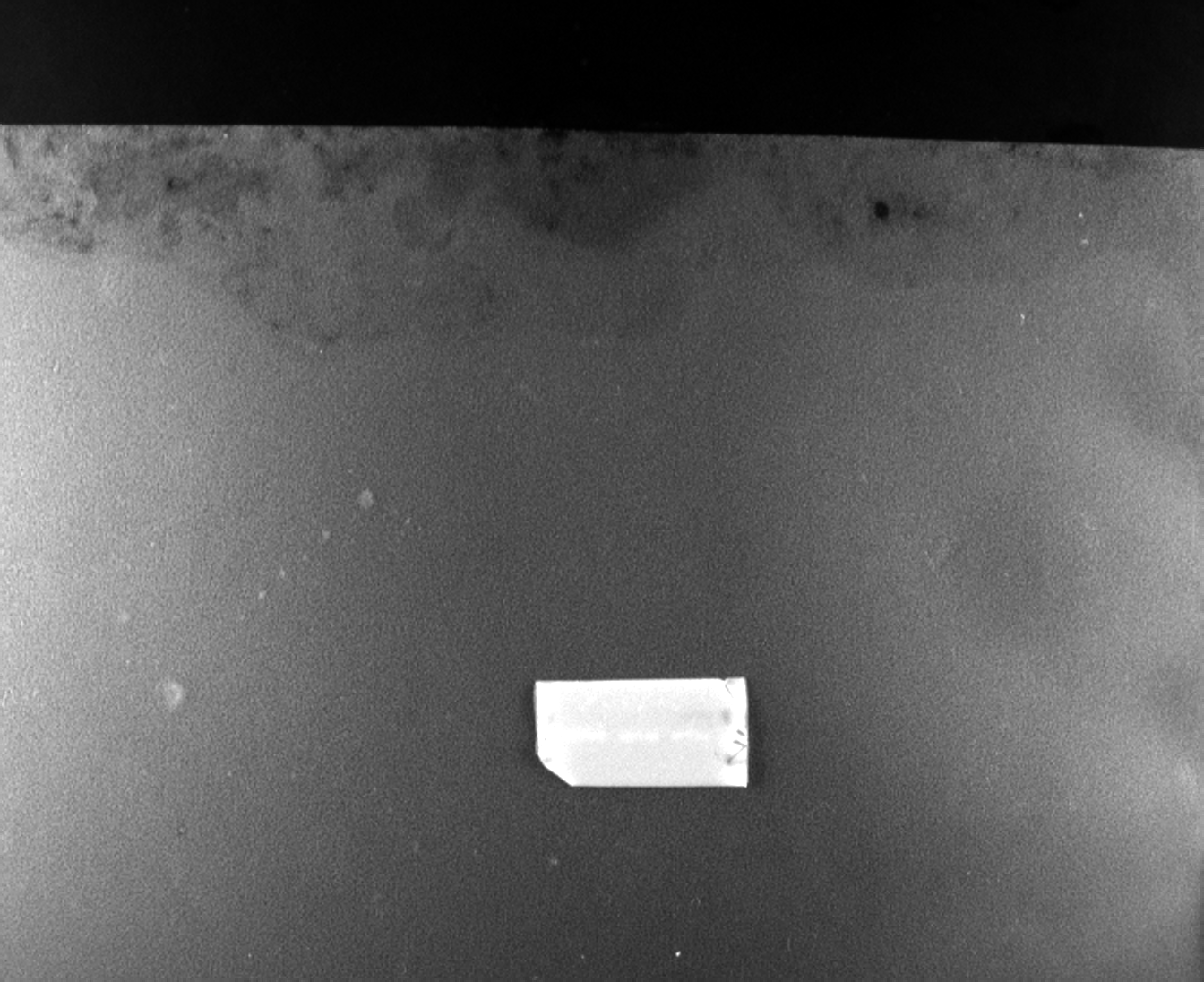

Supplement: Supplementary file 2 — Additional file 2: The raw experimental data related to this study. [file 12935_2022_2689_MOESM2_ESM.zip › WB/AKT1/229-1-2.Tif]

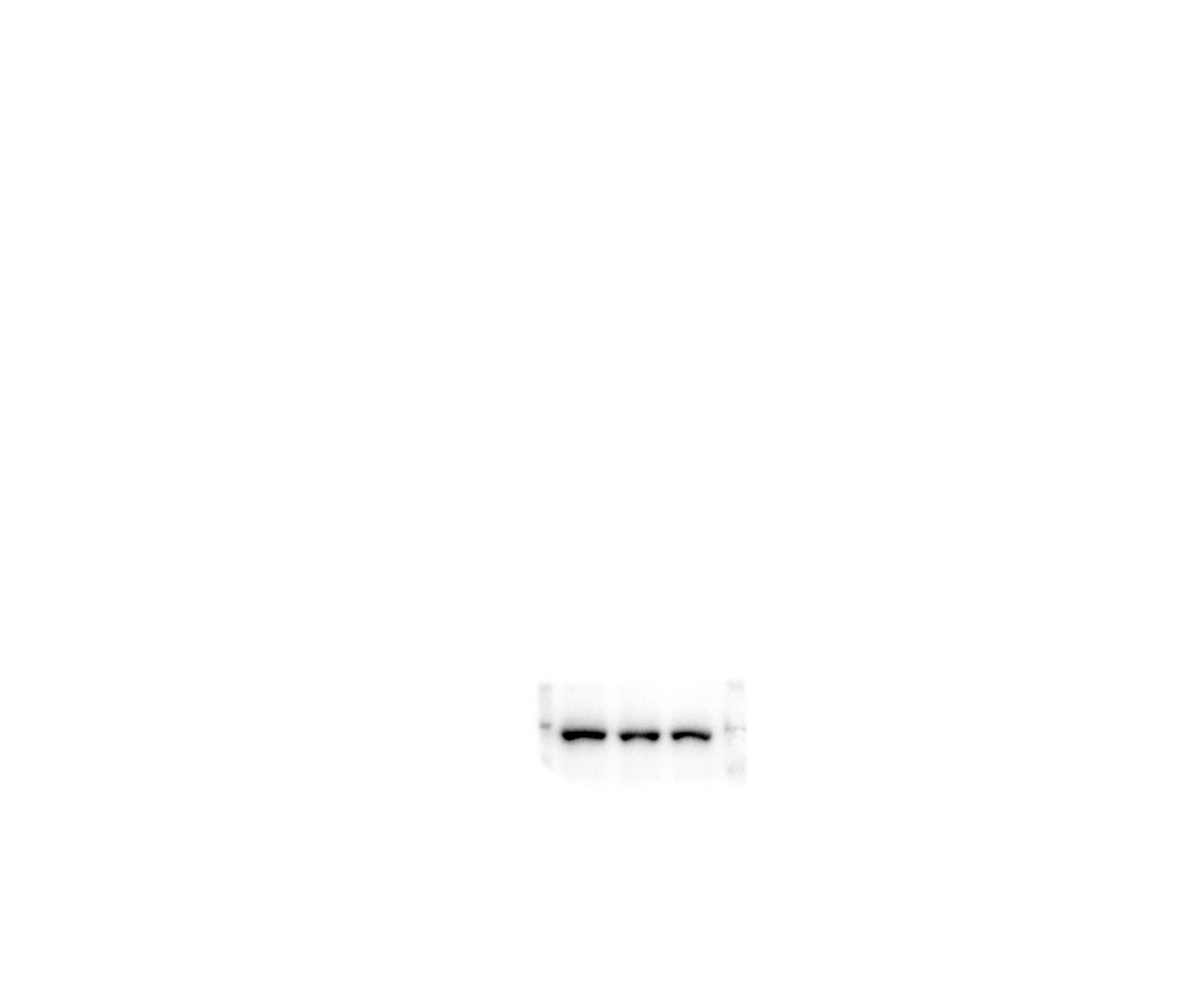

Supplement: Supplementary file 2 — Additional file 2: The raw experimental data related to this study. [file 12935_2022_2689_MOESM2_ESM.zip › WB/AKT1/229-1.Tif]

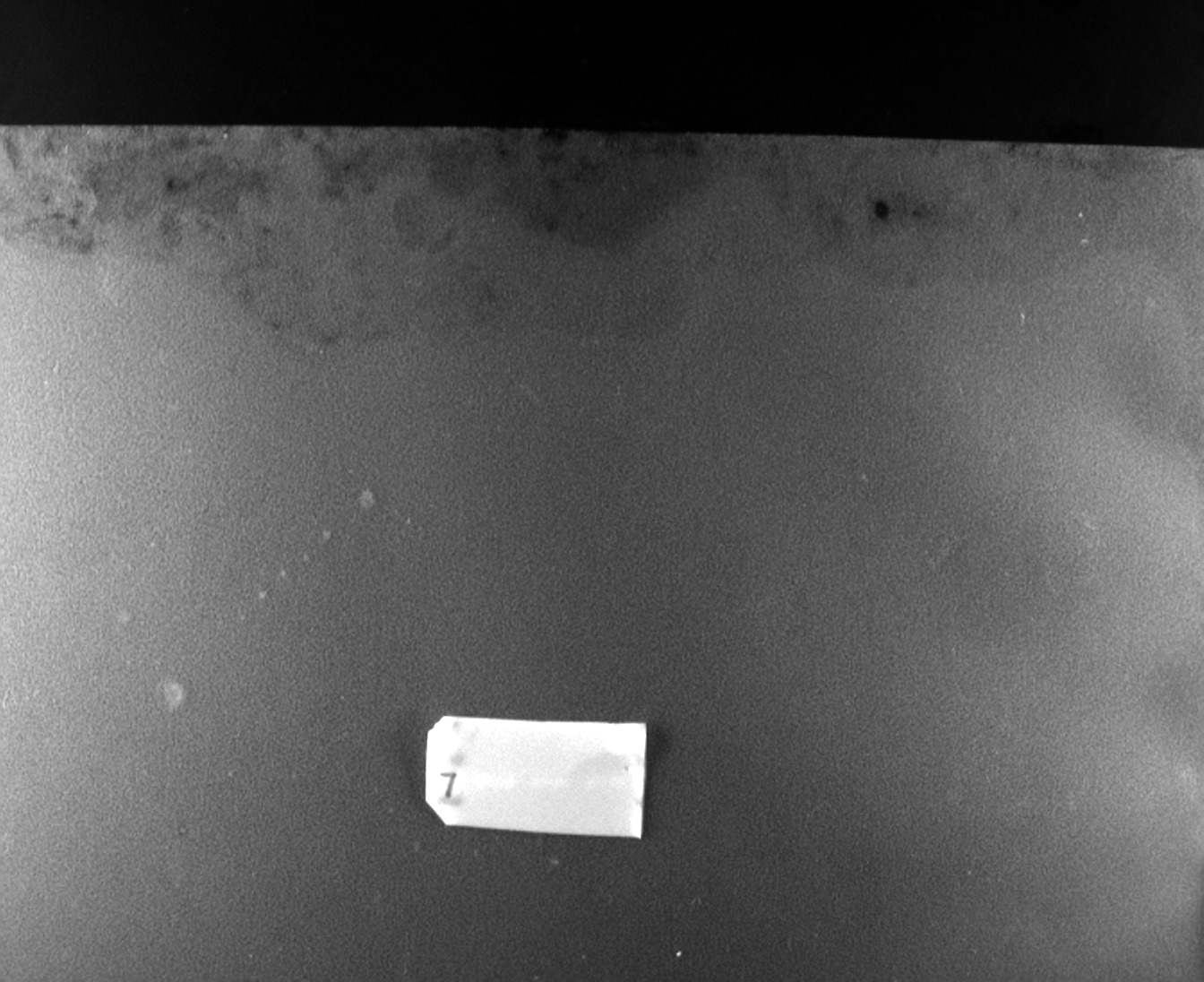

Supplement: Supplementary file 2 — Additional file 2: The raw experimental data related to this study. [file 12935_2022_2689_MOESM2_ESM.zip › WB/AKT1/229-2-2.Tif]

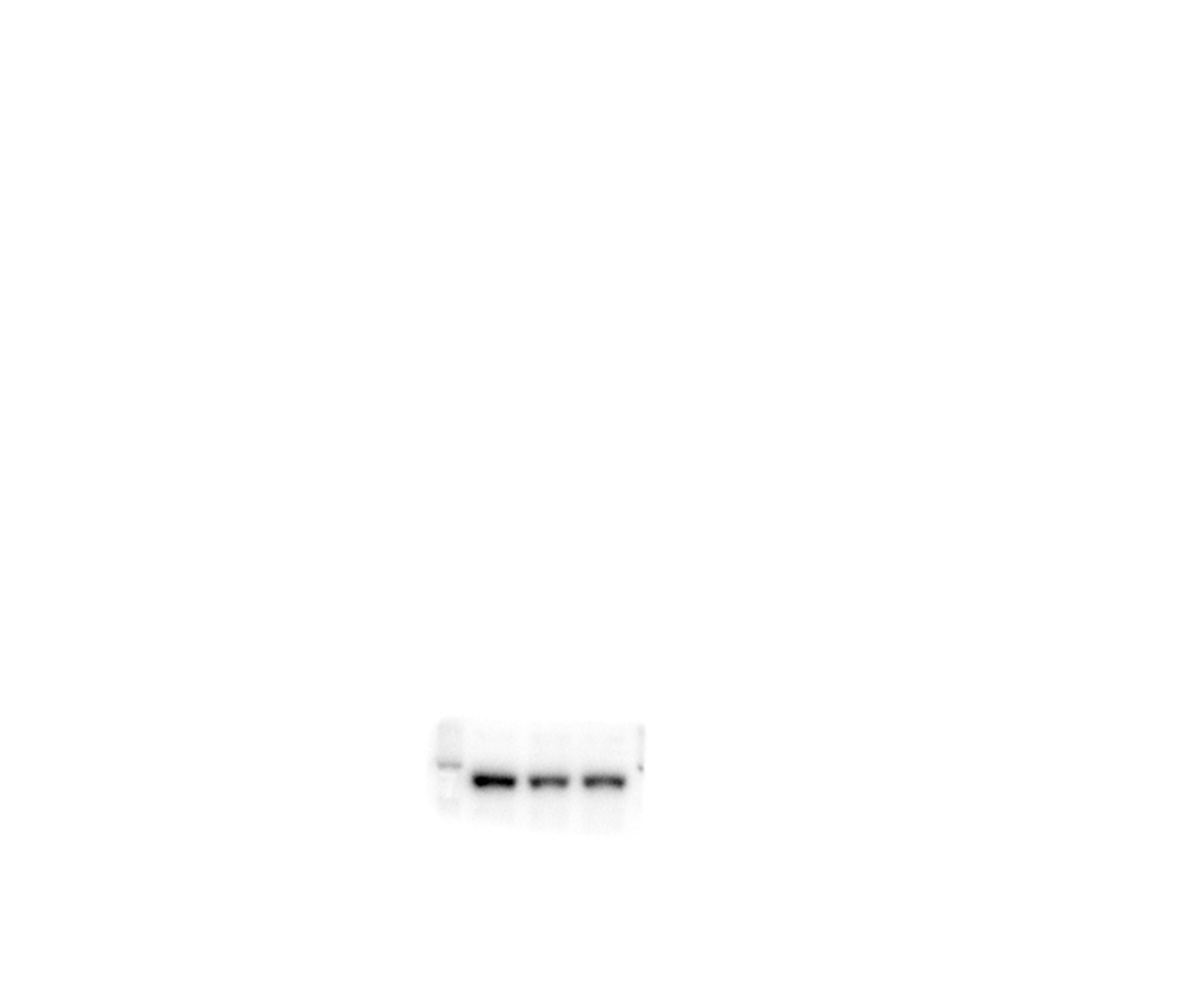

Supplement: Supplementary file 2 — Additional file 2: The raw experimental data related to this study. [file 12935_2022_2689_MOESM2_ESM.zip › WB/AKT1/229-2.Tif]

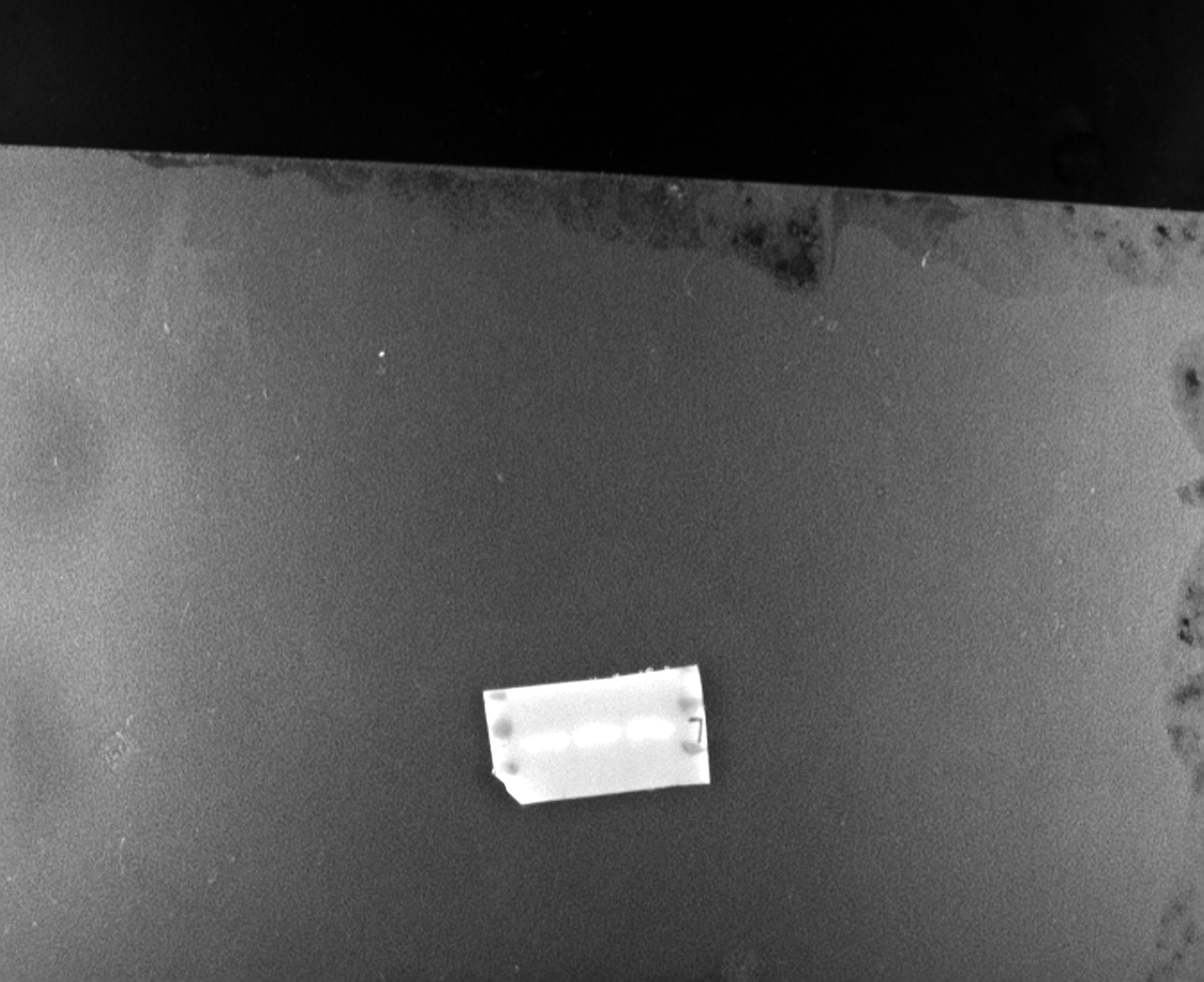

Supplement: Supplementary file 2 — Additional file 2: The raw experimental data related to this study. [file 12935_2022_2689_MOESM2_ESM.zip › WB/AKT1/229-3-2.Tif]

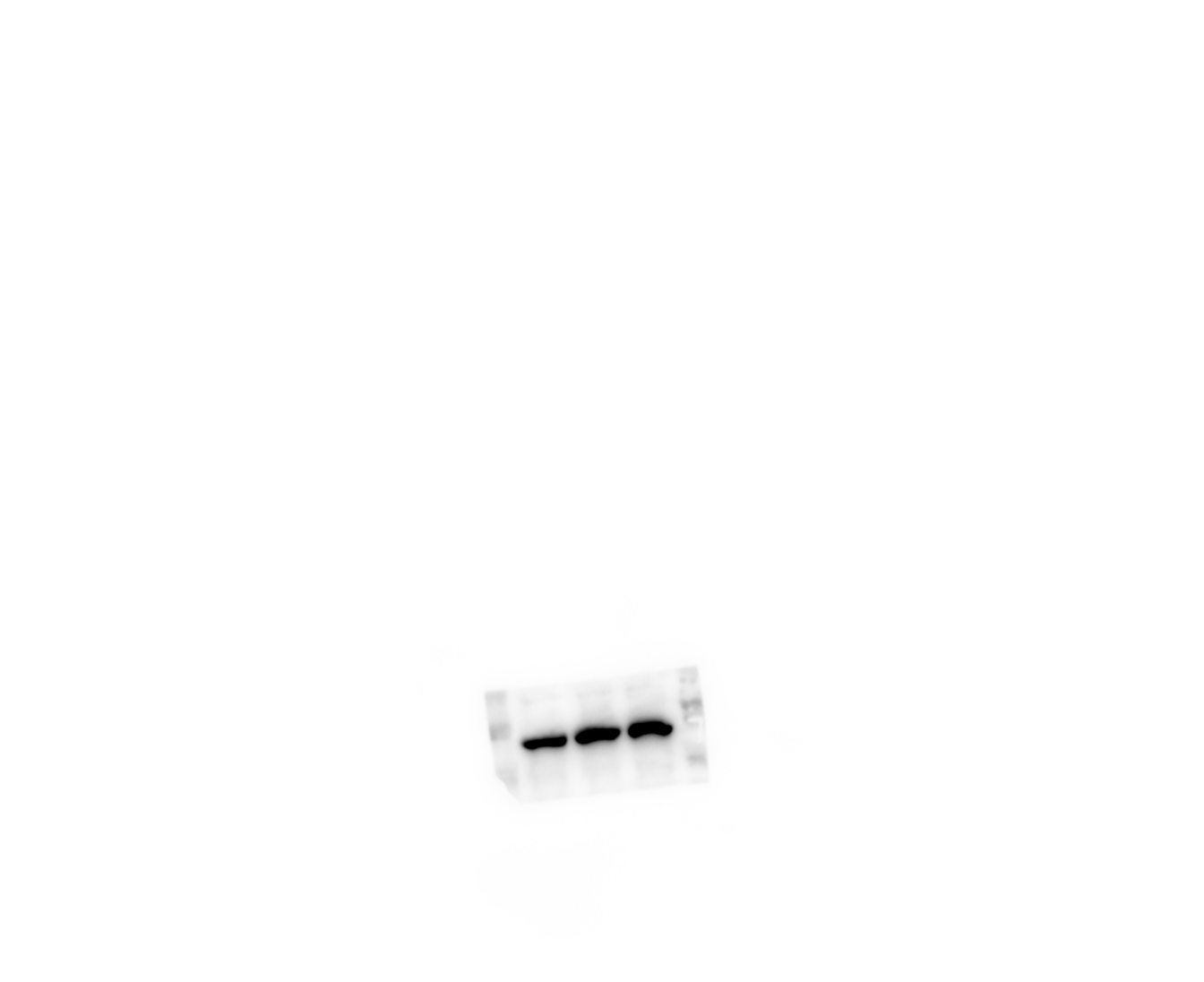

Supplement: Supplementary file 2 — Additional file 2: The raw experimental data related to this study. [file 12935_2022_2689_MOESM2_ESM.zip › WB/AKT1/229-3.Tif]

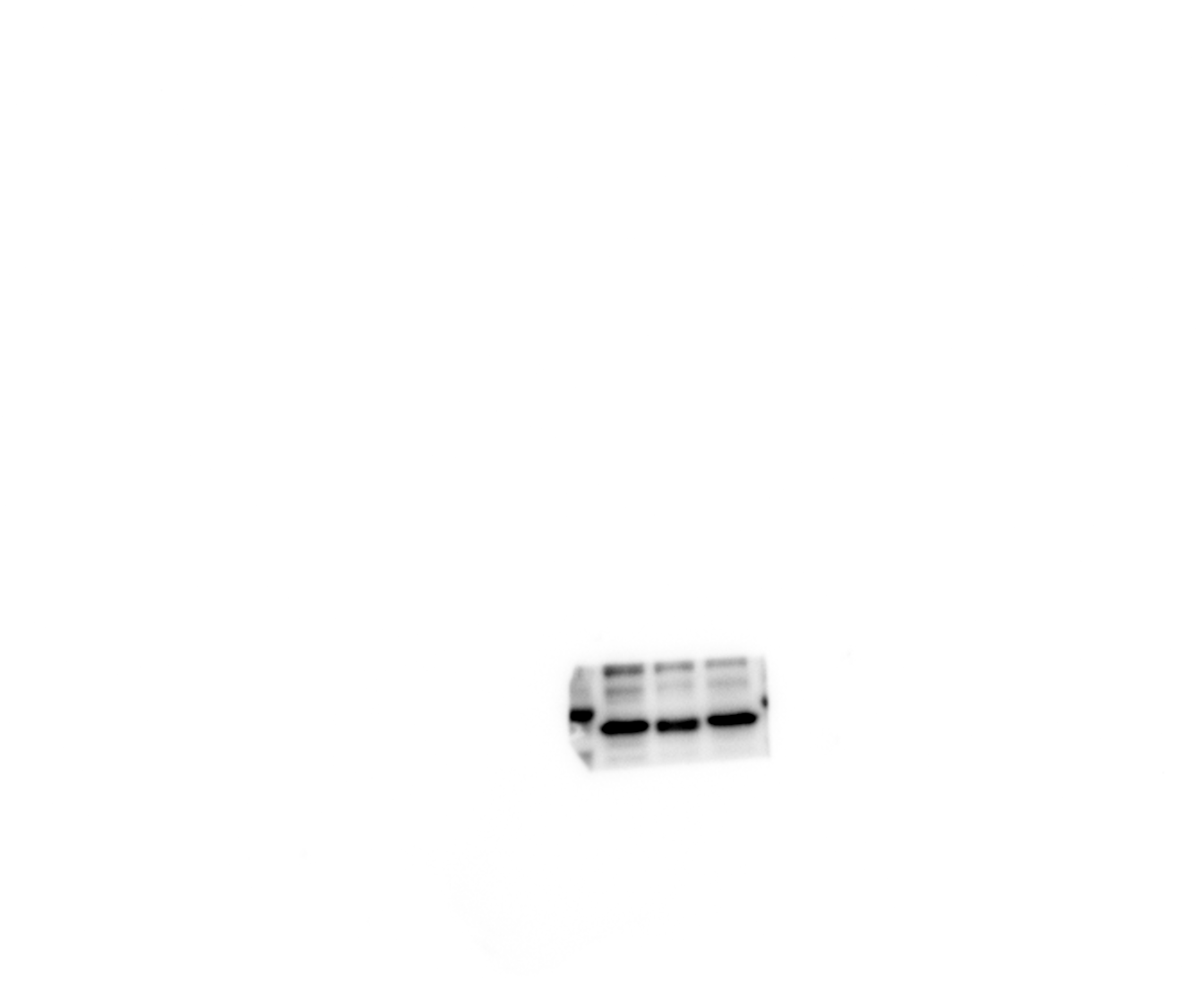

Supplement: Supplementary file 2 — Additional file 2: The raw experimental data related to this study. [file 12935_2022_2689_MOESM2_ESM.zip › WB/AKT1/87-1.Tif]

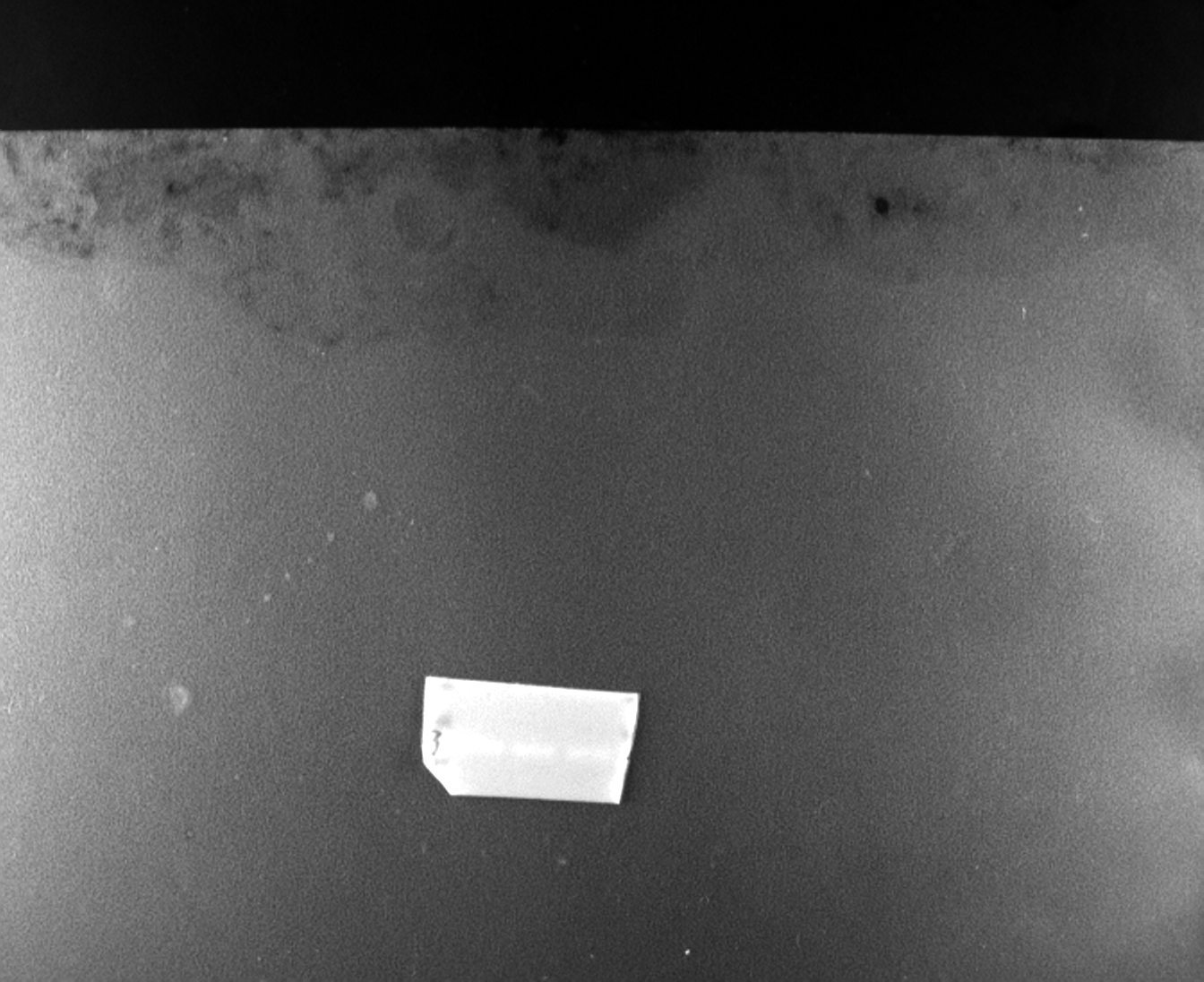

Supplement: Supplementary file 2 — Additional file 2: The raw experimental data related to this study. [file 12935_2022_2689_MOESM2_ESM.zip › WB/AKT1/87-2-2.Tif]

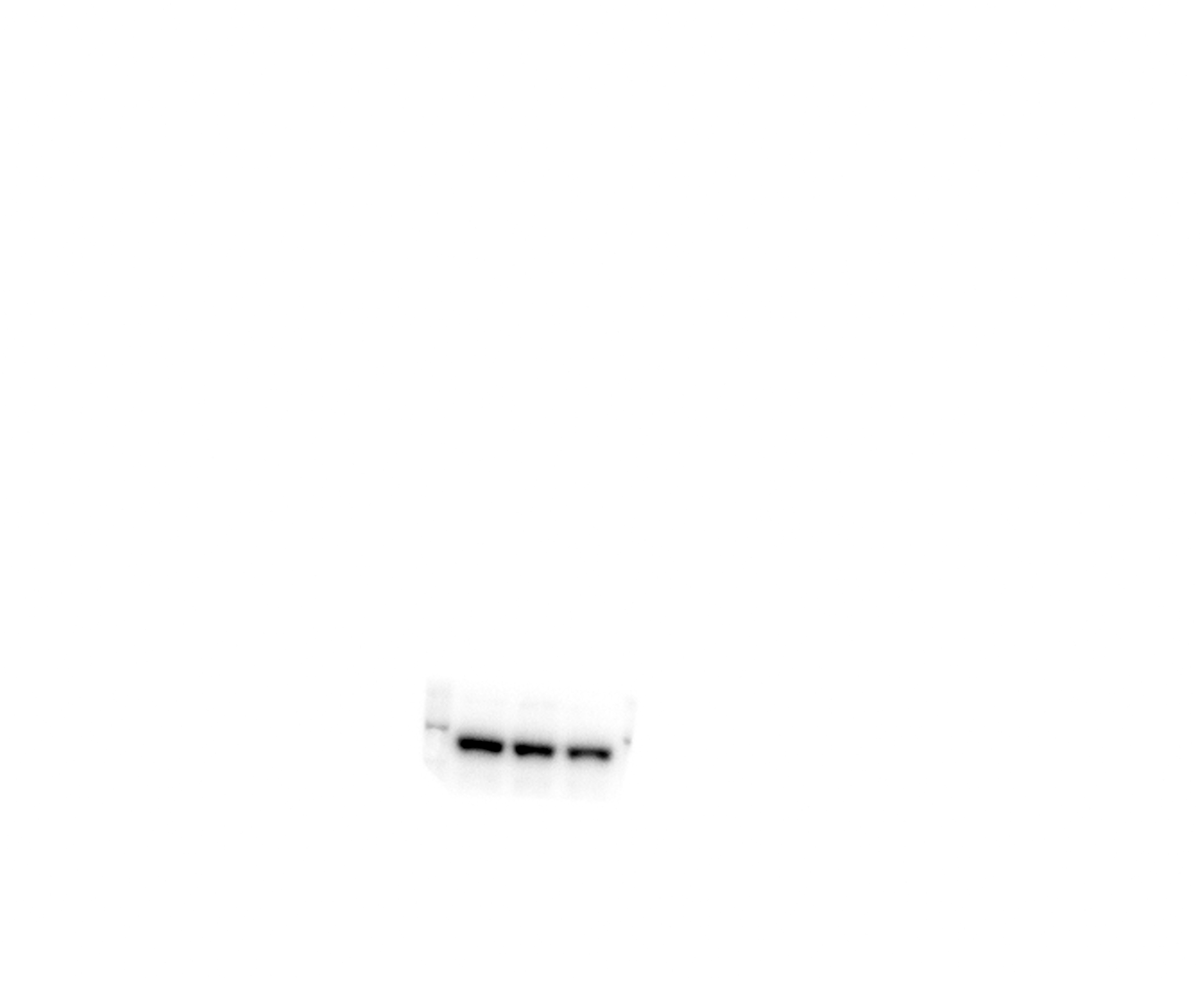

Supplement: Supplementary file 2 — Additional file 2: The raw experimental data related to this study. [file 12935_2022_2689_MOESM2_ESM.zip › WB/AKT1/87-2.Tif]

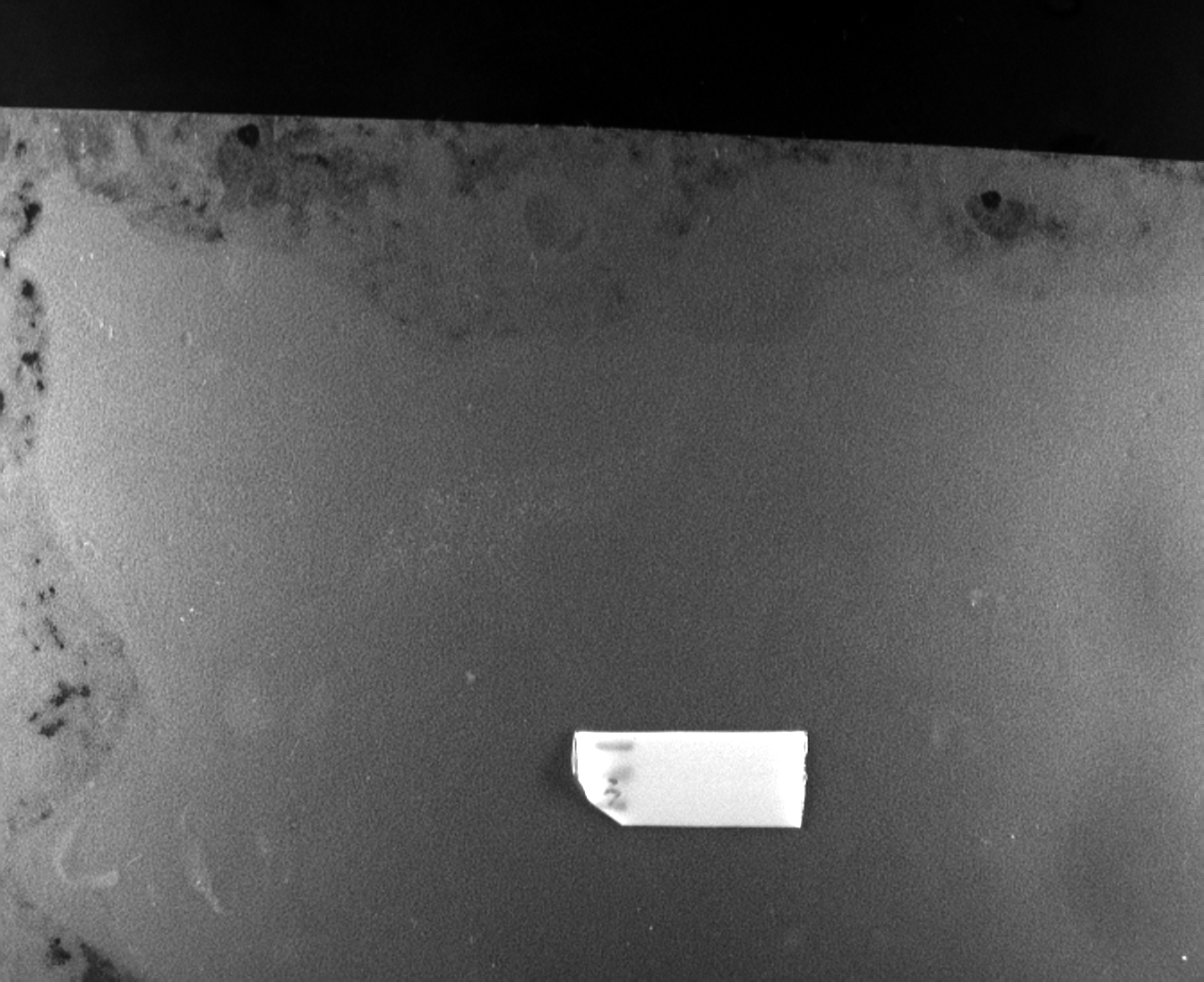

Supplement: Supplementary file 2 — Additional file 2: The raw experimental data related to this study. [file 12935_2022_2689_MOESM2_ESM.zip › WB/AKT1/87-3-2.Tif]

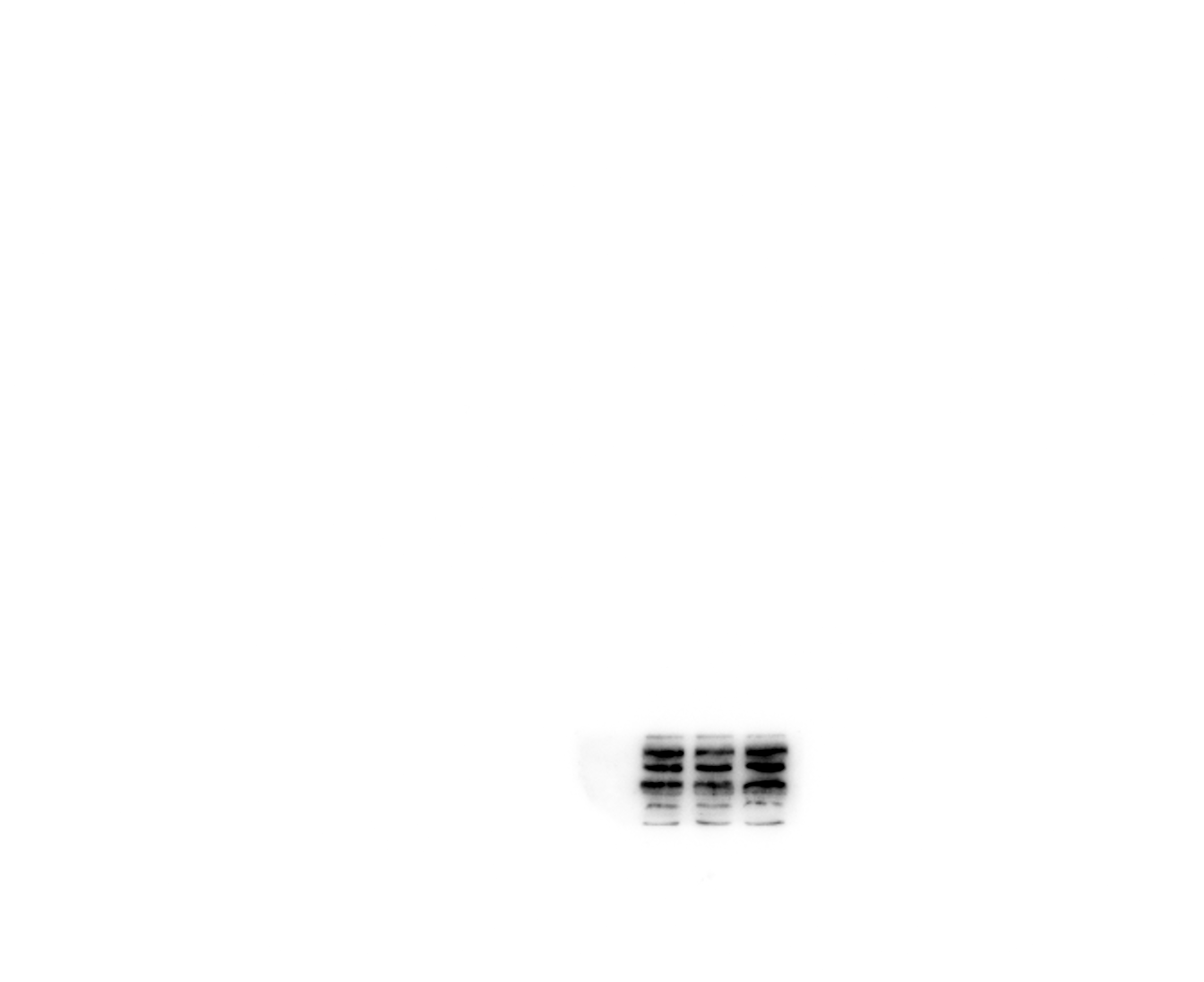

Supplement: Supplementary file 2 — Additional file 2: The raw experimental data related to this study. [file 12935_2022_2689_MOESM2_ESM.zip › WB/AKT1/87-3.Tif]

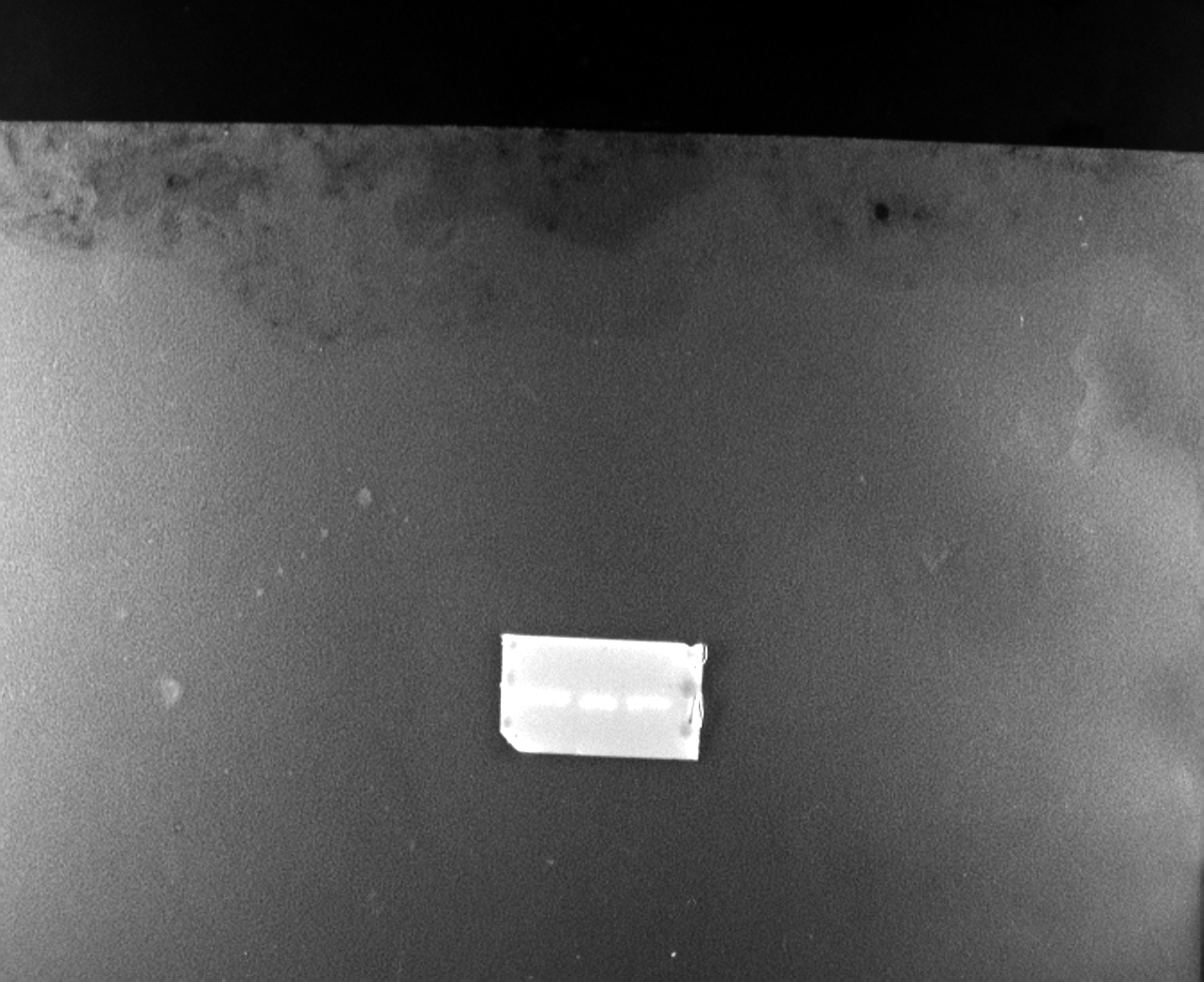

Supplement: Supplementary file 2 — Additional file 2: The raw experimental data related to this study. [file 12935_2022_2689_MOESM2_ESM.zip › WB/AKT1/87-4-2.Tif]

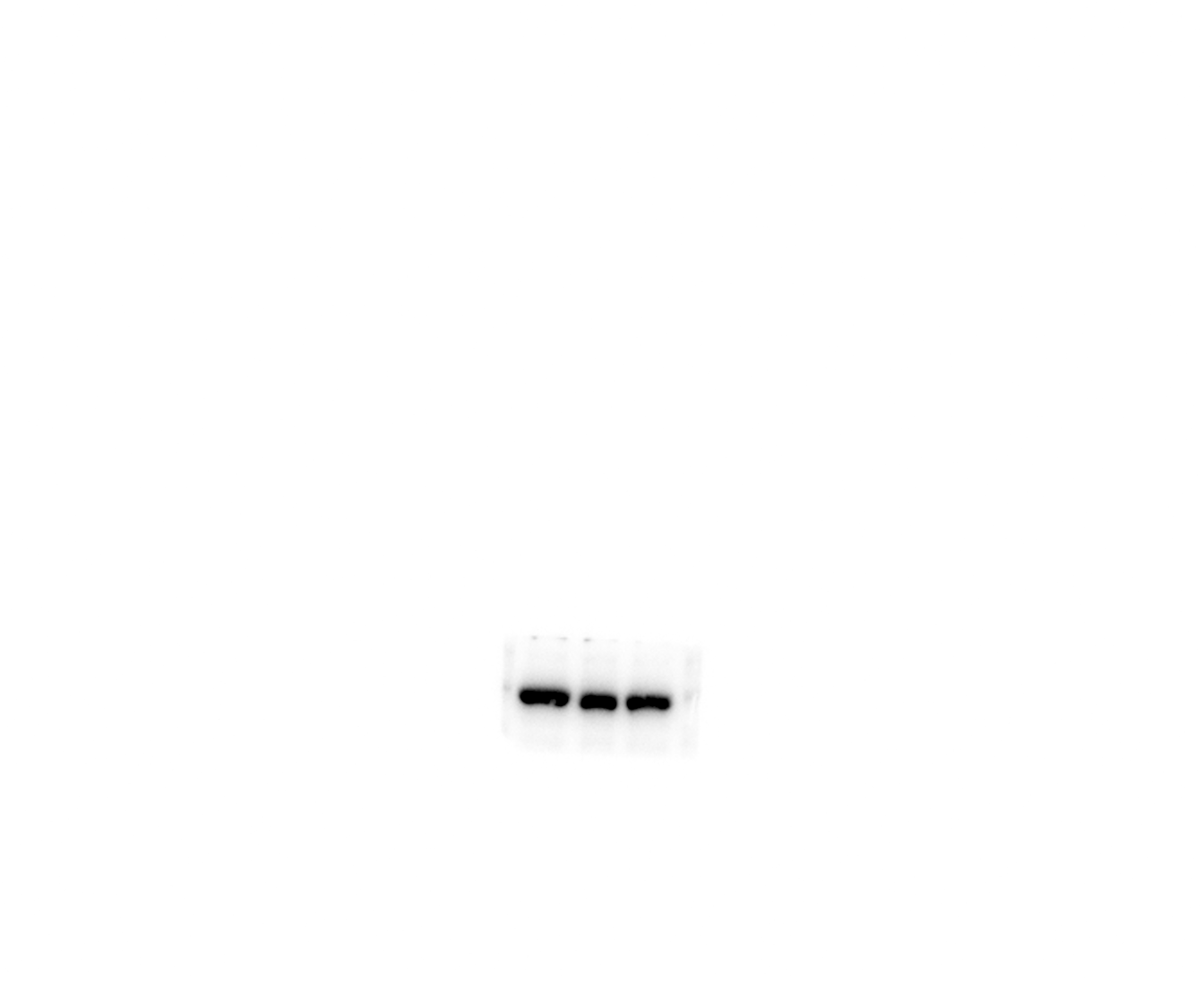

Supplement: Supplementary file 2 — Additional file 2: The raw experimental data related to this study. [file 12935_2022_2689_MOESM2_ESM.zip › WB/AKT1/87-4.Tif]

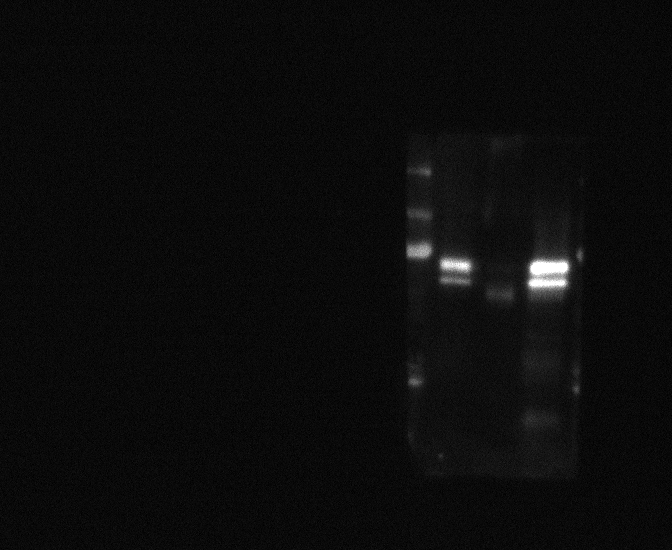

Supplement: Supplementary file 2 — Additional file 2: The raw experimental data related to this study. [file 12935_2022_2689_MOESM2_ESM.zip › WB/AKT1-IP/229-AKT--2.tif]

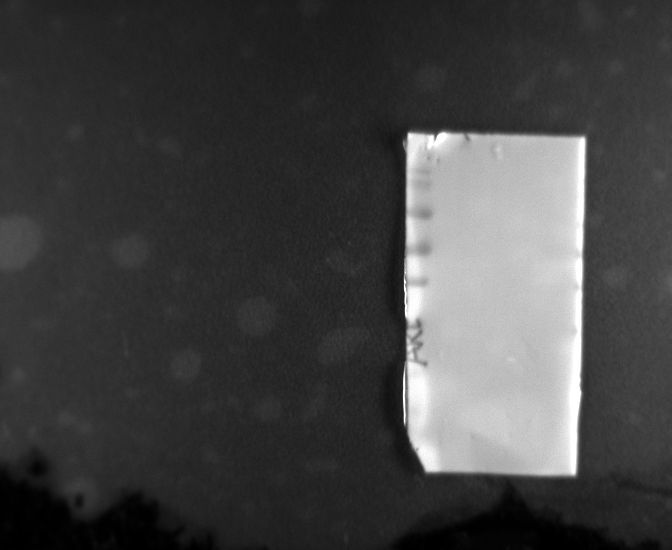

Supplement: Supplementary file 2 — Additional file 2: The raw experimental data related to this study. [file 12935_2022_2689_MOESM2_ESM.zip › WB/AKT1-IP/229-AKT--3.tif]

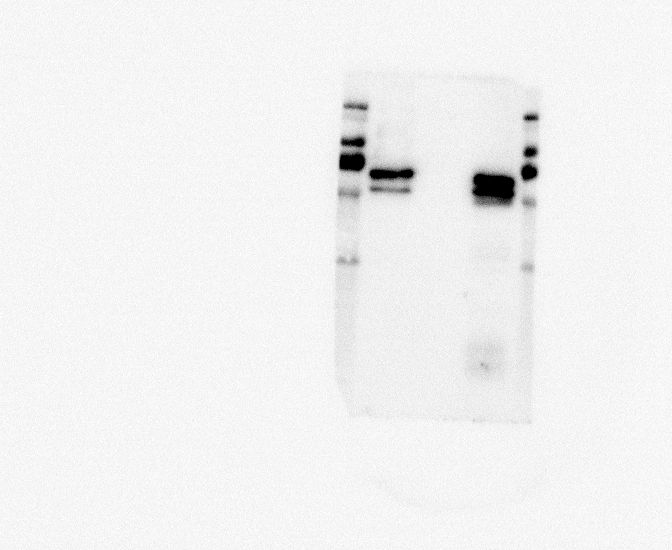

Supplement: Supplementary file 2 — Additional file 2: The raw experimental data related to this study. [file 12935_2022_2689_MOESM2_ESM.zip › WB/AKT1-IP/229-AKT-1.tif]

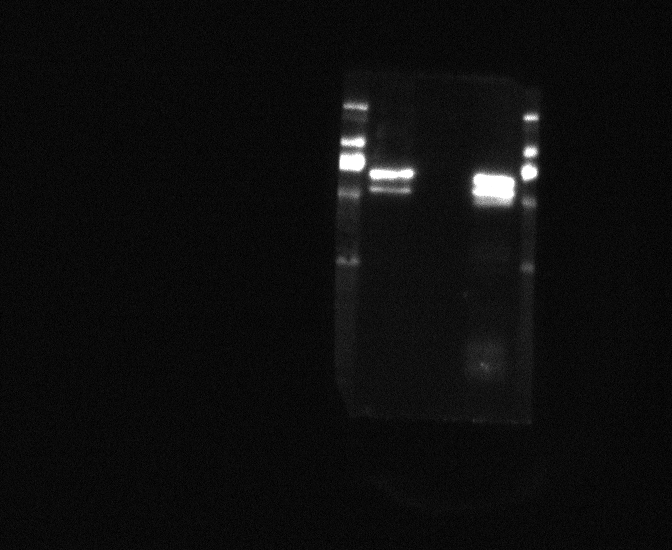

Supplement: Supplementary file 2 — Additional file 2: The raw experimental data related to this study. [file 12935_2022_2689_MOESM2_ESM.zip › WB/AKT1-IP/229-AKT-2.tif]

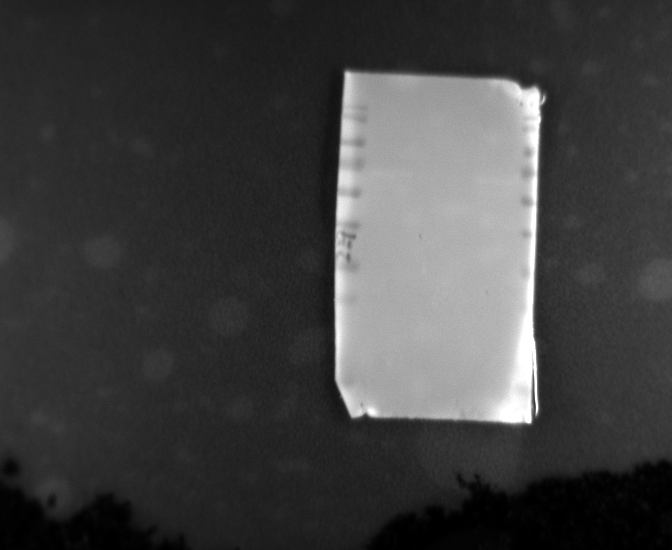

Supplement: Supplementary file 2 — Additional file 2: The raw experimental data related to this study. [file 12935_2022_2689_MOESM2_ESM.zip › WB/AKT1-IP/229-AKT-3.tif]

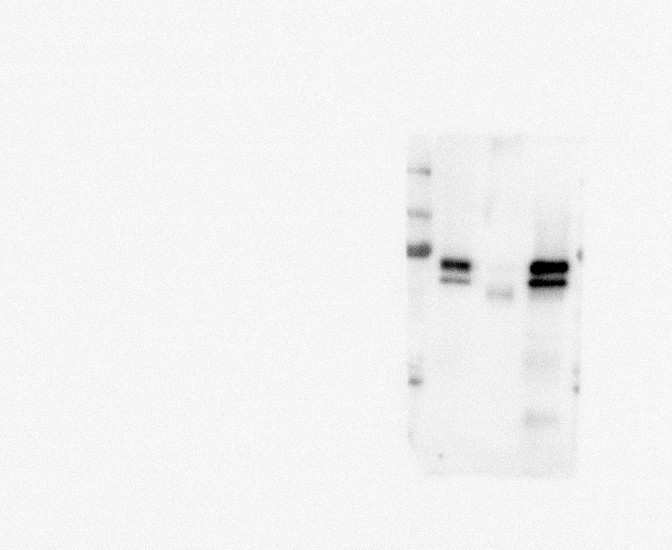

Supplement: Supplementary file 2 — Additional file 2: The raw experimental data related to this study. [file 12935_2022_2689_MOESM2_ESM.zip › WB/AKT1-IP/229-AKT.tif]

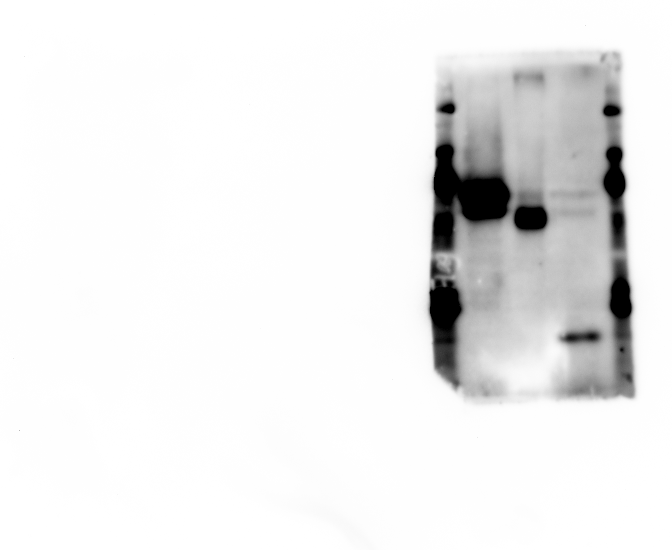

Supplement: Supplementary file 2 — Additional file 2: The raw experimental data related to this study. [file 12935_2022_2689_MOESM2_ESM.zip › WB/AKT1-IP/229-FLAG-1.tif]

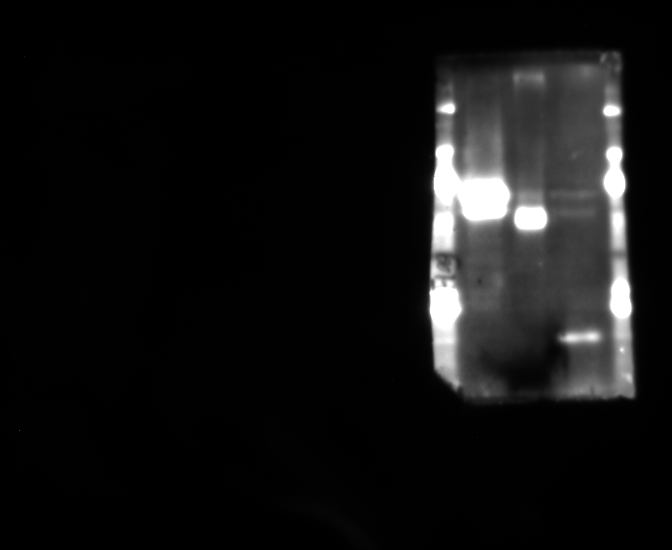

Supplement: Supplementary file 2 — Additional file 2: The raw experimental data related to this study. [file 12935_2022_2689_MOESM2_ESM.zip › WB/AKT1-IP/229-FLAG-2.tif]

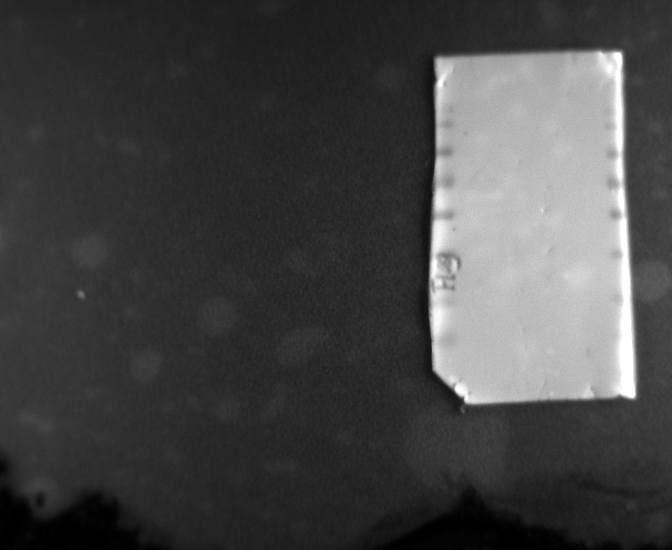

Supplement: Supplementary file 2 — Additional file 2: The raw experimental data related to this study. [file 12935_2022_2689_MOESM2_ESM.zip › WB/AKT1-IP/229-FLAG-3.tif]

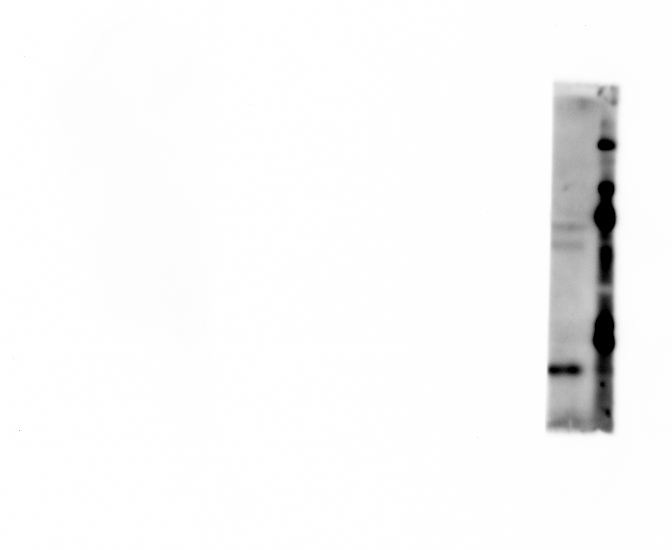

Supplement: Supplementary file 2 — Additional file 2: The raw experimental data related to this study. [file 12935_2022_2689_MOESM2_ESM.zip › WB/AKT1-IP/229-FLAG.tif]

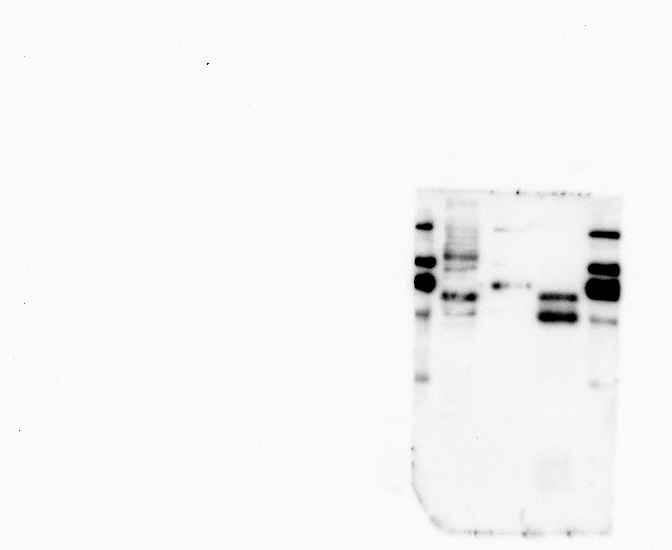

Supplement: Supplementary file 2 — Additional file 2: The raw experimental data related to this study. [file 12935_2022_2689_MOESM2_ESM.zip › WB/AKT1-IP/251-AKT-1.tif]

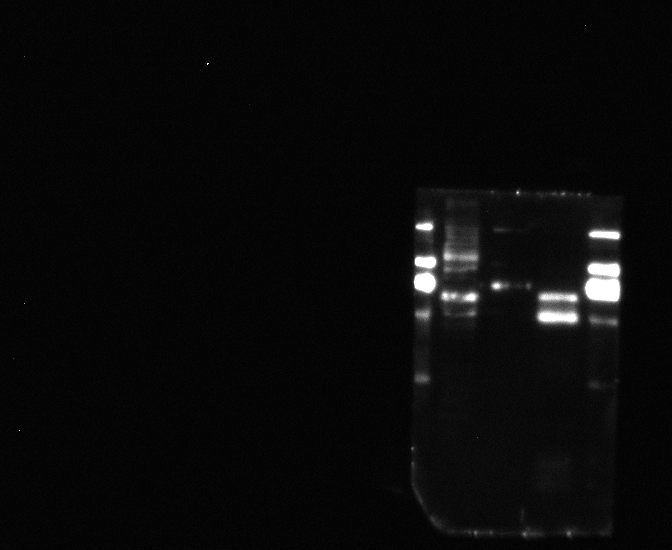

Supplement: Supplementary file 2 — Additional file 2: The raw experimental data related to this study. [file 12935_2022_2689_MOESM2_ESM.zip › WB/AKT1-IP/251-AKT-2.tif]

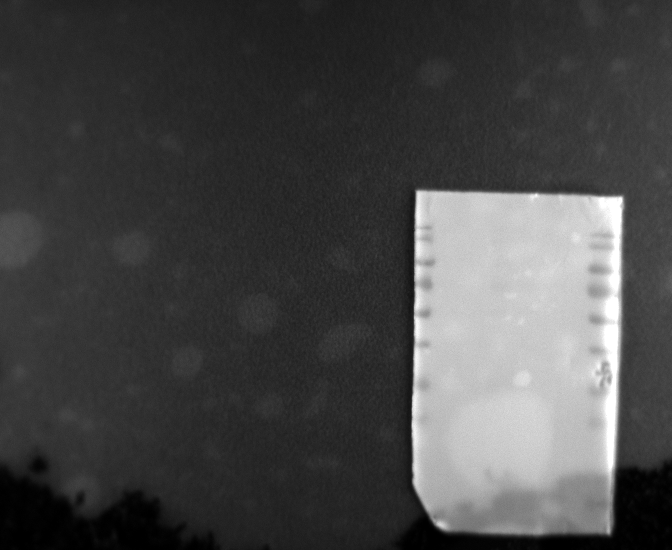

Supplement: Supplementary file 2 — Additional file 2: The raw experimental data related to this study. [file 12935_2022_2689_MOESM2_ESM.zip › WB/AKT1-IP/251-AKT-3.tif]

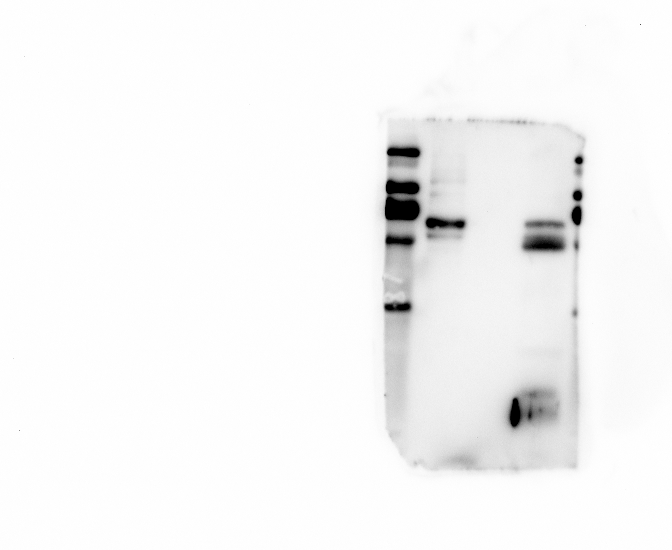

Supplement: Supplementary file 2 — Additional file 2: The raw experimental data related to this study. [file 12935_2022_2689_MOESM2_ESM.zip › WB/AKT1-IP/87-AKT-1.tif]

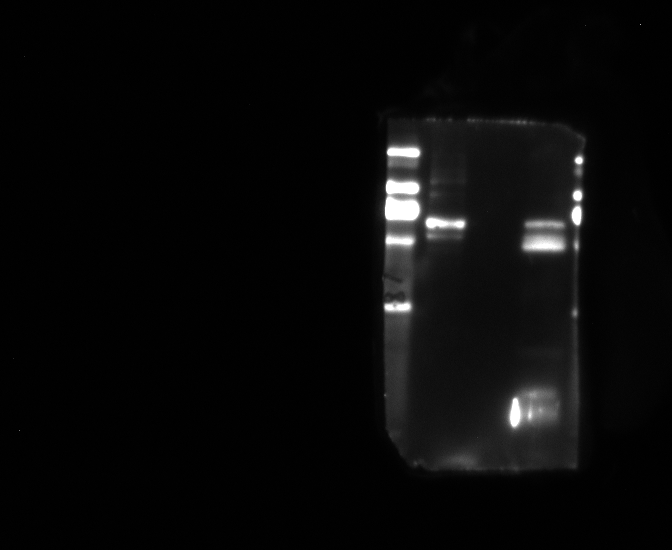

Supplement: Supplementary file 2 — Additional file 2: The raw experimental data related to this study. [file 12935_2022_2689_MOESM2_ESM.zip › WB/AKT1-IP/87-AKT-2.tif]

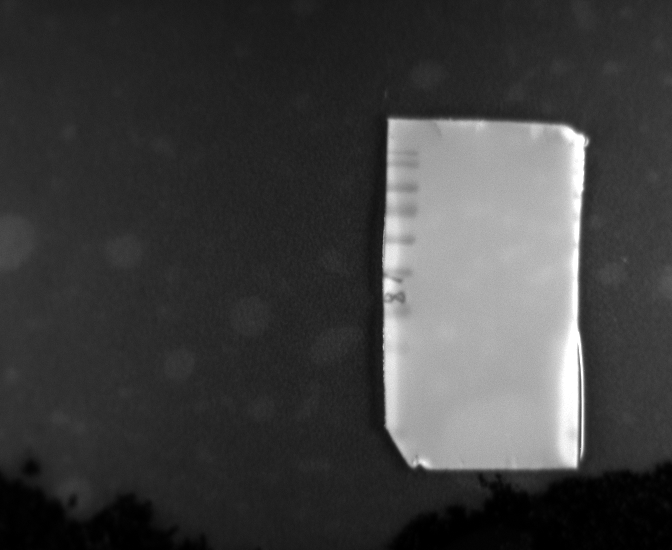

Supplement: Supplementary file 2 — Additional file 2: The raw experimental data related to this study. [file 12935_2022_2689_MOESM2_ESM.zip › WB/AKT1-IP/87-AKT-3.tif]

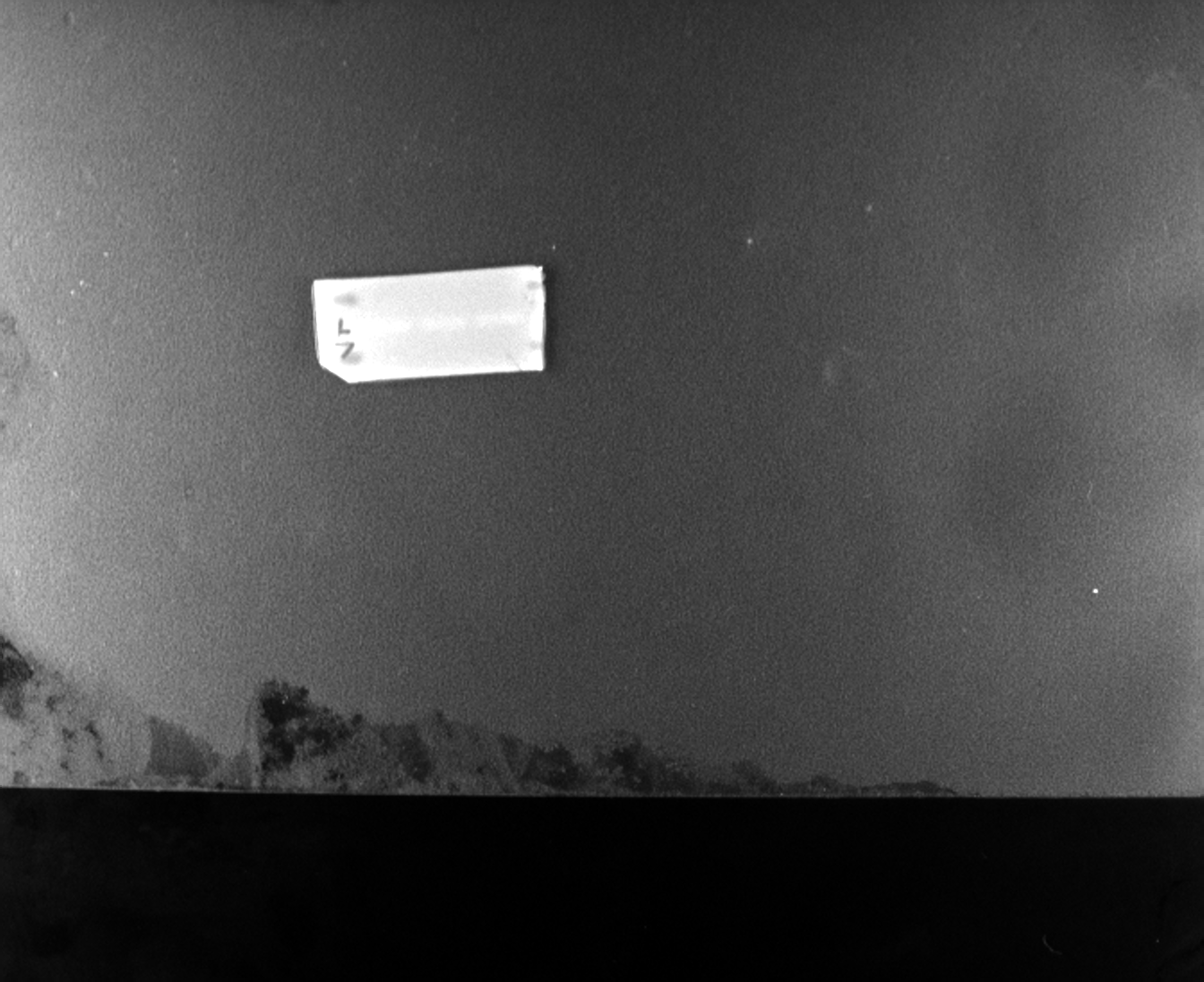

Supplement: Supplementary file 2 — Additional file 2: The raw experimental data related to this study. [file 12935_2022_2689_MOESM2_ESM.zip › WB/GAPDH/229-1-2.Tif]

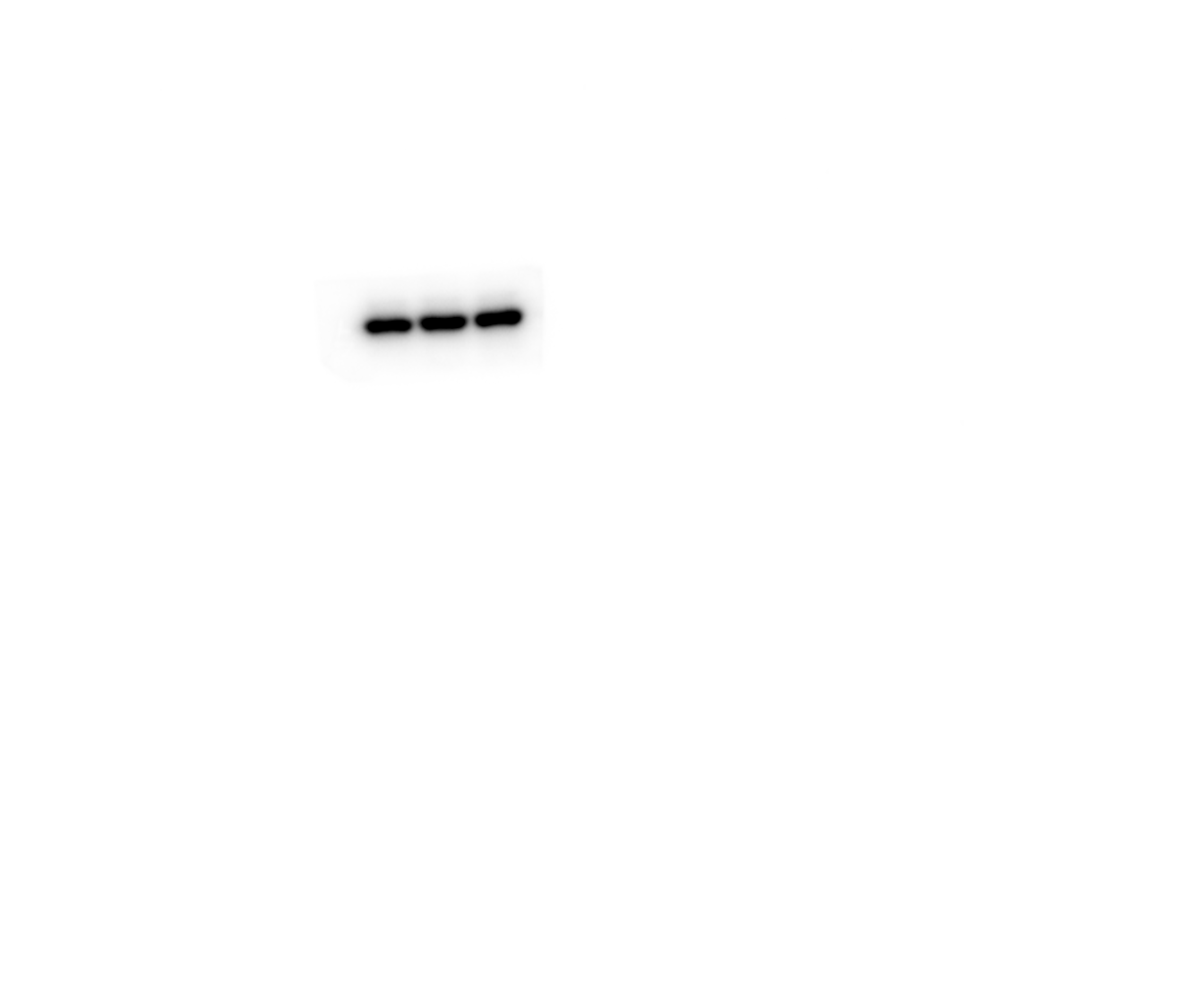

Supplement: Supplementary file 2 — Additional file 2: The raw experimental data related to this study. [file 12935_2022_2689_MOESM2_ESM.zip › WB/GAPDH/229-1.Tif]

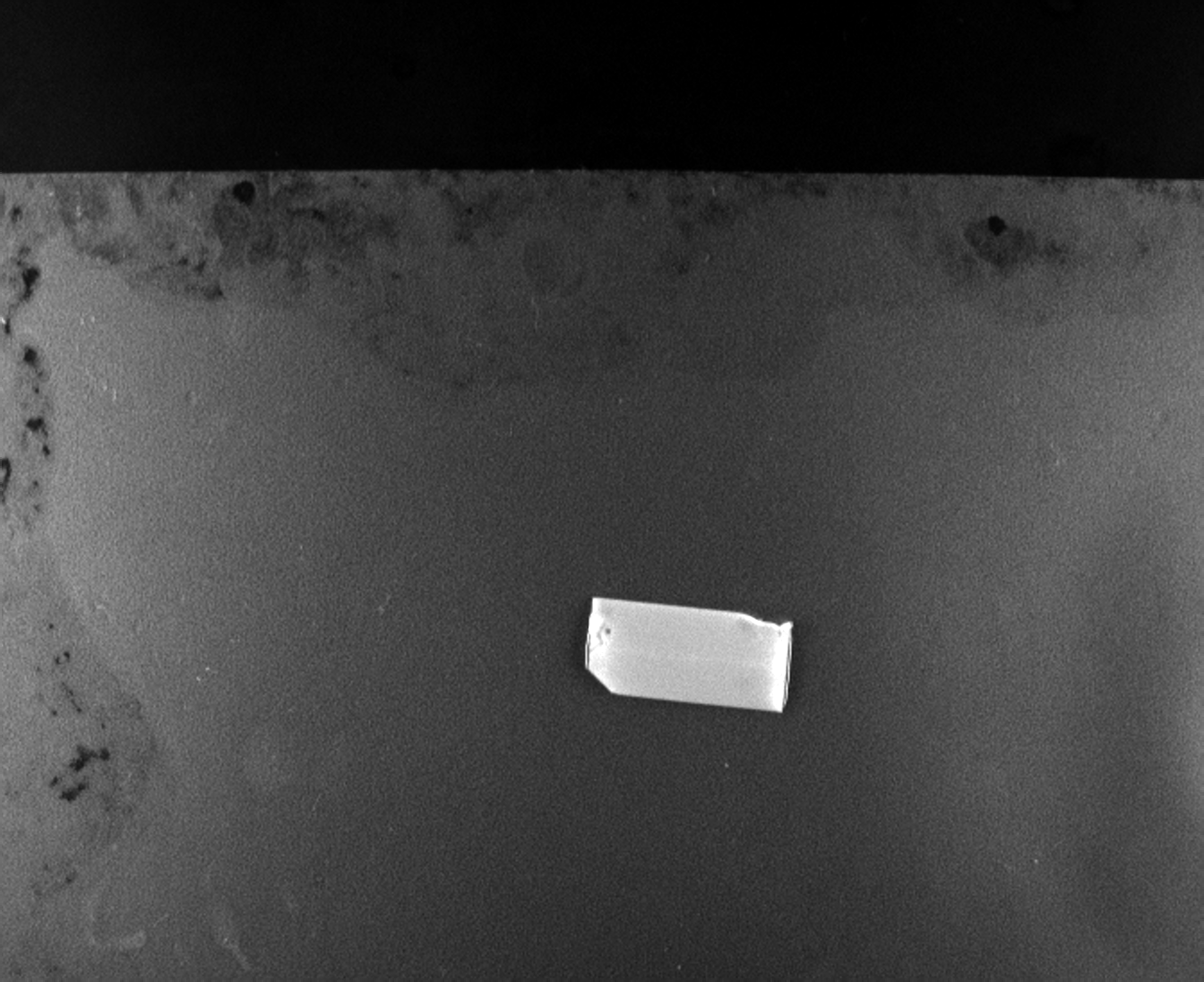

Supplement: Supplementary file 2 — Additional file 2: The raw experimental data related to this study. [file 12935_2022_2689_MOESM2_ESM.zip › WB/GAPDH/229-2-2.Tif]

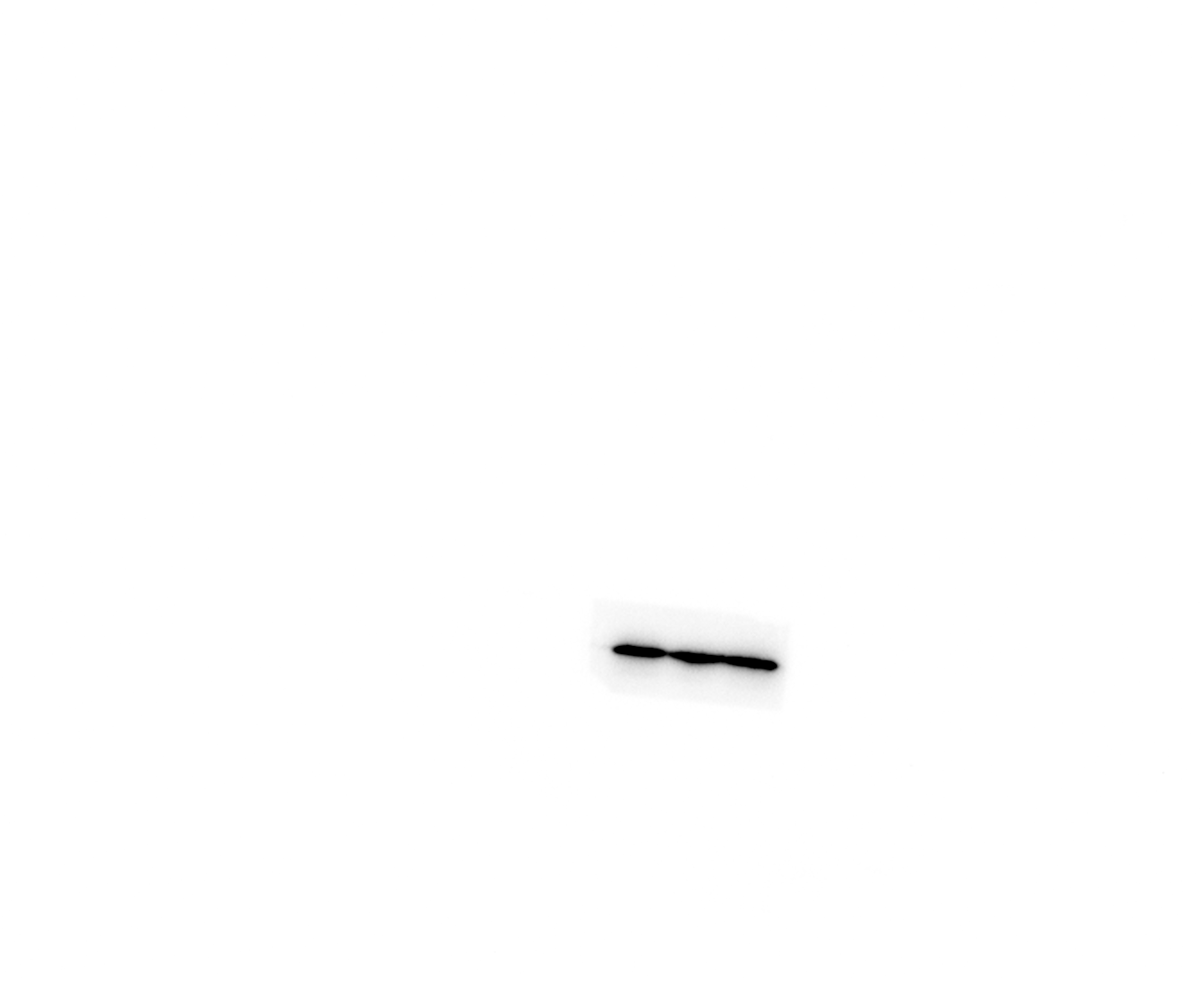

Supplement: Supplementary file 2 — Additional file 2: The raw experimental data related to this study. [file 12935_2022_2689_MOESM2_ESM.zip › WB/GAPDH/229-2.Tif]

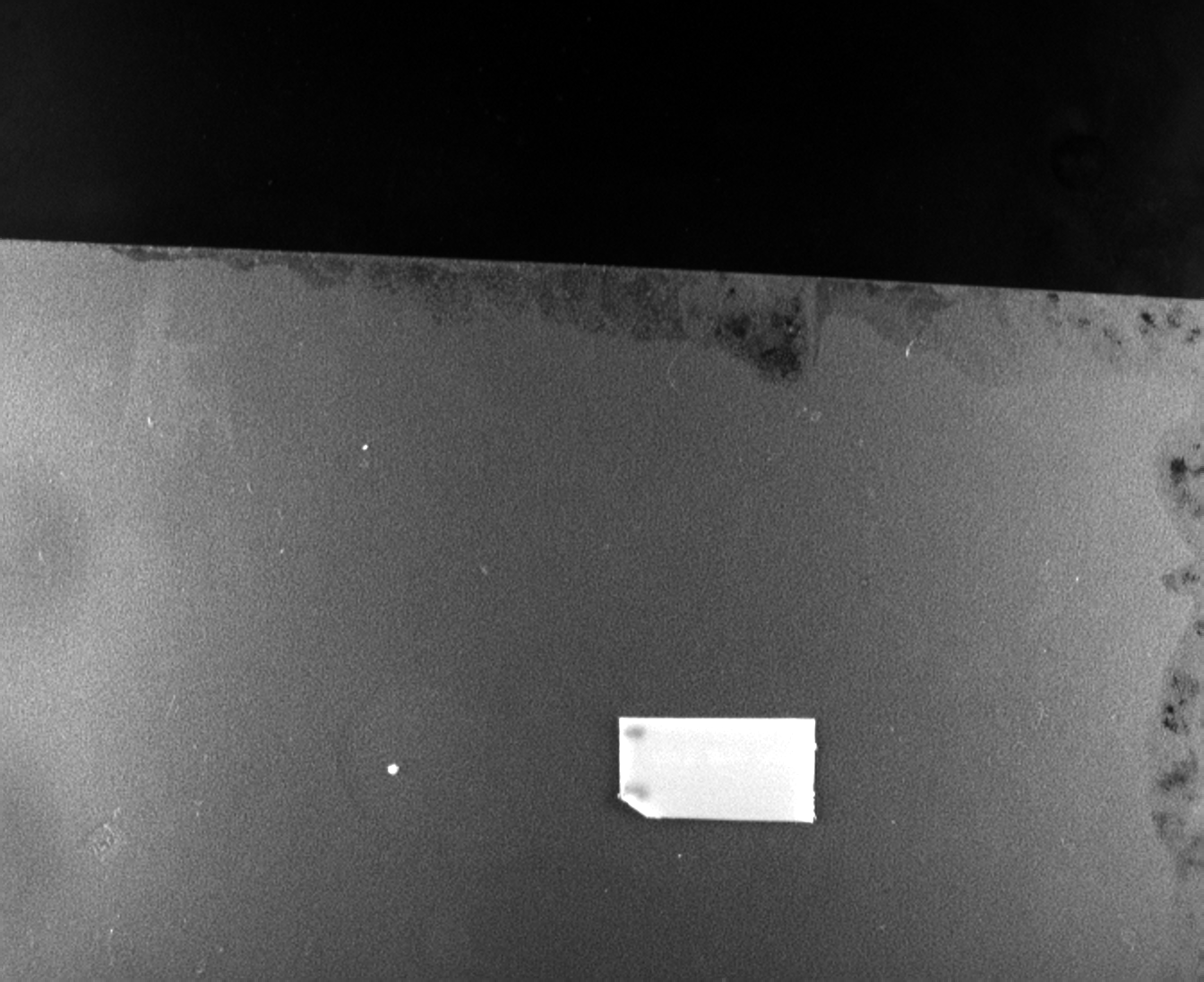

Supplement: Supplementary file 2 — Additional file 2: The raw experimental data related to this study. [file 12935_2022_2689_MOESM2_ESM.zip › WB/GAPDH/229-3-2.Tif]

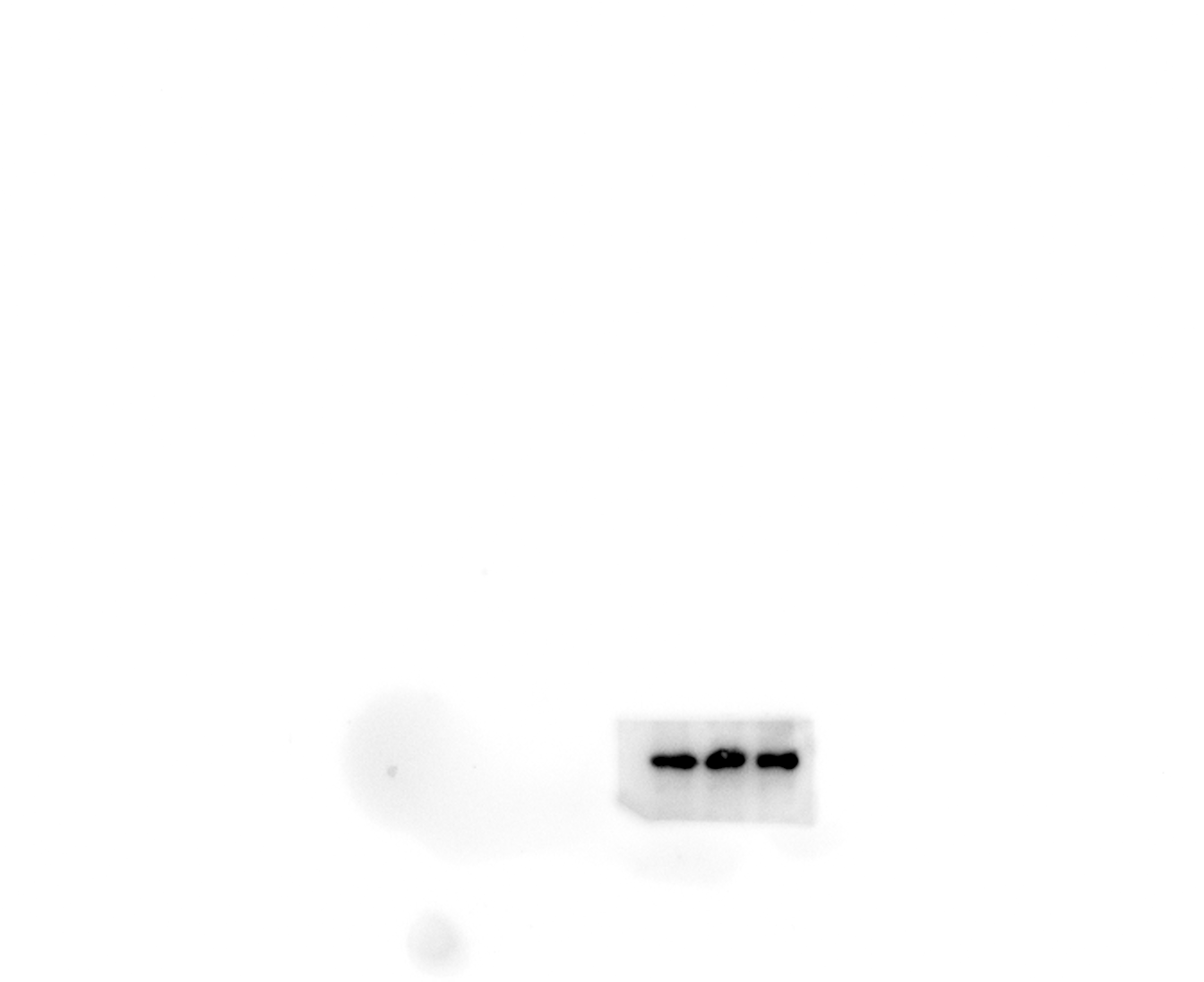

Supplement: Supplementary file 2 — Additional file 2: The raw experimental data related to this study. [file 12935_2022_2689_MOESM2_ESM.zip › WB/GAPDH/229-3.Tif]

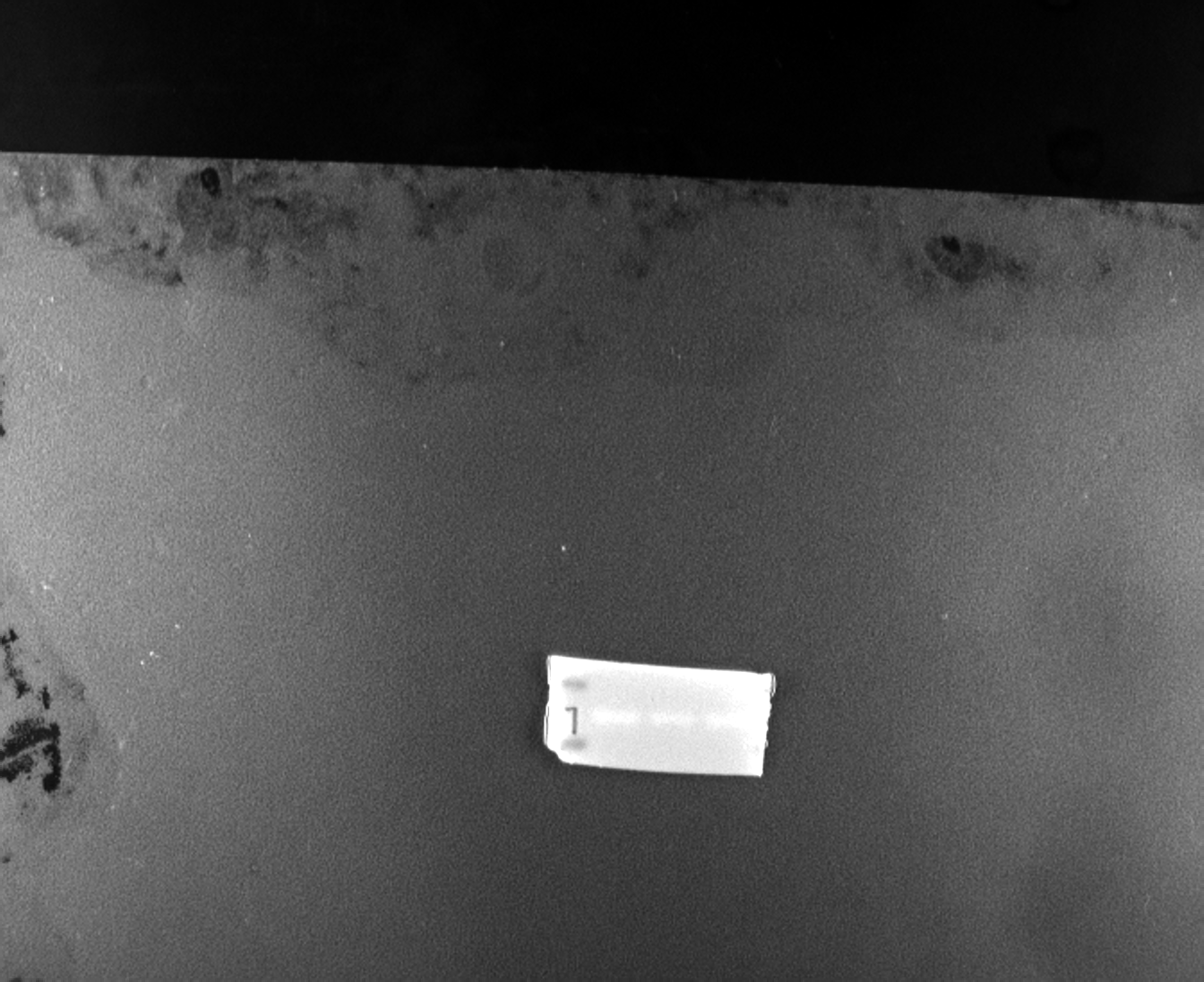

Supplement: Supplementary file 2 — Additional file 2: The raw experimental data related to this study. [file 12935_2022_2689_MOESM2_ESM.zip › WB/GAPDH/229-4-2.Tif]

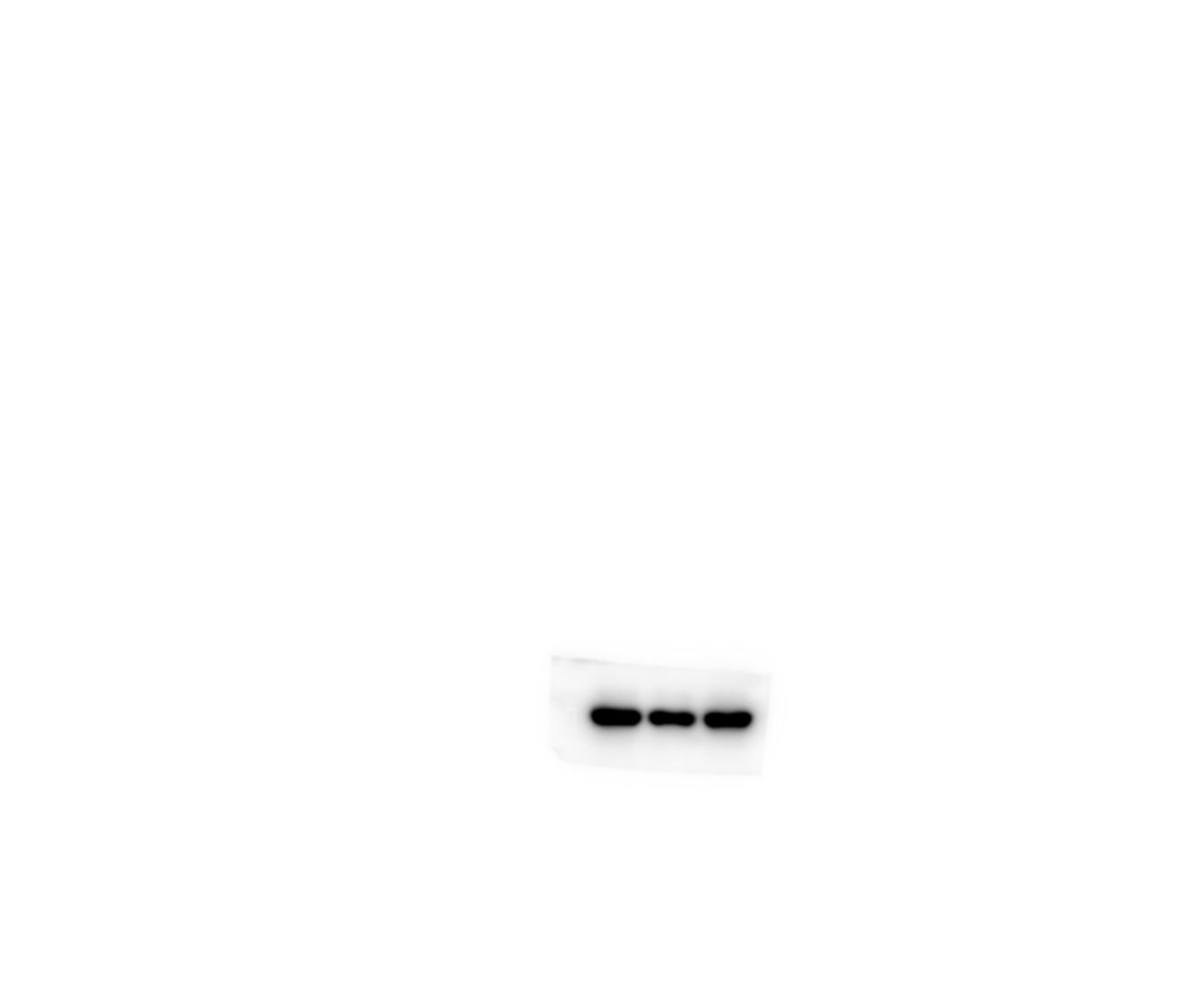

Supplement: Supplementary file 2 — Additional file 2: The raw experimental data related to this study. [file 12935_2022_2689_MOESM2_ESM.zip › WB/GAPDH/229-4.Tif]

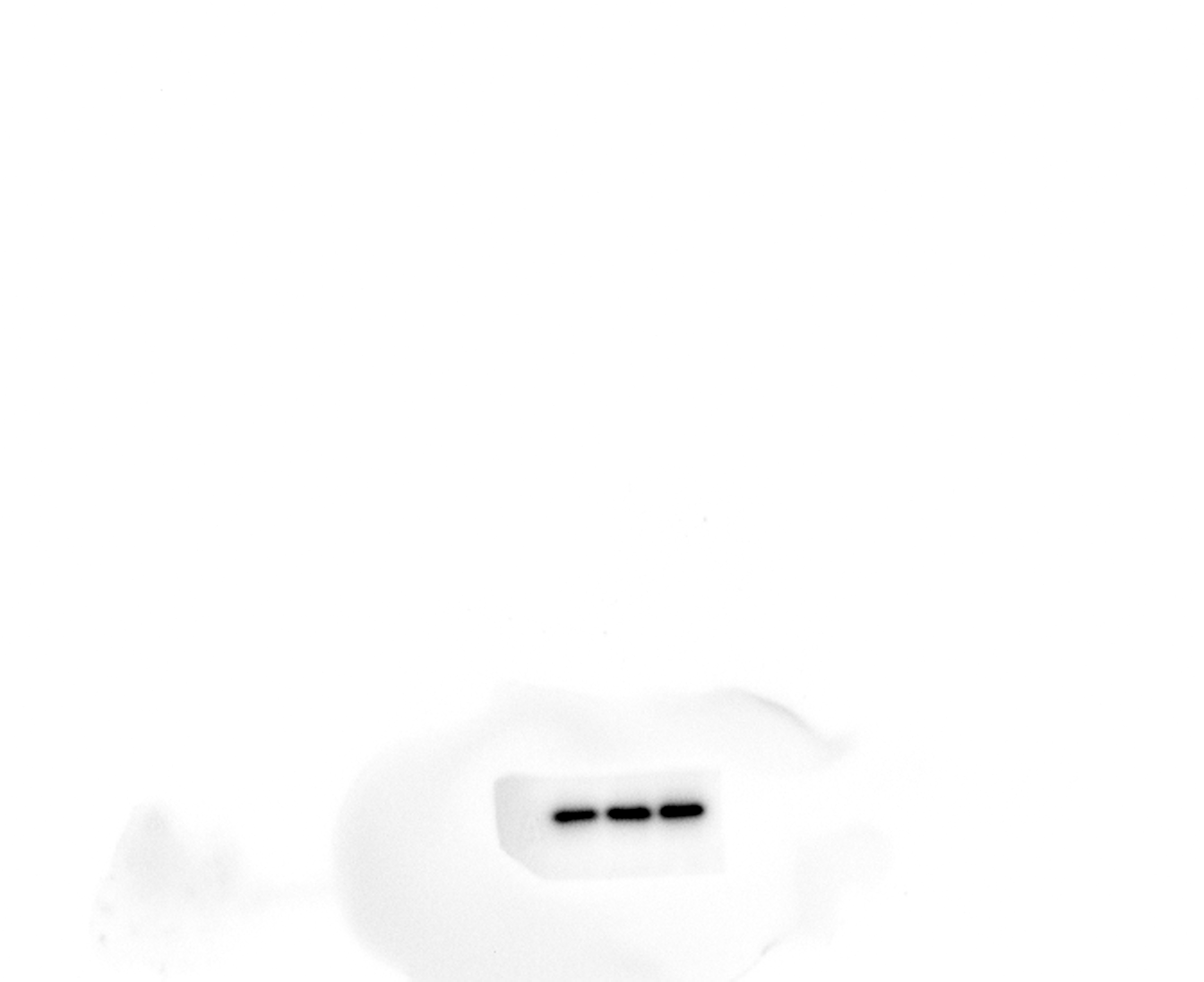

Supplement: Supplementary file 2 — Additional file 2: The raw experimental data related to this study. [file 12935_2022_2689_MOESM2_ESM.zip › WB/GAPDH/229-5.Tif]

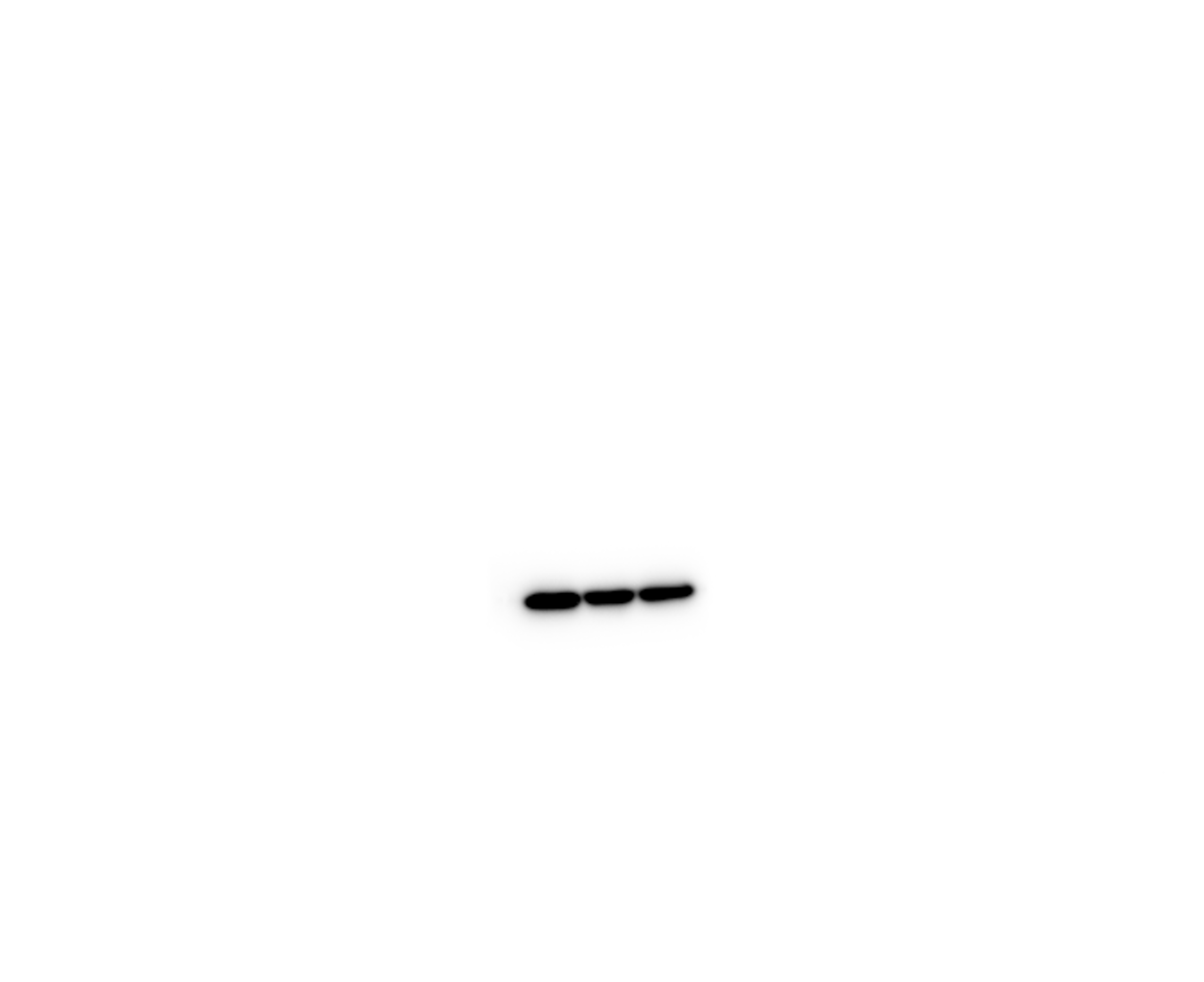

Supplement: Supplementary file 2 — Additional file 2: The raw experimental data related to this study. [file 12935_2022_2689_MOESM2_ESM.zip › WB/GAPDH/87-1.Tif]

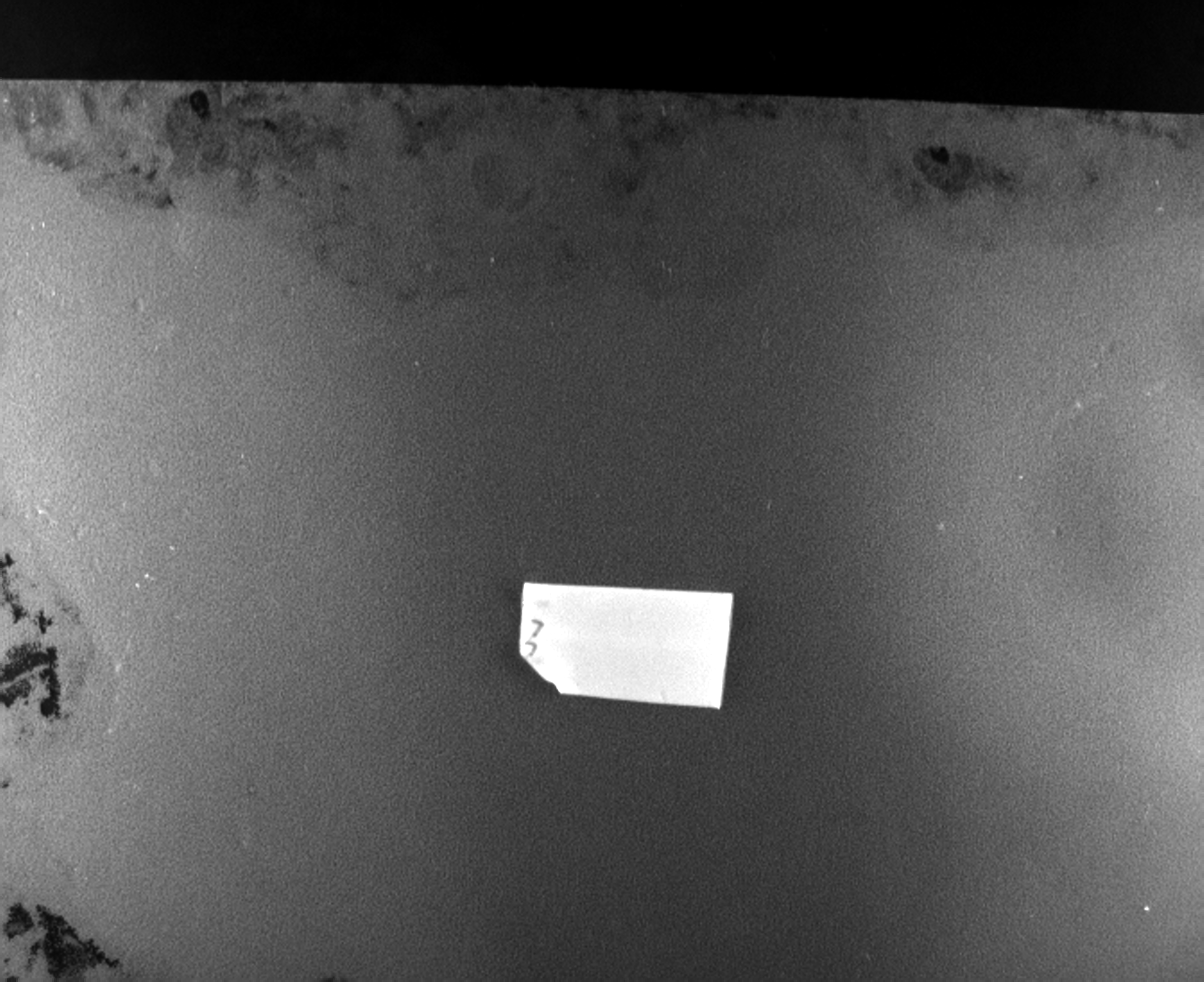

Supplement: Supplementary file 2 — Additional file 2: The raw experimental data related to this study. [file 12935_2022_2689_MOESM2_ESM.zip › WB/GAPDH/87-2-2.Tif]

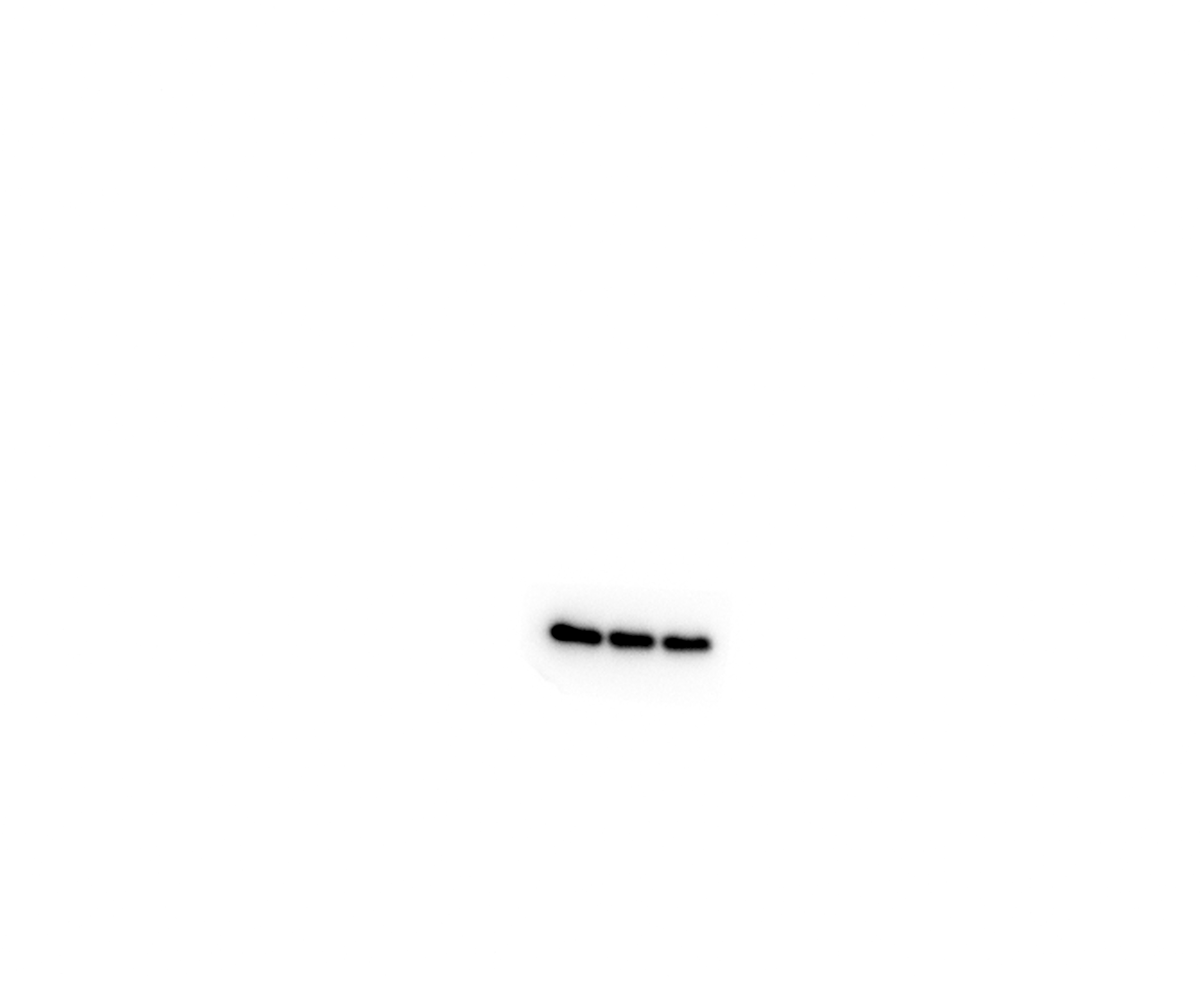

Supplement: Supplementary file 2 — Additional file 2: The raw experimental data related to this study. [file 12935_2022_2689_MOESM2_ESM.zip › WB/GAPDH/87-2.Tif]

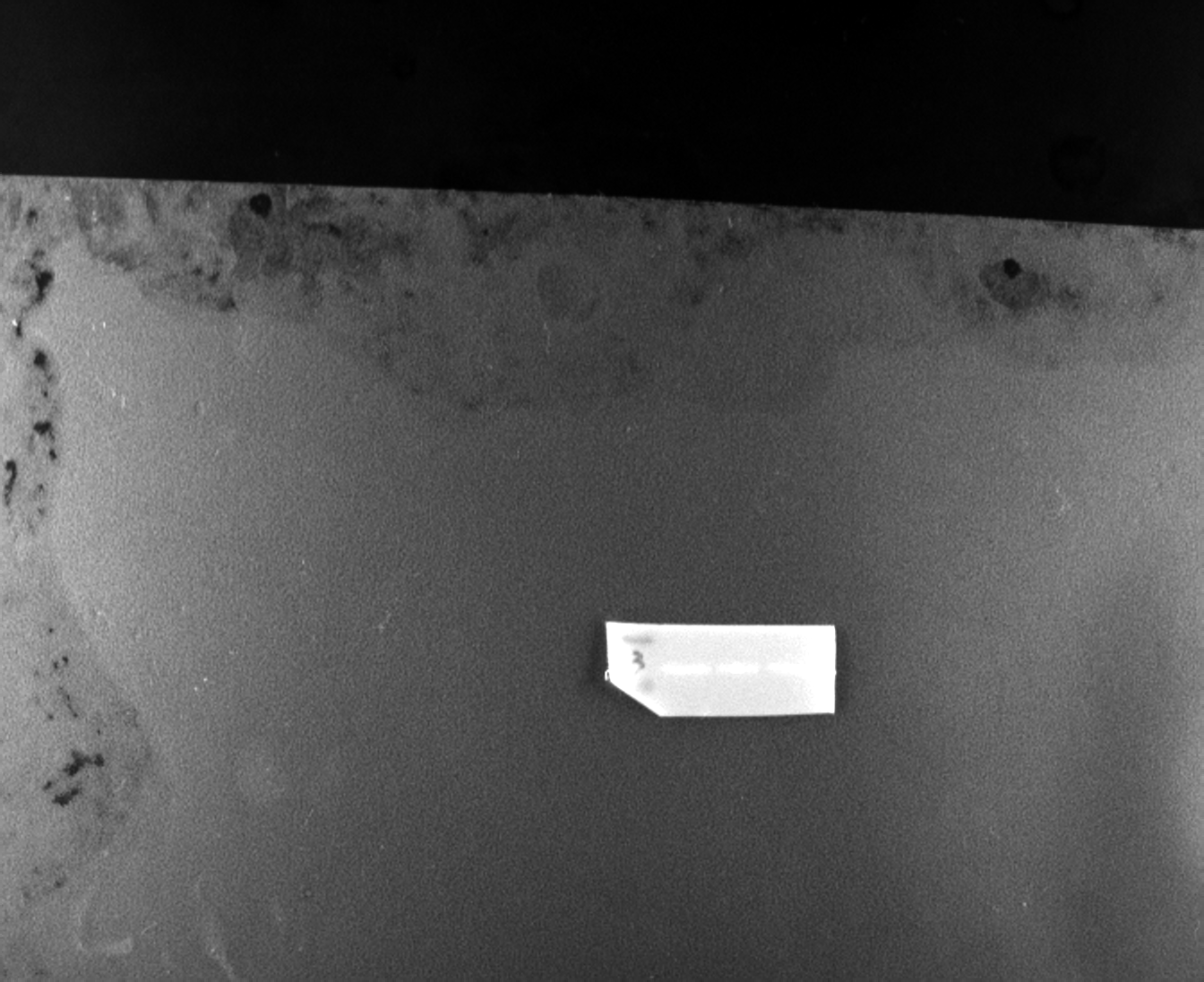

Supplement: Supplementary file 2 — Additional file 2: The raw experimental data related to this study. [file 12935_2022_2689_MOESM2_ESM.zip › WB/GAPDH/87-3-2.Tif]

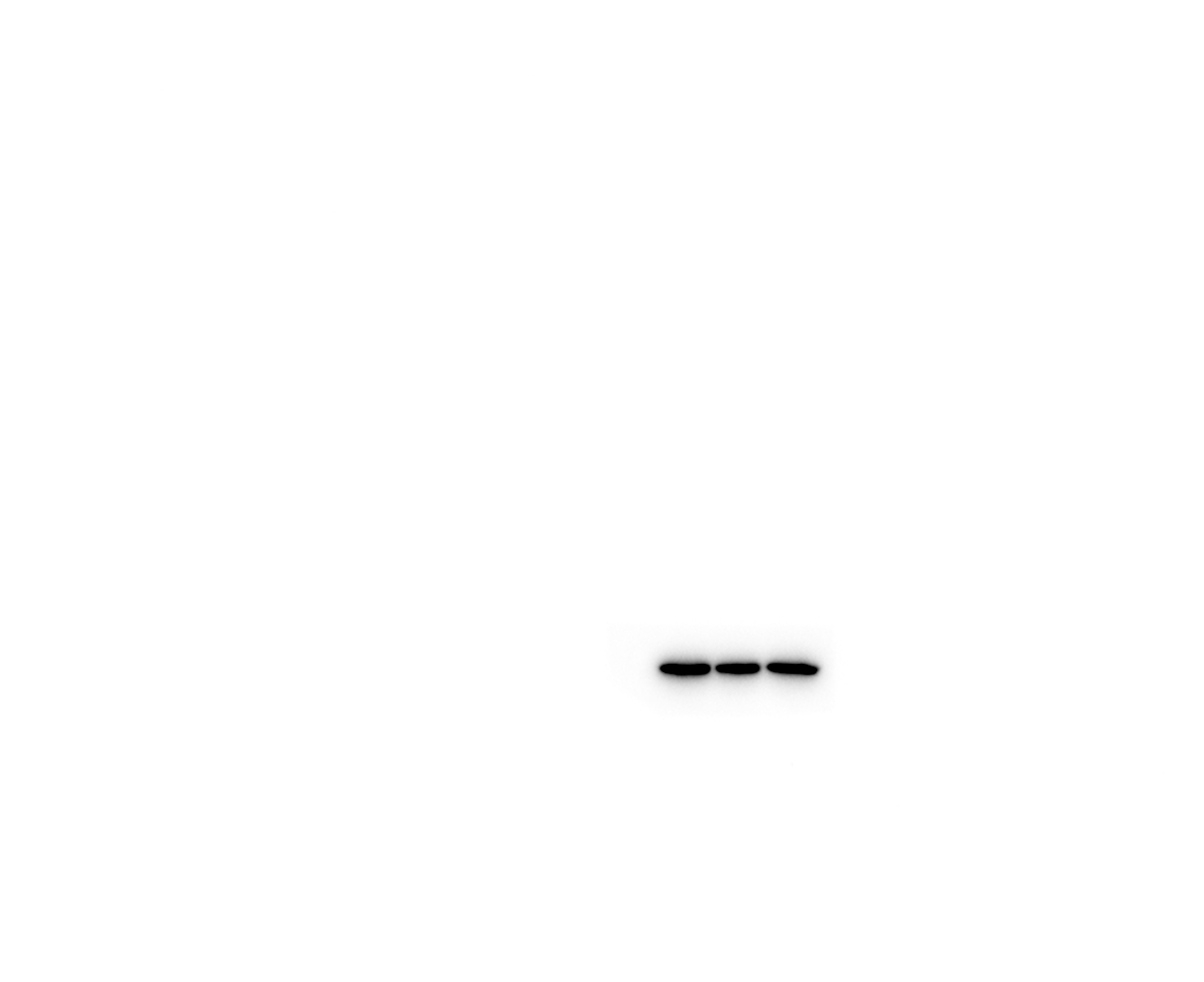

Supplement: Supplementary file 2 — Additional file 2: The raw experimental data related to this study. [file 12935_2022_2689_MOESM2_ESM.zip › WB/GAPDH/87-3.Tif]

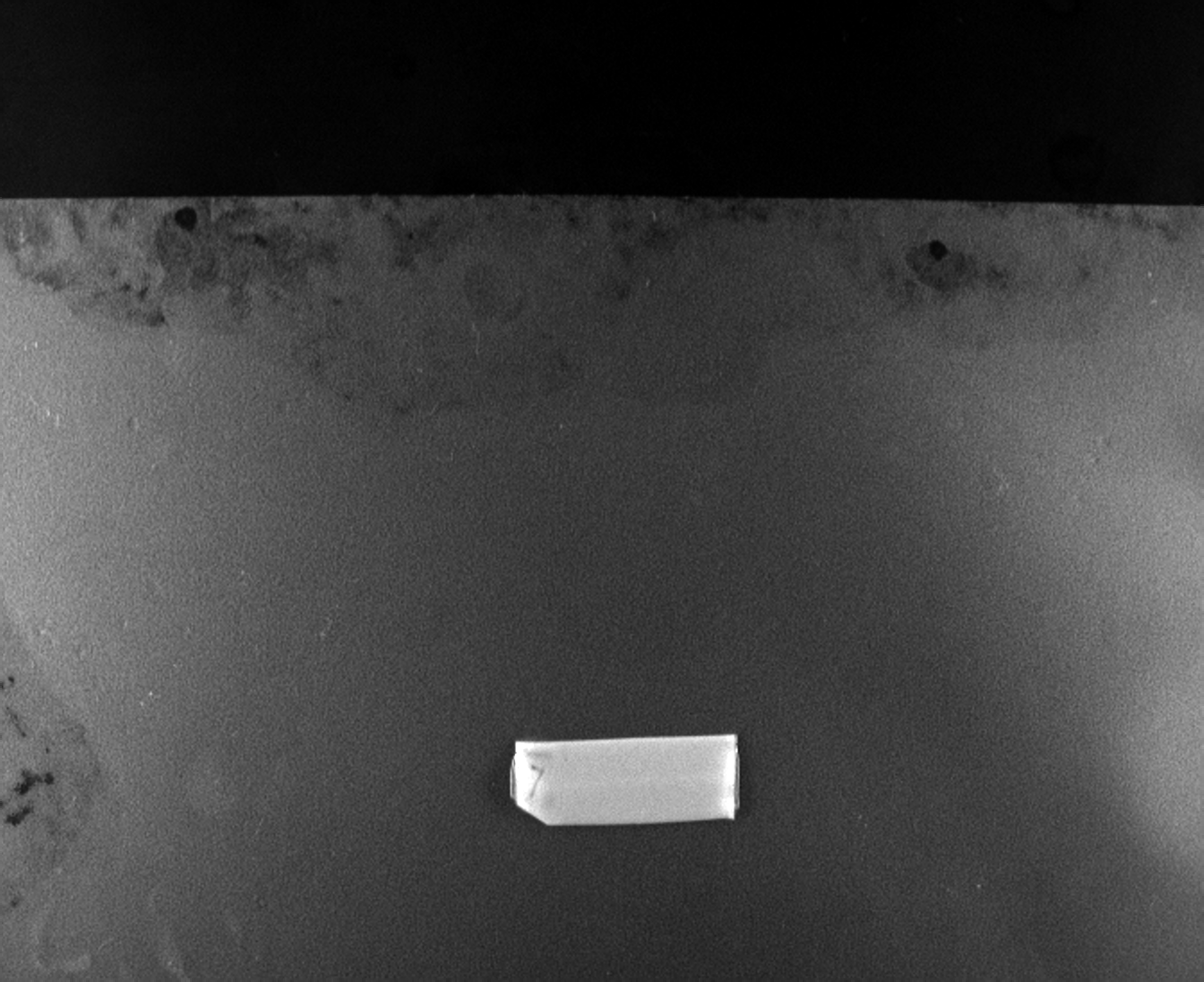

Supplement: Supplementary file 2 — Additional file 2: The raw experimental data related to this study. [file 12935_2022_2689_MOESM2_ESM.zip › WB/GAPDH/87-4-2.Tif]

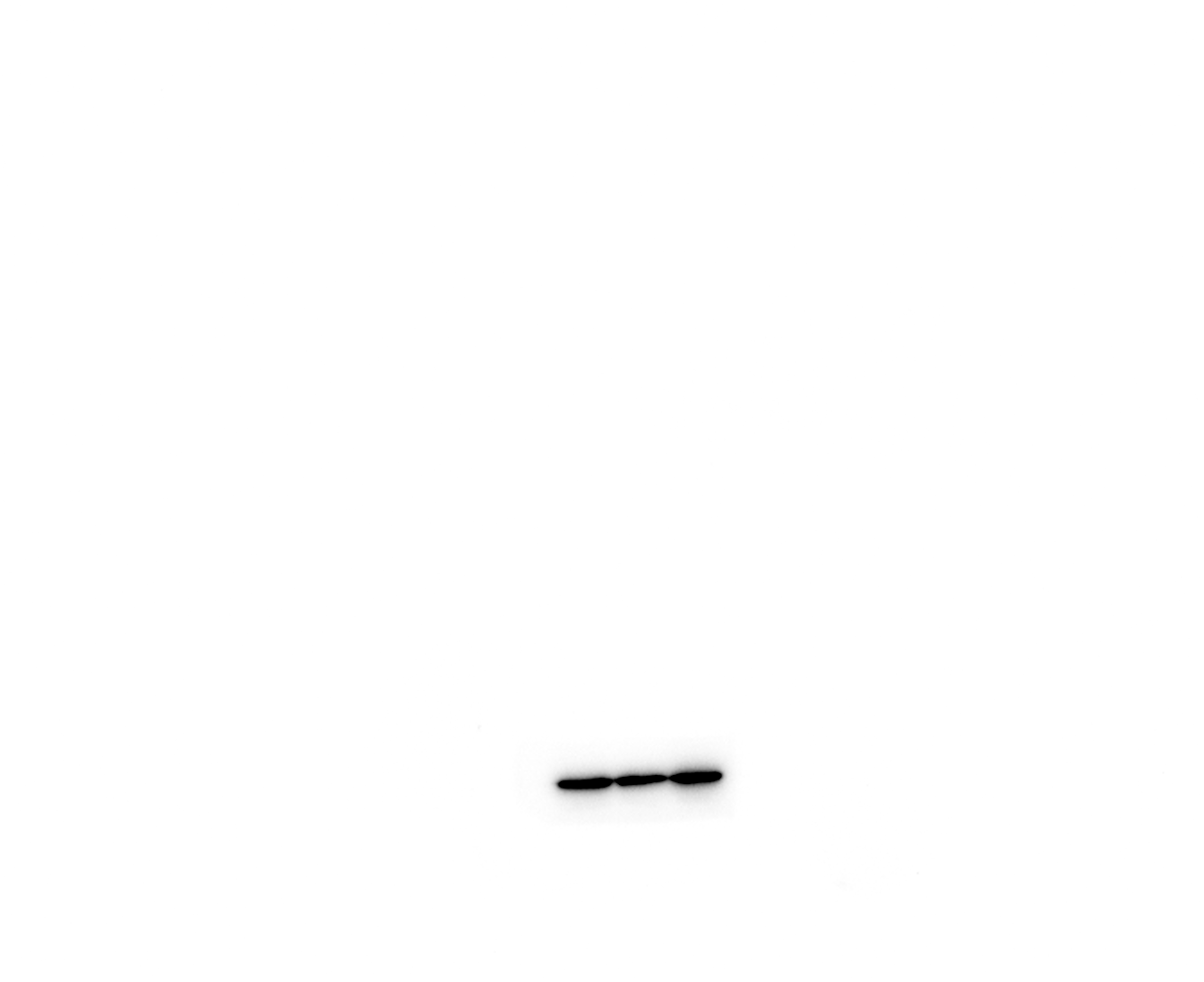

Supplement: Supplementary file 2 — Additional file 2: The raw experimental data related to this study. [file 12935_2022_2689_MOESM2_ESM.zip › WB/GAPDH/87-4.Tif]

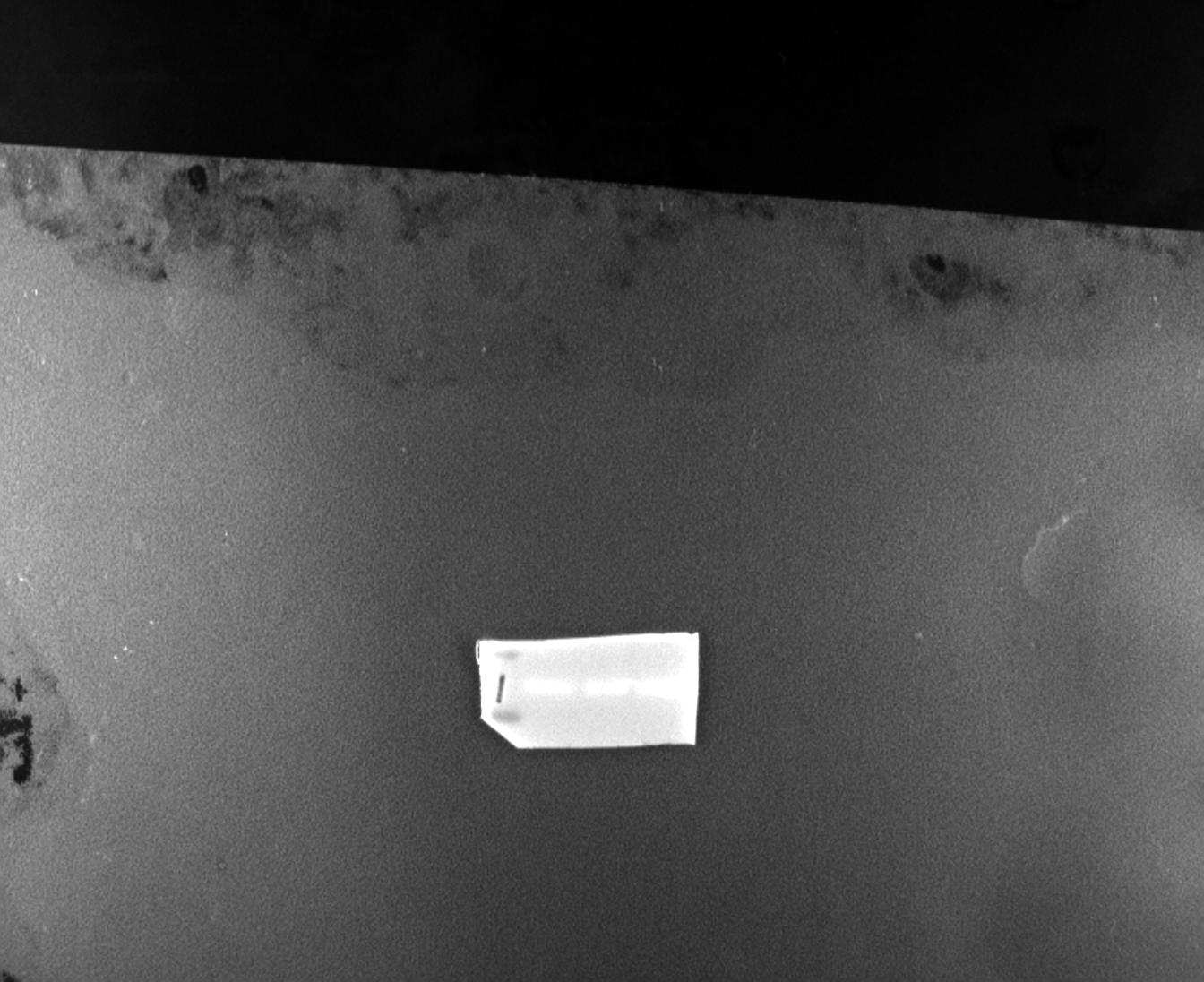

Supplement: Supplementary file 2 — Additional file 2: The raw experimental data related to this study. [file 12935_2022_2689_MOESM2_ESM.zip › WB/GAPDH/87-5-2.Tif]

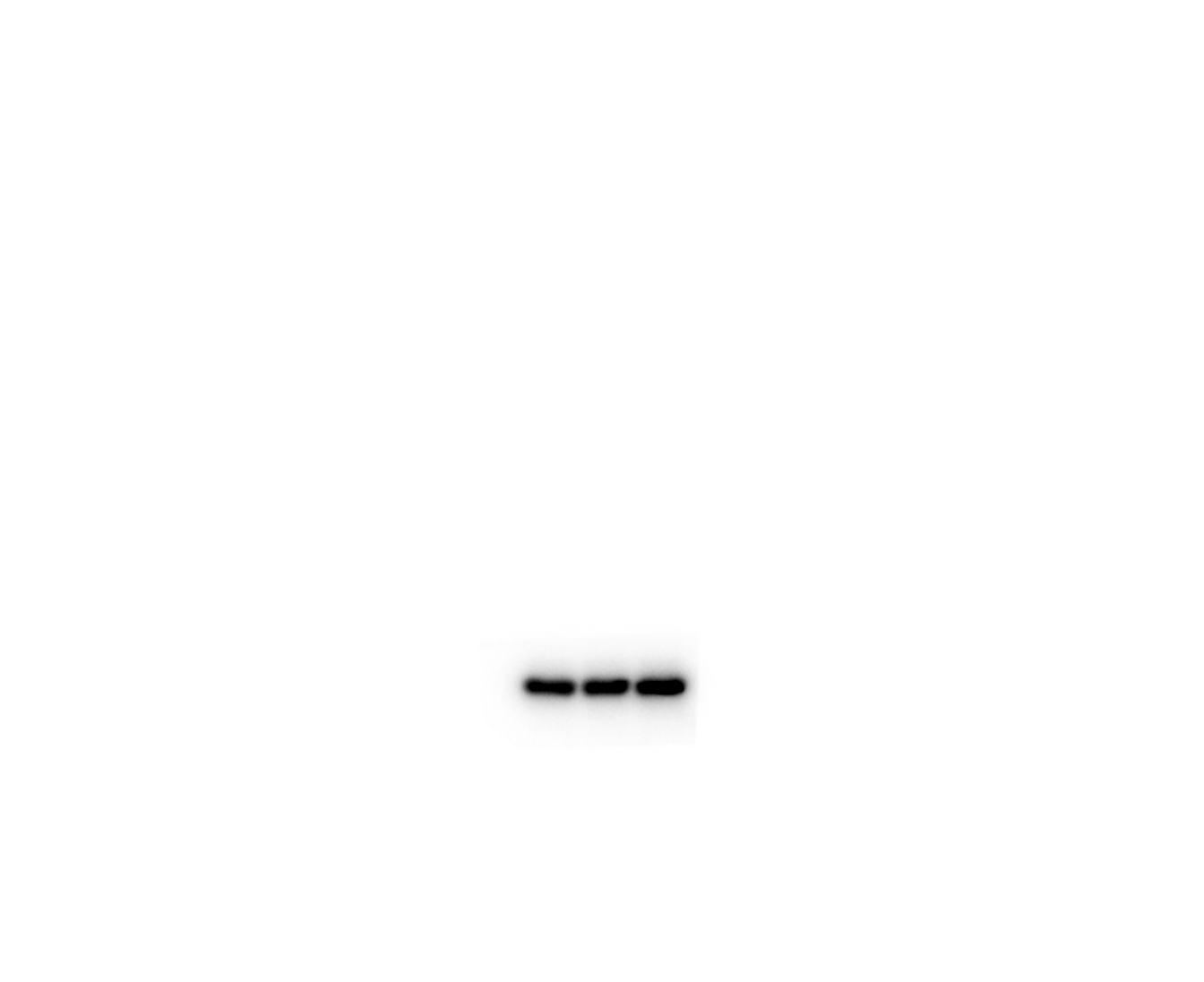

Supplement: Supplementary file 2 — Additional file 2: The raw experimental data related to this study. [file 12935_2022_2689_MOESM2_ESM.zip › WB/GAPDH/87-5.Tif]

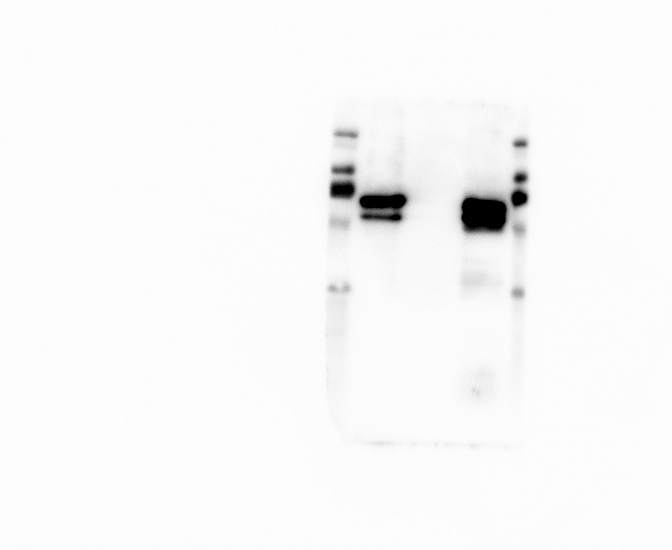

Supplement: Supplementary file 2 — Additional file 2: The raw experimental data related to this study. [file 12935_2022_2689_MOESM2_ESM.zip › WB/IP-AKT1/229-AKT1-1-1.tif]

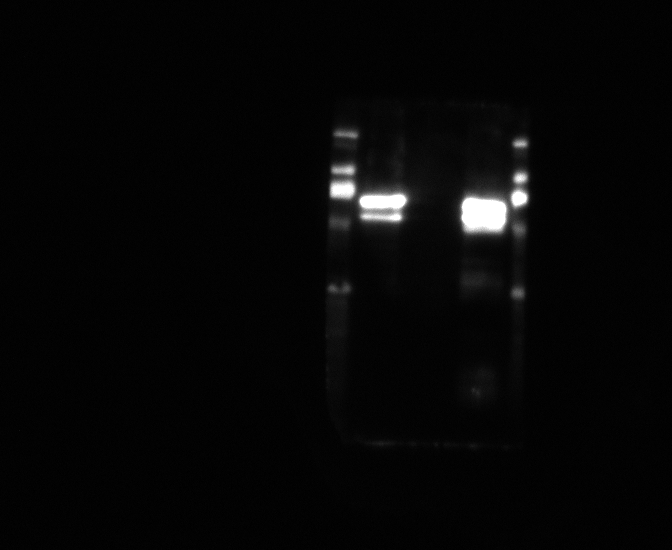

Supplement: Supplementary file 2 — Additional file 2: The raw experimental data related to this study. [file 12935_2022_2689_MOESM2_ESM.zip › WB/IP-AKT1/229-AKT1-1-2.tif]

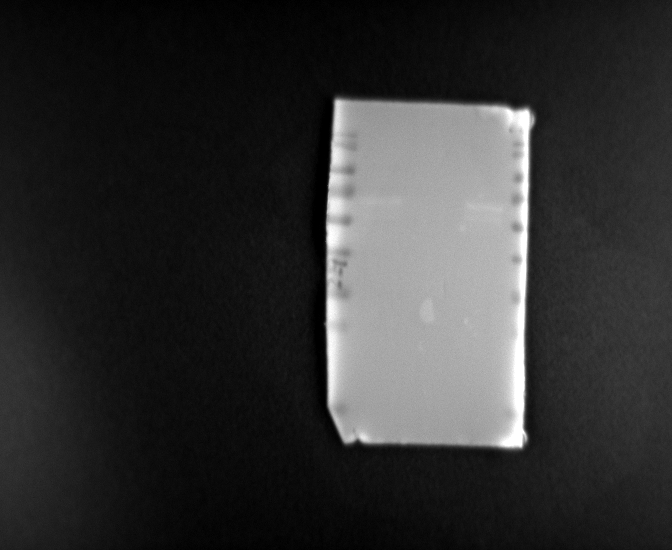

Supplement: Supplementary file 2 — Additional file 2: The raw experimental data related to this study. [file 12935_2022_2689_MOESM2_ESM.zip › WB/IP-AKT1/229-AKT1-1-3.tif]

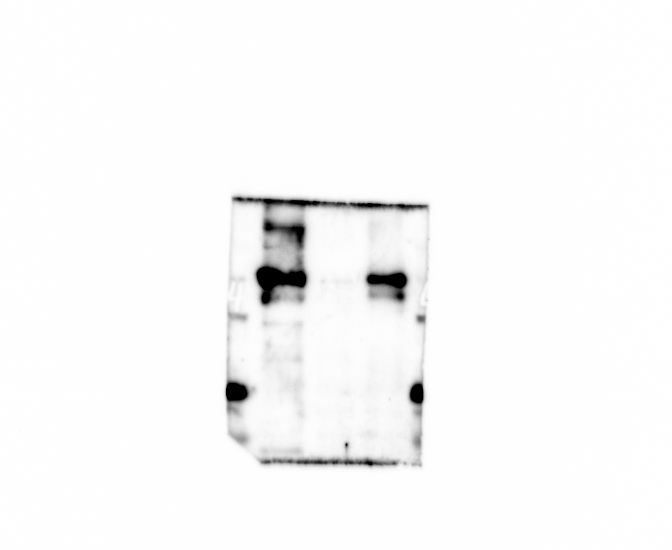

Supplement: Supplementary file 2 — Additional file 2: The raw experimental data related to this study. [file 12935_2022_2689_MOESM2_ESM.zip › WB/IP-AKT1/87-AKT-2-1.tif]

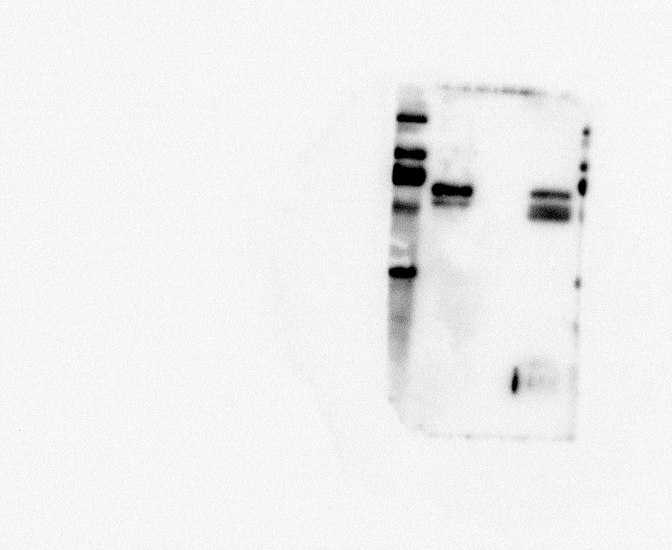

Supplement: Supplementary file 2 — Additional file 2: The raw experimental data related to this study. [file 12935_2022_2689_MOESM2_ESM.zip › WB/IP-AKT1/87-AKT1-1-1.tif]

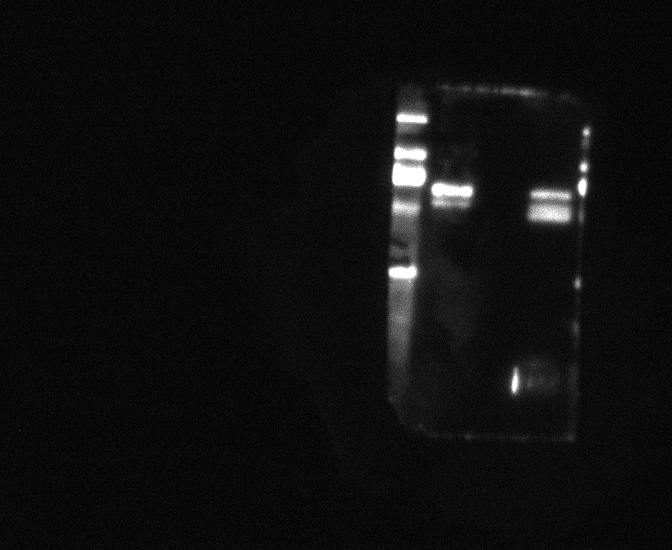

Supplement: Supplementary file 2 — Additional file 2: The raw experimental data related to this study. [file 12935_2022_2689_MOESM2_ESM.zip › WB/IP-AKT1/87-AKT1-1-2.tif]

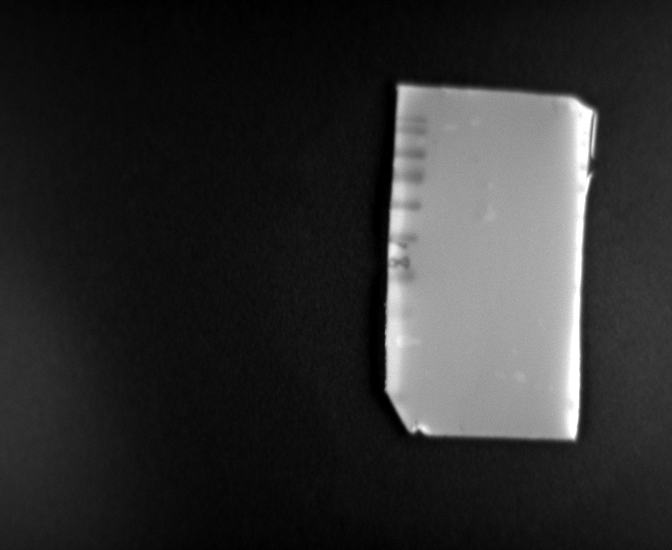

Supplement: Supplementary file 2 — Additional file 2: The raw experimental data related to this study. [file 12935_2022_2689_MOESM2_ESM.zip › WB/IP-AKT1/87-AKT1-1-3.tif]

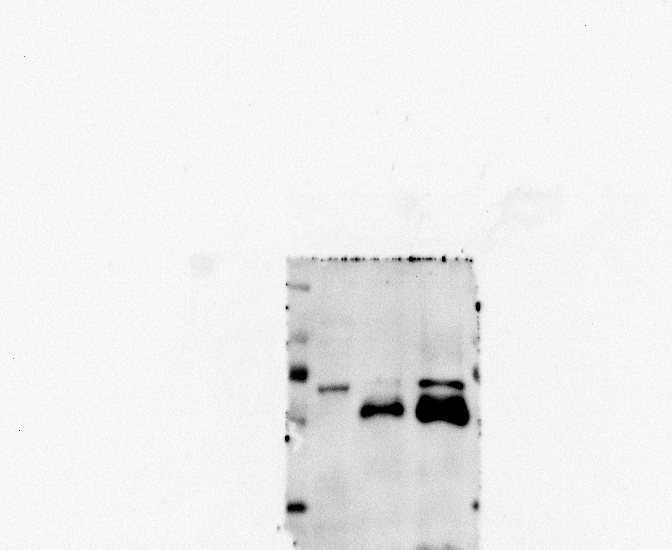

Supplement: Supplementary file 2 — Additional file 2: The raw experimental data related to this study. [file 12935_2022_2689_MOESM2_ESM.zip › WB/IP-AKT1/87-AKT1-3-1.tif]

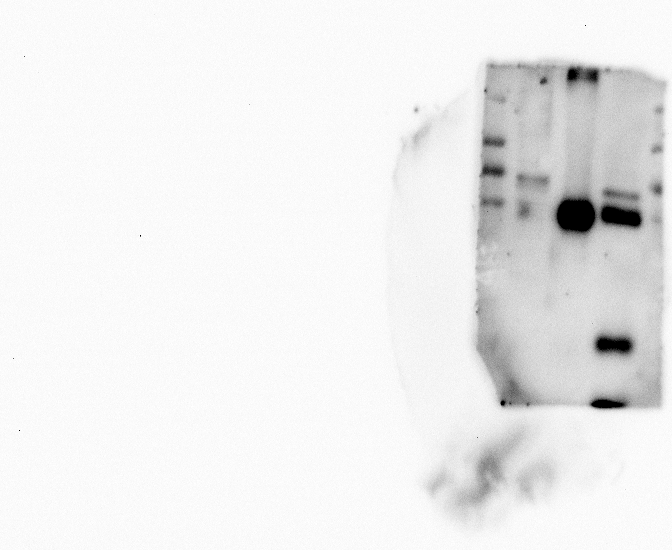

Supplement: Supplementary file 2 — Additional file 2: The raw experimental data related to this study. [file 12935_2022_2689_MOESM2_ESM.zip › WB/IP-PAK1/229-AKT-2-1.tif]

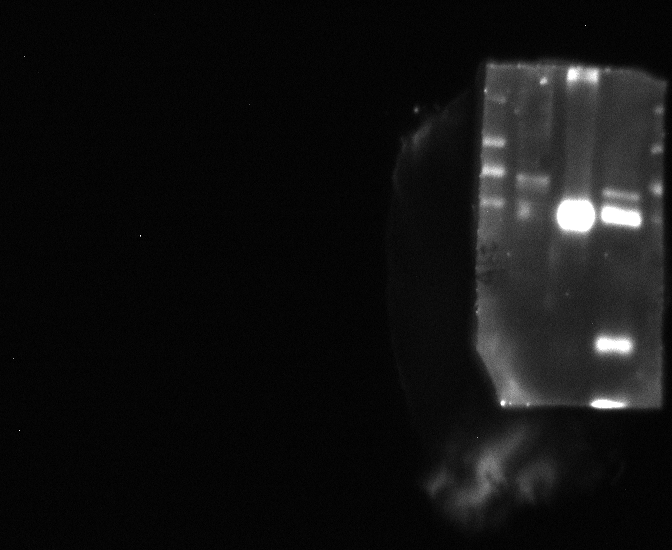

Supplement: Supplementary file 2 — Additional file 2: The raw experimental data related to this study. [file 12935_2022_2689_MOESM2_ESM.zip › WB/IP-PAK1/229-AKT-2-2.tif]

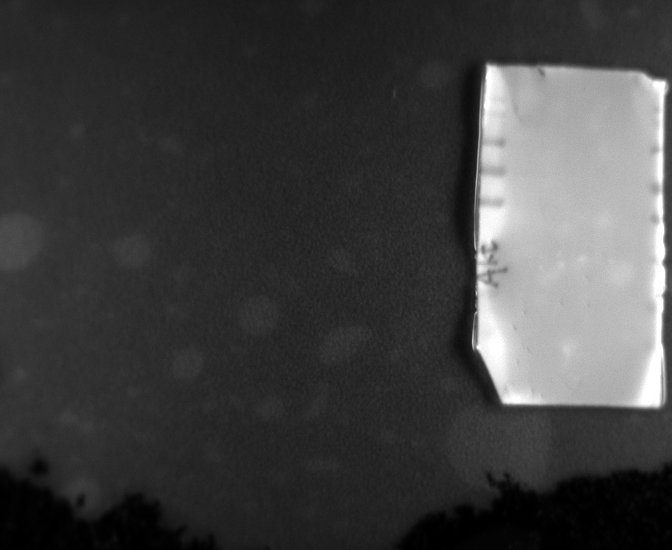

Supplement: Supplementary file 2 — Additional file 2: The raw experimental data related to this study. [file 12935_2022_2689_MOESM2_ESM.zip › WB/IP-PAK1/229-AKT-2-3.tif]

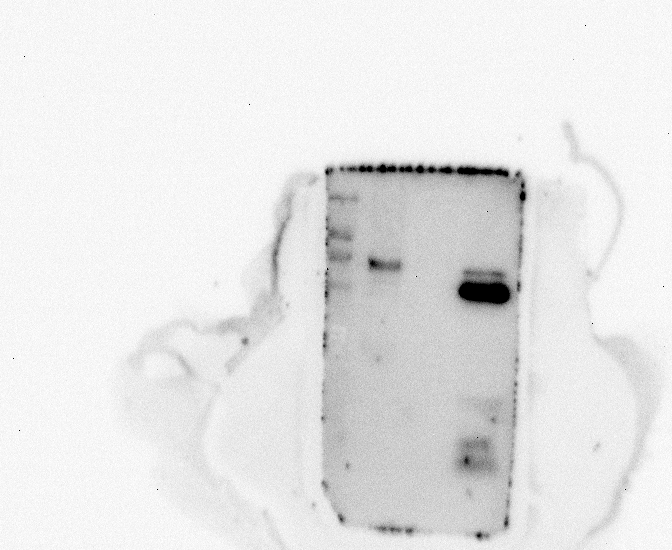

Supplement: Supplementary file 2 — Additional file 2: The raw experimental data related to this study. [file 12935_2022_2689_MOESM2_ESM.zip › WB/IP-PAK1/229-AKT1-1.tif]

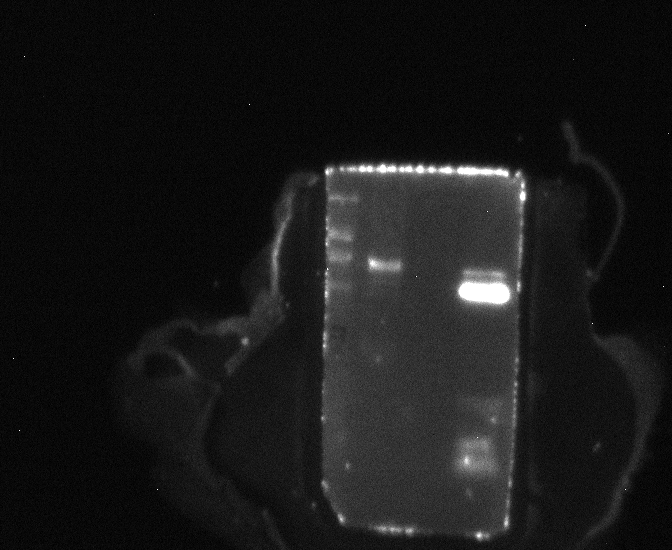

Supplement: Supplementary file 2 — Additional file 2: The raw experimental data related to this study. [file 12935_2022_2689_MOESM2_ESM.zip › WB/IP-PAK1/229-AKT1-2.tif]

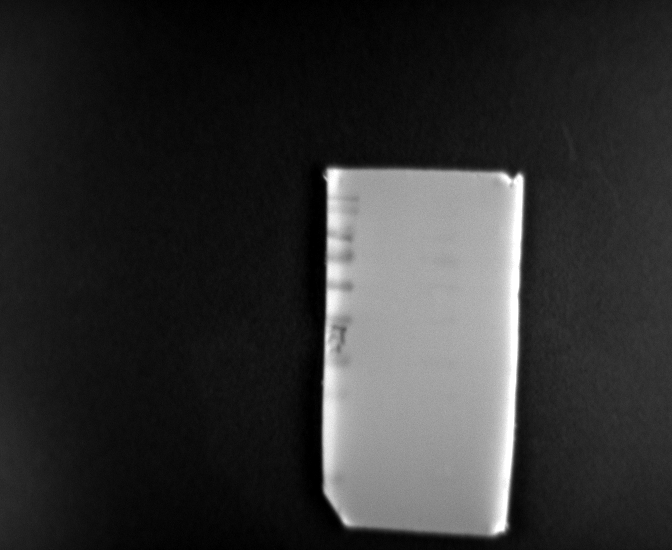

Supplement: Supplementary file 2 — Additional file 2: The raw experimental data related to this study. [file 12935_2022_2689_MOESM2_ESM.zip › WB/IP-PAK1/229-AKT1-3.tif]

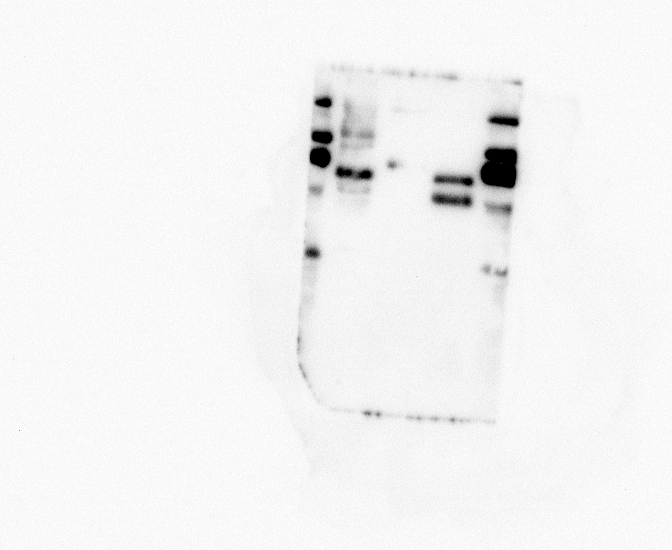

Supplement: Supplementary file 2 — Additional file 2: The raw experimental data related to this study. [file 12935_2022_2689_MOESM2_ESM.zip › WB/IP-PAK1/87-AKT1-1-1.tif]

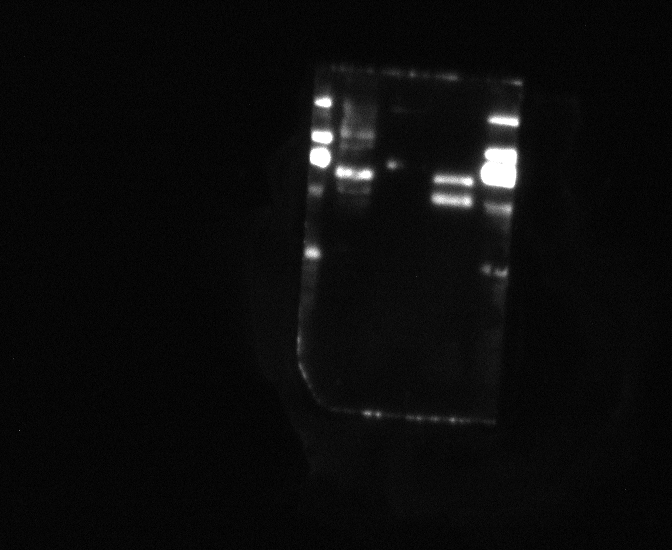

Supplement: Supplementary file 2 — Additional file 2: The raw experimental data related to this study. [file 12935_2022_2689_MOESM2_ESM.zip › WB/IP-PAK1/87-AKT1-1-2.tif]

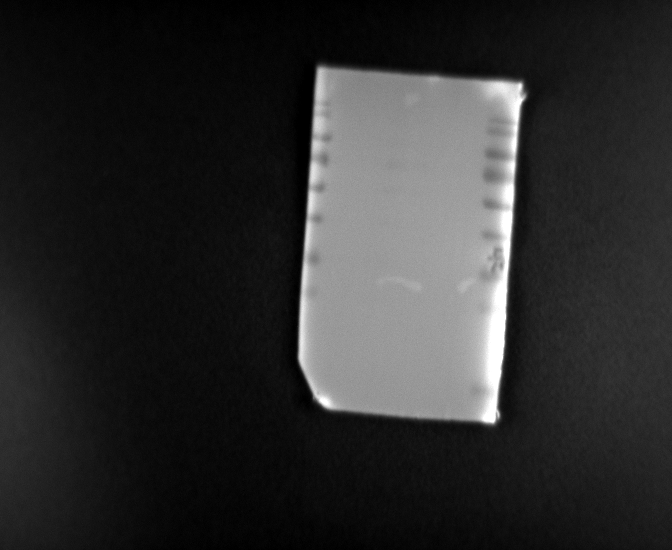

Supplement: Supplementary file 2 — Additional file 2: The raw experimental data related to this study. [file 12935_2022_2689_MOESM2_ESM.zip › WB/IP-PAK1/87-AKT1-1-3.tif]

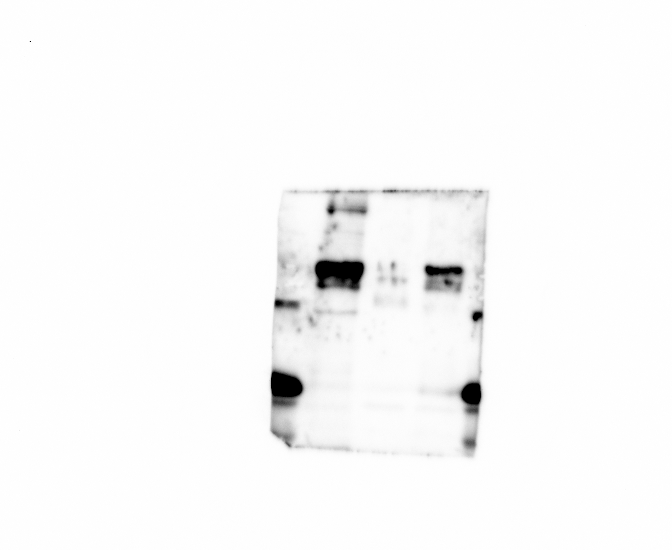

Supplement: Supplementary file 2 — Additional file 2: The raw experimental data related to this study. [file 12935_2022_2689_MOESM2_ESM.zip › WB/IP-PAK1/87-AKT1-2-1.tif]

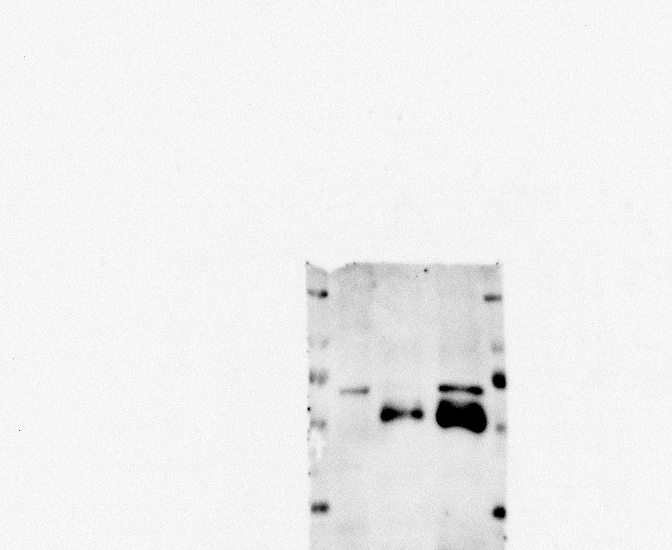

Supplement: Supplementary file 2 — Additional file 2: The raw experimental data related to this study. [file 12935_2022_2689_MOESM2_ESM.zip › WB/IP-PAK1/87-AKT1-3-1.tif]

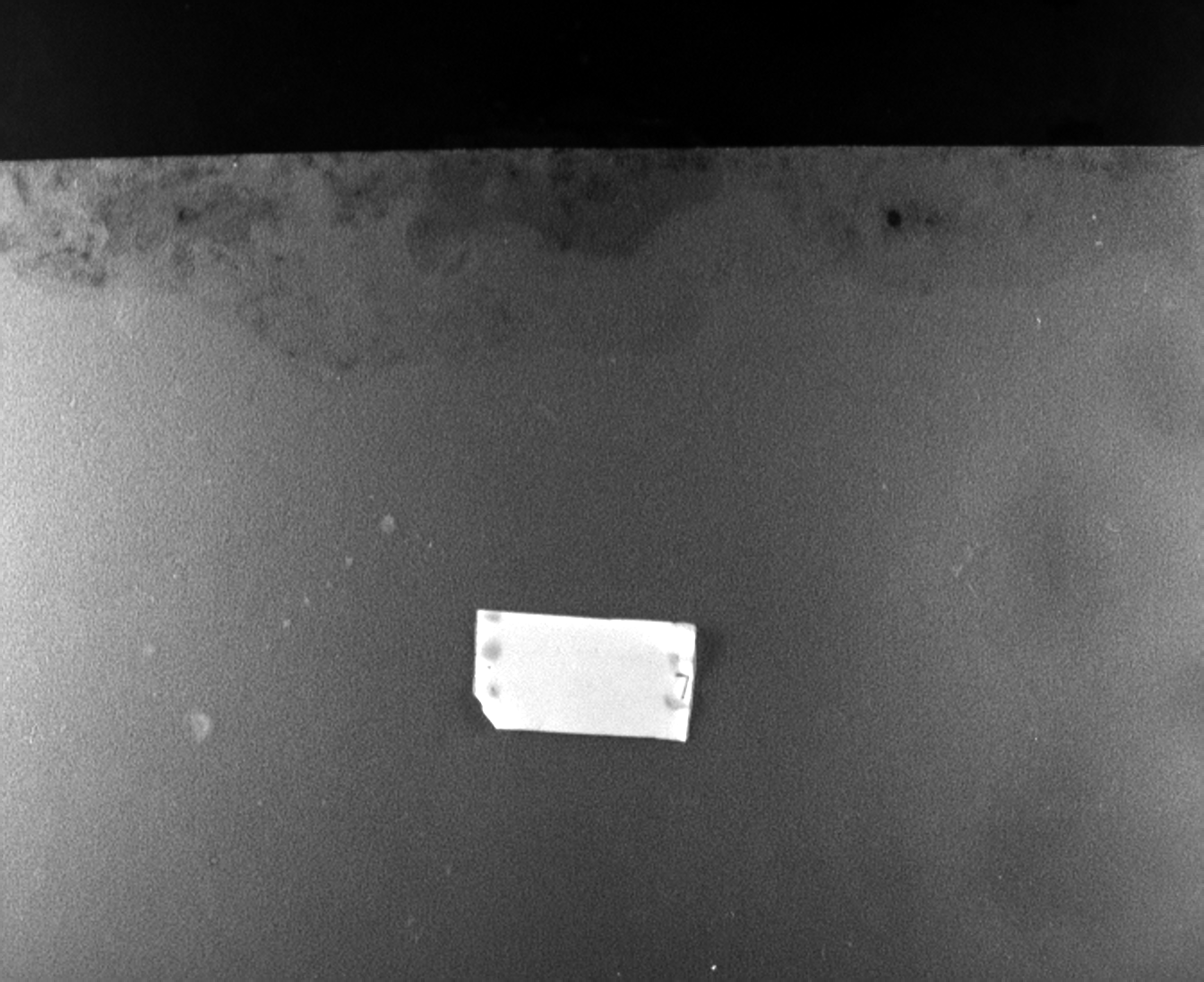

Supplement: Supplementary file 2 — Additional file 2: The raw experimental data related to this study. [file 12935_2022_2689_MOESM2_ESM.zip › WB/PAK1/229-1-2.Tif]

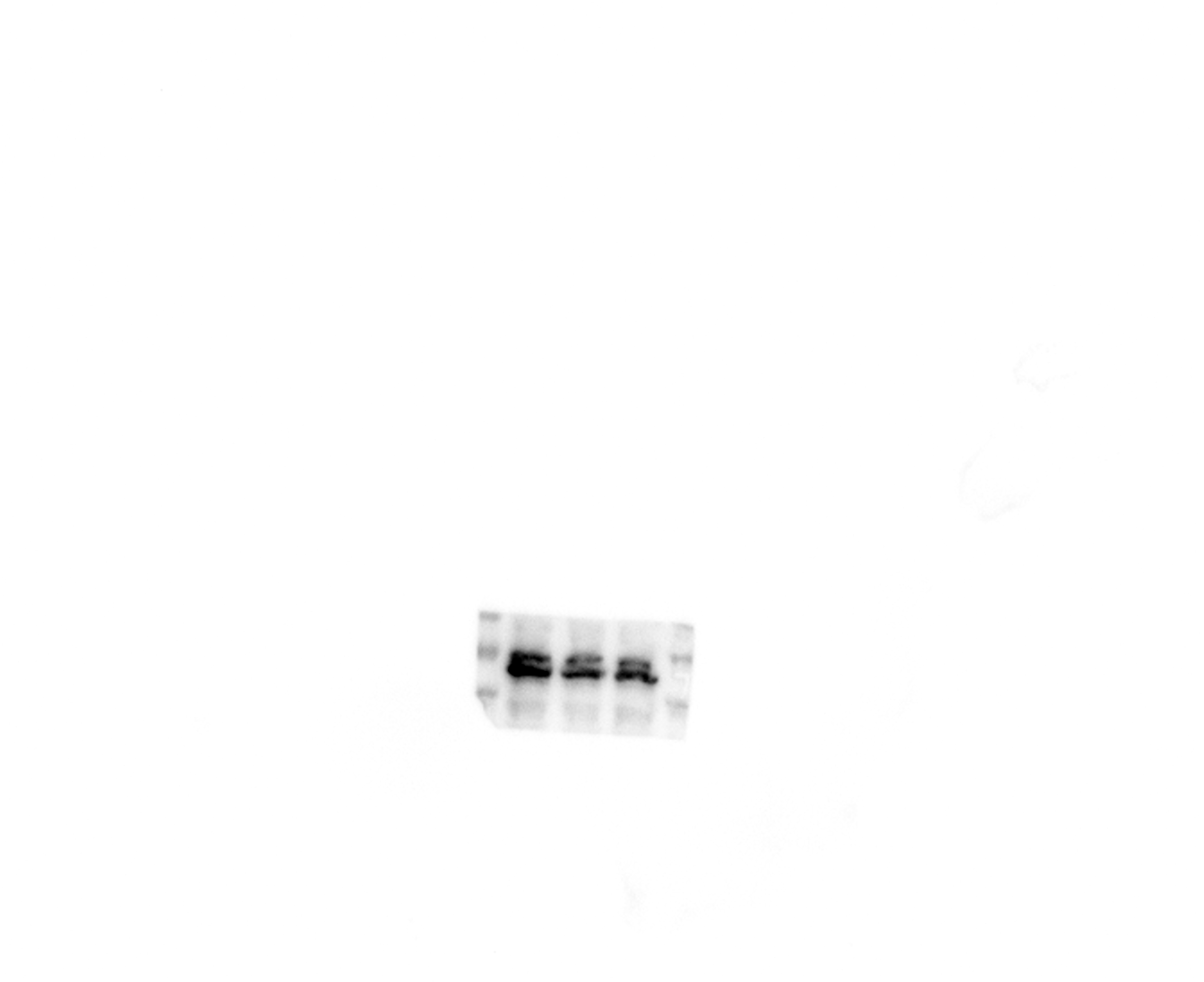

Supplement: Supplementary file 2 — Additional file 2: The raw experimental data related to this study. [file 12935_2022_2689_MOESM2_ESM.zip › WB/PAK1/229-1.Tif]

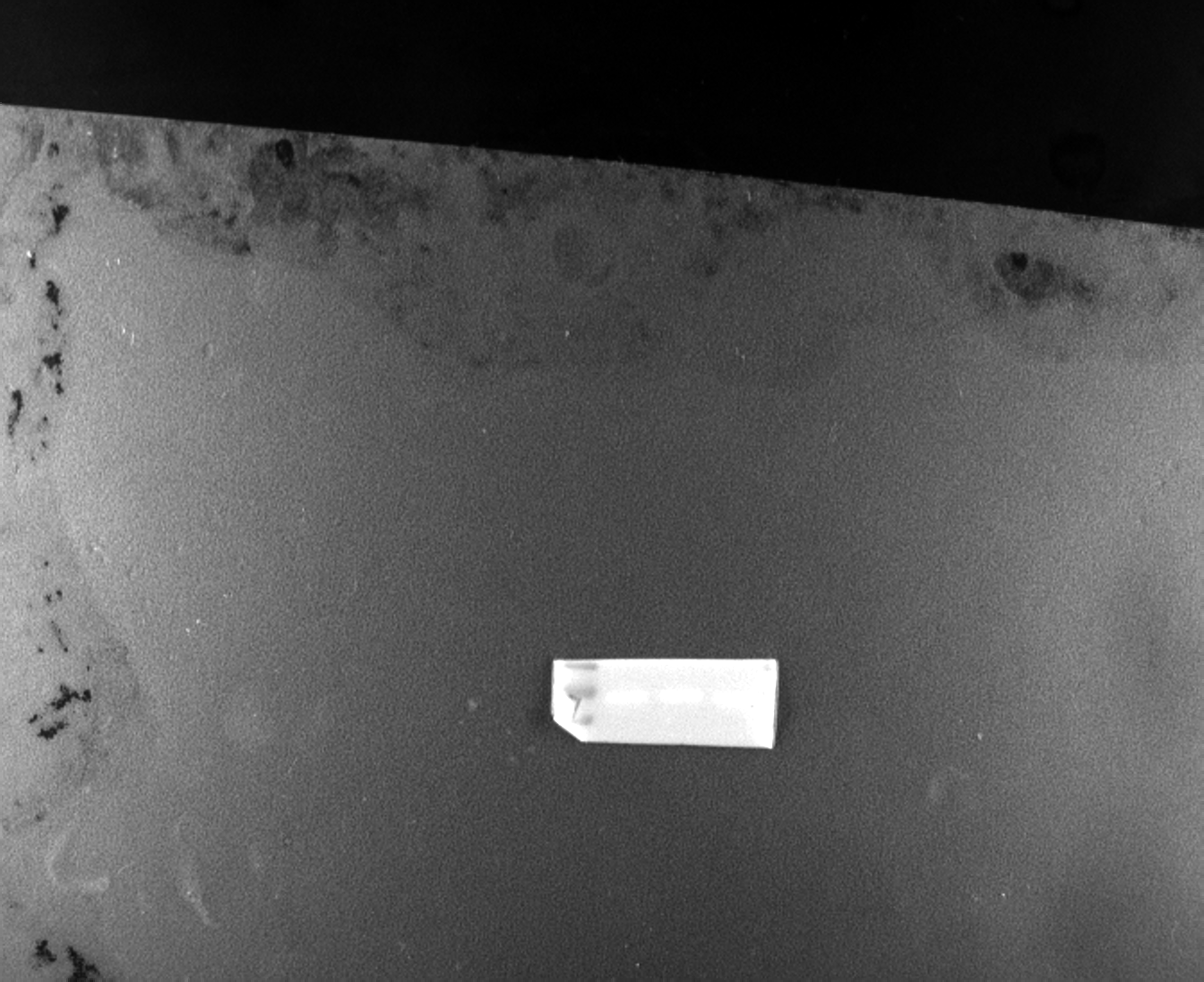

Supplement: Supplementary file 2 — Additional file 2: The raw experimental data related to this study. [file 12935_2022_2689_MOESM2_ESM.zip › WB/PAK1/229-2-2.Tif]

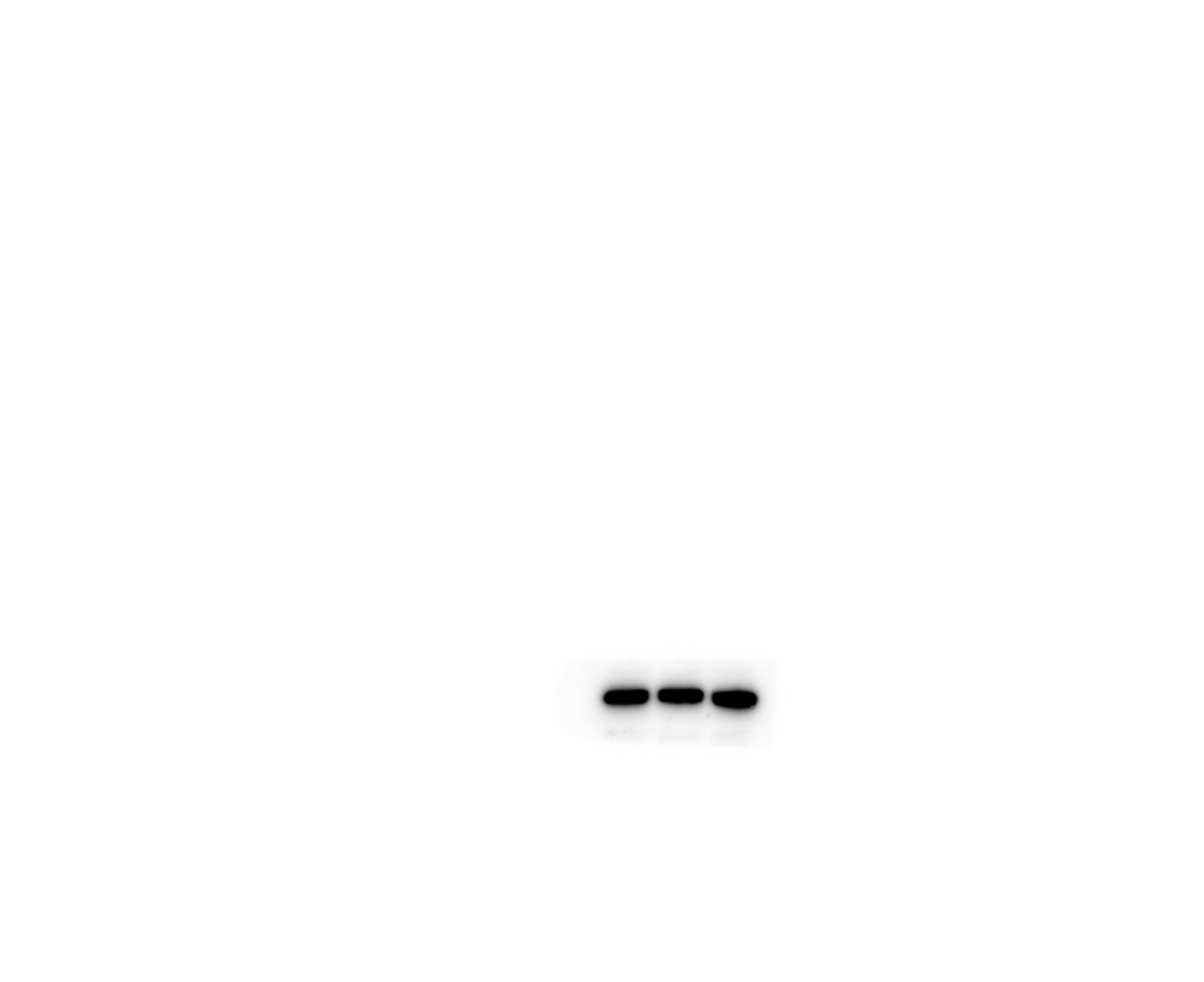

Supplement: Supplementary file 2 — Additional file 2: The raw experimental data related to this study. [file 12935_2022_2689_MOESM2_ESM.zip › WB/PAK1/229-2.Tif]

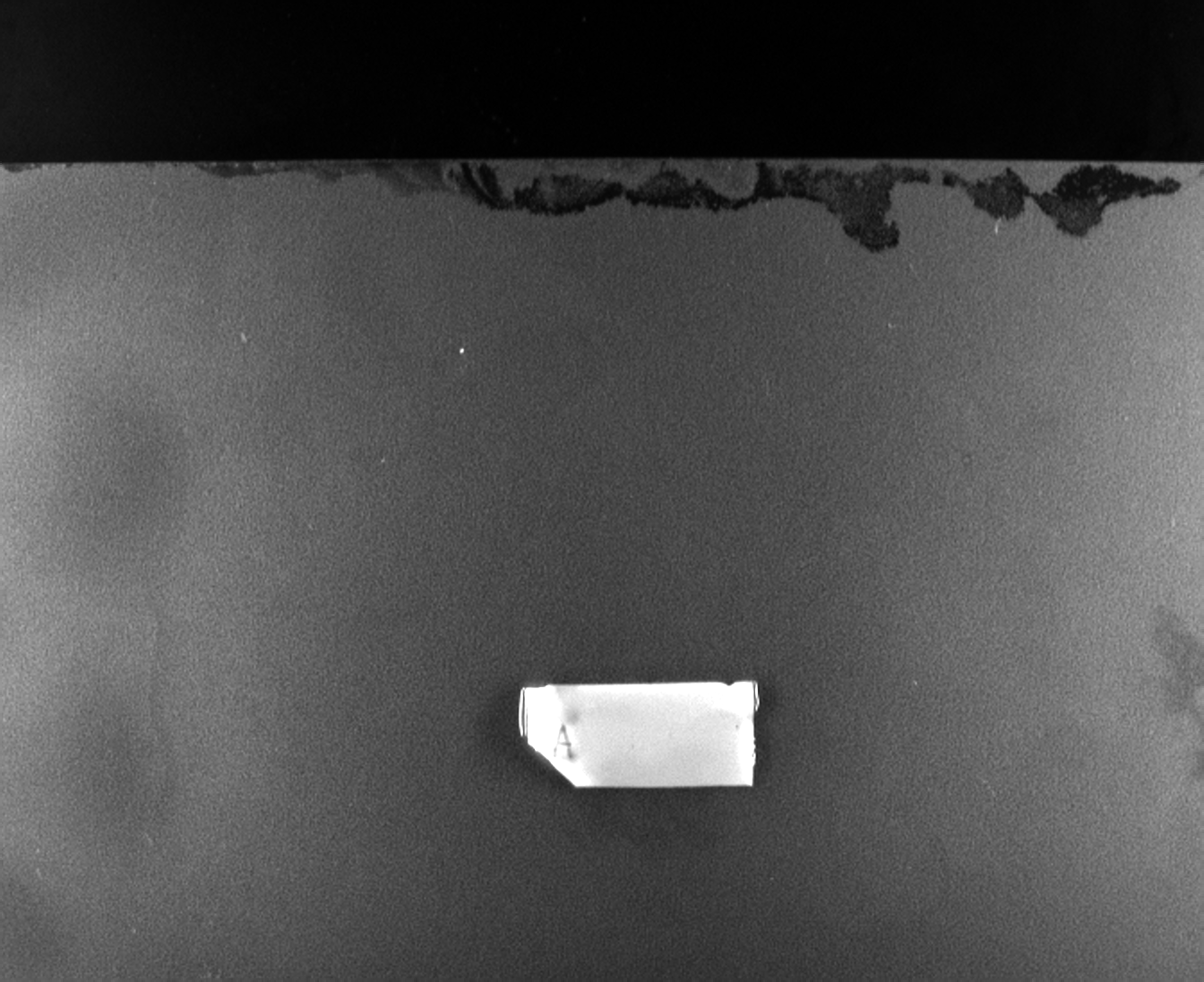

Supplement: Supplementary file 2 — Additional file 2: The raw experimental data related to this study. [file 12935_2022_2689_MOESM2_ESM.zip › WB/PAK1/229-3-2.Tif]

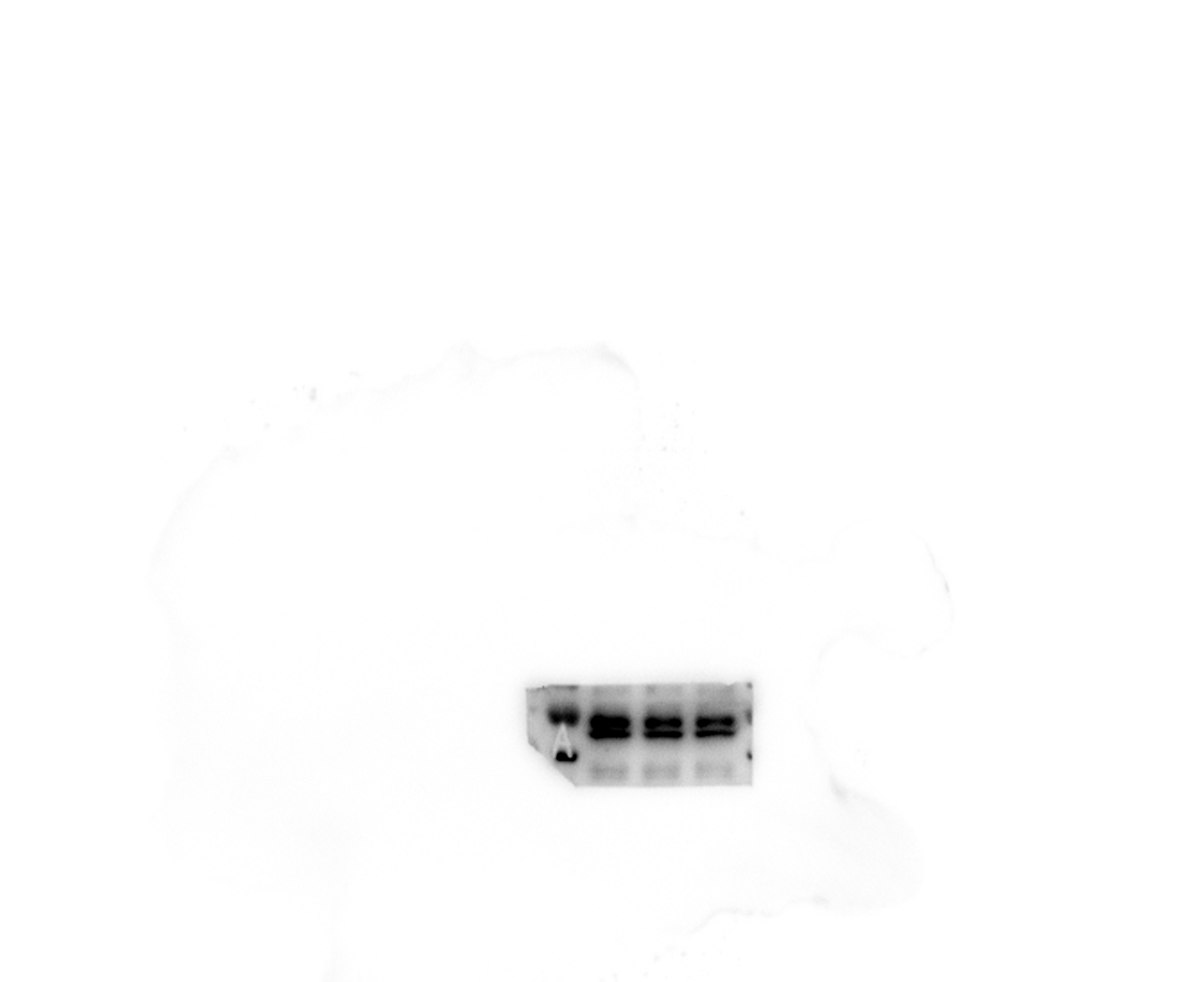

Supplement: Supplementary file 2 — Additional file 2: The raw experimental data related to this study. [file 12935_2022_2689_MOESM2_ESM.zip › WB/PAK1/229-3.Tif]

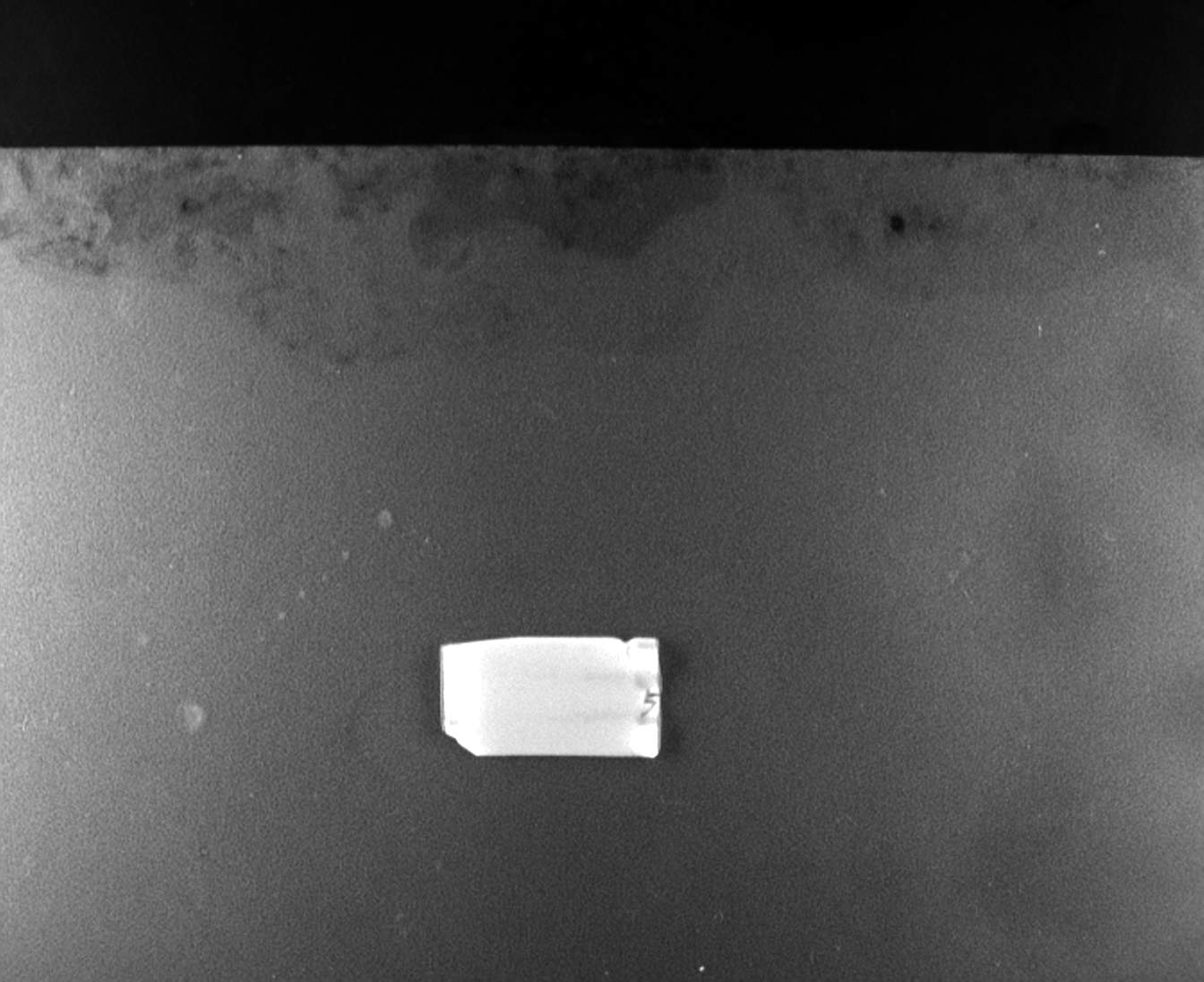

Supplement: Supplementary file 2 — Additional file 2: The raw experimental data related to this study. [file 12935_2022_2689_MOESM2_ESM.zip › WB/PAK1/87-1-2.Tif]

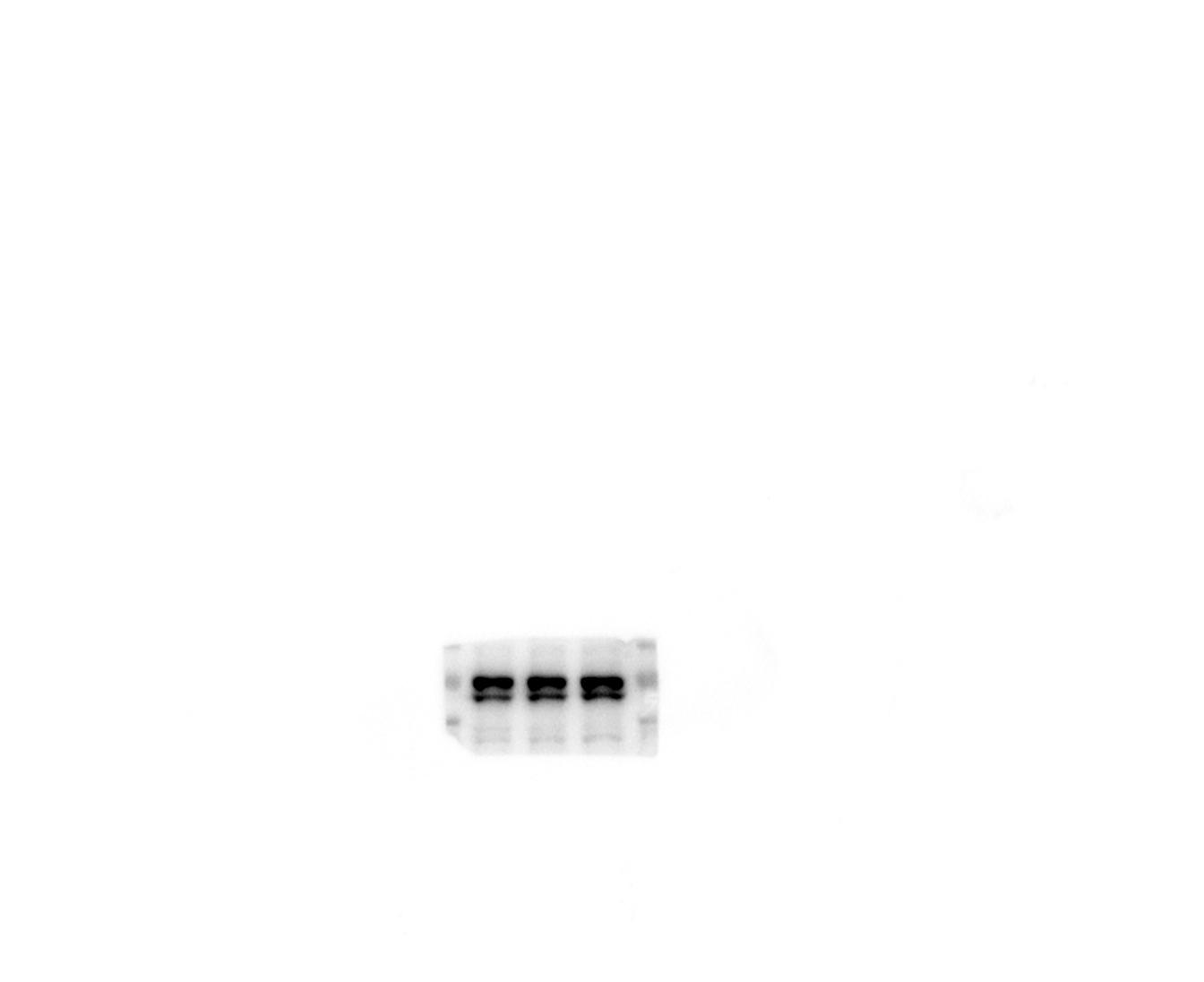

Supplement: Supplementary file 2 — Additional file 2: The raw experimental data related to this study. [file 12935_2022_2689_MOESM2_ESM.zip › WB/PAK1/87-1.Tif]

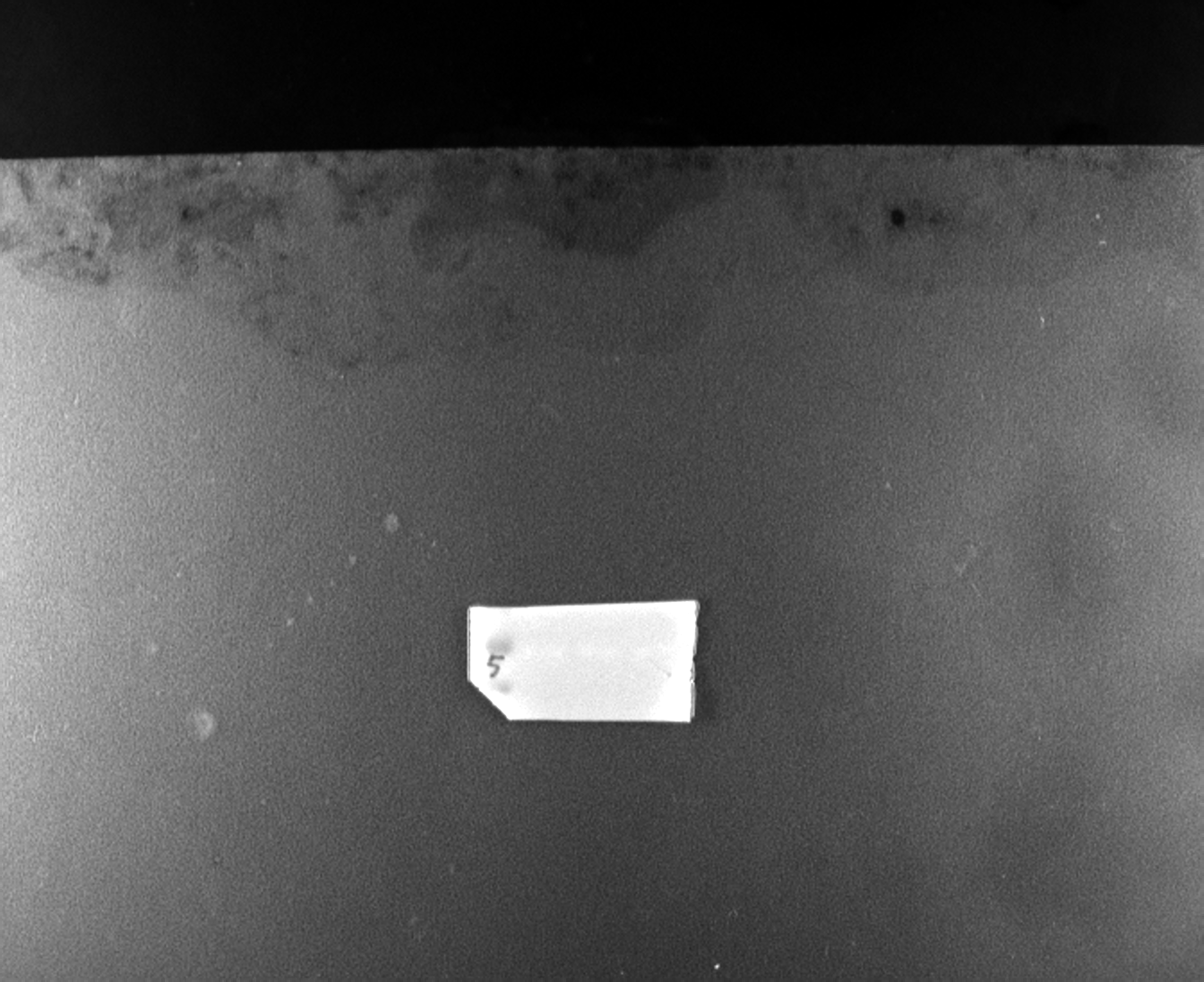

Supplement: Supplementary file 2 — Additional file 2: The raw experimental data related to this study. [file 12935_2022_2689_MOESM2_ESM.zip › WB/PAK1/87-2-2.Tif]

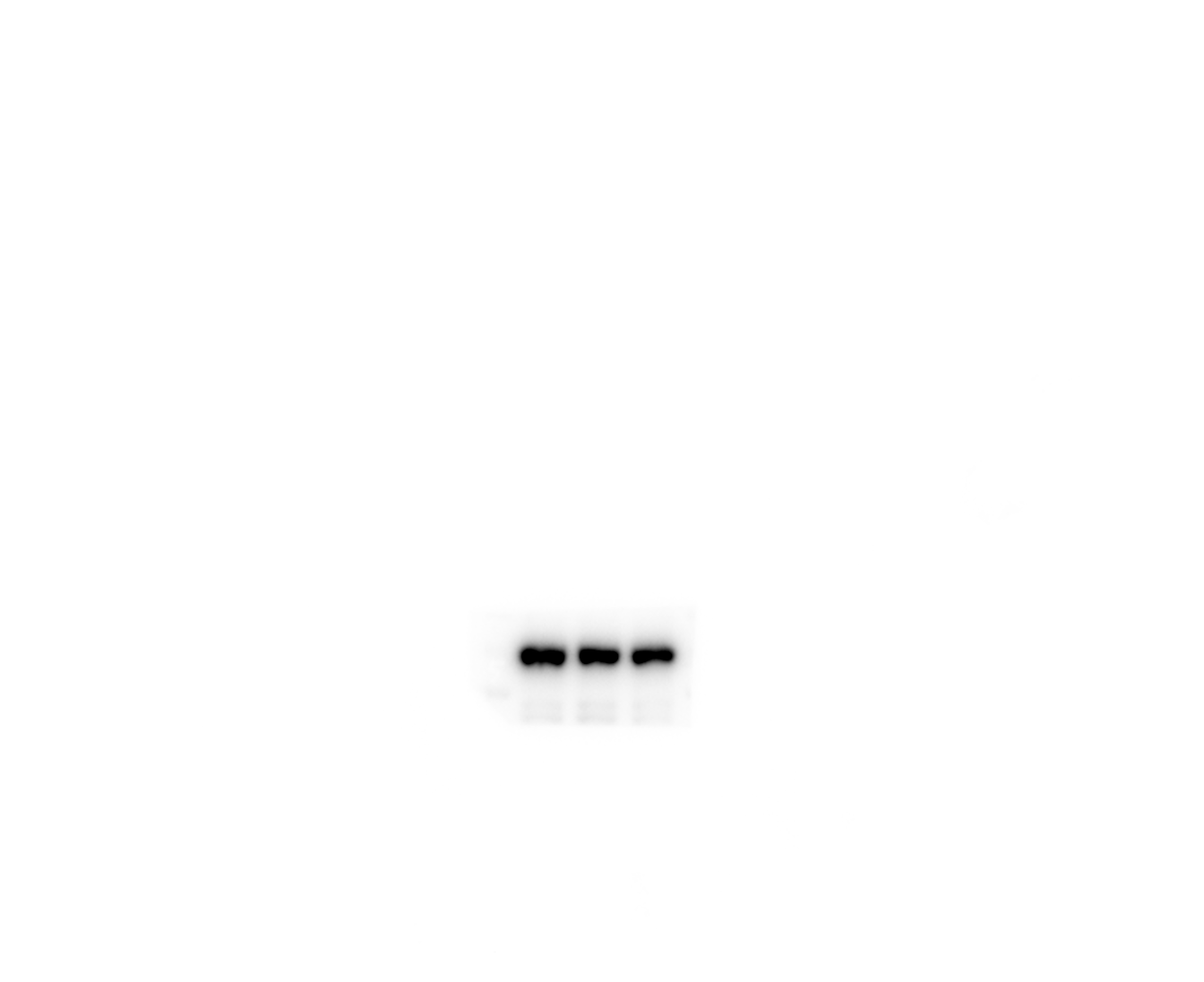

Supplement: Supplementary file 2 — Additional file 2: The raw experimental data related to this study. [file 12935_2022_2689_MOESM2_ESM.zip › WB/PAK1/87-2.Tif]

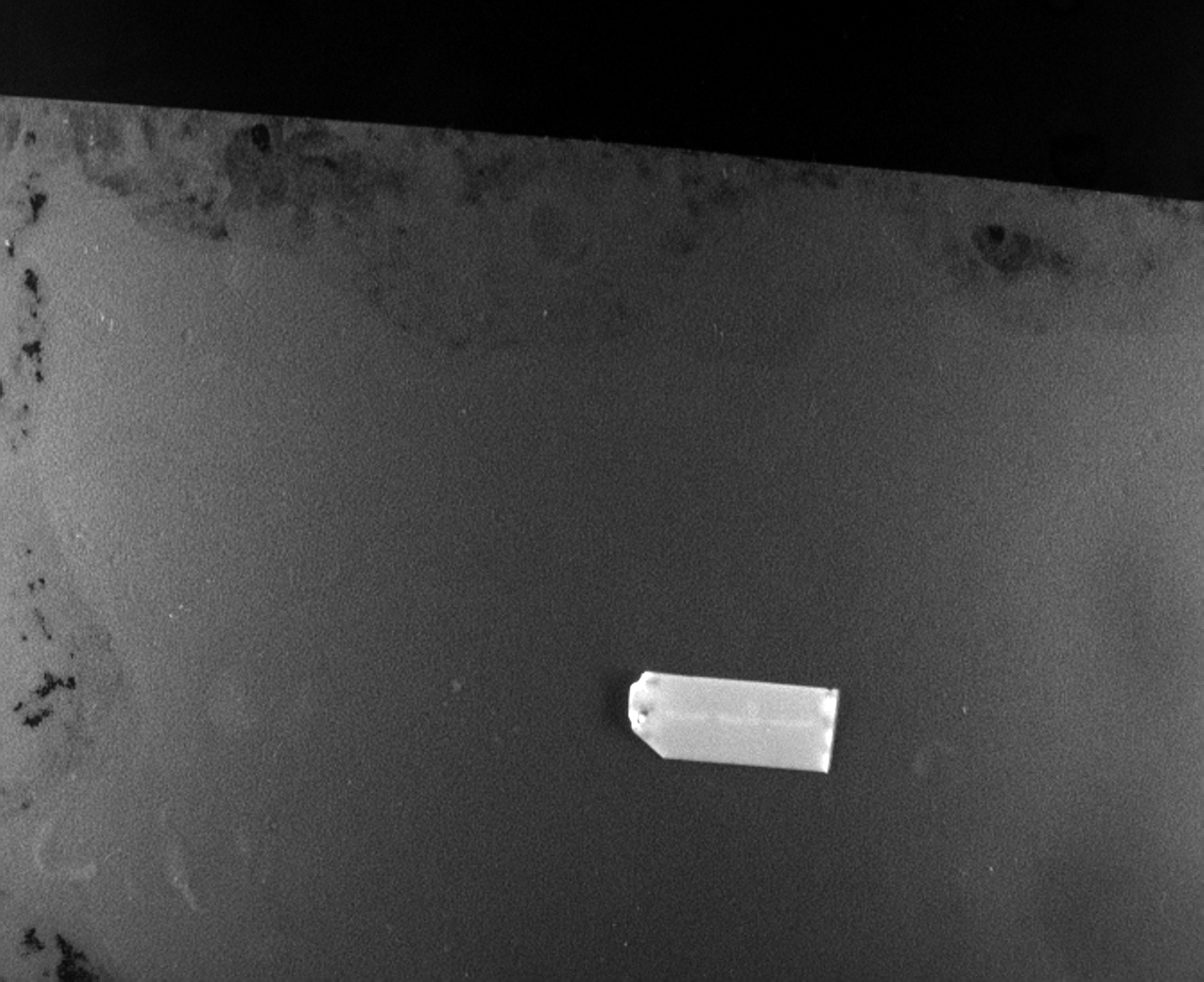

Supplement: Supplementary file 2 — Additional file 2: The raw experimental data related to this study. [file 12935_2022_2689_MOESM2_ESM.zip › WB/PAK1/87-3-2.Tif]

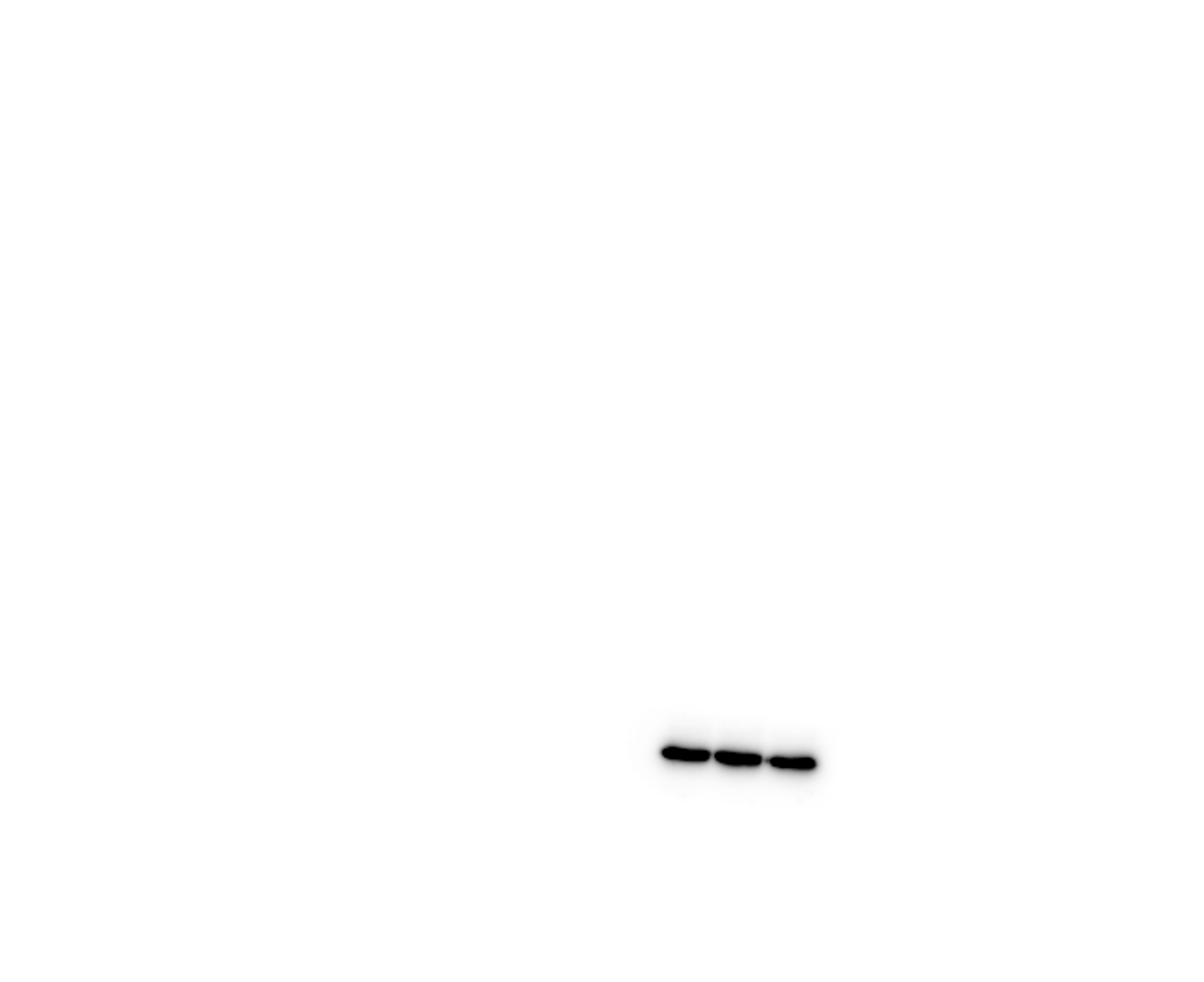

Supplement: Supplementary file 2 — Additional file 2: The raw experimental data related to this study. [file 12935_2022_2689_MOESM2_ESM.zip › WB/PAK1/87-3.Tif]

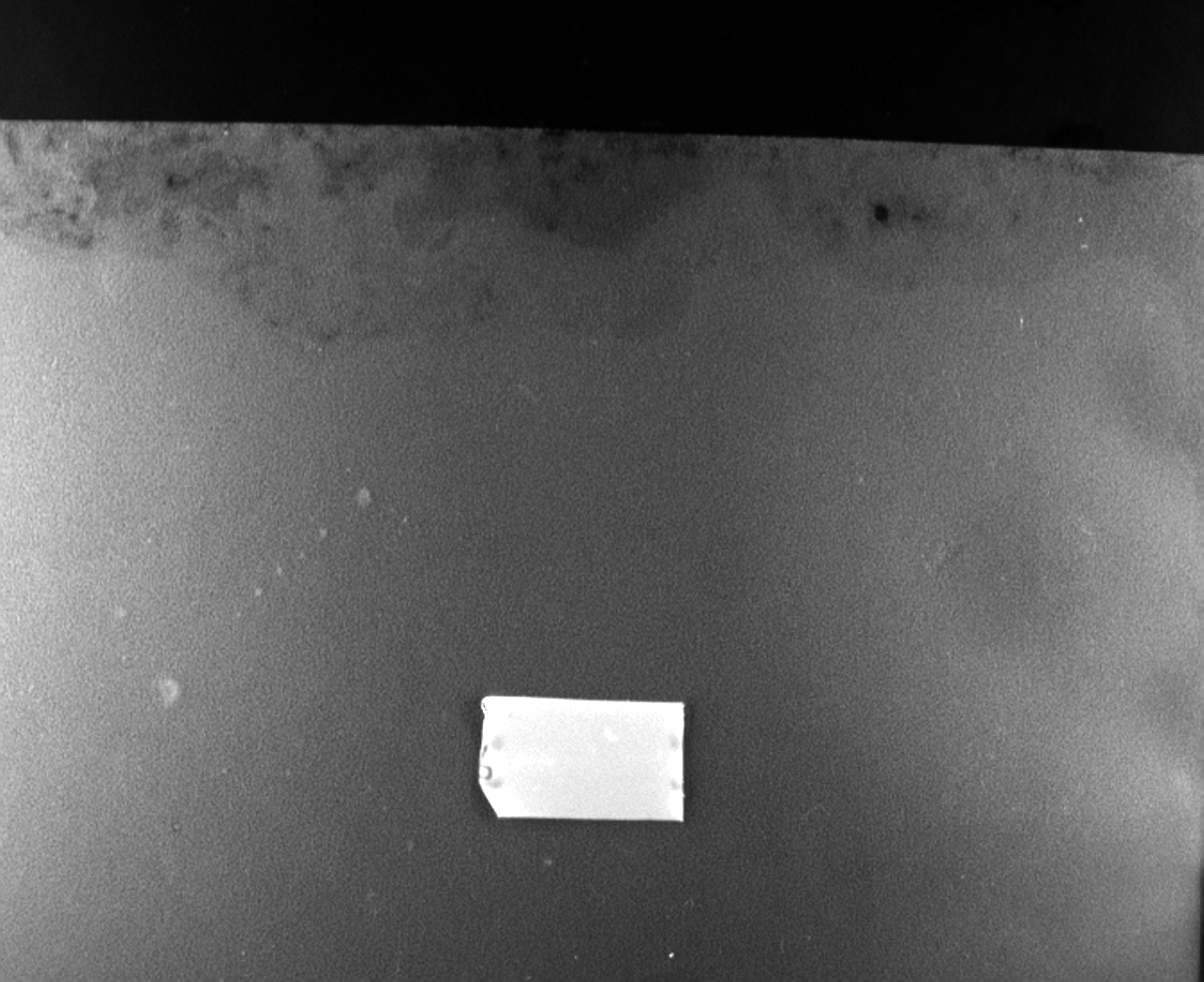

Supplement: Supplementary file 2 — Additional file 2: The raw experimental data related to this study. [file 12935_2022_2689_MOESM2_ESM.zip › WB/pAKT/229-1-2.Tif]

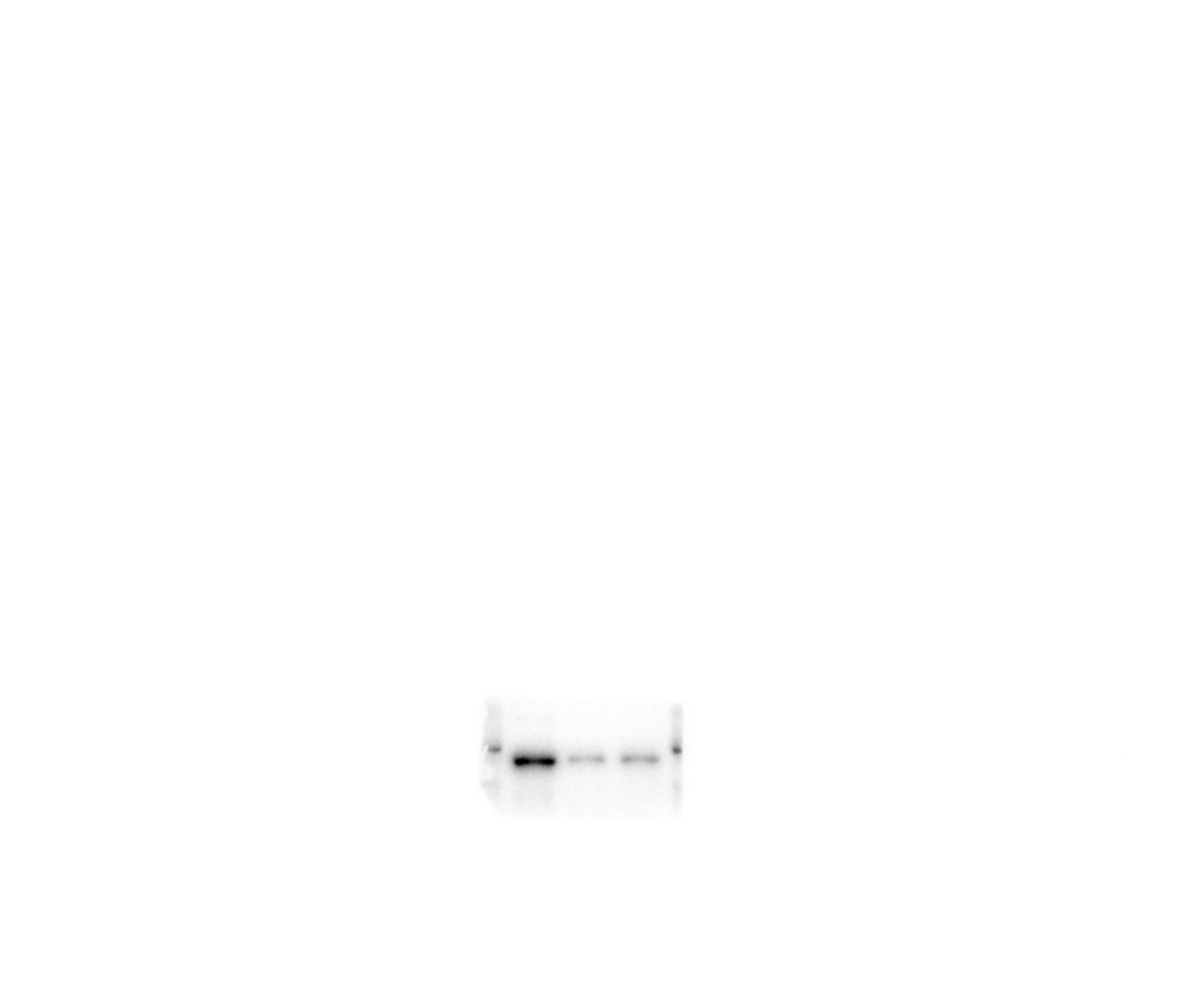

Supplement: Supplementary file 2 — Additional file 2: The raw experimental data related to this study. [file 12935_2022_2689_MOESM2_ESM.zip › WB/pAKT/229-1.Tif]

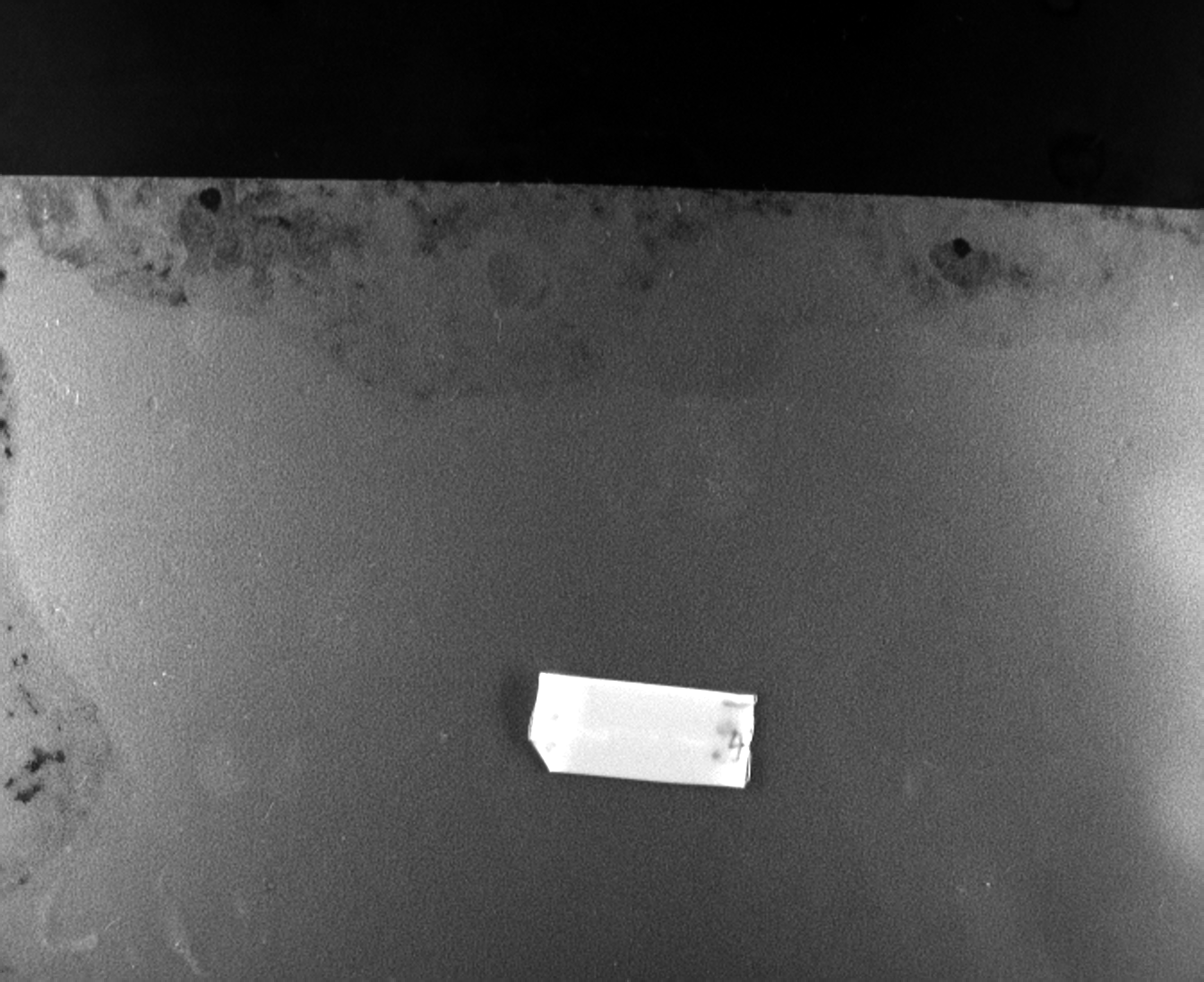

Supplement: Supplementary file 2 — Additional file 2: The raw experimental data related to this study. [file 12935_2022_2689_MOESM2_ESM.zip › WB/pAKT/229-2-2.Tif]

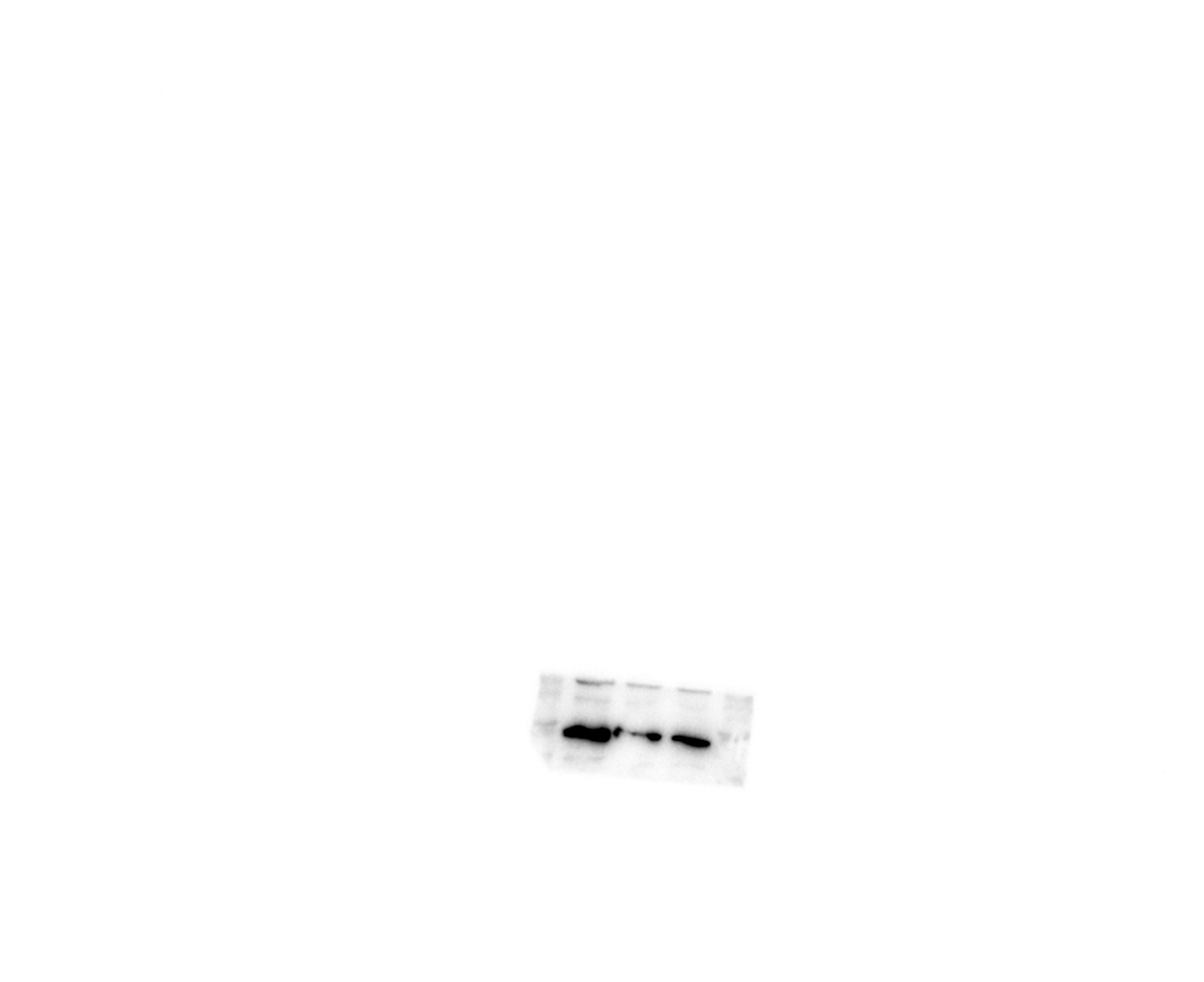

Supplement: Supplementary file 2 — Additional file 2: The raw experimental data related to this study. [file 12935_2022_2689_MOESM2_ESM.zip › WB/pAKT/229-2.Tif]

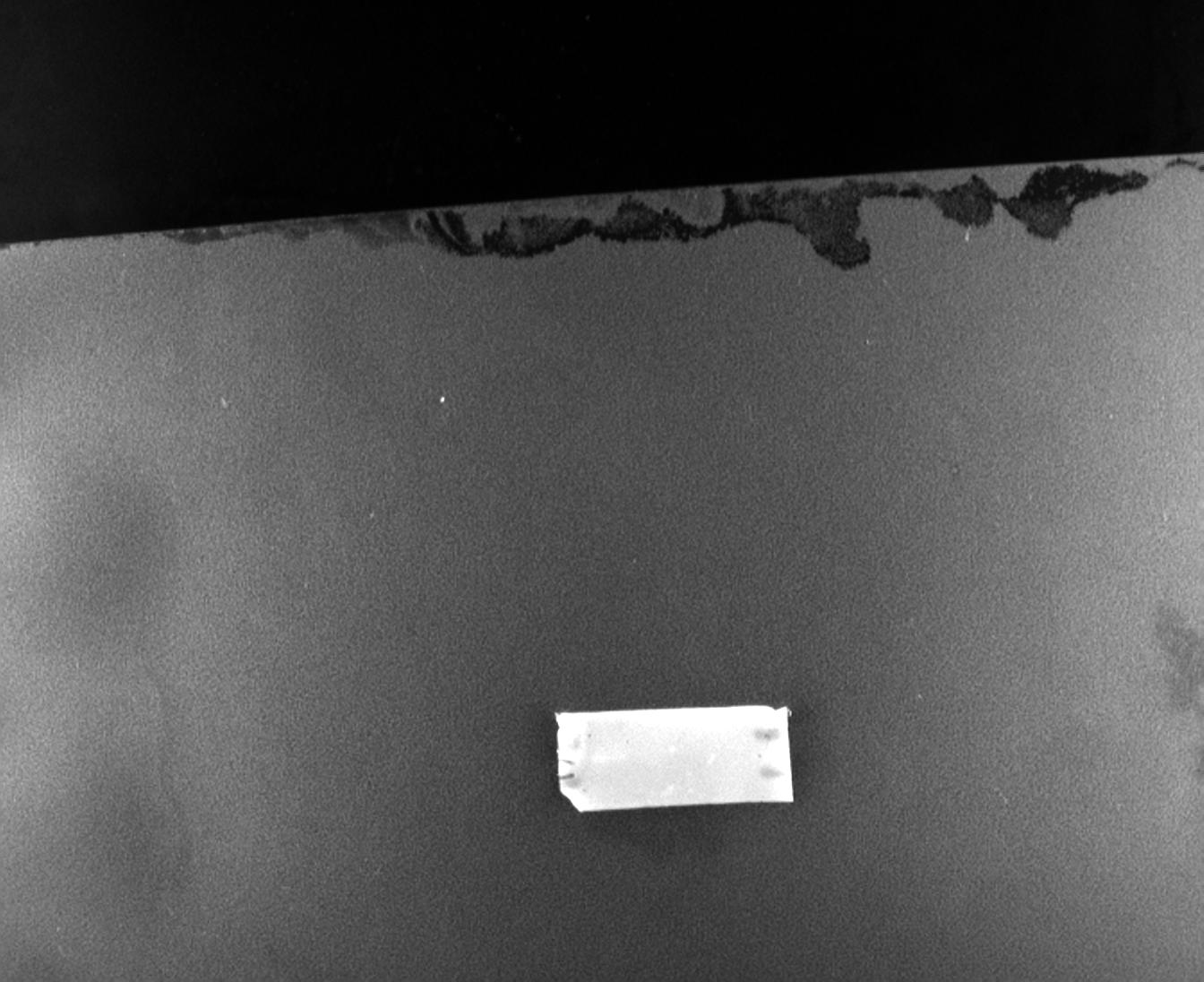

Supplement: Supplementary file 2 — Additional file 2: The raw experimental data related to this study. [file 12935_2022_2689_MOESM2_ESM.zip › WB/pAKT/229-3-2.Tif]

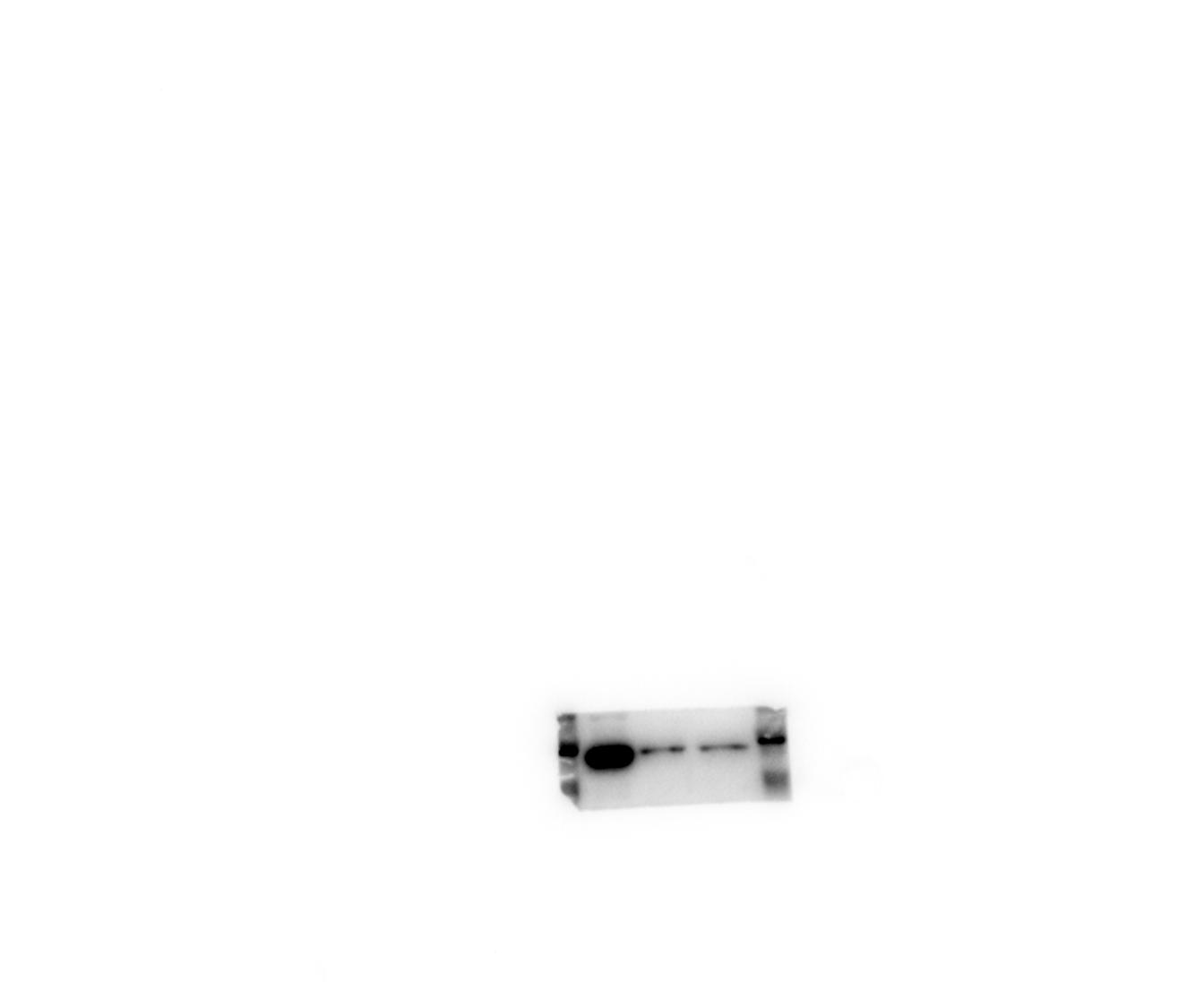

Supplement: Supplementary file 2 — Additional file 2: The raw experimental data related to this study. [file 12935_2022_2689_MOESM2_ESM.zip › WB/pAKT/229-3.Tif]

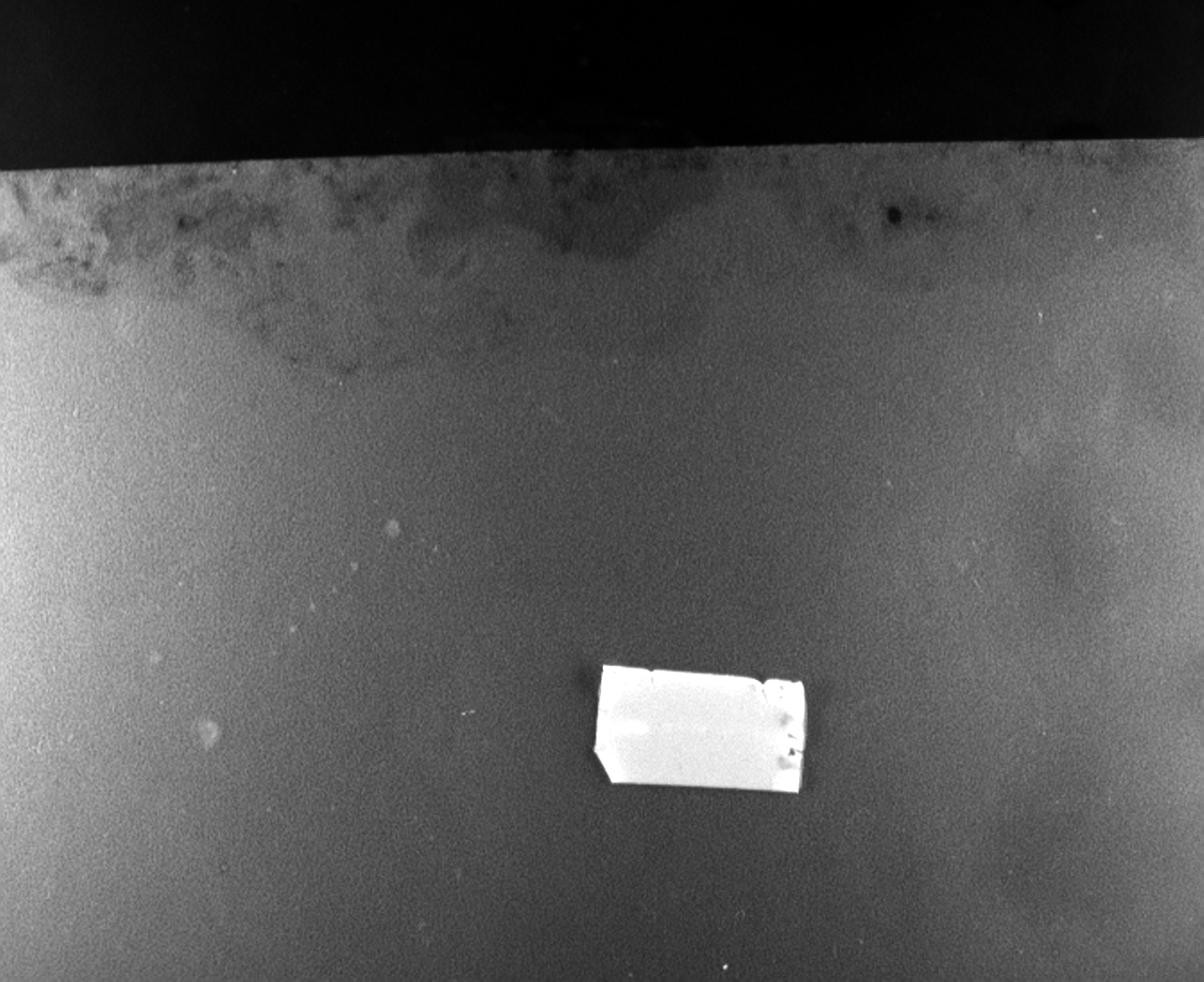

Supplement: Supplementary file 2 — Additional file 2: The raw experimental data related to this study. [file 12935_2022_2689_MOESM2_ESM.zip › WB/pAKT/87-1-2.Tif]

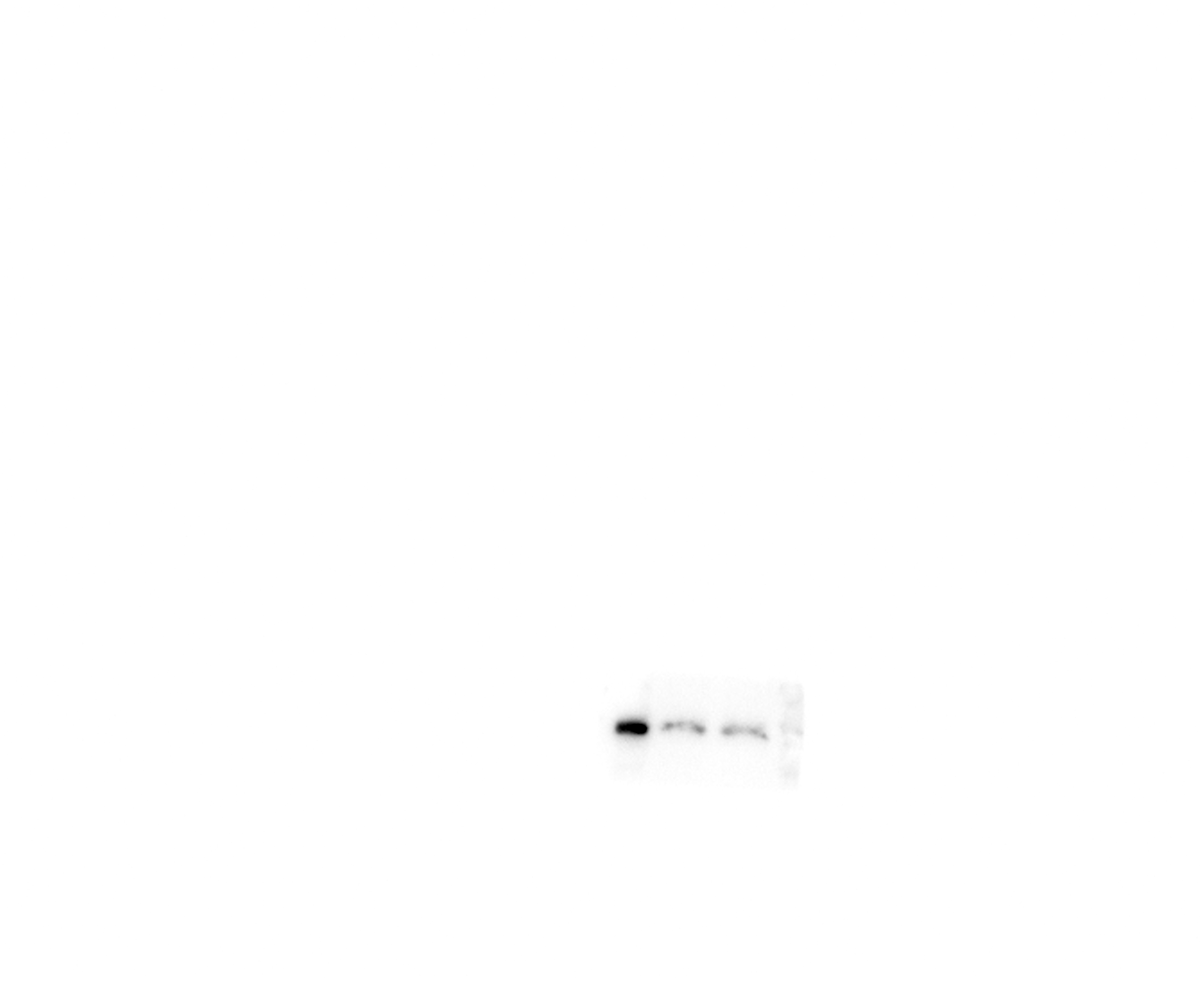

Supplement: Supplementary file 2 — Additional file 2: The raw experimental data related to this study. [file 12935_2022_2689_MOESM2_ESM.zip › WB/pAKT/87-1.Tif]

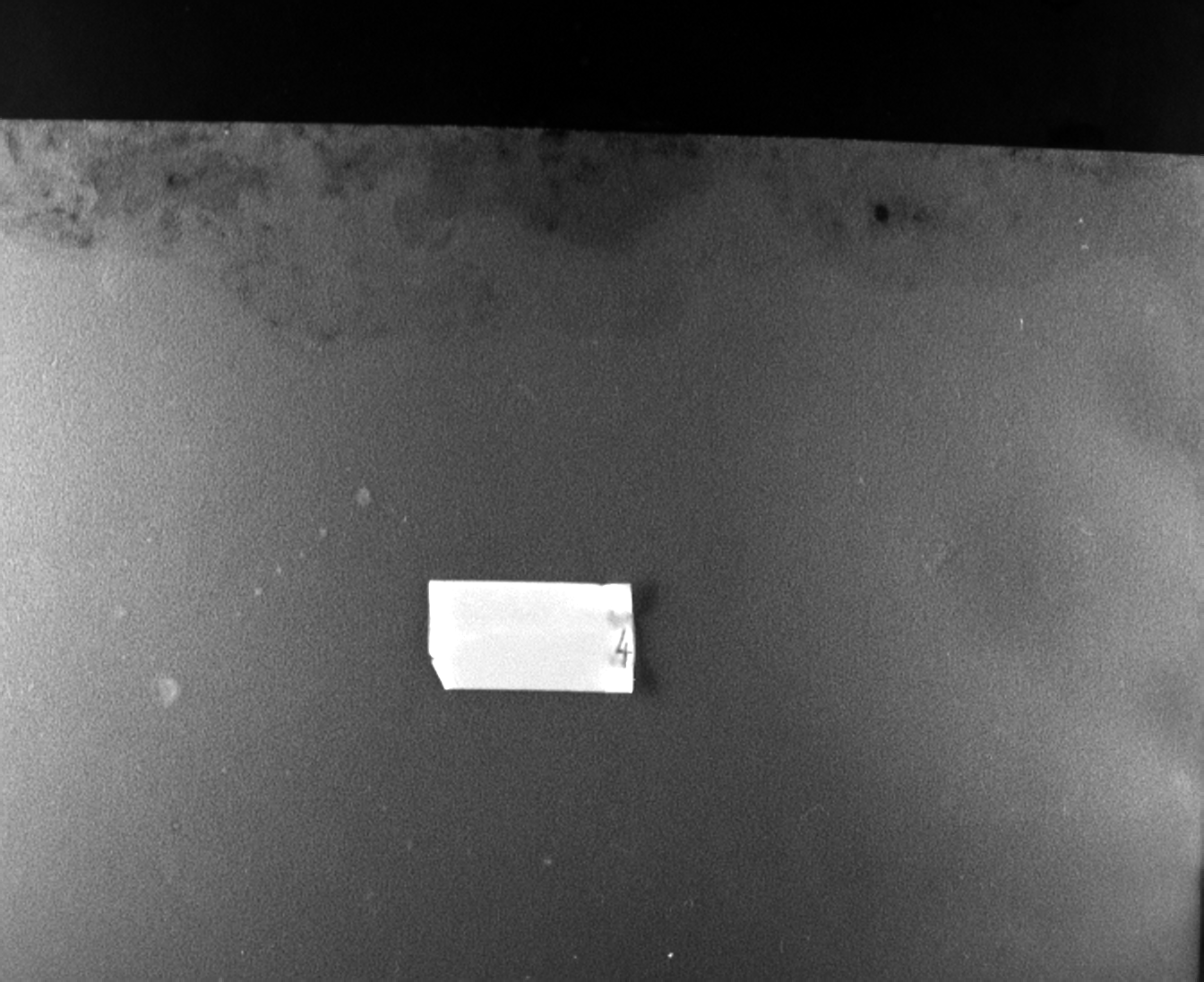

Supplement: Supplementary file 2 — Additional file 2: The raw experimental data related to this study. [file 12935_2022_2689_MOESM2_ESM.zip › WB/pAKT/87-2-2.Tif]

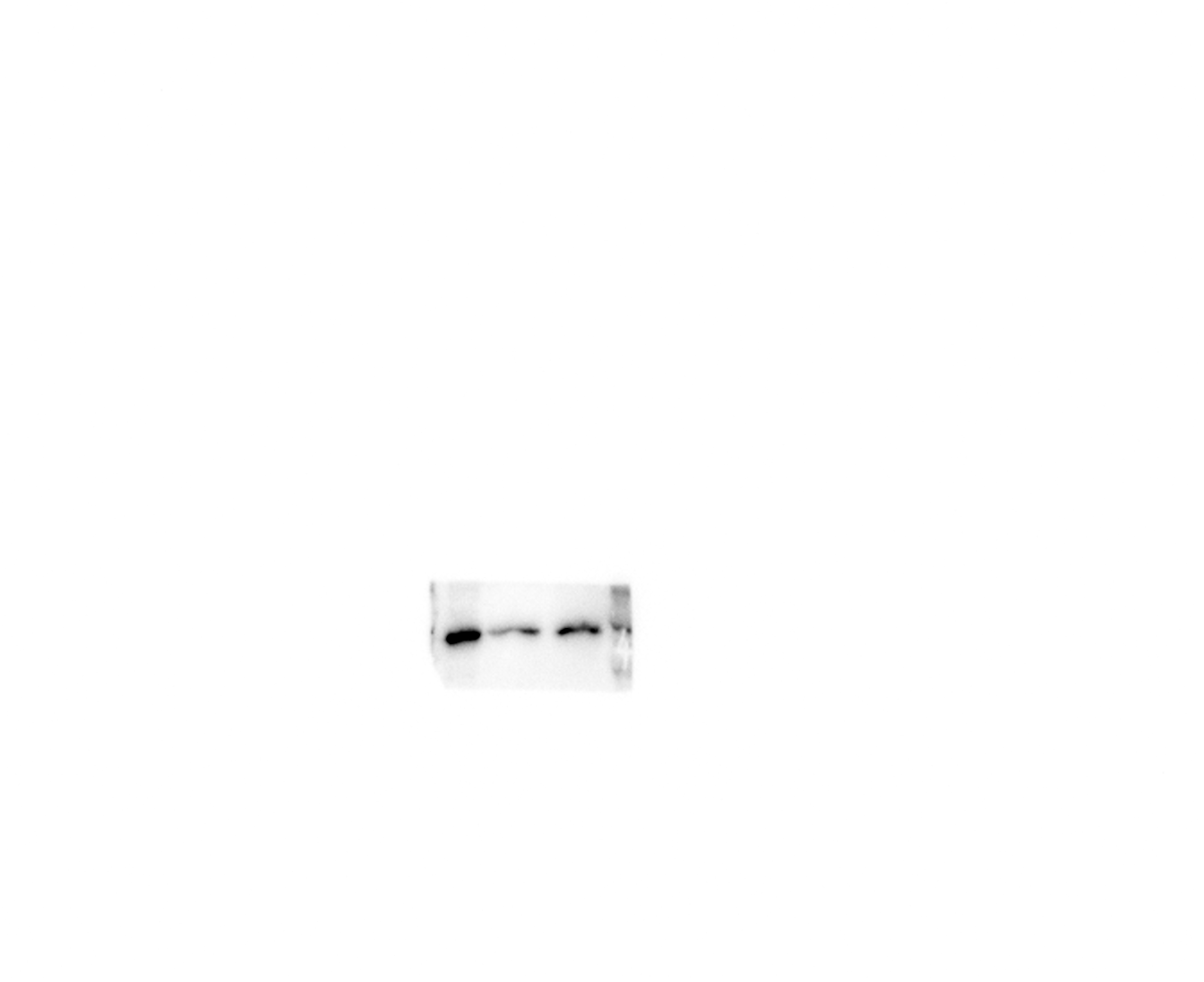

Supplement: Supplementary file 2 — Additional file 2: The raw experimental data related to this study. [file 12935_2022_2689_MOESM2_ESM.zip › WB/pAKT/87-2.Tif]

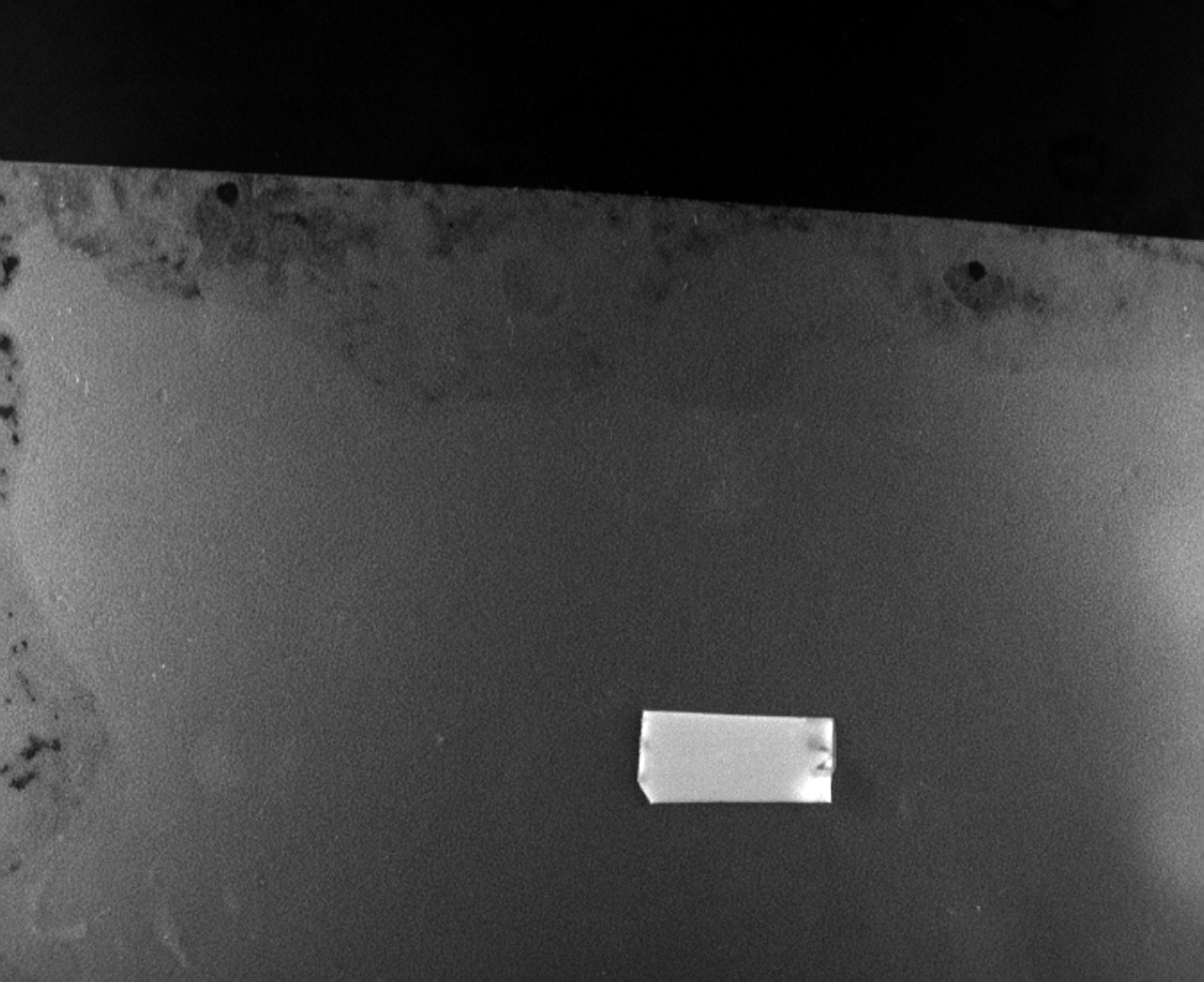

Supplement: Supplementary file 2 — Additional file 2: The raw experimental data related to this study. [file 12935_2022_2689_MOESM2_ESM.zip › WB/pAKT/87-3-2.Tif]

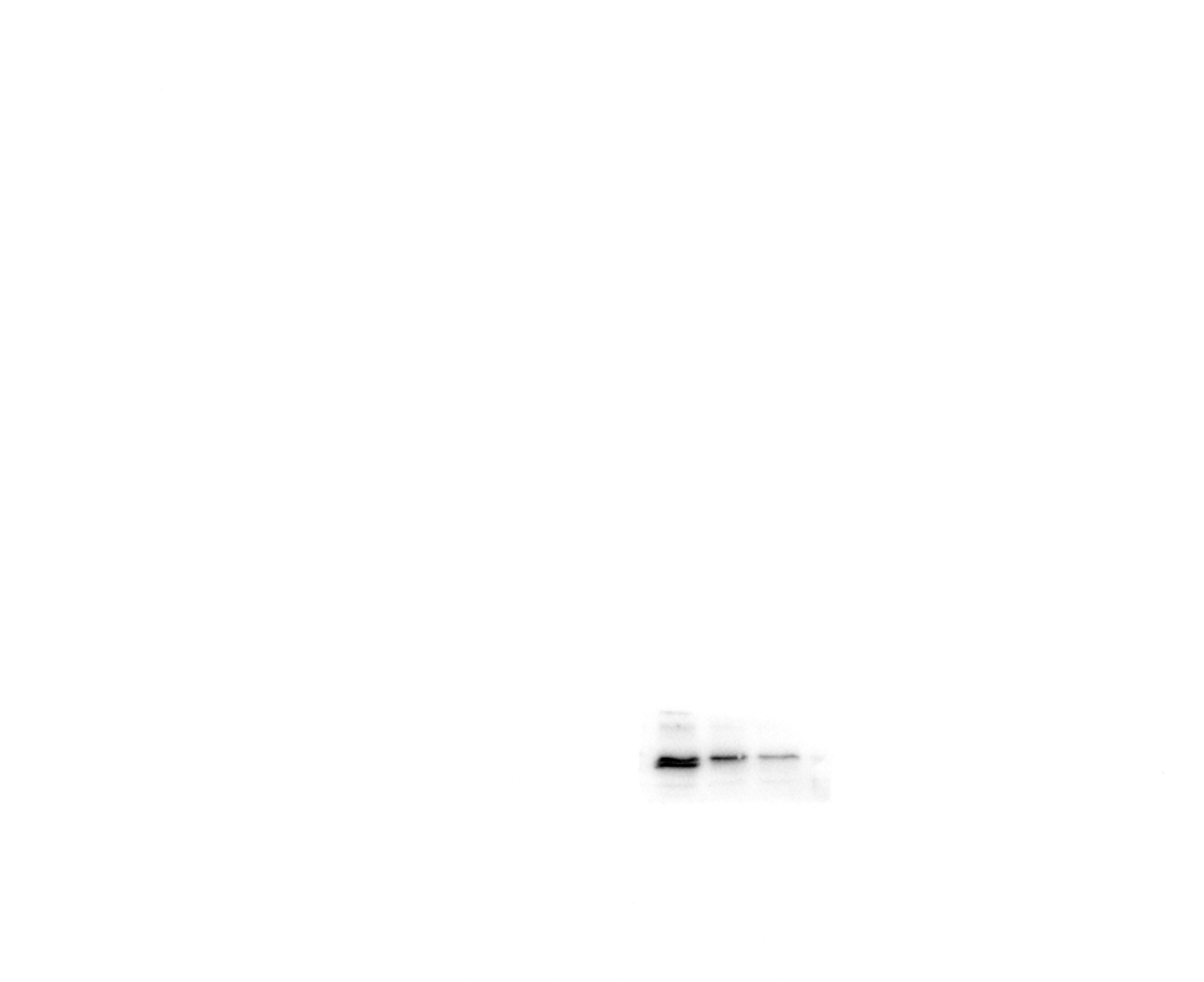

Supplement: Supplementary file 2 — Additional file 2: The raw experimental data related to this study. [file 12935_2022_2689_MOESM2_ESM.zip › WB/pAKT/87-3.Tif]

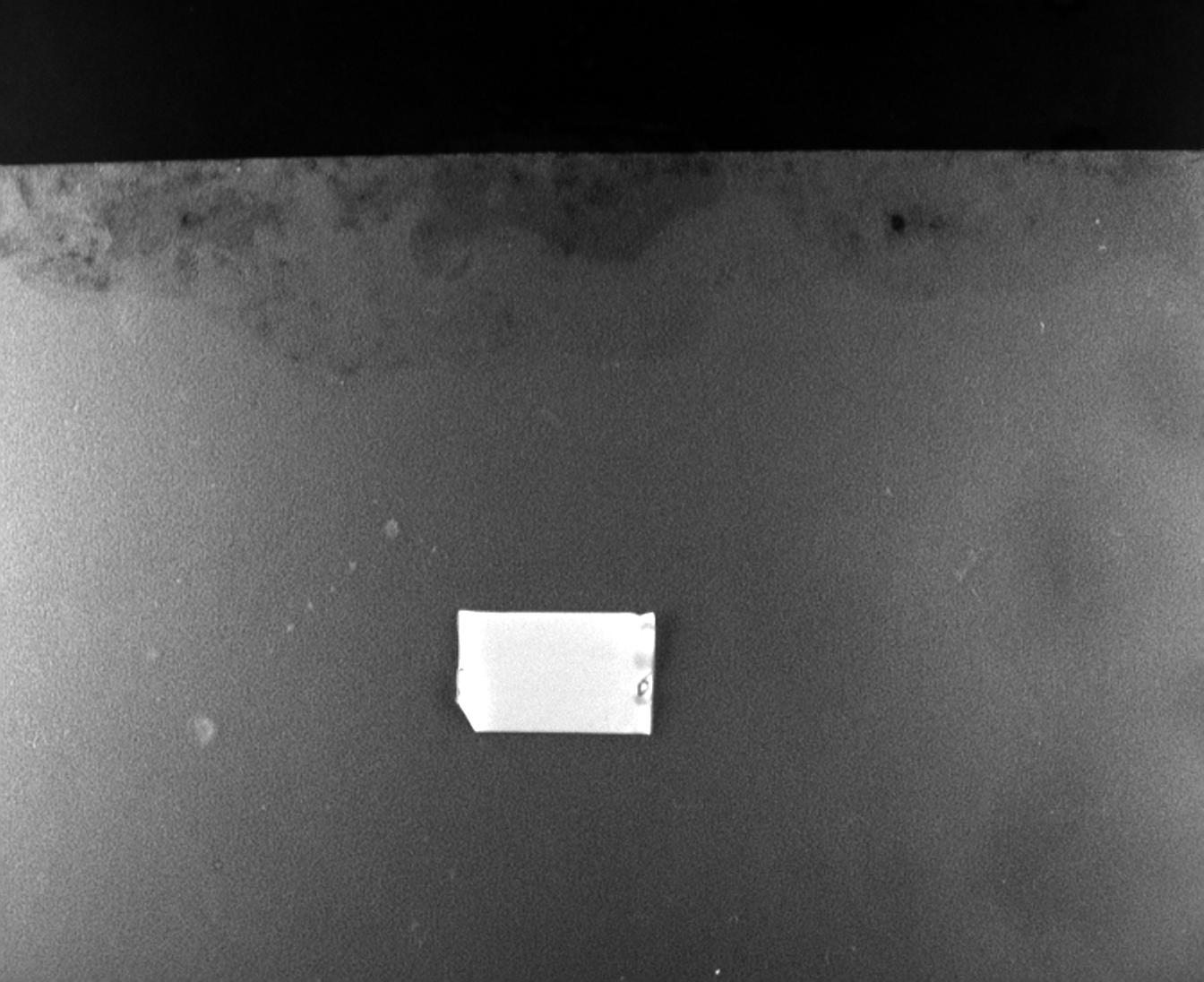

Supplement: Supplementary file 2 — Additional file 2: The raw experimental data related to this study. [file 12935_2022_2689_MOESM2_ESM.zip › WB/pPAK/229-1-2.Tif]

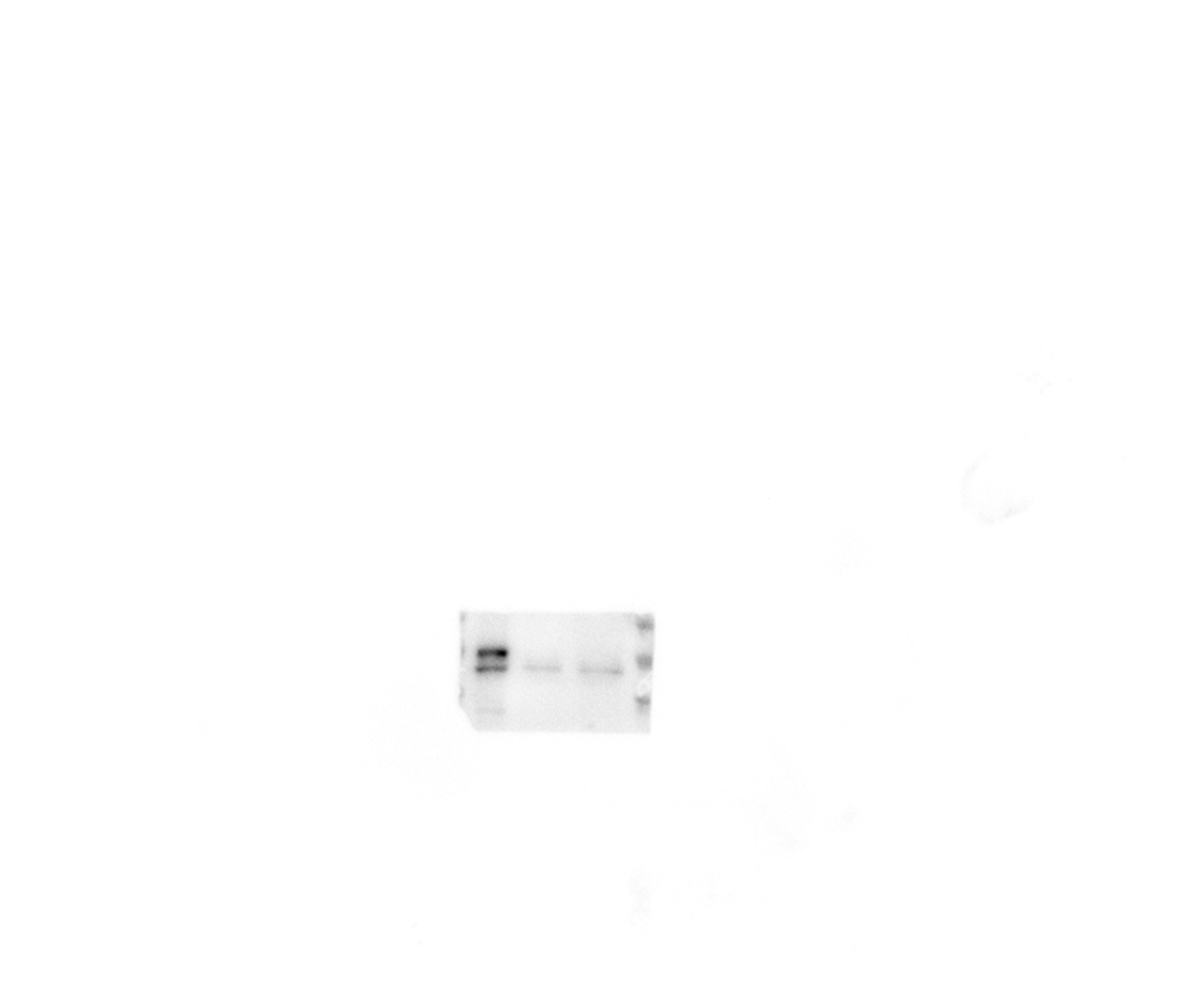

Supplement: Supplementary file 2 — Additional file 2: The raw experimental data related to this study. [file 12935_2022_2689_MOESM2_ESM.zip › WB/pPAK/229-1.Tif]

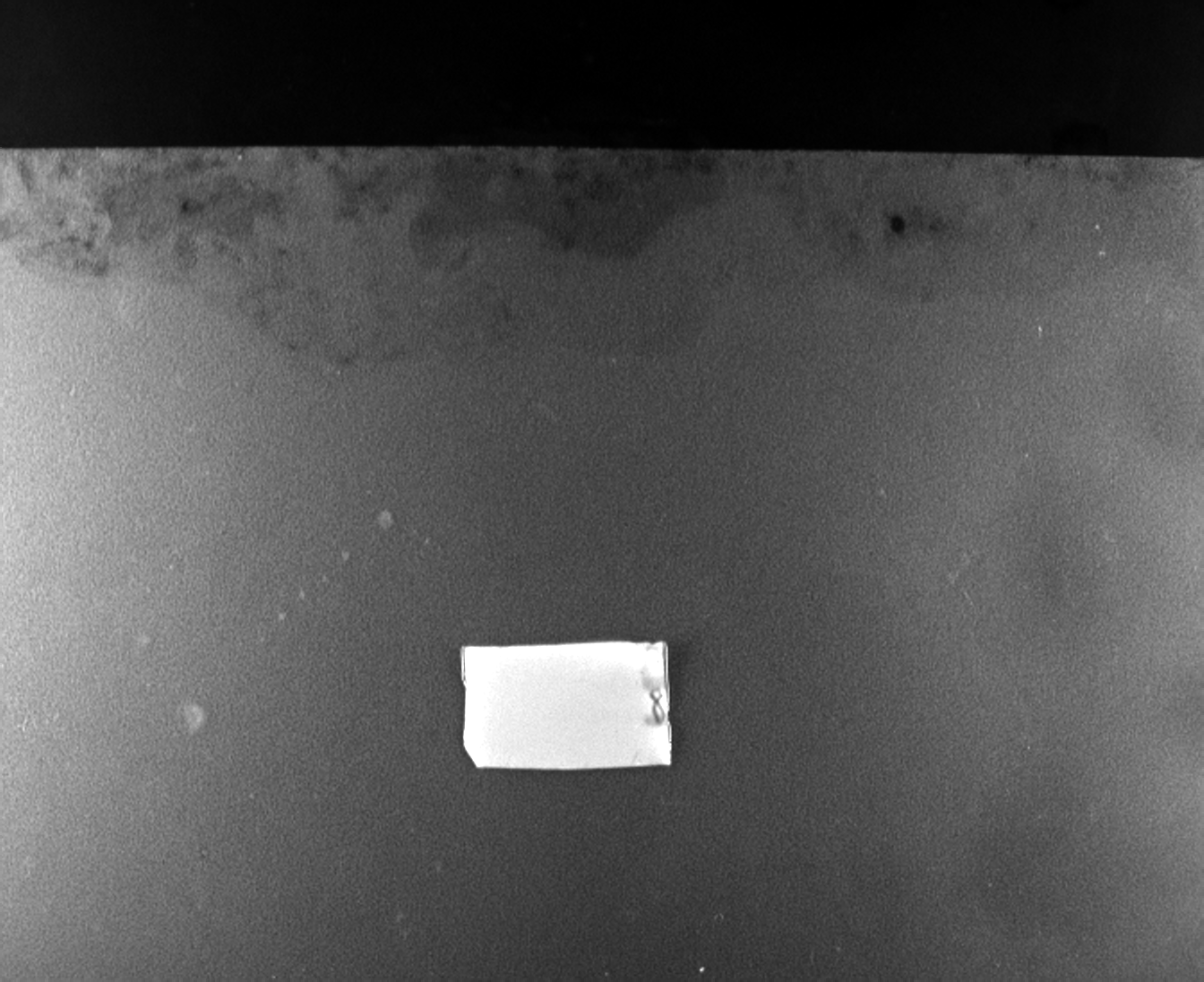

Supplement: Supplementary file 2 — Additional file 2: The raw experimental data related to this study. [file 12935_2022_2689_MOESM2_ESM.zip › WB/pPAK/229-2-2.Tif]

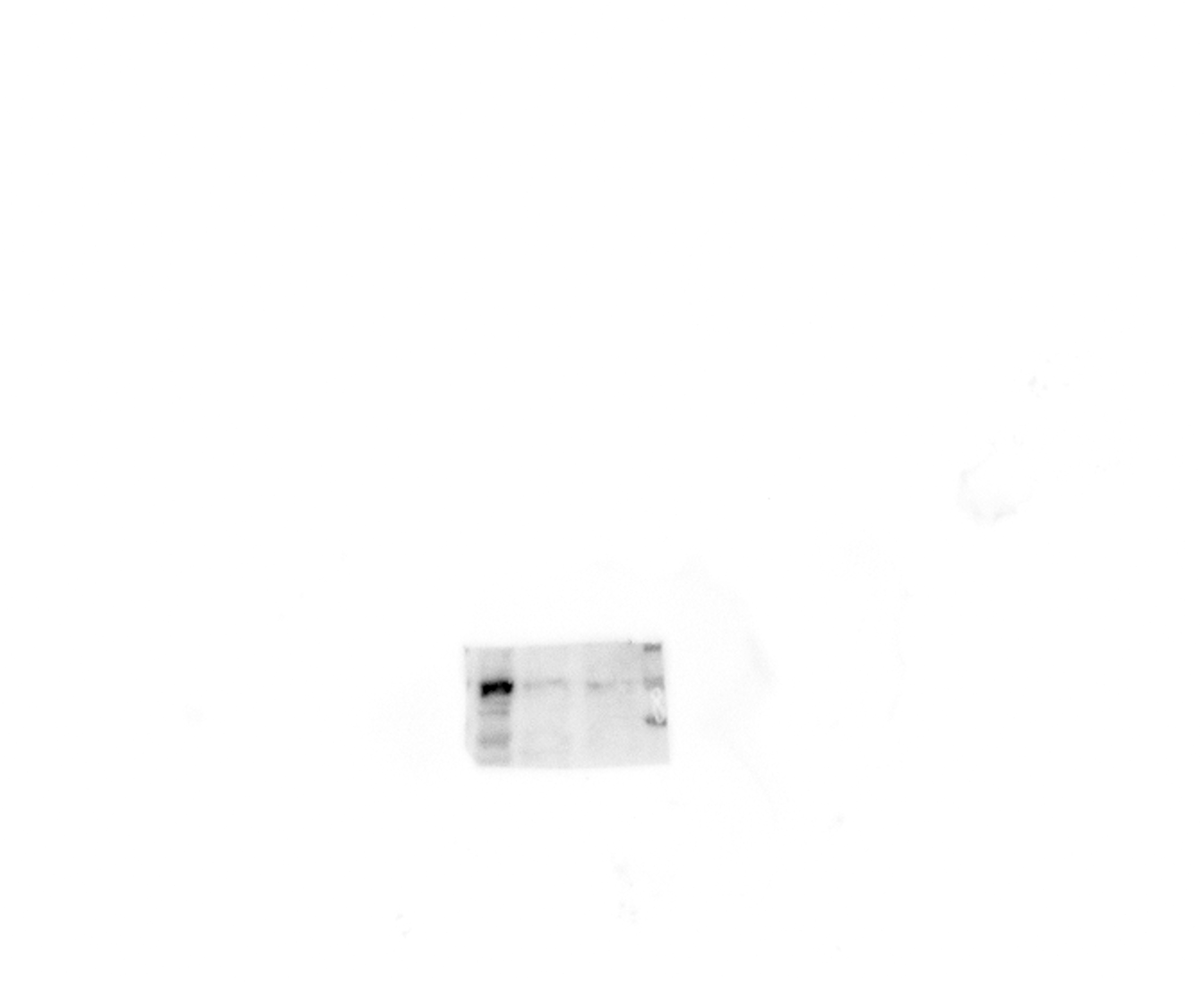

Supplement: Supplementary file 2 — Additional file 2: The raw experimental data related to this study. [file 12935_2022_2689_MOESM2_ESM.zip › WB/pPAK/229-2.Tif]

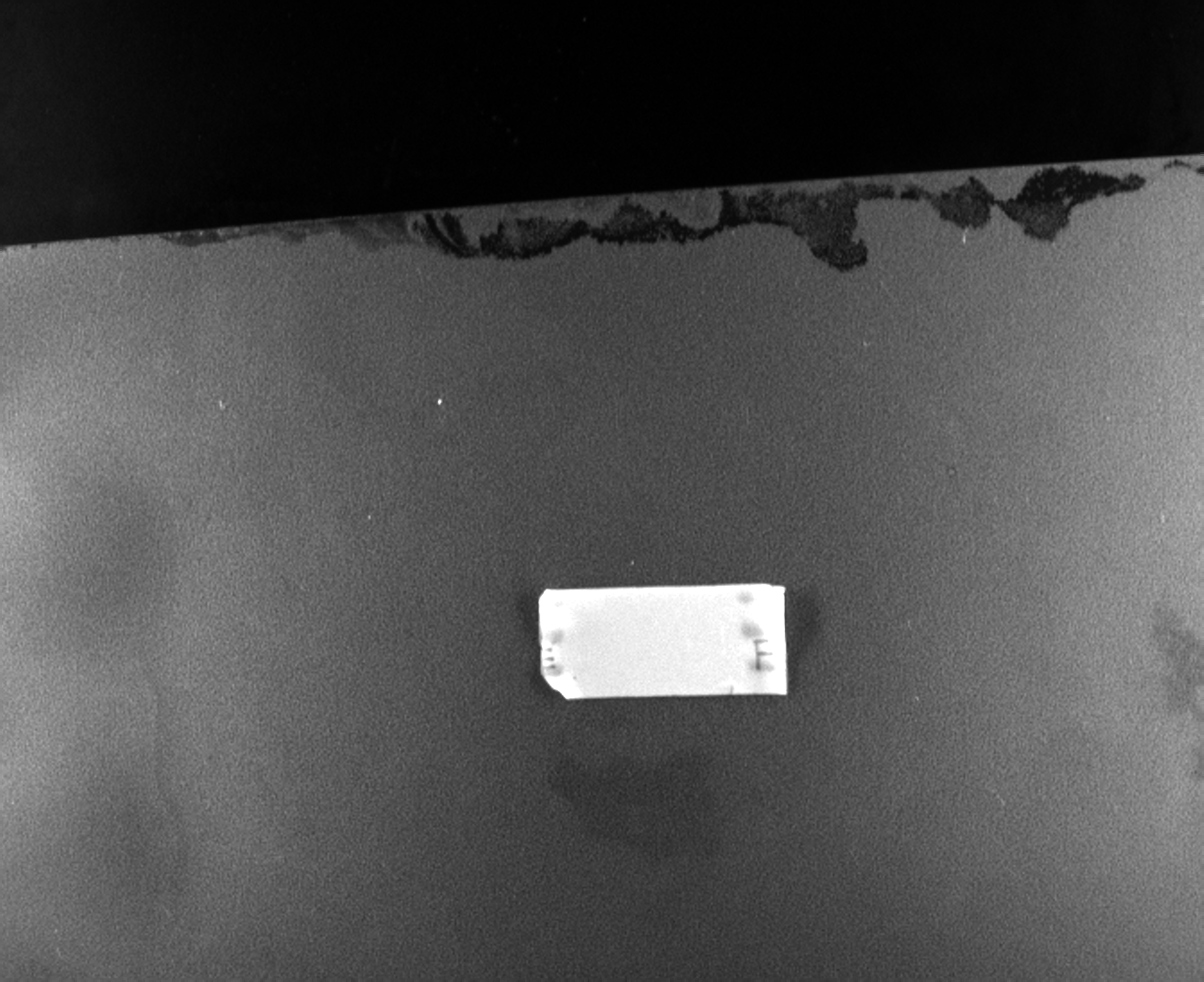

Supplement: Supplementary file 2 — Additional file 2: The raw experimental data related to this study. [file 12935_2022_2689_MOESM2_ESM.zip › WB/pPAK/229-3-2.Tif]

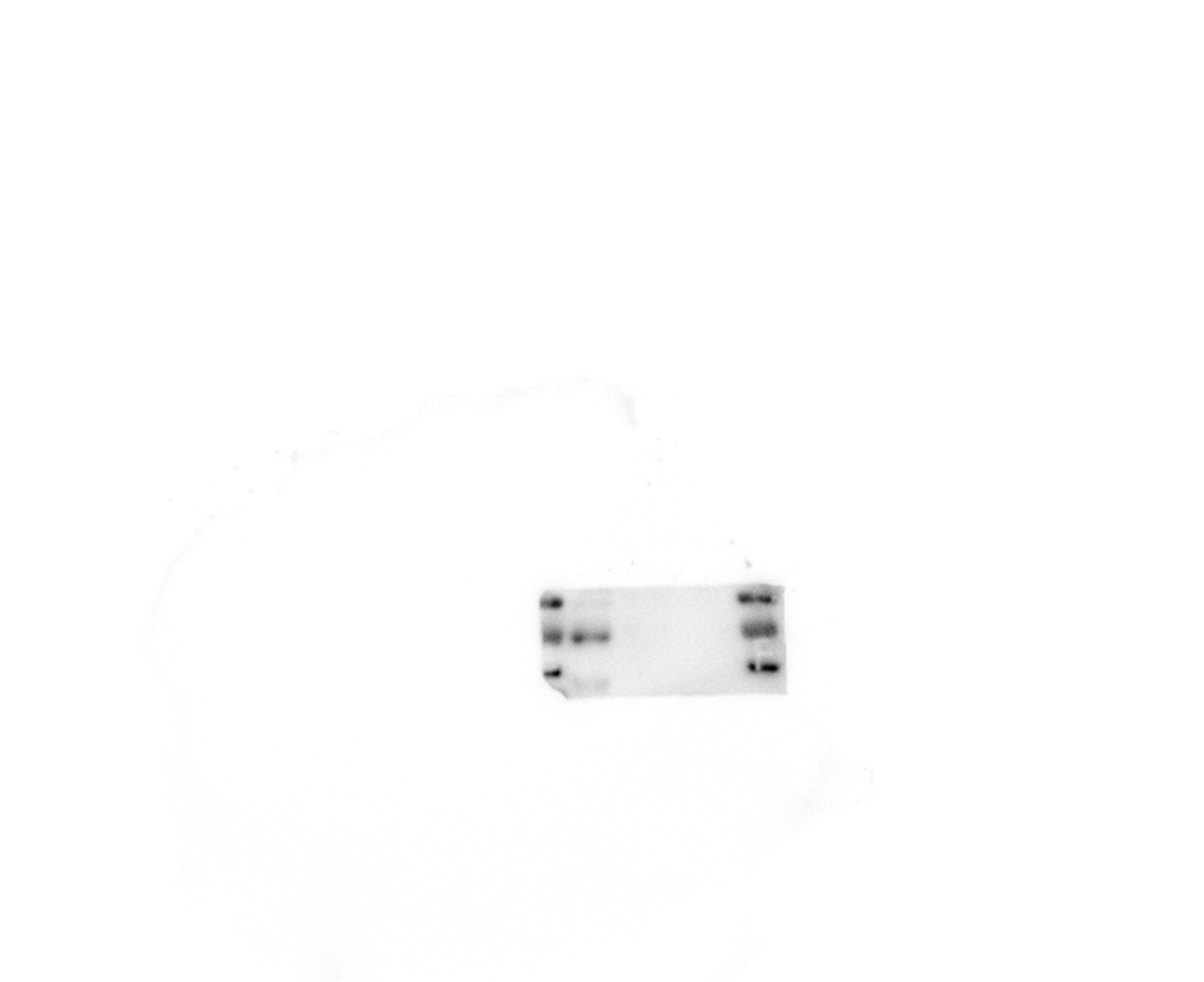

Supplement: Supplementary file 2 — Additional file 2: The raw experimental data related to this study. [file 12935_2022_2689_MOESM2_ESM.zip › WB/pPAK/229-3.Tif]

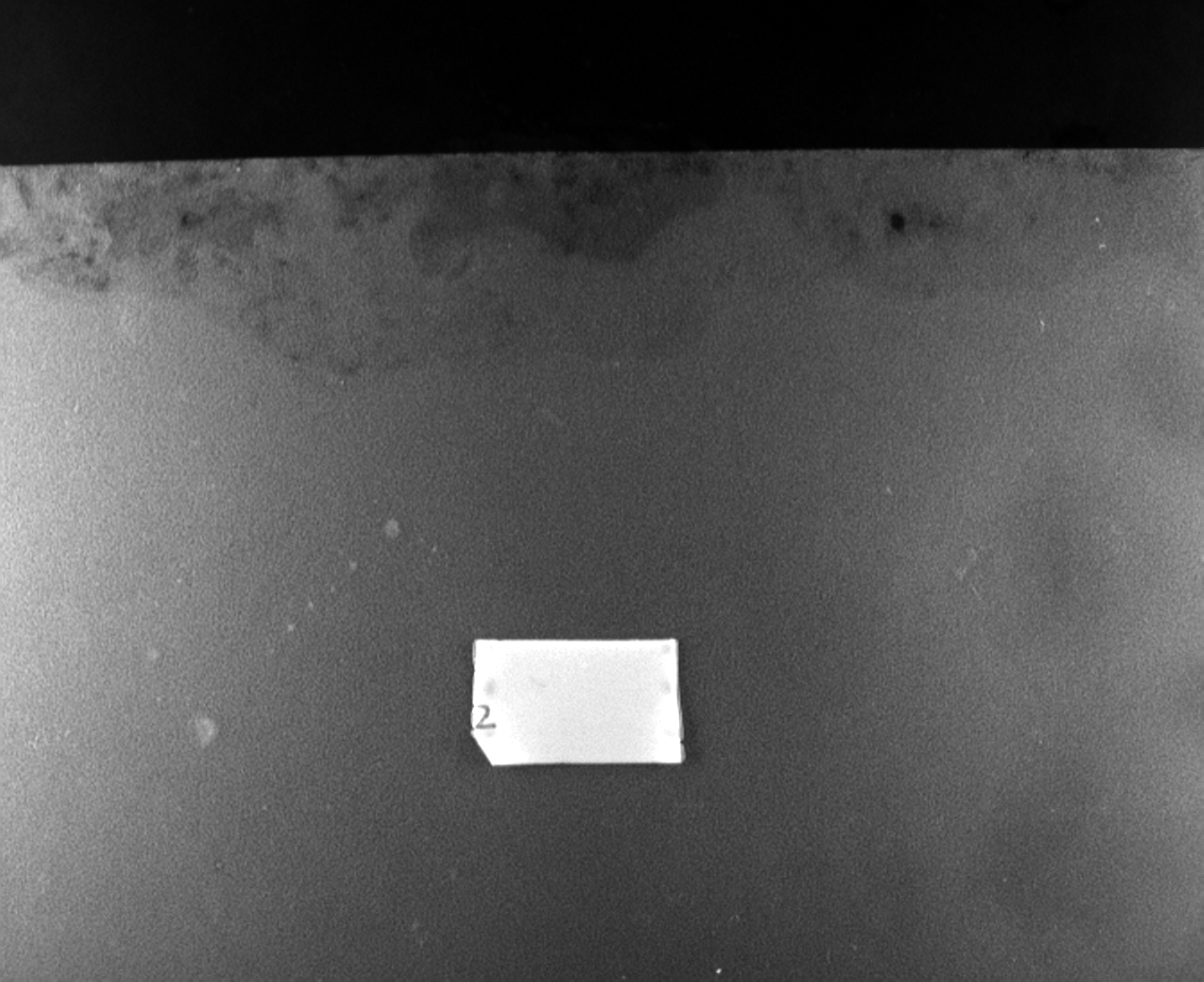

Supplement: Supplementary file 2 — Additional file 2: The raw experimental data related to this study. [file 12935_2022_2689_MOESM2_ESM.zip › WB/pPAK/87-1-2.Tif]

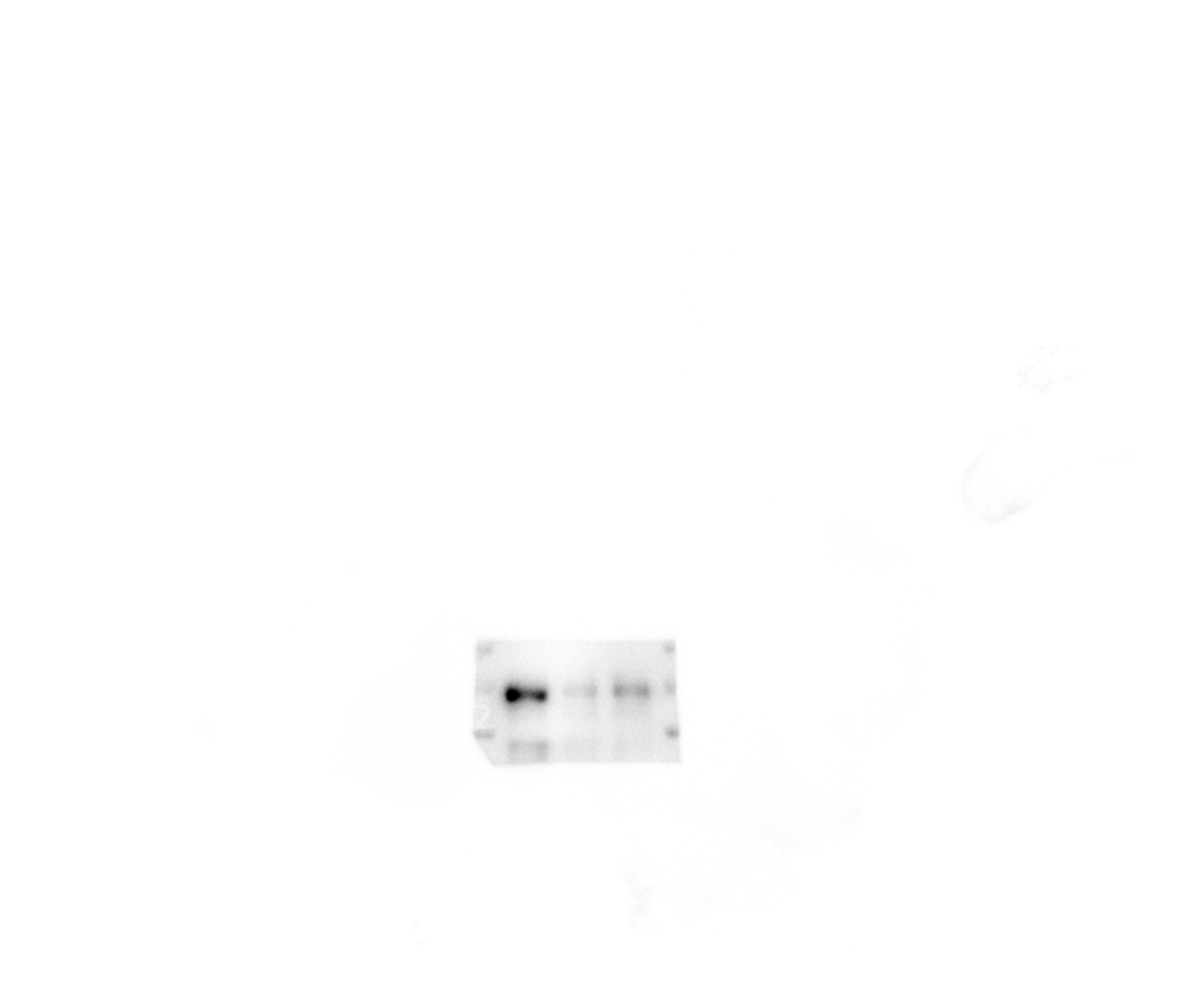

Supplement: Supplementary file 2 — Additional file 2: The raw experimental data related to this study. [file 12935_2022_2689_MOESM2_ESM.zip › WB/pPAK/87-1.Tif]
